# The current state of polygenic scores for the development of lung cancer: a systematic review and validation in UK Biobank

**DOI:** 10.1038/s41416-025-03330-9

**Published:** 2026-01-08

**Authors:** Bayan Galal, Joe Dennis, Antonis C. Antoniou, Hannah Harrison

**Affiliations:** 1 Perelman School of Medicine, University of Pennsylvania, Philadelphia, PA USA; 2 Centre for Cancer Genetic Epidemiology, Department of Public Health and Primary Care, University of Cambridge, Cambridge, UK

**Keywords:** Risk factors, Lung cancer

## Abstract

**Background:**

Risk-stratified lung cancer screening programs identify high-risk individuals who use tobacco but do not account for underlying genetic susceptibility. Many polygenic scores (PGS) have been developed for lung cancer, but it is unclear which, if any, are suitable for identifying high-risk individuals in the general population.

**Methods:**

We used a systematic review to identify published lung cancer PGS, which were implemented and validated in the UK Biobank (UKB) cohort. Performance (discrimination and accuracy) was compared. Subgroup analyses by sex, ethnicity, and smoking status identified differences across the population.

**Results:**

We identified 60 lung cancer PGS published since 2012. Most scores were associated with lung cancer risk in UKB. Of the 39 evaluated PGS, 33 had a hazard ratio per standard deviation greater than 1.1 and 22 had a C-index greater than 0.55. Most PGS perform better in individuals who use tobacco than those who do not, although for a small number of scores (*n* = 8) the reverse is true.

**Discussion:**

Performance of lung cancer PGS is weak compared to scores for other cancers; the potential benefit of combining genetics with other risk factors for lung cancer remains unclear. Selection of a suitable score is context dependent and requires consideration of the characteristics of the target population (such as ethnicity and tobacco usage).

## Introduction

Lung cancer remains the leading cause of cancer-related deaths worldwide [1], in the United Kingdom (UK) there are 45,000 incident cases and 35,000 deaths annually [2]. Lung cancer is often asymptomatic in the early stages, which contributes to the high number of late stage diagnoses; in 2018, 65% of lung cancer diagnoses in the UK were either at Stage III or IV [3]. Survival rates for lung cancer vary significantly depending on the stage at diagnosis. Approximately 5% of patients diagnosed at stage IV survive for 5 years or more after diagnosis, compared to around 65% of those diagnosed at stage I [4].

Screening with low-dose computed tomography (LDCT) aims to detect lung cancer at earlier stages when the possibility of curative treatment is greater, and it is a proven method of reducing mortality [5]. LDCT’s effectiveness has been demonstrated in large randomized controlled trials, including the National Lung Screening Trial (NLST) in the United States and the Dutch-Belgian NELSON study [6]. Following pilot studies [6, 7], the UK National Screening Committee has recommended a national lung cancer screening program for high-risk individuals who use tobacco [8, 9]. In these trials, risk stratification protocols have been used to select these individuals for screening [10, 11]. However, this approach restricts screening to those who are at high risk due to lifestyle risk factors and does not consider all individuals (such as individuals who do not smoke) who may have an underlying susceptibility to lung cancer.

Polygenic scores (PGS) estimate the likelihood of developing a disease by aggregating the small, cumulative effects of single nucleotide polymorphisms (SNPs), typically identified through genome-wide association studies (GWAS) [12–15]. The increased availability of large-scale genetic datasets in the last 10 years has led to a considerable increase in the number of SNPs known to be associated with lung cancer development [16]. Several PGS have been developed for lung cancer, with moderate performance shown in some validation studies (C-index>0.65) [14]. However, it remains challenging to select the most suitable PGS for a specific population [17]. There is known variation in how PGS are developed and in the populations used (both to identify variants and to evaluate the scores). A recent head-to-head validation of nine lung cancer PGS in a cohort of individuals who smoke (*n* = 550) found that all PGS were associated with lung cancer risk, and the best-performing PGS had an AUC of 0.59 [18].

To date, there has been no comprehensive identification of all existing PGS for the development of lung cancer and no study has compared their performance in a general population cohort (including individuals who do and do not smoke). In this study, we use a systematic review approach to identify a comprehensive set of previously published lung cancer PGS, which we then validate in the UK Biobank (UKB) cohort.

## Methods

### Systematic review

A systematic review was conducted to identify previously published PGS that predict individual risk of lung cancer. We searched Medline and EMBASE for peer-reviewed literature published in English between January 2012 and January 2024. This timeframe was selected as GWAS identifying lung cancer susceptibility loci became more prevalent in 2012, following the discovery of multiple susceptibility regions including 5p15, 6p21, and 15q25 [16]. The search strategy combined terms across three categories: “lung cancer,” “genetic risk,” and “cancer prediction/diagnosis.” Additionally, a grey literature search was performed in the Polygenic Score (PGS) Catalog, an open-access database where researchers upload PGS metadata [17]. The Catalog was searched using the terms “lung cancer,” “lung adenocarcinoma,” “squamous cell lung carcinoma,” “small cell lung carcinoma,” and “non-small cell lung carcinoma (NSCLC).” The complete search strategy is available in the supplementary materials (Table S1, S2). All identified articles were imported into EndNote for deduplication using the Bramer method [19].

Studies were included if they:

1. Were published in English, peer-reviewed, primary research papers.
2. Described a method using at least two SNPs to predict individual lung cancer risk.
3. Used a genome-wide approach (e.g. GWAS) to select SNPs associated with lung cancer.
4. Were developed for use in the general population rather than population subsets (e.g. pediatrics).

Where studies developed multiple PGS targeting specific populations (e.g., individuals who do vs. do not smoke), all relevant models were included. However, where multiple PGS were presented to compare development methodologies, only the best-performing model was included. Studies describing PGS for specific tumor subtypes (e.g., mesothelioma) were excluded.

Title and abstract screening were conducted using Rayyan [20, 21]. Pilot screening of 250 articles was performed by two reviewers (BG/HH), and discrepancies were resolved through discussion. After consistency in the application of the inclusion criteria was confirmed, the remaining articles were screened by one reviewer (BG), with 10% of the articles independently screened by a second reviewer (HH). If an article could not be excluded based on the title and abstract, the full text was retrieved. One reviewer screened all the full text articles (BG), and a 10% sample was checked by a second reviewer (HH). Reasons for exclusion at full-text screening were documented (Fig. S1).

A standardized data extraction form was developed. Information on the identification of SNPs associated with lung cancer, PGS development and evaluation cohorts, the number of SNPs included in each PGS, the PGS construction method, the outcome used, performance metrics, and the country (based on the affiliation of the first author), were recorded for all included studies. We used the classification system used in the PGS Catalog to group cohorts used in the development and validation of the PGS into the following three types:

1. G—Source of variant associations (e.g., previously published GWAS, meta-analyses, or newly conducted GWAS).
2. D—Score development/training datasets (e.g., optimization methods).
3. E—PGS evaluation cohorts (e.g., external validations).

For each cohort identified, cases, controls, and ethnicity were documented. A narrative synthesis approach was used to describe the characteristics of the identified lung cancer PGS and the cohorts used in their development and evaluation.

### Validation

The identified PGS were validated in the UKB cohort. UKB is a population-based cohort comprising approximately 500,000 participants aged 40–69 at baseline from England, Scotland, and Wales [22]. Participants were recruited between 2006 and 2011 with a 5.5% response rate [23]. Data on cancer incidence and mortality are available through linkage to national cancer registries. Analysis was restricted to participants with genetic data available (*n* = 488,377). Participants of all ethnicities were included in the analysis. Blood samples collected at baseline were genotyped using Affymetrix UK BiLEVE Axiom Array and Affymetrix UK Biobank Axiom array and imputed to the combined 1000 Genomes Project v.3 and UK10K reference panels using SHAPEIT3 and IMPUTE3 yielding data on approximately 96 million SNPs [24]. Participants with a previous cancer diagnosis (except non-melanoma skin cancer) were excluded from analysis [25]. Individuals included in the validation were followed from recruitment until the first cancer diagnosis, death, or the end of available follow-up (30/11/2022).

All identified PGS models, for which sufficient information was included in either the original publication or on the PGS Catalog to implement them, were calculated in the UKB cohort. We constructed all the PGS using the formula:

1 $${{{\mathrm{PGS}}}}={{\Sigma }_{i=1}^{n}\beta }_{i}x{G}_{i}$$

where n is the number of SNPs included in the PGS, ${\beta }_{i}$ is the effect size of *SNP*_*i*_, and ${G}_{i}$ is the genotype or imputed dosage of *SNP*_*i*_ for each individual in the cohort.

We used survival analysis to assess the association between PGS and lung cancer risk. Cox proportional hazards models were used to estimate hazard ratios (HR) per standard deviation (SD) increase in PGS. Discrimination was evaluated using Harrell’s concordance index (C-index). We also divided the cohort into percentiles to assess the accuracy of the PGS at identifying cases across risk categories. Subgroup analyses were conducted to assess PGS performance by smoking status, sex, and ethnicity. All analyses were performed using R version 4.2.2.

## Results

### Identified studies

The systematic review identified 46 studies and two additional entries from the PGS Catalog, collectively describing 60 PGS developed between 2016 and 2024 (Table 1, Fig. S1). Most studies were conducted in China (*n* = 21), the U.S. (*n* = 15), and the U.K. (*n* = 3). Included SNPs and their association with lung cancer risk were derived using GWAS (Table S3, S4), which were largely ethnically homogeneous, focusing primarily on European (*n* = 36) and East Asian (*n* = 6) populations. Development cohorts were not widely used (*n* = 8) (Table S5), but when they were, they were also ethnically homogenous. Whereas most studies directly applied SNPs and effect sizes from GWAS summary statistics, these studies used development data to refine PGS construction. Approaches included selecting optimal p-value thresholds for SNP inclusion, applying LD clumping to ensure independence of variants, and tuning shrinkage parameters in penalized regression methods such as LASSO or lassosum. Three studies used UKB in this step (although one of these models was excluded from the validation analysis as described in the next section) [26–28]. In contrast, fixed-weight scores constructed directly from GWAS summary statistics did not require parameter tuning.

**Table 1:** PGS included in Systematic Review.

First Author and Year	G: GWAS (Ethnicity)	D: Development (Ethnicity)	E: Validation	Tobacco Usage	Number of SNPs	Included in Validation	PGS Ref (where > 1 in validation)	Missing SNPs
Cheng (2016)	Chinese	Chinese	Same study	Adjusted	38	Y		0
Qian (2016)	European	European		Adjusted	301	Y		0
Dai (2019)	Chinese and European		Same study, elsewhere	Adjusted	19	Y		0
Shi (2019)	European		Same study, elsewhere	Not considered	6	Y		-
Fritsche (2020)	European	European	Same study, elsewhere	Not considered	19	N		-
Jia (2020)	European		Same study, elsewhere	Not considered	19	Y		0
Kachuri (2020)	European		Same study	Adjusted	109	Y	A (OR)	0
					109	N	B (unweighted)	-
					109	N	C (weighted by logOR divided by variance)	-
Yu (2020)	European		Same study	Stratified analysis	31	Y		1
Zhang (2020)	European		Same study	Not considered	15	N		-
Graff (2021)	European		Same study, elsewhere	Not considered	109	Y		0
Huang (2021)	European		Same study	Adjusted	18	Y		0
Hung (2021)	European	European	Same study, elsewhere	Stratified analysis	128	Y		4
Jia (2021)	European		Same study	Stratified analysis	19	Y		0
Wang (2021)	European		Same study	Adjusted	18	Y		0
Xie (2021)	European		Same study	Adjusted	18	Y		0
Barnett (2022)	European			Not considered	25	Y	A (OR)	3
					25	N	B (unweighted)	-
J. Choi (2022) - 1	European			Not considered	19	Y	A (LUAD)	0
					17	Y	B (LUSC)	0
J. Choi (2022) - 2	European			Not considered	19	Y	C	0
Liu (2022)	European		Same study	Not considered	15	Y	A (LUAD)	2
					6	Y	B (LUSC)	0
Qin (2022)	Not stated		Same study	Adjusted	20	Y		0
Tang (2022)	European	European		Adjusted	299	N		-
L. Wang (2022)	European		Same study	Not considered	73	Y		36
X. Wang (2022)	European		Same study	Stratified analysis	18	Y		1
Wei (2022)	European		Same study	Adjusted	18	N		-
P. Zhang (2022)	European		Same study, elsewhere	Stratified analysis	33	Y		0
R. Zhang (2022)	European		Same study	Not considered	128	Y	A (OR)	4
					128	N	B (weighted by OR and gene-gene interaction score)	-
Blechter (2023)	East Asian		Same study	Only individuals with no history of tobacco use	25	Y		0
Bryne (2023)	European		Same study	Adjusted	109	N		-
He (2023)	European			Not considered	18	N		-
Kim (2023)	European	European	Same study	Not considered	43	Y		0
Liang (2023)	European		Same study	Adjusted	18	Y		1
Namba (2023)	European		Same study	Not considered	Variable	N	A (LUCA)	-
					Variable	N	B (LUAD)	-
					Variable	N	C (LUSC)	-
Shi (2023)	East Asian			Stratified analysis, Smoking-specific PGS	28	Y	A (All)	0
					22	Y	B (Individuals with no history of tobacco use)	0
					22	Y	C (Individuals with history of tobacco use)	0
Trendowski (2023)	European		Same study	Adjusted	80	N	A (White)	-
					75	N	B (Black)	-
Wang (2023)	European		Same study	Stratified analysis	19	Y		0
Wei (2023)	East Asian		Same study	Only individuals with no history of tobacco use	16	Y	A (16 SNPs)	0
					21	Y	B (21 SNPs)	0
Xiao (2023)	European			Not considered	20	Y	A (LUCA)	1
					10	N	B (LUAD)	-
					15	N	C (LUSC)	-
Xin (2023)	European	European	Same study	Adjusted	16	Y		0
J. Zhang (2023)	European		Same study	Stratified analysis	18	Y		0
S. Zhang (2023)	European		Same study	Stratified analysis	18	N		-
Zhu (2023)	Chinese	Chinese	Same study	Stratified analysis	19	Y	A (Chinese)	2
					23	Y	B (European)	2
					25	Y	C (Trans-ancestry)	2
Duncan (2024)	European		Same study	Stratified analysis	120	N	A	-
					120	N	B	-
Felici (2024)	Not stated		Same study	Not considered	21	N		-

Summary of identified PGS.

The smoking categorization framework classifies studies based on how they account for smoking in PGS analysis. “Stratified analysis” applies when PGS performance is explicitly compared between individuals who do and do not use tobacco through separate models or subgroup analyses. “ Adjusted” is used when smoking is included as a covariate in statistical models but PGS performance is not stratified by smoking status. “Smoking-specific PGS” applies when separate PGS are developed for individuals who do and do not use tobacco, such as distinct models for individuals who have ever used tobacco and those who have never used tobacco. “Not considered” indicates that the study did not account for smoking in any way. “Only individuals with no history of tobacco use” apply to studies that exclusively analyzed individuals with no history of tobacco use.

*LUCA* lung cancer, *LUAD* lung adenocarcinoma, *LUSC* lung squamous cell carcinoma

Forty-four (44) of the identified PGS had been previously validated. All 44 PGS were validated in the same study that described their construction and 7 of them also underwent external validation in a separate study [29–35]. Among the studies that reported validation performance metrics, 18 reported AUC or C-index values, ranging from 0.53 to 0.67, indicating moderate discriminatory power. Based on previous validations, the PGS with the best discrimination is Zhang 2020 [14] (C-index= 0.67[se: 0.01]). PGS were generally found to be less predictive for lung cancer compared to PGS for other common cancers. 15 studies provided a comparison between the performance of lung cancer PGS and other cancer PRS. Of these studies, 11 reported that the lung cancer PRS performed poorly compared to other cancers [14, 27, 28, 30, 33, 36–41]. For example, Ho et al., [40] which evaluated PGS for 4 cancer types (breast, prostrate, colorectal, and lung cancer) in individuals of East Asian descent, found the lung cancer PGS to be the least predictive (AUC of 0.58 [95% CI: 0.55–0.61] and 0.55 [95% CI: 0.50**–**0.59] for men and women respectively).

Some efforts to tackle the lack of ethnic diversity in the development of lung cancer PGS have been made. Zhu et al. [42] developed three PGS (Chinese-specific, European-specific, and trans-ancestry) and found that the ethnicity-specific PGS performed better than the trans-ancestry PGS, and that the association between PGS and lung cancer risk was reduced when the ethnicity-specific PGS were cross-used (i.e. Chinese-specific score was evaluated in the European evaluation cohort and vice versa).

Following the systematic review, 39 (of the 60 identified in the review) were implemented in the UKB cohort. Fifteen were excluded due to insufficient information in the original study to implement them (including no information about the SNPs, weights or effect allele) (Table S8). A further five were excluded as they were duplicates (using both the same SNPs and the same weights) of other PGS (Table S11). When a study developed PGS for both overall lung cancer and specific subtypes, only the score for overall lung cancer was included. However, if a study developed PGS exclusively for subtypes, these were retained for the validation.

### Validation cohort

We identified 429,665 UKB participants with genetic data and no cancer diagnoses before baseline assessment (Fig. S2). The cohort (Table 2) was 52.8% women, had a mean [standard deviation] age at baseline assessment of 56.3 [8.1], comprised of 94% white individuals and includes a relatively high proportion of individuals with no history of tobacco use (54.8%). We identified 3541 individuals with a diagnosis of lung cancer during follow-up (0.82%). There was higher number of incident lung cancers among men (0.91%), white individuals (0.85%), individuals with smoking history (1.06%) and individuals who smoke (3.23%). Missing data were low for all variables used to define subgroup analyses (0%, 0.6% and 0.5% of the cohort had no data for sex, ethnicity and smoking status, respectively).

**Table 2:** Characteristics of the UKB Validation Cohort.

COHORT CHARACTERISTICS		ALL	CONTROLS	INCIDENT CASES	% INCIDENT WITH LUNG CANCER
COHORT SIZE	*n*	429,665	426,124	3541	0.82
AGE AT BASELINE (years)	Mean (SD^α^)	56.3 (8.1)	56.3 (8.1)	61.6 (5.9)	-
	Missing (%)	0 (0)	0 (0)	0 (0)	-
Sex	Women (%)	226,951 (52.8)	225,263 (52.9)	1688 (47.7)	0.74
	Men (%)	202,714 (47.2)	200,861 (47.1)	1853 (52.3)	0.91
	Missing (%)	0 (0)	0 (0)	0 (0)	-
Ethnicity	White (%)	402,318 (93.6)	398,904 (93.6)	3414 (96.4)	0.85
	Not White (%)	24,558 (5.7)	24,459 (5.7)	99 (2.8)	0.40
	Missing (%)	2789 (0.6)	2761 (0.6)	28 (0.8)	1.00
BMI (kg/m^2^)	Median (IQR^β^)	26.8 (24.2–29.9)	26.8 (24.2–29.9)	26.9 (24.1–30.1)	-
	Missing (%)	1766 (0.4)	1739 (0.4)	27 (0.8)	1.53
TOBACCO USAGE	Never (%)	235,297 (54.8)	234,825 (55.1)	472 (13.3)	0.20
	Former (%)	146,777 (34.2)	145,215 (34.1)	1562 (44.1)	1.06
	Current (%)	45,391 (10.6)	43,925 (10.3)	1466 (41.4)	3.23
	Missing (%)	2200 (0.5)	2159 (0.5)	41 (1.2)	1.86
Alcohol Consumption (units/DAY)	Median (IQR^β^)	1.6 (1.5–3.8)	1.6 (1.5–3.8)	1.8 (1.5–4.2)	-
	Missing (%)	1146 (0.3)	1132 (0.3)	14 (0.4)	1.22
Time to censoring (YEARS)	Median (IQR^β^)	13.6 (12.7–14.4)	13.6 (12.7–14.4)	6.8 (3.9–9.5)	-

^α^SD–standard deviation

^**β**^IQR–interquartile range

SNPs were identified in UKB based on their rsID and chromosomal position (Table S9). All SNPs from the original score were identified for 27 PGS (Table S10). For 11 PGS, a small number of SNPs ( < 5) were not identified within the UKB data. 36 SNPs were not identified for the Wang et al. 2022 score [41], >50% of the SNPs for this score. This PGS was excluded from the validation for this reason, leaving 38 PGS [26–30, 32–35, 37, 38, 42–60]. Several PGS included in the validation used the same set of included SNPs as another included PGS (Table S11), although in each case at least some of the associations used when computing the scores were different. Among the studies included in the validation, none of them used UKB as the GWAS source and only two, Kim (2023) [27] and Xin (2023) [28], used UKB in the development phase (Tables S3-S7).

These 38 PGS were implemented in the UKB cohort (Eq. 1). The distributions of each PGS, in cases and controls can be found in the supplementary materials (Fig. S3).

### Performance in validation

**Fig. 1:**
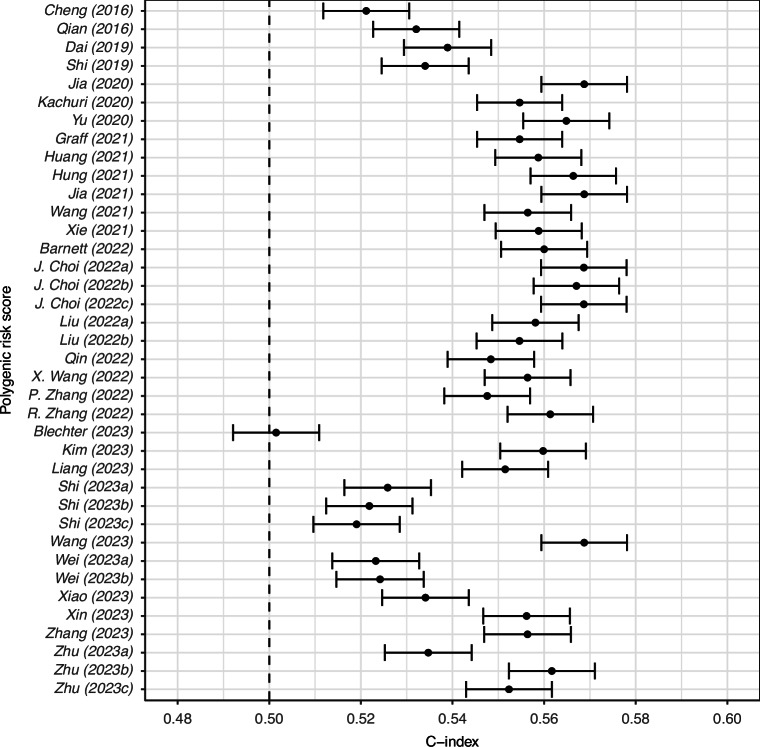
C-indices of PGS in the UKB validation cohort (ordered by year of publication and author name).

**Fig. 2:**
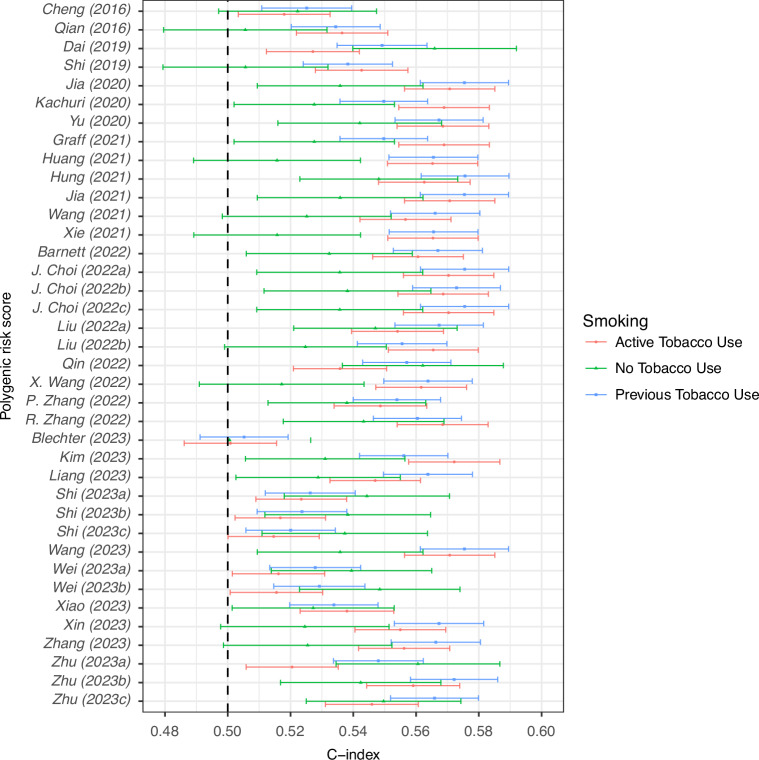
C-indices of PGS in the UKB validation cohort stratified by smoking status (ordered by year of publication and author name).

The discriminative ability of the PGS models was found to be relatively similar, with all 38 achieving C-indices in the range 0.49-0.58 when tested in the whole cohort (Fig. 1, Table S8). We draw attention to the five most discriminative PGS in this cohort (of which four use the same 19 SNPs and the fifth uses 17 of those 19, and which all drew their SNPs from the same study [61])—Jia2020 [32], Jia2021 [47], J. Choi2022a [51], J. Choi2022b [51] and Wang2023 [57]—which all have a C-index of 0.569 [95% CI: 0.559-0.578] for this population. These five models have an HR per SD of 1.28 [95% CI: 1.23–1.32] for association with lung cancer (Table S13). We see no improvement in discriminative performance of the PGS over time (Fig. 1) or as the number of SNPs is increased (Fig. S4). Differences in discriminative performance in the sub-cohorts with only men and only women are minimal (Fig. S5); however, we note that most (*n* = 30) perform slightly better in women than men. Similarly, there was little difference in discrimination when the models were tested in the sub-cohort of only white individuals (Fig. S6). In subgroup analysis by smoking status, however, the differences in discrimination were more substantial (Fig. 2). In most PGS (*n* = 27) the C-index was higher for individuals with prior and active tobacco use (compared to individuals with no history of tobacco use). However, a small number of PGS (*n* = 8) showed a trend in the other direction, in particular, we note, Dai2019 [29] (the C-index in individuals with no history of tobacco use is 0.566 [0.540–0.592] compared to 0.527 [0.512–0.542] in individuals with active tobacco use), Qin2022 [52] (0.562 [0.536–0.588] and 0.536 [0.521–0.551] and Zhu2023a [42] (0.561 [0.535–0.587] and 0.521 [0.506–0.535]).

When we look at the ability of the models to identify cases by risk percentile, we find that the best performing PGS varies depending on the threshold used (Table S14). For example, the PGS developed by R. Zhang et al. (2022) [54] identifies 1.7% of cases in the top 1% of the risk distribution and 7.8% in the top 5%. The PGS by Yu et al. (2020) [45], however, does better for slightly larger percentile groups, identifying 15.1% and 27.4% of cases in the highest 10 and 20 percentiles, respectively. The PGS by Jia et al. (2020) [32], Jia et al. (2021) [47] and Shi et al. (2023)[62] identify 60.2% of cases in the highest 50 percentiles and 94.5% of people in the highest 90 percentiles. As seen for discrimination, accuracy in identifying cases is notably different when stratified by tobacco usage (Table S18-20). While the best performing PGS are largely similar in the sub-cohorts of individuals with active and prior tobacco use, in individuals with no history of tobacco use, most PGS are less accurate but a small number perform better. Of note, the PGS developed by Dai et al. (2019) [29] correctly identifies 9.3% of cases in the highest 5 percentiles, 26.9% in the highest 20 percentiles and 61.2% in the highest 50 percentiles.

## Discussion

We systematically identified 60 PGS (across 46 studies) for the development of lung cancer which have been published since 2012. We were able to evaluate 39 of these PGS in a UKB cohort which included more than 3500 lung cancer cases. We found that the discrimination of all but one of these models was better than random allocation (C-indices > 0.5), and that almost all were associated with the development of lung cancer in this cohort. However, the ability of–even the best performing–PGS to identify high-risk cases is modest (i.e. less than 2% of cases are found in the highest percentile), meaning that at present lung cancer PGS have little scope for clinical implementation. No improvement to performance was seen over time (of publication) or for PGS with a higher number of included SNPs. The best performing PGS were different for individuals with and without history of tobacco use. Our results are in line with the performance seen in a recent validation of 9 lung cancer PGS using a cohort of high-risk individuals who smoke [18].

Compared to other cancers with similar incidence to lung cancer, the performance of these PGS is relatively poor. A PGS developed by Huyghe et al. [63]. for colorectal cancer had an AUC of 0.63 (95% CI, 0.61-0.64) in a validation using the UKB cohort [64], and the 313-SNP PGS for breast cancer used in the BOADICEA model had a C-index of 0.638 (95% CI: 0.632–0.645) in a UKB validation study of 10-year breast cancer risk [65]. The performance seen is, however, comparable to that seen in a recent validation of kidney cancer PGS (also in UKB), where the best performing model [66] had an AUC of 0.551 (95% CI: 0.528-0.573) [67]. While these PGS perform relatively poorly as standalone models, PGS used in clinical settings for other cancers typically do so as one part of a multifactorial risk assessment. Several studies have shown that lung cancer PGS may improve performance when combined with other risk models using demographic, lifestyle or clinical variables. Cheng et al. [43]. developed an epidemiological model, a PGS, and an extended model that incorporated both components. The extended model performed the best, with the highest AUC (0.70 compared to 0.61 and 0.65 for the epidemiologic and genetic models, respectively). In the validation of nine PGS in individuals who smoke conducted by Lebrett et al., [18] combining the PGS with clinical risk factors improved discrimination, although gains were modest (AUC increased by 0.002–0.015).

We see a large amount of duplicative efforts between the studies identified in this review of lung cancer PGS. Namely, many identify SNPs associated with lung cancer PGS from the same GWAS (for example, 34 of the studies use the McKay GWAS [61]) and there is overlap in the SNPs used between PGS. We excluded six PGS from the validation that used the same SNPs and weights as another PGS and found five sets of PGS that used the same sets of SNPs (with different associations).

As is seen throughout genetic research [68, 69], a lack of ethnic diversity remains a major limitation of this area. Of the 31 GWAS studies identified as being sources of SNPs in this review, only 11 used populations that included any individuals with non-European ancestry. This included 7 GWAS using East Asian ancestry populations and 3 GWAS using Chinese ancestry population. Only one, relatively small, GWAS used a cohort of African ancestry [70]. This GWAS was used as one of the sources of SNP associations used in one identified PGS [50]. The proportion of individuals included in our UKB cohort with non-white ancestry is relatively low (5.7% of controls and 2.8% of cases). Given the low numbers of lung cancer cases, the results for the sub-analysis of non-white individuals are largely inconclusive (Tables S8, S9). For most included PGS we are unable to conclude if an association with lung cancer risk is present. Removing non-white individuals from the analysis has no significant effect of the performance of any PGS (Fig. S6). The study by Zhu et al. (2023) [42] developed three PGS using three populations with different ancestries. Unsurprisingly, in this validation, the PGS developed using data from individuals with European ancestry has the highest discrimination (0.562 [95% CI: 0.552–0.571]).

Unlike previous validations [18], we use a general population cohort (including both individuals with and without a history of tobacco use). This allows for assessment of the PGS in subgroups by tobacco usage history as well as in the combined overall cohort. The identification of SNPs for lung cancer PGS has previously led to discussion that many associations identified were linked to addictive behaviour that may pre-dispose individuals to high rates of smoking, rather than to underlying pre-disposition to developing lung cancer [35, 71, 72]. In this study, we found that most of the PGS had worse discriminative ability in a sub-analysis using individuals with no history of tobacco use, compared to those with prior or active use. Interestingly, we found a small number of PGS that had improved performance in individuals with no history of tobacco use–including those developed by Dai et al. (2019) [29], Qin et al. (2022) [52] and Zhu et al. (2023) [42]. All three of these PGS included adjustment for smoking status in their development (Table 1); however, other PGS (such as Huang et al. (2021) [46]) with adjustment for smoking status perform poorly in individuals with no history of tobacco use. PGS that perform well in individuals with no history of tobacco use may include a higher proportion of SNPs associated with an underlying pre-disposition to lung cancer; they may be more suitable for identifying high-risk individuals in non-smoking populations or combining with smoking related risk factors in multifactorial models.

One limitation of our validation is that, where a direct match (by rsID or chromosomal position) to a SNP included in a score could not be found, we did not impute or substitute surrogate markers with high linkage disequilibrium. Additionally, this work does not attempt to address the complex gene-associations between lung cancer and smoking behaviour, nor other confounding outcomes (such as chronic lung diseases) that make interpretation of GWAS for lung cancer challenging. Finally, many of the GWAS from which the identified PGS were derived used case-control studies. Such designs are prone to survivor bias, since rapidly fatal cases are less likely to be included, and often recruit only older patients. As a result, the SNPs captured by these studies may not include more aggressive forms or early onset lung cancer [73].

This study was, in part, motivated by the challenge of selecting an appropriate lung cancer PGS to use when incorporating genetic information into multifactorial risk models, or modelling the use of a PGS as part of a more personalized approach to lung cancer screening. Our results show that, although many of the previously developed PGS have similar performance, selection of the most appropriate is context dependent. In a general population, we have identified five PGS (Jia2020 [32], Jia2021 [47], J. Choi2022a [51], J. Choi2022b [51] and Wang2023 [57]) with the highest discrimination. However, for researchers interested in the potential applications of genetic risk (for example as part of a multifactorial risk assessment for lung cancer screening), consideration of the accuracy of the PGS at different thresholds will be important; different PGS perform best at identifying cases in the highest risk percentiles, while others were more suited to identify the highest risk 20% or 50% of the population. If looking to model the potential of genetic risk in populations including high proportions of individuals with no history of tobacco use, or considering combining with smoking risk factors, the PGS developed by Dai et al. (2019) [29] may be the most appropriate choice at present. This study does not, however, consider whether introducing a genetic component to lung cancer screening has the potential to improve early detection rates or be cost-effective.

These PGS were developed to identify underlying genetic predisposition to lung cancer, and in this study were validated for the outcome of a primary lung cancer diagnosis. Therefore, they may be of interest in a screening context. Public health modelling approaches could be used to ascertain any of the expected impact (both short term—such as changes to number of people screened or diagnosed, and long term—such as impact on overall mortality) of adding a genetic component to a lung cancer screening program. Although performance of the PGS is modest, they may have potential to improve the effectiveness of screening selection when combined with other risk factors already used in this context. Given that these PGS do not perform as well as similar models for other cancers, there may also be research interest in improving understanding of genetic predisposition for lung cancer. This study has highlighted the need for research investigating the interaction of genetic and smoking risk factors, as well as the lack of ethnic diversity in lung cancer genetic research to date.

## Supplementary information

[updated] Supplementary Tables

Supplementary Figures

## Data Availability

Data may be obtained from a third party and are not publicly available. This research has been conducted using the UK Biobank Resource under Application Number 28126. This paper uses data from UK Biobank that the authors do not have permission to distribute. Bona-fide researchers can apply for access to this data from UK Biobank https://www.ukbiobank.ac.uk/. Data relating to the systematic review and validation (including template data extraction forms, lists of excluded studies and analysis code) can be requested from the corresponding author.
