# Supplementary material for: The current state of polygenic scores for the development of lung cancer: a systematic review and validation in UK Biobank
Source: Br J Cancer. 2026 Jan 8;134(6):939–48. doi: 10.1038/s41416-025-03330-9 (PMC12960659; doi:10.1038/s41416-025-03330-9)
Supplement: Supplementary file 1 — [updated] Supplementary Tables [file 41416_2025_3330_MOESM1_ESM.pdf]

## S1 MEDLINE Search

| | Search Line | Results |
|---|---|---|
| 1 | exp lung neoplasms/ or exp pleural neoplasms/ or exp tracheal neoplasms/ | 293,992 |
| 2 | ((lung or pleura* or trachea* or bronch*) adj3 (cancer* or neoplas* or tumo?r* or malignan* or carcinom*)).ti,ab. | 287,483 |
| 3 | (SCLC or NSCLC).ti,ab. | 69,750 |
| 4 | (("multi*" or "pan" or "cross" or "several" or "joint" or "combined") adj3 (cancer* or neoplas* or tumo?r* or malignan* or carcinom*)).ti,ab. | 193,485 |
| 5 | exp Genetic Predisposition to Disease/ | 158,597 |
| 6 | genetic predisposition to disease.ti,ab. | 90 |
| 7 | exp Multifactorial Inheritance/ | 4,189 |
| 8 | multifactorial inheritance.ti,ab. | 357 |
| 9 | (polygenic or polygenic score*).ti,ab. | 15,154 |
| 10 | polygenic risk score*.ti,ab. | 3,952 |
| 11 | genetic risk score*.ti,ab. | 2,615 |
| 12 | polygenic inheritance.ti,ab. | 554 |
| 13 | multigenic inheritance.ti,ab. | 42 |
| 14 | exp Prognosis/ | 1,952,500 |
| 15 | prognosis.ti,ab. | 530,944 |
| 16 | exp "Early Detection of Cancer"/ | 39,298 |
| 17 | early detection of cancer.ti,ab. | 1,593 |
| 18 | cancer prediction.ti,ab. | 665 |
| 19 | prediction model*.ti,ab. | 40,262 |
| 20 | prediction tool*.ti,ab. | 5,963 |
| 21 | mathematical model*.ti,ab. | 61,100 |
| 22 | (cancer adj5 (probability or chance or likelihood or risk)).ti,ab. | 166,791 |
| 23 | 1 or 2 or 3 or 4 | 565,863 |
| 24 | 5 or 6 or 7 or 8 or 9 or 10 or 11 or 12 or 13 | 173,011 |
| 25 | 14 or 15 or 16 or 17 or 18 or 19 or 20 or 21 or 22 | 2,508,805 |
| 26 | 23 and 24 and 25 | 3,900 |
| 27 | limit 26 to yr="2012 -Current" | 2,549 |

## S2 Embase Search

| | Search Line | Results |
|---|---|---|
| 1 | exp lung cancer/ or exp pleura malignancy/ or exp trachea cancer/ | 453,143 |
| 2 | ((lung or pleura* or trachea* or bronch*) adj3 (cancer* or neoplas* or tumo?r* or malignan* or carcinom*)).ti,ab. | 420,928 |
| 3 | (SCLC or NSCLC).ti,ab. | 127,123 |
| 4 | (("multi*" or "pan" or "cross" or "several" or "joint" or "combined") adj3 (cancer* or neoplas* or tumo?r* or malignan* or carcinom*)).ti,ab. | 276,207 |
| 5 | exp genetic predisposition/ | 211,863 |
| 6 | genetic predisposition to disease.ti,ab. | 121 |
| 7 | exp multifactorial inheritance/ | 271,149 |
| 8 | multifactorial inheritance.ti,ab. | 482 |
| 9 | genetic risk score/ | 10,236 |
| 10 | genetic risk score.ti,ab. | 3,141 |
| 11 | (polygenic or polygenic score*).ti,ab. | 21,793 |
| 12 | polygenic risk score*.ti,ab. | 6,852 |
| 13 | polygenic inheritance.ti,ab. | 692 |
| 14 | multigenic inheritance.ti,ab. | 58 |
| 15 | exp prognosis/ or exp cancer prognosis/ | 939,280 |
| 16 | prognosis.ti,ab. | 786,251 |
| 17 | cancer prognosis.ti,ab. | 6,811 |
| 18 | early cancer diagnosis/ | 14,621 |
| 19 | early cancer diagnosis.ti,ab. | 811 |
| 20 | cancer prediction.ti,ab. | 946 |
| 21 | prediction model*.ti,ab. | 52,665 |
| 22 | prediction tool*.ti,ab. | 8,672 |
| 23 | mathematical model*.ti,ab. | 72,365 |
| 24 | (cancer adj5 (probability or chance or likelihood or risk)).ti,ab. | 239,508 |
| 25 | 1 or 2 or 3 or 4 | 828,288 |
| 26 | 5 or 6 or 7 or 8 or 9 or 10 or 11 or 12 or 13 or 14 | 287,682 |
| 27 | 15 or 16 or 17 or 18 or 19 or 20 or 21 or 22 or 23 or 24 | 1,578,827 |
| 28 | 25 and 26 and 27 | 5,627 |
| 29 | limit 28 to yr="2012 -Current" | 4,386 |

**S3 (G) GWAS Studies Used**

| GWAS Reference | Study (first author, year) | Number of Studies Used By | Description | Ethnicity | Cases | Controls |
|---|---|---|---|---|---|---|
| G1 | McKay, 2017 (1) | 34 | Newly performed OncoArray studies and meta-analysis | European | **29,266** | 56,450 |
| G2 | Bossé, 2018 (36) | 7 | Review that synthesizes published GWAS | European | Varies by study | Varies by study |
| G3 | Wang, 2008 (39) | 6 | Newly performed GWAS, meta-analysis, and replication | European | GWAS: 1,952; Meta-analysis: 5,095; Replication: 2,484 | GWAS: 1,438; Meta-analysis: 5,200; Replication: 3,036 |
| G4 | Timofeeva, 2012 (40) | 5 | Meta-analysis | European | 14,900 | 29,485 |
| G5 | Wang, 2014 (41) | 5 | Meta-analysis of GWAS | European | 11,348 | 15,861 |
| G6 | Byun, 2018 (42) | 4 | Newly performed GWAS and evaluation | European | GWAS: 685<br>Evaluation: 1,993 | GWAS: 744<br>Evaluation: 33,690 |
| G7 | Landi, 2009 (43) | 4 | Newly performed GWAS and meta-analysis | European | 13,300 | 19,666 |
| G8 | Poirier, 2015 (44) | 4 | Two-stage GWAS | European | Stage 1: 3,953; Stage 2: 4,730 | Stage 1: 4,730; Stage 2: 7,476 |
| G9 | Brenner, 2015 (45) | 3 | Meta-analysis of GWAS | European | 14,049 | 33,456 |
| G10 | Kachuri, Linda, 2016 (46) | 3 | Newly performed GWAS | European | 5,164 | 5,716 |
| G11 | Weissfield, 2015 (47) | 3 | Newly performed GWAS | European | 778 | 1,166 |
| G12 | Broderick, 2009 (48) | 2 | Two-phase GWAS meta-analysis | European | Phase 1: 1,952; Phase 2: 2,465 | Phase 1: 1,438; Phase 2: 3,005 |
| G13 | Dai, 2019 (14) | 2 | Newly performed GWAS and meta-analysis | Chinese (48.94%) and European (51.06%) | 27,120 (Chinese: 13,327; European: 13,793) | 27,355 (Chinese: 13,328; European: 14,027) |
| G14 | Hu, 2011 (50) | 2 | Newly performed GWAS | Chinese | 2,331 | 3,077 |
| G15 | Hung, 2008 (52) | 2 | Newly performed GWAS with replication | European | GWAS: 1,989;<br>Replication: 2,513 | GWAS: 2,625;<br>Replication: 4,752 |
| G16 | Shiraishi, 2012 (53) | 2 | Newly performed GWAS | East Asian | GWAS: 6,029; Validation 1: 2,955; Validation 2: 1,379 | GWAS: 13,535; Validation 1: 7,036; Validation 2: 1,166 |
| G17 | Amos, 2008 (54) | 1 | Multi-stage GWAS | European | 1,154 (Replication 1: 711; Replication 2: 2,013) | 1,137 (Replication 1: 632; Replication 2: 3,062) |
| G18 | Dong, 2012 (55) | 1 | Two-stage replication | Chinese | 7,436<br>(Stage 1: 2,283; Stage 2: 5,153) | 7,483 (Stage 1: 2,243; Stage 2: 5,240) |
| G19 | Fehringer, 2016 (56) | 1 | Multi-stage GWAS meta-analysis | European | 12,160<br>Replication: 3,865<br>Generalizability: 2,331 | 16,838<br>Replication: 196,658<br>Generalizability: 3,077 |
| G20 | Hsiung, 2010 (57) | 1 | Newly performed GWAS with replication | East Asian | 584<br>Replication: 1,164 | 585<br>Replication: 1,736 |
| G21 | Lan, 2012 (58) | 1 | Newly performed multi-stage GWAS | East Asian | Stage 1: 5,510;<br>Stage 2: 1,099 | Stage 1: 4,544; Stage 2: 2,913 |
| G22 | Landi, 2009 (43) | 1 | Newly performed GWAS followed by meta-analysis | European | GWAS: 5,739;<br>Meta-analysis: 13,300 | GWAS: 5,848; Meta-analysis: 19,666 |
| G23 | Liu, 2008 (59) | 1 | Newly performed GWAS | European | 194 | 219 |
| G24 | McKay, 2008 (60) | 1 | Newly performed GWAS | European | 3,259 (Replication: 2,899) | 4,159 (Replication: 5,573) |
| G25 | Miki, 2010 (61) | 1 | Newly performed GWAS with replication | East Asian | 1,004 (Replication: 1,094) | 1,900 (Replication: 9,148) |
| G26 | Seow, 2017 (62) | 1 | Newly performed GWAS meta-analysis | East Asian | 11,725 | 14,490 |
| G27 | Shi, 2023 (49) | 1 | Two-stage GWAS meta-analysis | East Asian | Discovery: 11,753; Replication: 9,905) | Discovery: 30,562; Replication: 120,114) |
| G28 | Thorgeirsson, 2008 (64) | 1 | Newly performed GWAS | European | 1,024 | 32,244 |
| G29 | Wang, 2016 (65) | 1 | Newly performed GWAS meta-analysis | East Asian | Discovery: 6,877; Replication: 5,878 | Discovery: 6,277; Replication: 7,046 |
| G30 | Wang, 2016 (66) | 1 | Pooled analysis of six GWAS | European | 12,160 | 16,838 |
| G31 | Zanetti, 2016 (67) | 1 | Two-stage case-control study | African American | Stage 1: 1,737<br>Stage 2: 866 | Stage 1: 3,602<br>Stage 2: 796 |

## S4 (G) Cohorts to Perform GWAS

| GWAS Cohort Reference | Cohort | Number of Studies Used By | Description | Disease | Ethnicity | Cases | Controls |
|---|---|---|---|---|---|---|---|
| GC1 | FLCCA | 2 | Newly performed GWAS | Lung cancer | East Asian | 4,438-5,510 | 4,544 |
| GC2 | NJMU GSA | 2 | Newly performed GWAS | NSCLC | Chinese | 10,248 | 9,298 |
| GC3 | NJMU GWAS | 2 | Three-stage case-control analysis | Lung cancer | Chinese | Scan: 2,331; Validation 1: 2,283; Validation 2: 4,030 | Scan: 3,077; Validation 1: 2,243; Validation 2: 4,166 |
| GC4 | ACC | 1 | Contributed to two-stage GWAS meta-analysis | Lung cancer | East Asian | 1,471 | 2,564 |
| GC5 | NCC | 1 | Contributed to two-stage GWAS meta-analysis | Lung cancer | East Asian | 3,921 | 19,910 |
| GC6 | NJLCS | 1 | Meta-analysis | Lung cancer | East Asian | 1,923 | 3,544 |
| GC7 | NJMU OncoArray | 1 | Unpublished data | NSCLC | Chinese | 953 | 953 |
| GC8 | TRICL | 1 | GWAS meta-analysis | Lung cancer | European | 7,766 | 37,482 |
| GC9 | UKB | 1 | Population-based cohort study | Lung cancer | European | Not stated | Not stated |

**S4 (G) Cohorts to Perform GWAS**

| GWAS Cohort Reference | Cohort | Number of Studies Used By | Description | Disease | Ethnicity | Cases | Controls |
|---|---|---|---|---|---|---|---|

*Abbreviations: FLCCA = Female Lung Cancer Consortium in Asia, NJMU = Nanjing Medical University, ACC = Aichi Cancer Center Japan, NCC = National Cancer Center Research Institute Japan, NJLCS = Nanjing Lung Cancer Study, TRICL = Transdisciplinary Research in Cancer of the Lung, UKB = UK Biobank*

## S5 (D) Development Cohorts Used

| Development Cohort Reference | Study | Number of Studies Used By | Description | Ethnicity | Cases | Controls |
|---|---|---|---|---|---|---|
| D1 | UK Biobank | 3 | Population-based cohort study | European | 1,048-2,232 (overall cohort values, but cohorts were split into development and training set) | Fritsche: 22,319 (overall cohort value, but cohort was split into development and training set)<br>Kim: Not stated<br>Xin: Not stated |
| D2 | INTEGRAL-ILCCO OncoArray Project | 2 | Consortium of lung cancer studies | European | 13,119-13,793 | 10,008-14,027 |
| D3 | Dai, 2019 | 1 | GWAS | Chinese | 13,327 | 13,328 |
| D4 | EAGLE | 1 | Population-based case-control study | European | 1,815 | 1,971 |
| D5 | McKay, 2017 | 1 | GWAS | European | 29,266 | 56,450 |
| D6 | MGI | 1 | Patient database | European | 874 (overall cohort value, but cohort was split into development and training set) | 8,724 (overall cohort value, but cohort was split into development and training set) |
| D7 | NJMU GWAS | 1 | GWAS | Chinese | 2,331 | 3,077 |
| D8 | PLCO | 1 | Randomized controlled trial | European | 1,453 (overall cohort, value, but 5-fold cross-validation used for development and evaluation) | 12,962 (overall cohort value, but 5-fold cross-validation used for development and evaluation) |

*Abbreviations: UKB = UK Biobank, ILCCO = International Lung Cancer Consortium, EAGLE = Environment and Genetics in Lung Cancer Etiology, MGI = Michigan Genomics Initiative, NJMU = Nanjing Medical University, PLCO = Prostate, Lung, Colorectal, and Ovarian Cancer Screening Trial*

**S6 (E) Evaluation Cohorts Used**

| Evaluation Cohort Reference | Evaluation Cohort | Number of Studies Used By | Description | Ethnicity | Cases | Controls |
|---|---|---|---|---|---|---|
| E1 | UKB | 21 | Population-based cohort study | European | 1,048-3,017 | 22,319-477,453 |
| E2 | CKB | 3 | Population-based cohort study | Chinese | 466-1,392 | 54,800-99,223 |
| E3 | TCGA | 3 | Genomics program | European | 334-908 | 422-6,178 |
| E4 | EAGLE | 1 | Population-based case-control study | European | 1,937 | 1,984 |
| E5 | eMERGE | 1 | Consortium of electronic medical records | European | Cases extracted from different study | 13,427 |
| E6 | ESTHER | 1 | Cohort study | European | 143 | 1,460 |
| E7 | FHS | 1 | Long-term, multigenerational study | European | 86 | 2,838 |
| E8 | GELAC | 1 | Case-control study | East Asian | 1,024 | 1,024 |
| E9 | GERA | 1 | Cohort study | African, East Asian, European, and Latino | 2,488 (total across two evaluation cohorts) | 410,354 (total across two evaluation cohorts) |
| E10 | INHALE | 1 | Case-control study | European and African American | non-Hispanic whites: 1,103; blacks: 558 | non-Hispanic whites: 812; blacks: 565 |
| E11 | MCRC | 1 | Biobank | European | Cases extracted from different study | 706 |
| E12 | Manchester LHC | 1 | Pilot lung cancer screening | European | 701 | Controls extracted from different study |
| E13 | MGI | 1 | Longitudinal biorepository | European | 874 (overall cohort value, but cohort was Esplit into development and training set) | 8,724 (overall cohort value, but cohort was split into development and training set) |
| E14 | NLCC | 1 | Case-control study | East Asian | 10,248 | 9,298 |
| E15 | Penn Medicine Biobank | 1 | Biobank | European and African American | European: 285<br>African American: 187 | European: 6,383<br>African American: 5,701 |
| E16 | SCHS | 1 | Cohort study | East Asian | 528 | 21,166 |

*Abbreviations: UKB = UK Biobank, CKB = China Kadoorie Biobank, TCGA = The Cancer Genome Atlas, EAGLE = Environment and Genetics in Lung Cancer Etiology, eMERGE = Electronic Medical Records and Genomics, FHS = Framingham Heart Study, GELAC = Genetic Epidemiological Study of Lung* Adenocarcinoma, GERA = Genetic *Epidemiology Research on Adult Health and Aging, MCRC = Manchester Cancer Research Center Biobank, Manchester LHC = Manchester Lung Health Check, MGI = Michigan Genomics Initiative, NLCC = Nanjing Lung Cancer Cohort, PLCO = Prostate, Lung, Colorectal, and Ovarian Cancer , SCHS = Singapore Chinese Health Study*

## S7 References for GDE type

| Ref | First Author | Year | Associated Studies | | | |
|---|---|---|---|---|---|---|
| | | | G | D | E | |
| *Cheng (2016)* | Cheng | 2016 | GC3 | D7 | E4 | |
| *Qian (2016)* | Qian | 2016 | GC8 | D4 | - | |
| *Dai (2019)* | Dia | 2019 | G1, G14, GC2 | - | E2 | |
| *Shi (2019)* | Shi | 2029 | G1, G3, G5, G19, G22 | - | E3, E5 | |
| *Fritsche (2020)* | Fritsche | 2020 | G1, G5, G6, G7, GC9 | D1, D6 | E1, E13 | |
| *Jia (2020)* | Jia | 2020 | G1, G2 | - | E1, E13 | |
| *Kachuri (2020)* | Kachuri | 2020 | G1, G3, G4,G5, G6 | - | E1 | |
| *Yu (2020)* | Yu | 2020 | G1, G3, G4, G5, G7, G12, G15, G17, G23, G24, G28 | - | E6 | |
| *Zhang (2020)* | Zhang | 2020 | G1 | - | - | |
| *Graff (2021)* | Graff | 2021 | G1, G3, G4, G6 | - | E1, E9 | |
| *Huang (2021)* | Huang | 2021 | G1 | - | - | |
| *Hung (2021)* | Hung | 2021 | G1, G2, G8, G9, G10, G11 | D2 | E1 | |
| *Jia (2021)* | Jia | 2021 | G1, G2 | - | E1 | |
| *Wang (2021)* | Wang | 2021 | G1 | - | - | |
| *Xie (2021)* | Xie | 2021 | G1 | - | E1 | |
| *Barnett (2022)* | Barnett | 2022 | G1, G3, G4, G7, G15, G31 | - | - | |
| *J. Choi (2022) - 1* | J. Choi | 2022 | G2 | - | - | |
| *J. Choi (2022) - 2* | J. Choi | 2022 | G2 | - | - | NB: HGG Advances |
| *Liu (2022)* | Liu | 2022 | G1, G7 | - | E3 | |
| *Qin (2022)* | Qin | 2022 | G1 | - | E1, E14 | |
| *Tang (2022)* | Tang | 2022 | - | D8 | - | |
| *L. Wang (2022)* | Wang | 2022 | G1 | - | E15 | |
| *X. Wang (2022)* | Wang | 2022 | G1 | - | E1 | |
| *Wei (2022)* | Wei | 2022 | - | - | - | |
| *P. Zhang (2022)* | P. Zhang | 2022 | G5, G8, G12, G13, G16, G18, G30 | - | E1 | |
| *R. Zhang (2022)* | R. Zhang | 2022 | G1, G2, G8, G9, G10, G11 | - | E1 | |
| *Blechter (2023)* | Blechter | 2023 | G27 | - | E8 | |
| *Bryne (2023)* | Bryne | 2023 | G1, G3, G4, G6 | - | E1 | |
| *He (2023)* | He | 2023 | G1 | - | - | |
| *Ho (2023)* | Ho | 2023 | - | - | E16 | |
| *Huntley (2023)* | Huntley | 2023 | - | - | - | |
| *Kim (2023)* | Kim | 2023 | G1 | D1 | E1 | |
| *Lebrett (2023)* | Lebrett | 2023 | - | - | E11, E12 | |
| *Liang (2023)* | Liang | 2023 | G1 | - | E1 | |
| *Namba (2023)* | Namba | 2023 | G1 | - | E1 | |
| *Shi (2023)* | Shi | 2023 | G1, G13, G14, G16, G20, G21, G25, G26, G29, GC1, GC4, GC5, GC6 | - | - | |
| *Trendowski (2023)* | Trendowski | 2023 | G1, G2, G8, G9, G10, G11 | - | E10 | |
| *Wang (2023)* | Wang | 2023 | G1 | - | E1 | |
| *Wei (2023)* | Wei | 2023 | GC1, GC2, GC3, GC7 | - | E1, E2 | |

**S7 References for GDE type**

| Ref | First Author | Year | Associated Studies | | |
|---|---|---|---|---|---|
| *Xiao (2023)* | Xiao | 2023 | G1 | - | - |
| *Xin (2023)* | Xin | 2023 | G1 | D1 | E3 |
| *J. Zhang (2023)* | J. Zhang | 2023 | G1 | - | E1 |
| *S. Zhang (2023)* | S. Zhang | 2023 | G1 | - | E1 |
| *Zhu (2023)* | Zhu | 2023 | Used GWAS, but specific studies not provided | D2, D3, D5 | E1, E2 |
| *Duncan (2024)* | Duncan | 2024 | G1 | - | E7 |
| *Felici (2024)* | Felici | 2024 | - | - | E1 |

**S8 PGS excluded from validation**

| Study | Year | First Author | Insufficient Information in orginal paper | Duplicate (both SNps and weights) of another PGS | Other | Details |
|---|---|---|---|---|---|---|
| | | | *14* | *5* | *3* | |
| Study 36 | 2024 | Felici, Alessio | x | | | No details of SNPs or weighting |
| Study 5, PGS A | 2024 | Duncan, Meredith | x | | | No details of SNPs or weighting |
| Study 5, PGS B | 2024 | Duncan, Meredith | x | | | No details of SNPs or weighting |
| Study 1 | 2023 | Byrne, Stephanie | x | | | No details of weightings, possible duplication of Graff |
| Study 15, PGS B | 2023 | Xiao, Zhihan | | | x | Outcome is LUAD (PGS A is all lung cancers) |
| Study 15, PGS C | 2023 | Xiao, Zhihan | | | x | Outcome is LUSC (PGS A is all lung cancers) |
| Study 6, PGS 1 | 2023 | Namba, Shinichi | x | | | No rsIDs |
| Study 6, PGS 2 | 2023 | Namba, Shinichi | x | | | No rsIDs, wrong outcome? |
| Study 6, PGS 3 | 2023 | Namba, Shinichi | x | | | No rsIDs, worng outcome? |
| Study 9 | 2023 | Zhang, Shanshan | x | | | No details of weighting |
| Study 46 | 2022 | Tang, Yingdan | x | | | No details of SNPs or weighting |
| Study 29 | 2020 | Zhang, Yan | x | | | No details of SNPs or weighting |
| Study 44, Evaluation 1 | 2020 | Fritsche, Lars | x | | | No rsIDs |
| | | | | | | |
| Study 24, PGS A | 2023 | Trendowski, Matthew | x | | | No effect allele |
| Study 24, PGS B | 2023 | Trendowski, Matthew | x | | | No effect allele |
| Study 13, Validation 1 | 2022 | Wang, Louise | | | x | >50% SNPs not found in UK Biobank |
| Study 12, PGS B | 2022 | Barnett, Gillian | x | | | No weights given |
| Study 39 | 2022 | Wei, Xiaoxia | | x | | duplicate |
| Study 21, PRS B | 2022 | Zhang, Rhuyang | | x | | Duplicate with alternative construction |
| Study 11 | 2022 | He, Heng | | x | | duplicate |
| Study 30, PGS B | 2020 | Kachuri, Linda | | x | | Duplicate with alternative construction |
| Study 30, PGS C | 2020 | Kachuri, Linda | | x | | Duplicate with alternative construction |

## S9 List of Included SNPs

| PGS_name | Label | rsID | Effect allele | Position | rsID in UKB extract | position in UKB extract | found in UKB extract | rsID in UKB | Position in UKB |
|---|---|---|---|---|---|---|---|---|---|
| PGP000049_Dai_2019 | Dai (2019) | rs17038564 | G | 2:65496058 | 1 | 1 | 1 | rs17038564 | 2:65496058_A_G |
| PGP000049_Dai_2019 | Dai (2019) | rs2293607 | T | 3:169482335 | 1 | 1 | 1 | rs2293607 | 3:169482335_T_C |
| PGP000049_Dai_2019 | Dai (2019) | rs11375254 | T | 3:189343242 | 1 | 1 | 1 | rs11375254 | 3:189343242_T_TA |
| PGP000049_Dai_2019 | Dai (2019) | rs13167280 | A | 5:1280477 | 1 | 1 | 1 | rs13167280 | 5:1280477_G_A |
| PGP000049_Dai_2019 | Dai (2019) | rs401681 | C | 5:1322087 | 1 | 1 | 1 | rs401681 | rs401681 |
| PGP000049_Dai_2019 | Dai (2019) | rs2517873 | A | 6:29875992 | 1 | 1 | 1 | rs2517873 | 6:29875992_G_A |
| PGP000049_Dai_2019 | Dai (2019) | rs3817963 | C | 6:32368087 | 1 | 1 | 1 | rs3817963 | rs3817963 |
| PGP000049_Dai_2019 | Dai (2019) | rs1853837 | A | 6:41497035 | 1 | 1 | 1 | rs1853837 | 6:41497035_C_A |
| PGP000049_Dai_2019 | Dai (2019) | rs5879422 | T | 6:117784658 | 1 | 1 | 1 | rs5879422 | 6:117784658_T_TTG |
| PGP000049_Dai_2019 | Dai (2019) | rs4236709 | G | 8:32410110 | 1 | 1 | 1 | rs4236709 | 8:32410110_G_A |
| PGP000049_Dai_2019 | Dai (2019) | rs10429489 | A | 9:21787521 | 1 | 1 | 1 | rs10429489 | 9:21787521_G_A |
| PGP000049_Dai_2019 | Dai (2019) | rs35201538 | C | 9:33422488 | 1 | 1 | 1 | rs35201538 | 9:33422488_C_CT |
| PGP000049_Dai_2019 | Dai (2019) | rs4573350 | T | 9:124955115 | 1 | 1 | 1 | rs4573350 | 9:124955115_T_C |
| PGP000049_Dai_2019 | Dai (2019) | rs12265047 | G | 10:114487925 | 1 | 1 | 1 | rs12265047 | 10:114487925_G_A |
| PGP000049_Dai_2019 | Dai (2019) | rs55768116 | C | 11:118108331 | 1 | 1 | 1 | rs55768116 | 11:118108331_C_A |
| PGP000049_Dai_2019 | Dai (2019) | rs11610143 | C | 12:52349071 | 1 | 1 | 1 | rs11610143 | 12:52349071_C_G |
| PGP000049_Dai_2019 | Dai (2019) | rs1200399 | C | 14:35293185 | 1 | 1 | 1 | rs1200399 | rs1200399 |
| PGP000049_Dai_2019 | Dai (2019) | rs77468143 | T | 15:49376624 | 1 | 1 | 1 | rs77468143 | 15:49376624_T_G |
| PGP000049_Dai_2019 | Dai (2019) | rs200595745 | A | 17:65915289 | 0 | 1 | 1 | rs777750649 | 17:65915289_AAATAATAAT_A |
| PGP000050_Graff_2021 | Graff (2021) | rs71658797 | A | 1:77967507 | 1 | 1 | 1 | rs71658797 | 1:77967507_T_A |
| PGP000050_Graff_2021 | Graff (2021) | rs13080835 | G | 3:189357199 | 1 | 1 | 1 | rs13080835 | 3:189357199_G_T |
| PGP000050_Graff_2021 | Graff (2021) | rs71603396 | A | 4:18012035 | 1 | 1 | 1 | rs71603396 | 4:18012035_G_A |
| PGP000050_Graff_2021 | Graff (2021) | rs13156167 | C | 5:1275857 | 1 | 1 | 1 | rs13156167 | 5:1275857_T_C |
| PGP000050_Graff_2021 | Graff (2021) | rs56345976 | G | 5:1276873 | 1 | 1 | 1 | rs56345976 | 5:1276873_G_A |
| PGP000050_Graff_2021 | Graff (2021) | rs33961405 | G | 5:1277577 | 1 | 1 | 1 | rs33961405 | 5:1277577_G_A |
| PGP000050_Graff_2021 | Graff (2021) | rs7705526 | A | 5:1285974 | 1 | 1 | 1 | rs7705526 | 5:1285974_C_A |
| PGP000050_Graff_2021 | Graff (2021) | rs2853677 | G | 5:1287194 | 1 | 1 | 1 | rs2853677 | rs2853677 |
| PGP000050_Graff_2021 | Graff (2021) | rs148297846 | A | 5:1298017 | 1 | 1 | 1 | rs148297846 | 5:1298017_G_A |
| PGP000050_Graff_2021 | Graff (2021) | rs62329683 | T | 5:1299087 | 1 | 1 | 1 | rs62329683 | 5:1299087_C_T |
| PGP000050_Graff_2021 | Graff (2021) | rs112401627 | A | 5:1300269 | 1 | 1 | 1 | rs112401627 | 5:1300269_G_A |
| PGP000050_Graff_2021 | Graff (2021) | rs4635969 | G | 5:1308552 | 1 | 1 | 1 | rs4635969 | rs4635969 |
| PGP000050_Graff_2021 | Graff (2021) | rs380286 | G | 5:1320247 | 1 | 1 | 1 | rs380286 | 5:1320247_G_A |
| PGP000050_Graff_2021 | Graff (2021) | rs410805 | T | 5:1323196 | 1 | 1 | 1 | rs410805 | 5:1323196_T_C |
| PGP000050_Graff_2021 | Graff (2021) | rs55901723 | T | 5:1342154 | 0 | 1 | 1 | rs391314 | 5:1342154_T_C |
| PGP000050_Graff_2021 | Graff (2021) | rs40183 | G | 5:1348352 | 1 | 1 | 1 | rs40183 | 5:1348352_G_A |
| PGP000050_Graff_2021 | Graff (2021) | rs6889886 | C | 5:1358822 | 1 | 1 | 1 | rs6889886 | 5:1358822_C_T |
| PGP000050_Graff_2021 | Graff (2021) | rs806794 | A | 6:26200677 | 1 | 1 | 1 | rs806794 | rs806794 |
| PGP000050_Graff_2021 | Graff (2021) | rs3999544 | C | 6:26285867 | 1 | 1 | 1 | rs3999544 | 6:26285867_C_T |
| PGP000050_Graff_2021 | Graff (2021) | rs9467711 | C | 6:26332605 | 1 | 1 | 1 | rs9467711 | rs9467711 |
| PGP000050_Graff_2021 | Graff (2021) | rs6912292 | A | 6:26511805 | 1 | 1 | 1 | rs6912292 | 6:26511805_C_A |
| PGP000050_Graff_2021 | Graff (2021) | rs116461399 | C | 6:28796071 | 0 | 1 | 1 | rs3131337 | 6:28796071_G_C |
| PGP000050_Graff_2021 | Graff (2021) | rs116045192 | T | 6:29272012 | 0 | 1 | 1 | rs3117426 | rs3117426 |
| PGP000050_Graff_2021 | Graff (2021) | rs114924489 | G | 6:29468809 | 0 | 1 | 1 | rs1233487 | rs1233487 |
| PGP000050_Graff_2021 | Graff (2021) | rs114050064 | C | 6:29698821 | 0 | 1 | 1 | rs1611350 | rs1611350 |
| PGP000050_Graff_2021 | Graff (2021) | rs115813114 | C | 6:29766085 | 0 | 1 | 1 | rs1632999 | 6:29766085_T_C |
| PGP000050_Graff_2021 | Graff (2021) | rs111597677 | C | 6:29829251 | 0 | 1 | 1 | rs1611713 | 6:29829251_A_C |
| PGP000050_Graff_2021 | Graff (2021) | rs115960997 | A | 6:29902109 | 0 | 1 | 1 | rs1611314 | 6:29902109_G_A |
| PGP000050_Graff_2021 | Graff (2021) | rs116405142 | G | 6:30066031 | 0 | 1 | 1 | rs916570 | rs916570 |
| PGP000050_Graff_2021 | Graff (2021) | rs115993819 | A | 6:30074163 | 0 | 1 | 1 | rs2517600 | rs2517600 |
| PGP000050_Graff_2021 | Graff (2021) | rs142696802 | T | 6:30190040 | 0 | 1 | 1 | rs2517602 | 6:30190040_C_T |
| PGP000050_Graff_2021 | Graff (2021) | rs116074010 | T | 6:30391975 | 0 | 1 | 1 | rs3130127 | 6:30391975_A_T |
| PGP000050_Graff_2021 | Graff (2021) | rs115915654 | T | 6:30769726 | 0 | 1 | 1 | rs1264372 | rs1264372 |
| PGP000050_Graff_2021 | Graff (2021) | rs116552815 | A | 6:30855907 | 0 | 1 | 1 | rs1264323 | rs1264323 |
| PGP000050_Graff_2021 | Graff (2021) | rs114103504 | A | 6:31002452 | 0 | 1 | 1 | rs3869098 | 6:31002452_A_G |
| PGP000050_Graff_2021 | Graff (2021) | rs115669859 | A | 6:31010200 | 0 | 1 | 1 | rs2508015 | rs2508015 |
| PGP000050_Graff_2021 | Graff (2021) | rs182364552 | A | 6:31069841 | 0 | 1 | 1 | rs9263573 | 6:31069841_T_A |
| PGP000050_Graff_2021 | Graff (2021) | rs3130552 | A | 6:31082127 | 1 | 1 | 1 | rs3130552 | rs3130552 |
| PGP000050_Graff_2021 | Graff (2021) | rs116651383 | C | 6:31138910 | 0 | 1 | 1 | rs3094191 | 6:31138910_T_C |
| PGP000050_Graff_2021 | Graff (2021) | rs115681412 | G | 6:31140741 | 0 | 1 | 1 | rs1265158 | rs1265158 |
| PGP000050_Graff_2021 | Graff (2021) | rs141707415 | A | 6:31239681 | 0 | 1 | 1 | rs9264669 | 6:31239681_A_T |
| PGP000050_Graff_2021 | Graff (2021) | rs111324505 | T | 6:31242082 | 0 | 1 | 1 | rs7764413 | 6:31242082_T_C |
| PGP000050_Graff_2021 | Graff (2021) | rs115494074 | T | 6:31313030 | 0 | 1 | 1 | rs2394982 | 6:31313030_C_T |
| PGP000050_Graff_2021 | Graff (2021) | rs139850307 | C | 6:31324586 | 0 | 1 | 1 | rs1050556 | 6:31324586_C_T |
| PGP000050_Graff_2021 | Graff (2021) | rs2249935 | A | 6:31327178 | 1 | 1 | 1 | rs2249935 | 6:31327178_G_A |
| PGP000050_Graff_2021 | Graff (2021) | rs115471405 | C | 6:31335647 | 0 | 1 | 1 | rs2844571 | 6:31335647_T_C |

## S9 List of Included SNPs

| PGS_name | Label | rsID | Effect allele | Position | rsID in UKB extract | position in UKB extract | found in UKB extract | rsID in UKB | Position in UKB |
|---|---|---|---|---|---|---|---|---|---|
| PGP000050_Graff_2021 | Graff (2021) | rs115595430 | T | 6:31346445 | 0 | 1 | 1 | rs2523627 | rs2523627 |
| PGP000050_Graff_2021 | Graff (2021) | rs114965247 | G | 6:31350303 | 0 | 1 | 1 | rs2844535 | 6:31350303_T_G |
| PGP000050_Graff_2021 | Graff (2021) | rs139089278 | T | 6:31373174 | 0 | 1 | 1 | rs2442715 | 6:31373174_C_T |
| PGP000050_Graff_2021 | Graff (2021) | rs114279165 | G | 6:31425499 | 0 | 1 | 1 | rs3131621 | rs3131621 |
| PGP000050_Graff_2021 | Graff (2021) | rs116822326 | G | 6:31434111 | 0 | 1 | 1 | rs3094604 | 6:31434111_A_G |
| PGP000050_Graff_2021 | Graff (2021) | rs115441327 | C | 6:31613739 | 0 | 1 | 1 | rs3130048 | rs3130048 |
| PGP000050_Graff_2021 | Graff (2021) | rs2763981 | T | 6:31840021 | 1 | 1 | 1 | rs2763981 | 6:31840021_T_A |
| PGP000050_Graff_2021 | Graff (2021) | rs652888 | G | 6:31851234 | 1 | 1 | 1 | rs652888 | rs652888 |
| PGP000050_Graff_2021 | Graff (2021) | rs114676850 | T | 6:32208324 | 0 | 1 | 1 | rs424232 | rs424232 |
| PGP000050_Graff_2021 | Graff (2021) | rs9270868 | G | 6:32571117 | 1 | 1 | 1 | rs9270868 | 6:32571117_A_G |
| PGP000050_Graff_2021 | Graff (2021) | rs116080888 | C | 6:32586236 | 0 | 1 | 1 | rs11751024 | 6:32586236_C_A |
| PGP000050_Graff_2021 | Graff (2021) | rs113574092 | G | 6:32586787 | 0 | 1 | 1 | rs9271364 | 6:32586787_A_G |
| PGP000050_Graff_2021 | Graff (2021) | rs35867827 | T | 6:32592068 | 1 | 1 | 1 | rs35867827 | 6:32592068_T_C |
| PGP000050_Graff_2021 | Graff (2021) | rs190788477 | A | 6:32597561 | 0 | 1 | 1 | rs9271971 | 6:32597561_G_A |
| PGP000050_Graff_2021 | Graff (2021) | rs115566240 | C | 6:32601718 | 1 | 1 | 1 | rs115566240 | 6:32601718_C_T |
| PGP000050_Graff_2021 | Graff (2021) | rs9272307 | T | 6:32603886 | 1 | 1 | 1 | rs9272307 | 6:32603886_G_T |
| PGP000050_Graff_2021 | Graff (2021) | rs1048372 | C | 6:32610436 | 1 | 1 | 1 | rs1048372 | 6:32610436_T_C |
| PGP000050_Graff_2021 | Graff (2021) | rs9273429 | A | 6:32627456 | 1 | 1 | 1 | rs9273429 | 6:32627456_G_A |
| PGP000050_Graff_2021 | Graff (2021) | rs114830752 | A | 6:32682812 | 0 | 1 | 1 | rs9275602 | rs9275602 |
| PGP000050_Graff_2021 | Graff (2021) | rs116036768 | G | 6:32747542 | 0 | 1 | 1 | rs2621408 | 6:32747542_C_G |
| PGP000050_Graff_2021 | Graff (2021) | rs7383287 | G | 6:32783086 | 1 | 1 | 1 | rs7383287 | rs7383287 |
| PGP000050_Graff_2021 | Graff (2021) | rs239935 | G | 6:167411788 | 1 | 1 | 1 | rs239935 | 6:167411788_G_A |
| PGP000050_Graff_2021 | Graff (2021) | rs11780471 | G | 8:27344719 | 1 | 1 | 1 | rs11780471 | 8:27344719_G_A |
| PGP000050_Graff_2021 | Graff (2021) | rs4236709 | G | 8:32410110 | 1 | 1 | 1 | rs4236709 | 8:32410110_G_A |
| PGP000050_Graff_2021 | Graff (2021) | rs885518 | G | 9:21830157 | 1 | 1 | 1 | rs885518 | 9:21830157_A_G |
| PGP000050_Graff_2021 | Graff (2021) | rs62560775 | G | 9:22052068 | 1 | 1 | 1 | rs62560775 | 9:22052068_A_G |
| PGP000050_Graff_2021 | Graff (2021) | rs1333040 | C | 9:22083404 | 1 | 1 | 1 | rs1333040 | rs1333040 |
| PGP000050_Graff_2021 | Graff (2021) | rs11591710 | C | 10:105687632 | 1 | 1 | 1 | rs11591710 | 10:105687632_A_C |
| PGP000050_Graff_2021 | Graff (2021) | rs7943272 | C | 11:118096028 | 1 | 1 | 1 | rs7943272 | 11:118096028_A_C |
| PGP000050_Graff_2021 | Graff (2021) | rs17121997 | T | 11:118131049 | 1 | 1 | 1 | rs17121997 | 11:118131049_T_C |
| PGP000050_Graff_2021 | Graff (2021) | rs7953330 | G | 12:998819 | 1 | 1 | 1 | rs7953330 | 12:998819_G_C |
| PGP000050_Graff_2021 | Graff (2021) | rs11064614 | A | 12:1064881 | 1 | 1 | 1 | rs11064614 | 12:1064881_G_A |
| PGP000050_Graff_2021 | Graff (2021) | rs56404467 | A | 13:32839990 | 1 | 1 | 1 | rs56404467 | 13:32839990_G_A |
| PGP000050_Graff_2021 | Graff (2021) | rs748404 | T | 15:43559231 | 1 | 1 | 1 | rs748404 | rs748404 |
| PGP000050_Graff_2021 | Graff (2021) | rs66759488 | A | 15:47577451 | 1 | 1 | 1 | rs66759488 | 15:47577451_G_A |
| PGP000050_Graff_2021 | Graff (2021) | rs8031813 | A | 15:49253961 | 1 | 1 | 1 | rs8031813 | 15:49253961_C_A |
| PGP000050_Graff_2021 | Graff (2021) | rs7359317 | G | 15:49276416 | 1 | 1 | 1 | rs7359317 | 15:49276416_G_A |
| PGP000050_Graff_2021 | Graff (2021) | rs146782107 | T | 15:49366480 | 1 | 1 | 1 | rs146782107 | 15:49366480_T_C |
| PGP000050_Graff_2021 | Graff (2021) | rs4887059 | T | 15:78782095 | 1 | 1 | 1 | rs4887059 | 15:78782095_C_T |
| PGP000050_Graff_2021 | Graff (2021) | rs3885951 | G | 15:78825917 | 1 | 1 | 1 | rs3885951 | rs3885951 |
| PGP000050_Graff_2021 | Graff (2021) | rs55781567 | G | 15:78857986 | 1 | 1 | 1 | rs55781567 | rs55781567 |
| PGP000050_Graff_2021 | Graff (2021) | rs56219480 | C | 15:78864024 | 1 | 1 | 1 | rs56219480 | rs56219480 |
| PGP000050_Graff_2021 | Graff (2021) | rs2229961 | A | 15:78880752 | 1 | 1 | 1 | rs2229961 | rs2229961 |
| PGP000050_Graff_2021 | Graff (2021) | rs3743078 | G | 15:78894759 | 1 | 1 | 1 | rs3743078 | 15:78894759_C_G |
| PGP000050_Graff_2021 | Graff (2021) | rs77438700 | A | 15:78906637 | 1 | 1 | 1 | rs77438700 | 15:78906637_A_G |
| PGP000050_Graff_2021 | Graff (2021) | rs8192479 | T | 15:78909398 | 1 | 1 | 1 | rs8192479 | rs8192479 |
| PGP000050_Graff_2021 | Graff (2021) | rs17487514 | T | 15:78953785 | 1 | 1 | 1 | rs17487514 | 15:78953785_C_T |
| PGP000050_Graff_2021 | Graff (2021) | rs143527274 | C | 15:78953924 | 1 | 1 | 1 | rs143527274 | 15:78953924_C_T |
| PGP000050_Graff_2021 | Graff (2021) | rs148832167 | A | 15:79018698 | 0 | 1 | 1 | rs1808065 | 15:79018698_G_A |
| PGP000050_Graff_2021 | Graff (2021) | rs12905116 | A | 15:79042734 | 1 | 1 | 1 | rs12905116 | 15:79042734_T_A |
| PGP000050_Graff_2021 | Graff (2021) | rs3861180 | G | 15:79043393 | 1 | 1 | 1 | rs3861180 | 15:79043393_A_G |
| PGP000050_Graff_2021 | Graff (2021) | rs112827102 | T | 15:79058968 | 1 | 1 | 1 | rs112827102 | 15:79058968_T_C |
| PGP000050_Graff_2021 | Graff (2021) | rs28450923 | A | 15:79065557 | 1 | 1 | 1 | rs28450923 | 15:79065557_A_G |
| PGP000050_Graff_2021 | Graff (2021) | rs36061084 | A | 15:79074253 | 1 | 1 | 1 | rs36061084 | 15:79074253_G_A |
| PGP000050_Graff_2021 | Graff (2021) | rs7182809 | C | 15:79105106 | 1 | 1 | 1 | rs7182809 | 15:79105106_G_C |
| PGP000050_Graff_2021 | Graff (2021) | rs77719127 | T | 15:79110783 | 1 | 1 | 1 | rs77719127 | 15:79110783_C_T |
| PGP000050_Graff_2021 | Graff (2021) | rs11072811 | A | 15:79132330 | 1 | 1 | 1 | rs11072811 | 15:79132330_A_C |
| PGP000050_Graff_2021 | Graff (2021) | rs1551821 | A | 18:42866470 | 1 | 1 | 1 | rs1551821 | 18:42866470_A_C |
| PGP000050_Graff_2021 | Graff (2021) | rs11879413 | C | 19:41337923 | 1 | 1 | 1 | rs11879413 | 19:41337923_C_T |
| PGP000050_Graff_2021 | Graff (2021) | rs56113850 | C | 19:41353107 | 1 | 1 | 1 | rs56113850 | 19:41353107_T_C |
| PGP000050_Graff_2021 | Graff (2021) | rs67210567 | G | 19:41357457 | 1 | 1 | 1 | rs67210567 | 19:41357457_G_T |
| PGP000050_Graff_2021 | Graff (2021) | rs41309931 | T | 20:62326579 | 1 | 1 | 1 | rs41309931 | 20:62326579_G_T |
| PGP000050_Graff_2021 | Graff (2021) | rs504022 | A | 21:44731138 | 1 | 1 | 1 | rs504022 | 21:44731138_T_G |
| PGP000075_Shi_2019 | Shi (2019) | rs13314271 | T | 3:189357602 | 1 | 1 | 1 | rs13314271 | 3:189357602_T_C |
| PGP000075_Shi_2019 | Shi (2019) | rs9258375 | G | 6:29752808 | 1 | 1 | 1 | rs9258375 | 6:29752808_A_G |

## S9 List of Included SNPs

| PGS_name | Label | rsID | Effect allele | Position | rsID in UKB extract | position in UKB extract | found in UKB extract | rsID in UKB | Position in UKB |
|---|---|---|---|---|---|---|---|---|---|
| PGP000075_Shi_2019 | Shi (2019) | rs66759488 | A | 15:47577451 | 1 | 1 | 1 | rs66759488 | 15:47577451_G_A |
| PGP000075_Shi_2019 | Shi (2019) | rs4887053 | C | 15:78712699 | 1 | 1 | 1 | rs4887053 | 15:78712699_A_C |
| PGP000075_Shi_2019 | Shi (2019) | rs1051730 | A | 15:78894339 | 1 | 1 | 1 | rs1051730 | rs1051730 |
| PGP000075_Shi_2019 | Shi (2019) | rs6495309 | T | 15:78915245 | 1 | 1 | 1 | rs6495309 | 15:78915245_C_T |
| PGP000135_Jia_2020 | Jia (2020) | rs71658797 | A | 1:77967507 | 1 | 1 | 1 | rs71658797 | 1:77967507_T_A |
| PGP000135_Jia_2020 | Jia (2020) | rs13080835 | G | 3:189357199 | 1 | 1 | 1 | rs13080835 | 3:189357199_G_T |
| PGP000135_Jia_2020 | Jia (2020) | rs7705526 | A | 5:1285974 | 1 | 1 | 1 | rs7705526 | 5:1285974_C_A |
| PGP000135_Jia_2020 | Jia (2020) | rs2853677 | G | 5:1287194 | 1 | 1 | 1 | rs2853677 | rs2853677 |
| PGP000135_Jia_2020 | Jia (2020) | rs465498 | A | 5:1325803 | 1 | 1 | 1 | rs465498 | 5:1325803_A_G |
| PGP000135_Jia_2020 | Jia (2020) | rs3115672 | T | 6:31727897 | 1 | 1 | 1 | rs3115672 | 6:31727897_C_T |
| PGP000135_Jia_2020 | Jia (2020) | rs6920364 | C | 6:167376466 | 1 | 1 | 1 | rs6920364 | 6:167376466_G_C |
| PGP000135_Jia_2020 | Jia (2020) | rs4236709 | G | 8:32410110 | 1 | 1 | 1 | rs4236709 | 8:32410110_G_A |
| PGP000135_Jia_2020 | Jia (2020) | rs11780471 | G | 8:27344719 | 1 | 1 | 1 | rs11780471 | 8:27344719_G_A |
| PGP000135_Jia_2020 | Jia (2020) | rs885518 | G | 9:21830157 | 1 | 1 | 1 | rs885518 | 9:21830157_A_G |
| PGP000135_Jia_2020 | Jia (2020) | rs62560775 | G | 9:22052068 | 1 | 1 | 1 | rs62560775 | 9:22052068_A_G |
| PGP000135_Jia_2020 | Jia (2020) | rs1056562 | T | 11:118125625 | 1 | 1 | 1 | rs1056562 | 11:118125625_T_C |
| PGP000135_Jia_2020 | Jia (2020) | rs7953330 | G | 12:998819 | 1 | 1 | 1 | rs7953330 | 12:998819_G_C |
| PGP000135_Jia_2020 | Jia (2020) | rs11571833 | T | 13:32972626 | 1 | 1 | 1 | rs11571833 | rs11571833 |
| PGP000135_Jia_2020 | Jia (2020) | rs66759488 | A | 15:47577451 | 1 | 1 | 1 | rs66759488 | 15:47577451_G_A |
| PGP000135_Jia_2020 | Jia (2020) | rs77468143 | T | 15:49376624 | 1 | 1 | 1 | rs77468143 | 15:49376624_T_G |
| PGP000135_Jia_2020 | Jia (2020) | rs55781567 | G | 15:78857986 | 1 | 1 | 1 | rs55781567 | rs55781567 |
| PGP000135_Jia_2020 | Jia (2020) | rs8042374 | A | 15:78908032 | 1 | 1 | 1 | rs8042374 | 15:78908032_A_G |
| PGP000135_Jia_2020 | Jia (2020) | rs56113850 | C | 19:41353107 | 1 | 1 | 1 | rs56113850 | 19:41353107_T_C |
| PGP000148_Hung_2021 | Hung (2021) | rs71658797 | A | 1:77967507 | 1 | 1 | 1 | rs71658797 | 1:77967507_T_A |
| PGP000148_Hung_2021 | Hung (2021) | rs13080835 | T | 3:189357199 | 1 | 1 | 1 | rs13080835 | 3:189357199_G_T |
| PGP000148_Hung_2021 | Hung (2021) | rs7705526 | A | 5:1285974 | 1 | 1 | 1 | rs7705526 | 5:1285974_C_A |
| PGP000148_Hung_2021 | Hung (2021) | rs112290073 | A | 5:1286032 | 1 | 1 | 1 | rs112290073 | 5:1286032_G_A |
| PGP000148_Hung_2021 | Hung (2021) | rs2736098 | T | 5:1294086 | 1 | 1 | 1 | rs2736098 | 5:1294086_C_T |
| PGP000148_Hung_2021 | Hung (2021) | rs2853668 | T | 5:1300025 | 1 | 1 | 1 | rs2853668 | 5:1300025_G_T |
| PGP000148_Hung_2021 | Hung (2021) | rs401681 | T | 5:1322087 | 1 | 1 | 1 | rs401681 | rs401681 |
| PGP000148_Hung_2021 | Hung (2021) | rs466502 | G | 5:1325767 | 1 | 1 | 1 | rs466502 | 5:1325767_A_G |
| PGP000148_Hung_2021 | Hung (2021) | rs6903823 | G | 6:28354519 | 0 | 0 | 0 | NA | NA |
| PGP000148_Hung_2021 | Hung (2021) | rs116822326 | G | 6:31434111 | 0 | 1 | 1 | rs3094604 | 6:31434111_A_G |
| PGP000148_Hung_2021 | Hung (2021) | rs2855812 | T | 6:31504943 | 0 | 0 | 0 | NA | NA |
| PGP000148_Hung_2021 | Hung (2021) | rs805262 | T | 6:31628733 | 1 | 1 | 1 | rs805262 | rs805262 |
| PGP000148_Hung_2021 | Hung (2021) | rs6916278 | A | 6:31678774 | 1 | 1 | 1 | rs6916278 | rs6916278 |
| PGP000148_Hung_2021 | Hung (2021) | rs3129763 | A | 6:32590925 | 1 | 1 | 1 | rs3129763 | 6:32590925_G_A |
| PGP000148_Hung_2021 | Hung (2021) | rs114544105 | A | 6:32667852 | 0 | 0 | 0 | NA | NA |
| PGP000148_Hung_2021 | Hung (2021) | rs6920364 | C | 6:167376466 | 1 | 1 | 1 | rs6920364 | 6:167376466_G_C |
| PGP000148_Hung_2021 | Hung (2021) | rs11780471 | A | 8:27344719 | 1 | 1 | 1 | rs11780471 | 8:27344719_G_A |
| PGP000148_Hung_2021 | Hung (2021) | rs4236709 | G | 8:32410110 | 1 | 1 | 1 | rs4236709 | 8:32410110_G_A |
| PGP000148_Hung_2021 | Hung (2021) | rs885518 | G | 9:21830157 | 1 | 1 | 1 | rs885518 | 9:21830157_A_G |
| PGP000148_Hung_2021 | Hung (2021) | rs2007153 | T | 9:136503819 | 1 | 1 | 1 | rs2007153 | rs2007153 |
| PGP000148_Hung_2021 | Hung (2021) | rs11591710 | C | 10:105687632 | 1 | 1 | 1 | rs11591710 | 10:105687632_A_C |
| PGP000148_Hung_2021 | Hung (2021) | rs1056562 | T | 11:118125625 | 1 | 1 | 1 | rs1056562 | 11:118125625_T_C |
| PGP000148_Hung_2021 | Hung (2021) | rs7953330 | C | 12:998819 | 1 | 1 | 1 | rs7953330 | 12:998819_G_C |
| PGP000148_Hung_2021 | Hung (2021) | rs11571833 | T | 13:32972626 | 1 | 1 | 1 | rs11571833 | rs11571833 |
| PGP000148_Hung_2021 | Hung (2021) | rs689647 | T | 15:43762196 | 1 | 1 | 1 | rs689647 | rs689647 |
| PGP000148_Hung_2021 | Hung (2021) | rs66759488 | A | 15:47577451 | 1 | 1 | 1 | rs66759488 | 15:47577451_G_A |
| PGP000148_Hung_2021 | Hung (2021) | rs77468143 | G | 15:49376624 | 1 | 1 | 1 | rs77468143 | 15:49376624_T_G |
| PGP000148_Hung_2021 | Hung (2021) | rs3885951 | G | 15:78825917 | 1 | 1 | 1 | rs3885951 | rs3885951 |
| PGP000148_Hung_2021 | Hung (2021) | rs55781567 | G | 15:78857986 | 1 | 1 | 1 | rs55781567 | rs55781567 |
| PGP000148_Hung_2021 | Hung (2021) | rs7177699 | C | 15:79089734 | 1 | 1 | 1 | rs7177699 | 15:79089734_T_C |
| PGP000148_Hung_2021 | Hung (2021) | rs62070270 | G | 17:29936962 | 0 | 0 | 0 | NA | NA |
| PGP000148_Hung_2021 | Hung (2021) | rs1542752 | T | 17:72938100 | 1 | 1 | 1 | rs1542752 | rs1542752 |
| PGP000148_Hung_2021 | Hung (2021) | rs56113850 | T | 19:41353107 | 1 | 1 | 1 | rs56113850 | 19:41353107_T_C |
| PGP000148_Hung_2021 | Hung (2021) | rs41309931 | T | 20:62326579 | 1 | 1 | 1 | rs41309931 | 20:62326579_G_T |
| PGP000148_Hung_2021 | Hung (2021) | rs17879961 | G | 22:29121087 | 1 | 1 | 1 | rs17879961 | rs17879961 |
| PGP000148_Hung_2021 | Hung (2021) | rs71641333 | A | 1:78743005 | 1 | 1 | 1 | rs71641333 | 1:78743005_T_A |
| PGP000148_Hung_2021 | Hung (2021) | rs78062588 | C | 1:154566225 | 1 | 1 | 1 | rs78062588 | 1:154566225_T_C |
| PGP000148_Hung_2021 | Hung (2021) | rs114737056 | A | 1:168511081 | 1 | 1 | 1 | rs114737056 | 1:168511081_G_A |
| PGP000148_Hung_2021 | Hung (2021) | rs145733018 | C | 2:38567201 | 1 | 1 | 1 | rs145733018 | 2:38567201_T_C |
| PGP000148_Hung_2021 | Hung (2021) | rs79368540 | T | 2:45189737 | 1 | 1 | 1 | rs79368540 | 2:45189737_C_T |
| PGP000148_Hung_2021 | Hung (2021) | rs11692700 | C | 2:67510377 | 1 | 1 | 1 | rs11692700 | 2:67510377_T_C |
| PGP000148_Hung_2021 | Hung (2021) | rs114928225 | A | 2:119449740 | 1 | 1 | 1 | rs114928225 | 2:119449740_T_A |

## S9 List of Included SNPs

| PGS_name | Label | rsID | Effect allele | Position | rsID in UKB extract | position in UKB extract | found in UKB extract | rsID in UKB | Position in UKB |
|---|---|---|---|---|---|---|---|---|---|
| PGP000148_Hung_2021 | Hung (2021) | rs7592999 | C | 2:140398327 | 1 | 1 | 1 | rs7592999 | 2:140398327_T_C |
| PGP000148_Hung_2021 | Hung (2021) | rs722864 | A | 2:173983204 | 1 | 1 | 1 | rs722864 | 2:173983204_A_G |
| PGP000148_Hung_2021 | Hung (2021) | rs1866631 | G | 2:174075761 | 1 | 1 | 1 | rs1866631 | 2:174075761_G_A |
| PGP000148_Hung_2021 | Hung (2021) | rs185666783 | G | 4:67833774 | 1 | 1 | 1 | rs185666783 | 4:67833774_C_G |
| PGP000148_Hung_2021 | Hung (2021) | rs7676823 | G | 4:164007992 | 1 | 1 | 1 | rs7676823 | 4:164007992_G_A |
| PGP000148_Hung_2021 | Hung (2021) | rs78154696 | A | 5:1000156 | 1 | 1 | 1 | rs78154696 | 5:1000156_G_A |
| PGP000148_Hung_2021 | Hung (2021) | rs112333466 | T | 5:1249816 | 1 | 1 | 1 | rs112333466 | 5:1249816_C_T |
| PGP000148_Hung_2021 | Hung (2021) | rs56345976 | G | 5:1276873 | 1 | 1 | 1 | rs56345976 | 5:1276873_G_A |
| PGP000148_Hung_2021 | Hung (2021) | rs2853677 | G | 5:1287194 | 1 | 1 | 1 | rs2853677 | rs2853677 |
| PGP000148_Hung_2021 | Hung (2021) | rs112401627 | A | 5:1300269 | 1 | 1 | 1 | rs112401627 | 5:1300269_G_A |
| PGP000148_Hung_2021 | Hung (2021) | rs6875416 | T | 5:90250631 | 1 | 1 | 1 | rs6875416 | 5:90250631_A_T |
| PGP000148_Hung_2021 | Hung (2021) | rs114136906 | C | 5:150121458 | 1 | 1 | 1 | rs114136906 | 5:150121458_G_C |
| PGP000148_Hung_2021 | Hung (2021) | rs2316515 | A | 6:410848 | 1 | 1 | 1 | rs2316515 | 6:410848_A_G |
| PGP000148_Hung_2021 | Hung (2021) | rs629444 | T | 6:25885814 | 1 | 1 | 1 | rs629444 | rs629444 |
| PGP000148_Hung_2021 | Hung (2021) | rs2179517 | C | 6:26198845 | 1 | 1 | 1 | rs2179517 | 6:26198845_G_C |
| PGP000148_Hung_2021 | Hung (2021) | rs68141011 | T | 6:28217797 | 1 | 1 | 1 | rs68141011 | 6:28217797_G_A |
| PGP000148_Hung_2021 | Hung (2021) | rs114722608 | C | 6:29223493 | 0 | 1 | 1 | rs3130825 | 6:29223493_G_C |
| PGP000148_Hung_2021 | Hung (2021) | rs115123779 | T | 6:29477821 | 0 | 1 | 1 | rs1233478 | rs1233478 |
| PGP000148_Hung_2021 | Hung (2021) | rs138488080 | A | 6:29606761 | 0 | 1 | 1 | rs3095268 | 6:29606761_G_A |
| PGP000148_Hung_2021 | Hung (2021) | rs114192654 | A | 6:29759750 | 0 | 1 | 1 | rs1611203 | 6:29759750_G_A |
| PGP000148_Hung_2021 | Hung (2021) | rs116675020 | G | 6:29922740 | 0 | 1 | 1 | rs16896742 | rs16896742 |
| PGP000148_Hung_2021 | Hung (2021) | rs115993819 | A | 6:30074163 | 0 | 1 | 1 | rs2517600 | rs2517600 |
| PGP000148_Hung_2021 | Hung (2021) | rs116534499 | G | 6:30138162 | 0 | 1 | 1 | rs1029239 | rs1029239 |
| PGP000148_Hung_2021 | Hung (2021) | rs116629156 | C | 6:30864829 | 0 | 1 | 1 | rs1049623 | rs1049623 |
| PGP000148_Hung_2021 | Hung (2021) | rs114103504 | G | 6:31002452 | 0 | 1 | 1 | rs3869098 | 6:31002452_A_G |
| PGP000148_Hung_2021 | Hung (2021) | rs114052224 | G | 6:31067852 | 0 | 1 | 1 | rs3130548 | 6:31067852_A_G |
| PGP000148_Hung_2021 | Hung (2021) | rs2233959 | C | 6:31081065 | 1 | 1 | 1 | rs2233959 | 6:31081065_T_C |
| PGP000148_Hung_2021 | Hung (2021) | rs114689412 | G | 6:31117577 | 0 | 1 | 1 | rs1265113 | 6:31117577_C_G |
| PGP000148_Hung_2021 | Hung (2021) | rs2596499 | A | 6:31321429 | 1 | 1 | 1 | rs2596499 | 6:31321429_T_A |
| PGP000148_Hung_2021 | Hung (2021) | rs2596496 | C | 6:31322782 | 1 | 1 | 1 | rs2596496 | 6:31322782_C_G |
| PGP000148_Hung_2021 | Hung (2021) | rs2596490 | G | 6:31324996 | 1 | 1 | 1 | rs2596490 | 6:31324996_G_A |
| PGP000148_Hung_2021 | Hung (2021) | rs115176861 | C | 6:31412961 | 0 | 1 | 1 | rs2596464 | Affx-28456540 |
| PGP000148_Hung_2021 | Hung (2021) | rs553108 | A | 6:31840455 | 1 | 1 | 1 | rs553108 | 6:31840455_A_G |
| PGP000148_Hung_2021 | Hung (2021) | rs115200960 | A | 6:32335204 | 0 | 1 | 1 | rs2143462 | rs2143462 |
| PGP000148_Hung_2021 | Hung (2021) | rs12722051 | T | 6:32609147 | 1 | 1 | 1 | rs12722051 | 6:32609147_A_T |
| PGP000148_Hung_2021 | Hung (2021) | rs116767258 | G | 6:32757737 | 0 | 1 | 1 | rs2857165 | rs2857165 |
| PGP000148_Hung_2021 | Hung (2021) | rs7383287 | G | 6:32783086 | 1 | 1 | 1 | rs7383287 | rs7383287 |
| PGP000148_Hung_2021 | Hung (2021) | rs117534741 | A | 6:72384541 | 1 | 1 | 1 | rs117534741 | rs117534741 |
| PGP000148_Hung_2021 | Hung (2021) | rs1321817 | G | 6:117734267 | 1 | 1 | 1 | rs1321817 | 6:117734267_A_G |
| PGP000148_Hung_2021 | Hung (2021) | rs6957511 | C | 7:130668618 | 1 | 1 | 1 | rs6957511 | 7:130668618_C_T |
| PGP000148_Hung_2021 | Hung (2021) | rs2565064 | C | 8:27327841 | 1 | 1 | 1 | rs2565064 | 8:27327841_C_G |
| PGP000148_Hung_2021 | Hung (2021) | rs67749759 | T | 8:27397087 | 0 | 1 | 1 | rs4149254 | 8:27397087_C_T |
| PGP000148_Hung_2021 | Hung (2021) | rs111960002 | C | 8:144722420 | 1 | 1 | 1 | rs111960002 | 8:144722420_T_C |
| PGP000148_Hung_2021 | Hung (2021) | rs10118776 | G | 9:6227418 | 1 | 1 | 1 | rs10118776 | 9:6227418_G_A |
| PGP000148_Hung_2021 | Hung (2021) | rs17185553 | C | 9:17934120 | 1 | 1 | 1 | rs17185553 | 9:17934120_G_C |
| PGP000148_Hung_2021 | Hung (2021) | rs2518717 | C | 9:21959751 | 1 | 1 | 1 | rs2518717 | 9:21959751_T_C |
| PGP000148_Hung_2021 | Hung (2021) | rs28557075 | A | 9:22066572 | 1 | 1 | 1 | rs28557075 | 9:22066572_G_A |
| PGP000148_Hung_2021 | Hung (2021) | rs1333040 | C | 9:22083404 | 1 | 1 | 1 | rs1333040 | rs1333040 |
| PGP000148_Hung_2021 | Hung (2021) | rs4879704 | C | 9:33427322 | 1 | 1 | 1 | rs4879704 | 9:33427322_A_C |
| PGP000148_Hung_2021 | Hung (2021) | rs191205566 | T | 9:102587233 | 1 | 1 | 1 | rs191205566 | 9:102587233_C_T |
| PGP000148_Hung_2021 | Hung (2021) | rs75685923 | T | 9:136275229 | 1 | 1 | 1 | rs75685923 | rs75685923 |
| PGP000148_Hung_2021 | Hung (2021) | rs7897454 | A | 10:102011702 | 1 | 1 | 1 | rs7897454 | 10:102011702_G_A |
| PGP000148_Hung_2021 | Hung (2021) | rs62621207 | T | 10:102672248 | 1 | 1 | 1 | rs62621207 | 10:102672248_A_T |
| PGP000148_Hung_2021 | Hung (2021) | rs78853063 | T | 11:57250026 | 1 | 1 | 1 | rs78853063 | 11:57250026_C_T |
| PGP000148_Hung_2021 | Hung (2021) | rs78334599 | A | 11:115998756 | 1 | 1 | 1 | rs78334599 | 11:115998756_G_A |
| PGP000148_Hung_2021 | Hung (2021) | rs7487683 | T | 12:1036042 | 1 | 1 | 1 | rs7487683 | rs7487683 |
| PGP000148_Hung_2021 | Hung (2021) | rs73351723 | A | 12:58831070 | 1 | 1 | 1 | rs73351723 | 12:58831070_G_A |
| PGP000148_Hung_2021 | Hung (2021) | rs9668978 | T | 12:64913237 | 1 | 1 | 1 | rs9668978 | rs9668978 |
| PGP000148_Hung_2021 | Hung (2021) | rs9602270 | T | 13:84281063 | 1 | 1 | 1 | rs9602270 | 13:84281063_A_T |
| PGP000148_Hung_2021 | Hung (2021) | rs8003466 | A | 14:34013721 | 1 | 1 | 1 | rs8003466 | 14:34013721_G_A |
| PGP000148_Hung_2021 | Hung (2021) | rs8031813 | C | 15:49253961 | 1 | 1 | 1 | rs8031813 | 15:49253961_C_A |
| PGP000148_Hung_2021 | Hung (2021) | rs6493361 | G | 15:49615952 | 1 | 1 | 1 | rs6493361 | 15:49615952_G_C |
| PGP000148_Hung_2021 | Hung (2021) | rs11855650 | T | 15:70431773 | 1 | 1 | 1 | rs11855650 | rs11855650 |
| PGP000148_Hung_2021 | Hung (2021) | rs79149102 | T | 15:75055819 | 1 | 1 | 1 | rs79149102 | 15:75055819_C_T |
| PGP000148_Hung_2021 | Hung (2021) | rs2229961 | A | 15:78880752 | 1 | 1 | 1 | rs2229961 | rs2229961 |

## S9 List of Included SNPs

| PGS_name | Label | rsID | Effect allele | Position | rsID in UKB extract | position in UKB extract | found in UKB extract | rsID in UKB | Position in UKB |
|---|---|---|---|---|---|---|---|---|---|
| PGP000148_Hung_2021 | Hung (2021) | rs8192479 | T | 15:78909398 | 1 | 1 | 1 | rs8192479 | rs8192479 |
| PGP000148_Hung_2021 | Hung (2021) | rs2869551 | G | 15:78981423 | 1 | 1 | 1 | rs2869551 | 15:78981423_A_G |
| PGP000148_Hung_2021 | Hung (2021) | rs12593207 | A | 15:78987225 | 1 | 1 | 1 | rs12593207 | rs12593207 |
| PGP000148_Hung_2021 | Hung (2021) | rs189146505 | G | 15:79058730 | 1 | 1 | 1 | rs189146505 | 15:79058730_A_G |
| PGP000148_Hung_2021 | Hung (2021) | rs28450923 | G | 15:79065557 | 1 | 1 | 1 | rs28450923 | 15:79065557_A_G |
| PGP000148_Hung_2021 | Hung (2021) | rs28624856 | C | 15:79075233 | 1 | 1 | 1 | rs28624856 | 15:79075233_C_T |
| PGP000148_Hung_2021 | Hung (2021) | rs77719127 | T | 15:79110783 | 1 | 1 | 1 | rs77719127 | 15:79110783_C_T |
| PGP000148_Hung_2021 | Hung (2021) | rs76164573 | G | 15:79198760 | 1 | 1 | 1 | rs76164573 | rs76164573 |
| PGP000148_Hung_2021 | Hung (2021) | rs78442819 | C | 16:10740982 | 1 | 1 | 1 | rs78442819 | 16:10740982_G_C |
| PGP000148_Hung_2021 | Hung (2021) | rs9926896 | C | 16:26980646 | 1 | 1 | 1 | rs9926896 | 16:26980646_T_C |
| PGP000148_Hung_2021 | Hung (2021) | rs17181550 | G | 17:70299958 | 1 | 1 | 1 | rs17181550 | rs17181550 |
| PGP000148_Hung_2021 | Hung (2021) | rs79421398 | C | 18:20741135 | 1 | 1 | 1 | rs79421398 | 18:20741135_T_C |
| PGP000148_Hung_2021 | Hung (2021) | rs66500423 | C | 19:41195170 | 1 | 1 | 1 | rs66500423 | 19:41195170_T_C |
| PGP000148_Hung_2021 | Hung (2021) | rs4803356 | G | 19:41207206 | 1 | 1 | 1 | rs4803356 | 19:41207206_C_G |
| PGP000148_Hung_2021 | Hung (2021) | rs11881918 | A | 19:41334199 | 1 | 1 | 1 | rs11881918 | 19:41334199_G_A |
| PGP000148_Hung_2021 | Hung (2021) | rs2258380 | G | 19:41338988 | 1 | 1 | 1 | rs2258380 | Affx-36248730 |
| PGP000148_Hung_2021 | Hung (2021) | rs67210567 | T | 19:41357457 | 1 | 1 | 1 | rs67210567 | 19:41357457_G_T |
| PGP000148_Hung_2021 | Hung (2021) | rs184589612 | C | 19:41412192 | 1 | 1 | 1 | rs184589612 | 19:41412192_T_C |
| PGP000148_Hung_2021 | Hung (2021) | rs12981718 | A | 19:54567858 | 1 | 1 | 1 | rs12981718 | 19:54567858_G_A |
| PGP000148_Hung_2021 | Hung (2021) | rs13036436 | G | 20:61988382 | 1 | 1 | 1 | rs13036436 | 20:61988382_G_A |
| PGP000148_Hung_2021 | Hung (2021) | rs61541144 | A | 20:62527305 | 1 | 1 | 1 | rs61541144 | 20:62527305_G_A |
| PGP000186_Kachuri_2020 | Kachuri (2020) | rs71658797 | A | 1:77967507 | 1 | 1 | 1 | rs71658797 | 1:77967507_T_A |
| PGP000186_Kachuri_2020 | Kachuri (2020) | rs13080835 | G | 3:189357199 | 1 | 1 | 1 | rs13080835 | 3:189357199_G_T |
| PGP000186_Kachuri_2020 | Kachuri (2020) | rs71603396 | A | 4:18012035 | 1 | 1 | 1 | rs71603396 | 4:18012035_G_A |
| PGP000186_Kachuri_2020 | Kachuri (2020) | rs13156167 | C | 5:1275857 | 1 | 1 | 1 | rs13156167 | 5:1275857_T_C |
| PGP000186_Kachuri_2020 | Kachuri (2020) | rs56345976 | G | 5:1276873 | 1 | 1 | 1 | rs56345976 | 5:1276873_G_A |
| PGP000186_Kachuri_2020 | Kachuri (2020) | rs33961405 | G | 5:1277577 | 1 | 1 | 1 | rs33961405 | 5:1277577_G_A |
| PGP000186_Kachuri_2020 | Kachuri (2020) | rs7705526 | A | 5:1285974 | 1 | 1 | 1 | rs7705526 | 5:1285974_C_A |
| PGP000186_Kachuri_2020 | Kachuri (2020) | rs2853677 | G | 5:1287194 | 1 | 1 | 1 | rs2853677 | rs2853677 |
| PGP000186_Kachuri_2020 | Kachuri (2020) | rs148297846 | A | 5:1298017 | 1 | 1 | 1 | rs148297846 | 5:1298017_G_A |
| PGP000186_Kachuri_2020 | Kachuri (2020) | rs62329683 | T | 5:1299087 | 1 | 1 | 1 | rs62329683 | 5:1299087_C_T |
| PGP000186_Kachuri_2020 | Kachuri (2020) | rs112401627 | A | 5:1300269 | 1 | 1 | 1 | rs112401627 | 5:1300269_G_A |
| PGP000186_Kachuri_2020 | Kachuri (2020) | rs4635969 | G | 5:1308552 | 1 | 1 | 1 | rs4635969 | rs4635969 |
| PGP000186_Kachuri_2020 | Kachuri (2020) | rs380286 | G | 5:1320247 | 1 | 1 | 1 | rs380286 | 5:1320247_G_A |
| PGP000186_Kachuri_2020 | Kachuri (2020) | rs410805 | T | 5:1323196 | 1 | 1 | 1 | rs410805 | 5:1323196_T_C |
| PGP000186_Kachuri_2020 | Kachuri (2020) | rs55901723 | T | 5:1342154 | 0 | 1 | 1 | rs391314 | 5:1342154_T_C |
| PGP000186_Kachuri_2020 | Kachuri (2020) | rs40183 | G | 5:1348352 | 1 | 1 | 1 | rs40183 | 5:1348352_G_A |
| PGP000186_Kachuri_2020 | Kachuri (2020) | rs6889886 | C | 5:1358822 | 1 | 1 | 1 | rs6889886 | 5:1358822_C_T |
| PGP000186_Kachuri_2020 | Kachuri (2020) | rs806794 | A | 6:26200677 | 1 | 1 | 1 | rs806794 | rs806794 |
| PGP000186_Kachuri_2020 | Kachuri (2020) | rs3999544 | C | 6:26285867 | 1 | 1 | 1 | rs3999544 | 6:26285867_C_T |
| PGP000186_Kachuri_2020 | Kachuri (2020) | rs9467711 | C | 6:26332605 | 1 | 1 | 1 | rs9467711 | rs9467711 |
| PGP000186_Kachuri_2020 | Kachuri (2020) | rs6912292 | A | 6:26511805 | 1 | 1 | 1 | rs6912292 | 6:26511805_C_A |
| PGP000186_Kachuri_2020 | Kachuri (2020) | rs116461399 | C | 6:28796071 | 0 | 1 | 1 | rs3131337 | 6:28796071_G_C |
| PGP000186_Kachuri_2020 | Kachuri (2020) | rs116045192 | T | 6:29272012 | 0 | 1 | 1 | rs3117426 | rs3117426 |
| PGP000186_Kachuri_2020 | Kachuri (2020) | rs114924489 | G | 6:29468809 | 0 | 1 | 1 | rs1233487 | rs1233487 |
| PGP000186_Kachuri_2020 | Kachuri (2020) | rs114050064 | C | 6:29698821 | 0 | 1 | 1 | rs1611350 | rs1611350 |
| PGP000186_Kachuri_2020 | Kachuri (2020) | rs115813114 | C | 6:29766085 | 0 | 1 | 1 | rs1632999 | 6:29766085_T_C |
| PGP000186_Kachuri_2020 | Kachuri (2020) | rs111597677 | C | 6:29829251 | 0 | 1 | 1 | rs1611713 | 6:29829251_A_C |
| PGP000186_Kachuri_2020 | Kachuri (2020) | rs115960997 | A | 6:29902109 | 0 | 1 | 1 | rs1611314 | 6:29902109_G_A |
| PGP000186_Kachuri_2020 | Kachuri (2020) | rs116405142 | G | 6:30066031 | 0 | 1 | 1 | rs916570 | rs916570 |
| PGP000186_Kachuri_2020 | Kachuri (2020) | rs115993819 | A | 6:30074163 | 0 | 1 | 1 | rs2517600 | rs2517600 |
| PGP000186_Kachuri_2020 | Kachuri (2020) | rs142696802 | T | 6:30190040 | 0 | 1 | 1 | rs2517602 | 6:30190040_C_T |
| PGP000186_Kachuri_2020 | Kachuri (2020) | rs116074010 | T | 6:30391975 | 0 | 1 | 1 | rs3130127 | 6:30391975_A_T |
| PGP000186_Kachuri_2020 | Kachuri (2020) | rs115915654 | T | 6:30769726 | 0 | 1 | 1 | rs1264372 | rs1264372 |
| PGP000186_Kachuri_2020 | Kachuri (2020) | rs116552815 | A | 6:30855907 | 0 | 1 | 1 | rs1264323 | rs1264323 |
| PGP000186_Kachuri_2020 | Kachuri (2020) | rs114103504 | A | 6:31002452 | 0 | 1 | 1 | rs3869098 | 6:31002452_A_G |
| PGP000186_Kachuri_2020 | Kachuri (2020) | rs115669859 | A | 6:31010200 | 0 | 1 | 1 | rs2508015 | rs2508015 |
| PGP000186_Kachuri_2020 | Kachuri (2020) | rs182364552 | A | 6:31069841 | 0 | 1 | 1 | rs9263573 | 6:31069841_T_A |
| PGP000186_Kachuri_2020 | Kachuri (2020) | rs3130552 | A | 6:31082127 | 1 | 1 | 1 | rs3130552 | rs3130552 |
| PGP000186_Kachuri_2020 | Kachuri (2020) | rs116651383 | C | 6:31138910 | 0 | 1 | 1 | rs3094191 | 6:31138910_T_C |
| PGP000186_Kachuri_2020 | Kachuri (2020) | rs115681412 | G | 6:31140741 | 0 | 1 | 1 | rs1265158 | rs1265158 |
| PGP000186_Kachuri_2020 | Kachuri (2020) | rs141707415 | A | 6:31239681 | 0 | 1 | 1 | rs9264669 | 6:31239681_A_T |
| PGP000186_Kachuri_2020 | Kachuri (2020) | rs111324505 | T | 6:31242082 | 0 | 1 | 1 | rs7764413 | 6:31242082_T_C |
| PGP000186_Kachuri_2020 | Kachuri (2020) | rs115494074 | T | 6:31313030 | 0 | 1 | 1 | rs2394982 | 6:31313030_C_T |
| PGP000186_Kachuri_2020 | Kachuri (2020) | rs139850307 | C | 6:31324586 | 0 | 1 | 1 | rs1050556 | 6:31324586_C_T |

**S9 List of Included SNPs**

| PGS_name | Label | rsID | Effect allele | Position | rsID in UKB extract | position in UKB extract | found in UKB extract | rsID in UKB | Position in UKB |
|---|---|---|---|---|---|---|---|---|---|
| PGP000186_Kachuri_2020 | Kachuri (2020) | rs2249935 | A | 6:31327178 | 1 | 1 | 1 | rs2249935 | 6:31327178_G_A |
| PGP000186_Kachuri_2020 | Kachuri (2020) | rs115471405 | C | 6:31335647 | 0 | 1 | 1 | rs2844571 | 6:31335647_T_C |
| PGP000186_Kachuri_2020 | Kachuri (2020) | rs115595430 | T | 6:31346445 | 0 | 1 | 1 | rs2523627 | rs2523627 |
| PGP000186_Kachuri_2020 | Kachuri (2020) | rs114965247 | G | 6:31350303 | 0 | 1 | 1 | rs2844535 | 6:31350303_T_G |
| PGP000186_Kachuri_2020 | Kachuri (2020) | rs139089278 | T | 6:31373174 | 0 | 1 | 1 | rs2442715 | 6:31373174_C_T |
| PGP000186_Kachuri_2020 | Kachuri (2020) | rs114279165 | G | 6:31425499 | 0 | 1 | 1 | rs3131621 | rs3131621 |
| PGP000186_Kachuri_2020 | Kachuri (2020) | rs116822326 | G | 6:31434111 | 0 | 1 | 1 | rs3094604 | 6:31434111_A_G |
| PGP000186_Kachuri_2020 | Kachuri (2020) | rs115441327 | C | 6:31613739 | 0 | 1 | 1 | rs3130048 | rs3130048 |
| PGP000186_Kachuri_2020 | Kachuri (2020) | rs2763981 | T | 6:31840021 | 1 | 1 | 1 | rs2763981 | 6:31840021_T_A |
| PGP000186_Kachuri_2020 | Kachuri (2020) | rs652888 | G | 6:31851234 | 1 | 1 | 1 | rs652888 | rs652888 |
| PGP000186_Kachuri_2020 | Kachuri (2020) | rs114676850 | T | 6:32208324 | 0 | 1 | 1 | rs424232 | rs424232 |
| PGP000186_Kachuri_2020 | Kachuri (2020) | rs9270868 | G | 6:32571117 | 1 | 1 | 1 | rs9270868 | 6:32571117_A_G |
| PGP000186_Kachuri_2020 | Kachuri (2020) | rs116080888 | C | 6:32586236 | 0 | 1 | 1 | rs11751024 | 6:32586236_C_A |
| PGP000186_Kachuri_2020 | Kachuri (2020) | rs113574092 | G | 6:32586787 | 0 | 1 | 1 | rs9271364 | 6:32586787_A_G |
| PGP000186_Kachuri_2020 | Kachuri (2020) | rs35867827 | T | 6:32592068 | 1 | 1 | 1 | rs35867827 | 6:32592068_T_C |
| PGP000186_Kachuri_2020 | Kachuri (2020) | rs190788477 | A | 6:32597561 | 0 | 1 | 1 | rs9271971 | 6:32597561_G_A |
| PGP000186_Kachuri_2020 | Kachuri (2020) | rs115566240 | C | 6:32601718 | 1 | 1 | 1 | rs115566240 | 6:32601718_C_T |
| PGP000186_Kachuri_2020 | Kachuri (2020) | rs9272307 | T | 6:32603886 | 1 | 1 | 1 | rs9272307 | 6:32603886_G_T |
| PGP000186_Kachuri_2020 | Kachuri (2020) | rs1048372 | C | 6:32610436 | 1 | 1 | 1 | rs1048372 | 6:32610436_T_C |
| PGP000186_Kachuri_2020 | Kachuri (2020) | rs9273429 | A | 6:32627456 | 1 | 1 | 1 | rs9273429 | 6:32627456_G_A |
| PGP000186_Kachuri_2020 | Kachuri (2020) | rs114830752 | A | 6:32682812 | 0 | 1 | 1 | rs9275602 | rs9275602 |
| PGP000186_Kachuri_2020 | Kachuri (2020) | rs116036768 | G | 6:32747542 | 0 | 1 | 1 | rs2621408 | 6:32747542_C_G |
| PGP000186_Kachuri_2020 | Kachuri (2020) | rs7383287 | G | 6:32783086 | 1 | 1 | 1 | rs7383287 | rs7383287 |
| PGP000186_Kachuri_2020 | Kachuri (2020) | rs239935 | G | 6:167411788 | 1 | 1 | 1 | rs239935 | 6:167411788_G_A |
| PGP000186_Kachuri_2020 | Kachuri (2020) | rs11780471 | G | 8:27344719 | 1 | 1 | 1 | rs11780471 | 8:27344719_G_A |
| PGP000186_Kachuri_2020 | Kachuri (2020) | rs4236709 | G | 8:32410110 | 1 | 1 | 1 | rs4236709 | 8:32410110_G_A |
| PGP000186_Kachuri_2020 | Kachuri (2020) | rs885518 | G | 9:21830157 | 1 | 1 | 1 | rs885518 | 9:21830157_A_G |
| PGP000186_Kachuri_2020 | Kachuri (2020) | rs62560775 | G | 9:22052068 | 1 | 1 | 1 | rs62560775 | 9:22052068_A_G |
| PGP000186_Kachuri_2020 | Kachuri (2020) | rs1333040 | C | 9:22083404 | 1 | 1 | 1 | rs1333040 | rs1333040 |
| PGP000186_Kachuri_2020 | Kachuri (2020) | rs11591710 | C | 10:105687632 | 1 | 1 | 1 | rs11591710 | 10:105687632_A_C |
| PGP000186_Kachuri_2020 | Kachuri (2020) | rs7943272 | C | 11:118096028 | 1 | 1 | 1 | rs7943272 | 11:118096028_A_C |
| PGP000186_Kachuri_2020 | Kachuri (2020) | rs17121997 | T | 11:118131049 | 1 | 1 | 1 | rs17121997 | 11:118131049_T_C |
| PGP000186_Kachuri_2020 | Kachuri (2020) | rs7953330 | G | 12:998819 | 1 | 1 | 1 | rs7953330 | 12:998819_G_C |
| PGP000186_Kachuri_2020 | Kachuri (2020) | rs11064614 | A | 12:1064881 | 1 | 1 | 1 | rs11064614 | 12:1064881_G_A |
| PGP000186_Kachuri_2020 | Kachuri (2020) | rs56404467 | A | 13:32839990 | 1 | 1 | 1 | rs56404467 | 13:32839990_G_A |
| PGP000186_Kachuri_2020 | Kachuri (2020) | rs748404 | T | 15:43559231 | 1 | 1 | 1 | rs748404 | rs748404 |
| PGP000186_Kachuri_2020 | Kachuri (2020) | rs66759488 | A | 15:47577451 | 1 | 1 | 1 | rs66759488 | 15:47577451_G_A |
| PGP000186_Kachuri_2020 | Kachuri (2020) | rs8031813 | A | 15:49253961 | 1 | 1 | 1 | rs8031813 | 15:49253961_C_A |
| PGP000186_Kachuri_2020 | Kachuri (2020) | rs7359317 | G | 15:49276416 | 1 | 1 | 1 | rs7359317 | 15:49276416_G_A |
| PGP000186_Kachuri_2020 | Kachuri (2020) | rs146782107 | T | 15:49366480 | 1 | 1 | 1 | rs146782107 | 15:49366480_T_C |
| PGP000186_Kachuri_2020 | Kachuri (2020) | rs4887059 | T | 15:78782095 | 1 | 1 | 1 | rs4887059 | 15:78782095_C_T |
| PGP000186_Kachuri_2020 | Kachuri (2020) | rs3885951 | G | 15:78825917 | 1 | 1 | 1 | rs3885951 | rs3885951 |
| PGP000186_Kachuri_2020 | Kachuri (2020) | rs55781567 | G | 15:78857986 | 1 | 1 | 1 | rs55781567 | rs55781567 |
| PGP000186_Kachuri_2020 | Kachuri (2020) | rs56219480 | C | 15:78864024 | 1 | 1 | 1 | rs56219480 | rs56219480 |
| PGP000186_Kachuri_2020 | Kachuri (2020) | rs2229961 | A | 15:78880752 | 1 | 1 | 1 | rs2229961 | rs2229961 |
| PGP000186_Kachuri_2020 | Kachuri (2020) | rs3743078 | G | 15:78894759 | 1 | 1 | 1 | rs3743078 | 15:78894759_C_G |
| PGP000186_Kachuri_2020 | Kachuri (2020) | rs77438700 | A | 15:78906637 | 1 | 1 | 1 | rs77438700 | 15:78906637_A_G |
| PGP000186_Kachuri_2020 | Kachuri (2020) | rs8192479 | T | 15:78909398 | 1 | 1 | 1 | rs8192479 | rs8192479 |
| PGP000186_Kachuri_2020 | Kachuri (2020) | rs17487514 | T | 15:78953785 | 1 | 1 | 1 | rs17487514 | 15:78953785_C_T |
| PGP000186_Kachuri_2020 | Kachuri (2020) | rs143527274 | C | 15:78953924 | 1 | 1 | 1 | rs143527274 | 15:78953924_C_T |
| PGP000186_Kachuri_2020 | Kachuri (2020) | rs148832167 | A | 15:79018698 | 0 | 1 | 1 | rs1808065 | 15:79018698_G_A |
| PGP000186_Kachuri_2020 | Kachuri (2020) | rs12905116 | A | 15:79042734 | 1 | 1 | 1 | rs12905116 | 15:79042734_T_A |
| PGP000186_Kachuri_2020 | Kachuri (2020) | rs3861180 | G | 15:79043393 | 1 | 1 | 1 | rs3861180 | 15:79043393_A_G |
| PGP000186_Kachuri_2020 | Kachuri (2020) | rs112827102 | T | 15:79058968 | 1 | 1 | 1 | rs112827102 | 15:79058968_T_C |
| PGP000186_Kachuri_2020 | Kachuri (2020) | rs28450923 | A | 15:79065557 | 1 | 1 | 1 | rs28450923 | 15:79065557_A_G |
| PGP000186_Kachuri_2020 | Kachuri (2020) | rs36061084 | A | 15:79074253 | 1 | 1 | 1 | rs36061084 | 15:79074253_G_A |
| PGP000186_Kachuri_2020 | Kachuri (2020) | rs7182809 | C | 15:79105106 | 1 | 1 | 1 | rs7182809 | 15:79105106_G_C |
| PGP000186_Kachuri_2020 | Kachuri (2020) | rs77719127 | T | 15:79110783 | 1 | 1 | 1 | rs77719127 | 15:79110783_C_T |
| PGP000186_Kachuri_2020 | Kachuri (2020) | rs11072811 | A | 15:79132330 | 1 | 1 | 1 | rs11072811 | 15:79132330_A_C |
| PGP000186_Kachuri_2020 | Kachuri (2020) | rs1551821 | A | 18:42866470 | 1 | 1 | 1 | rs1551821 | 18:42866470_A_C |
| PGP000186_Kachuri_2020 | Kachuri (2020) | rs11879413 | C | 19:41337923 | 1 | 1 | 1 | rs11879413 | 19:41337923_C_T |
| PGP000186_Kachuri_2020 | Kachuri (2020) | rs56113850 | C | 19:41353107 | 1 | 1 | 1 | rs56113850 | 19:41353107_T_C |
| PGP000186_Kachuri_2020 | Kachuri (2020) | rs67210567 | G | 19:41357457 | 1 | 1 | 1 | rs67210567 | 19:41357457_G_T |
| PGP000186_Kachuri_2020 | Kachuri (2020) | rs41309931 | T | 20:62326579 | 1 | 1 | 1 | rs41309931 | 20:62326579_G_T |
| PGP000186_Kachuri_2020 | Kachuri (2020) | rs504022 | A | 21:44731138 | 1 | 1 | 1 | rs504022 | 21:44731138_T_G |

## S9 List of Included SNPs

| PGS_name | Label | rsID | Effect allele | Position | rsID in UKB extract | position in UKB extract | found in UKB extract | rsID in UKB | Position in UKB |
|---|---|---|---|---|---|---|---|---|---|
| PGP000226_Xie_2021 | Xie (2021) | rs71658797 | A | 1:77967507 | 1 | 1 | 1 | rs71658797 | 1:77967507_T_A |
| PGP000226_Xie_2021 | Xie (2021) | rs13080835 | G | 3:189357199 | 1 | 1 | 1 | rs13080835 | 3:189357199_G_T |
| PGP000226_Xie_2021 | Xie (2021) | rs7705526 | A | 5:1285974 | 1 | 1 | 1 | rs7705526 | 5:1285974_C_A |
| PGP000226_Xie_2021 | Xie (2021) | rs116822326 | G | 6:31434111 | 0 | 1 | 1 | rs3094604 | 6:31434111_A_G |
| PGP000226_Xie_2021 | Xie (2021) | rs6920364 | C | 6:167376466 | 1 | 1 | 1 | rs6920364 | 6:167376466_G_C |
| PGP000226_Xie_2021 | Xie (2021) | rs11780471 | G | 8:27344719 | 1 | 1 | 1 | rs11780471 | 8:27344719_G_A |
| PGP000226_Xie_2021 | Xie (2021) | rs4236709 | G | 8:32410110 | 1 | 1 | 1 | rs4236709 | 8:32410110_G_A |
| PGP000226_Xie_2021 | Xie (2021) | rs885518 | G | 9:21830157 | 1 | 1 | 1 | rs885518 | 9:21830157_A_G |
| PGP000226_Xie_2021 | Xie (2021) | rs11591710 | C | 10:105687632 | 1 | 1 | 1 | rs11591710 | 10:105687632_A_C |
| PGP000226_Xie_2021 | Xie (2021) | rs1056562 | T | 11:118125625 | 1 | 1 | 1 | rs1056562 | 11:118125625_T_C |
| PGP000226_Xie_2021 | Xie (2021) | rs7953330 | G | 12:998819 | 1 | 1 | 1 | rs7953330 | 12:998819_G_C |
| PGP000226_Xie_2021 | Xie (2021) | rs11571833 | T | 13:32869864 | 1 | 1 | 1 | rs56084662 | 13:32869864_G_A |
| PGP000226_Xie_2021 | Xie (2021) | rs55781567 | G | 15:78857986 | 1 | 1 | 1 | rs55781567 | rs55781567 |
| PGP000226_Xie_2021 | Xie (2021) | rs77468143 | T | 15:49376624 | 1 | 1 | 1 | rs77468143 | 15:49376624_T_G |
| PGP000226_Xie_2021 | Xie (2021) | rs66759488 | A | 15:47577451 | 1 | 1 | 1 | rs66759488 | 15:47577451_G_A |
| PGP000226_Xie_2021 | Xie (2021) | rs56113850 | C | 19:41353107 | 1 | 1 | 1 | rs56113850 | 19:41353107_T_C |
| PGP000226_Xie_2021 | Xie (2021) | rs41309931 | T | 20:62326579 | 1 | 1 | 1 | rs41309931 | 20:62326579_G_T |
| PGP000226_Xie_2021 | Xie (2021) | rs17879961 | A | 22:29121087 | 1 | 1 | 1 | rs17879961 | rs17879961 |
| PGP000300_Zhang_2022 | P. Zhang (2022) | rs71658797 | A | 1:77967507 | 1 | 1 | 1 | rs71658797 | 1:77967507_T_A |
| PGP000300_Zhang_2022 | P. Zhang (2022) | rs17038564 | G | 2:65496058 | 1 | 1 | 1 | rs17038564 | 2:65496058_A_G |
| PGP000300_Zhang_2022 | P. Zhang (2022) | rs3769821 | C | 2:202123430 | 1 | 1 | 1 | rs3769821 | 2:202123430_C_T |
| PGP000300_Zhang_2022 | P. Zhang (2022) | rs2293607 | T | 3:169482335 | 1 | 1 | 1 | rs2293607 | 3:169482335_T_C |
| PGP000300_Zhang_2022 | P. Zhang (2022) | rs13314271 | T | 3:189357602 | 1 | 1 | 1 | rs13314271 | 3:189357602_T_C |
| PGP000300_Zhang_2022 | P. Zhang (2022) | rs13167280 | A | 5:1280477 | 1 | 1 | 1 | rs13167280 | 5:1280477_G_A |
| PGP000300_Zhang_2022 | P. Zhang (2022) | rs7705526 | A | 5:1285974 | 1 | 1 | 1 | rs7705526 | 5:1285974_C_A |
| PGP000300_Zhang_2022 | P. Zhang (2022) | rs4975616 | G | 5:1315660 | 1 | 1 | 1 | rs4975616 | rs4975616 |
| PGP000300_Zhang_2022 | P. Zhang (2022) | rs1056503 | G | 5:82648977 | 1 | 1 | 1 | rs1056503 | 5:82648977_T_G |
| PGP000300_Zhang_2022 | P. Zhang (2022) | rs2895680 | C | 5:146644115 | 1 | 1 | 1 | rs2895680 | 5:146644115_C_T |
| PGP000300_Zhang_2022 | P. Zhang (2022) | rs2517873 | A | 6:29875992 | 1 | 1 | 1 | rs2517873 | 6:29875992_G_A |
| PGP000300_Zhang_2022 | P. Zhang (2022) | rs3817963 | C | 6:32368087 | 1 | 1 | 1 | rs3817963 | rs3817963 |
| PGP000300_Zhang_2022 | P. Zhang (2022) | rs1853837 | A | 6:41497035 | 1 | 1 | 1 | rs1853837 | 6:41497035_C_A |
| PGP000300_Zhang_2022 | P. Zhang (2022) | rs5879422 | T | 6:117784658 | 1 | 1 | 1 | rs5879422 | 6:117784658_T_TTG |
| PGP000300_Zhang_2022 | P. Zhang (2022) | rs6920364 | C | 6:167376466 | 1 | 1 | 1 | rs6920364 | 6:167376466_G_C |
| PGP000300_Zhang_2022 | P. Zhang (2022) | rs11780471 | G | 8:27344719 | 1 | 1 | 1 | rs11780471 | 8:27344719_G_A |
| PGP000300_Zhang_2022 | P. Zhang (2022) | rs4236709 | G | 8:32410110 | 1 | 1 | 1 | rs4236709 | 8:32410110_G_A |
| PGP000300_Zhang_2022 | P. Zhang (2022) | rs10429489 | A | 9:21787521 | 1 | 1 | 1 | rs10429489 | 9:21787521_G_A |
| PGP000300_Zhang_2022 | P. Zhang (2022) | rs62560775 | G | 9:22052068 | 1 | 1 | 1 | rs62560775 | 9:22052068_A_G |
| PGP000300_Zhang_2022 | P. Zhang (2022) | rs1333040 | C | 9:22083404 | 1 | 1 | 1 | rs1333040 | rs1333040 |
| PGP000300_Zhang_2022 | P. Zhang (2022) | rs4573350 | T | 9:124955115 | 1 | 1 | 1 | rs4573350 | 9:124955115_T_C |
| PGP000300_Zhang_2022 | P. Zhang (2022) | rs1663689 | T | 10:9025195 | 1 | 1 | 1 | rs1663689 | 10:9025195_T_C |
| PGP000300_Zhang_2022 | P. Zhang (2022) | rs12415204 | A | 10:95330890 | 1 | 1 | 1 | rs12415204 | 10:95330890_C_A |
| PGP000300_Zhang_2022 | P. Zhang (2022) | rs11591710 | C | 10:105687632 | 1 | 1 | 1 | rs11591710 | 10:105687632_A_C |
| PGP000300_Zhang_2022 | P. Zhang (2022) | rs12265047 | G | 10:114487925 | 1 | 1 | 1 | rs12265047 | 10:114487925_G_A |
| PGP000300_Zhang_2022 | P. Zhang (2022) | rs55768116 | C | 11:118108331 | 1 | 1 | 1 | rs55768116 | 11:118108331_C_A |
| PGP000300_Zhang_2022 | P. Zhang (2022) | rs7953330 | G | 12:998819 | 1 | 1 | 1 | rs7953330 | 12:998819_G_C |
| PGP000300_Zhang_2022 | P. Zhang (2022) | rs11571833 | T | 13:32972626 | 1 | 1 | 1 | rs11571833 | rs11571833 |
| PGP000300_Zhang_2022 | P. Zhang (2022) | rs1200399 | C | 14:35293185 | 1 | 1 | 1 | rs1200399 | rs1200399 |
| PGP000300_Zhang_2022 | P. Zhang (2022) | rs66759488 | A | 15:47577451 | 1 | 1 | 1 | rs66759488 | 15:47577451_G_A |
| PGP000300_Zhang_2022 | P. Zhang (2022) | rs77468143 | T | 15:49376624 | 1 | 1 | 1 | rs77468143 | 15:49376624_T_G |
| PGP000300_Zhang_2022 | P. Zhang (2022) | rs8034191 | C | 15:78806023 | 1 | 1 | 1 | rs8034191 | rs8034191 |
| PGP000300_Zhang_2022 | P. Zhang (2022) | rs56113850 | C | 19:41353107 | 1 | 1 | 1 | rs56113850 | 19:41353107_T_C |
| PGP000387_Qin_2022 | Qin (2022) | rs71658797 | A | NA:NA | 1 | 0 | 1 | NA | NA |
| PGP000387_Qin_2022 | Qin (2022) | rs3769821 | C | NA:NA | 1 | 0 | 1 | NA | NA |
| PGP000387_Qin_2022 | Qin (2022) | rs13080835 | G | NA:NA | 1 | 0 | 1 | NA | NA |
| PGP000387_Qin_2022 | Qin (2022) | rs13167280 | A | NA:NA | 1 | 0 | 1 | NA | NA |
| PGP000387_Qin_2022 | Qin (2022) | rs2853677 | G | NA:NA | 1 | 0 | 1 | NA | NA |
| PGP000387_Qin_2022 | Qin (2022) | rs465498 | A | NA:NA | 1 | 0 | 1 | NA | NA |
| PGP000387_Qin_2022 | Qin (2022) | rs2517873 | A | NA:NA | 1 | 0 | 1 | NA | NA |
| PGP000387_Qin_2022 | Qin (2022) | rs3094604 | G | NA:NA | 1 | 0 | 1 | NA | NA |
| PGP000387_Qin_2022 | Qin (2022) | rs6920364 | C | NA:NA | 1 | 0 | 1 | NA | NA |
| PGP000387_Qin_2022 | Qin (2022) | rs4236709 | G | NA:NA | 1 | 0 | 1 | NA | NA |
| PGP000387_Qin_2022 | Qin (2022) | rs885518 | G | NA:NA | 1 | 0 | 1 | NA | NA |
| PGP000387_Qin_2022 | Qin (2022) | rs1333040 | C | NA:NA | 1 | 0 | 1 | NA | NA |
| PGP000387_Qin_2022 | Qin (2022) | rs11591710 | C | NA:NA | 1 | 0 | 1 | NA | NA |
| PGP000387_Qin_2022 | Qin (2022) | rs1056562 | T | NA:NA | 1 | 0 | 1 | NA | NA |

S9 List of Included SNPs

| PGS_name | Label | rsID | Effect allele | Position | rsID in UKB extract | position in UKB extract | found in UKB extract | rsID in UKB | Position in UKB |
|---|---|---|---|---|---|---|---|---|---|
| PGP000387_Qin_2022 | Qin (2022) | rs6489769 | T | NA:NA | 1 | 0 | 1 | NA | NA |
| PGP000387_Qin_2022 | Qin (2022) | rs11571833 | T | NA:NA | 1 | 0 | 1 | NA | NA |
| PGP000387_Qin_2022 | Qin (2022) | rs66759488 | A | NA:NA | 1 | 0 | 1 | NA | NA |
| PGP000387_Qin_2022 | Qin (2022) | rs77468143 | T | NA:NA | 1 | 0 | 1 | NA | NA |
| PGP000387_Qin_2022 | Qin (2022) | rs41309931 | T | NA:NA | 1 | 0 | 1 | NA | NA |
| PGP000387_Qin_2022 | Qin (2022) | rs17879961 | A | NA:NA | 1 | 0 | 1 | NA | NA |
| PGP000518a_Wei_2023 | Wei (2023a) | rs11917154 | T | 3:193058019 | 1 | 1 | 1 | rs11917154 | 3:193058019_T_G |
| PGP000518a_Wei_2023 | Wei (2023a) | rs11928222 | G | 3:189350265 | 1 | 1 | 1 | rs11928222 | 3:189350265_T_G |
| PGP000518a_Wei_2023 | Wei (2023a) | rs1498606 | C | 5:58337549 | 1 | 1 | 1 | rs1498606 | 5:58337549_C_T |
| PGP000518a_Wei_2023 | Wei (2023a) | rs201857317 | T | 5:1307632 | 1 | 1 | 1 | rs201857317 | 5:1307632_G_T |
| PGP000518a_Wei_2023 | Wei (2023a) | rs7726159 | A | 5:1282319 | 1 | 1 | 1 | rs7726159 | rs7726159 |
| PGP000518a_Wei_2023 | Wei (2023a) | rs184888497 | G | 6:32599757 | 1 | 1 | 1 | rs184888497 | 6:32599757_G_A |
| PGP000518a_Wei_2023 | Wei (2023a) | rs2496646 | T | 6:41483367 | 1 | 1 | 1 | rs2496646 | 6:41483367_T_C |
| PGP000518a_Wei_2023 | Wei (2023a) | rs3207985 | C | 6:32609227 | 1 | 1 | 1 | rs3207985 | 6:32609227_A_C |
| PGP000518a_Wei_2023 | Wei (2023a) | rs78098131 | T | 6:32492238 | 1 | 1 | 1 | rs78098131 | 6:32492238_C_T |
| PGP000518a_Wei_2023 | Wei (2023a) | rs9275164 | C | 6:32652929 | 1 | 1 | 1 | rs9275164 | 6:32652929_T_C |
| PGP000518a_Wei_2023 | Wei (2023a) | rs9374663 | A | 6:117782634 | 1 | 1 | 1 | rs9374663 | 6:117782634_A_G |
| PGP000518a_Wei_2023 | Wei (2023a) | rs11196063 | C | 10:114460013 | 1 | 1 | 1 | rs11196063 | 10:114460013_A_C |
| PGP000518a_Wei_2023 | Wei (2023a) | rs12275876 | T | 11:118575254 | 1 | 1 | 1 | rs12275876 | 11:118575254_A_T |
| PGP000518a_Wei_2023 | Wei (2023a) | rs55768116 | C | 11:118108331 | 1 | 1 | 1 | rs55768116 | 11:118108331_C_A |
| PGP000518a_Wei_2023 | Wei (2023a) | rs7962469 | G | 12:52348259 | 1 | 1 | 1 | rs7962469 | 12:52348259_A_G |
| PGP000518a_Wei_2023 | Wei (2023a) | rs72845278 | T | 17:65877076 | 1 | 1 | 1 | rs72845278 | 17:65877076_C_T |
| PGP000518b_Wei_2023 | Wei (2023b) | rs2293607 | T | 3:169482335 | 1 | 1 | 1 | rs2293607 | 3:169482335_T_C |
| PGP000518b_Wei_2023 | Wei (2023b) | rs11928222 | G | 3:189350265 | 1 | 1 | 1 | rs11928222 | 3:189350265_T_G |
| PGP000518b_Wei_2023 | Wei (2023b) | rs11917154 | T | 3:193058019 | 1 | 1 | 1 | rs11917154 | 3:193058019_T_G |
| PGP000518b_Wei_2023 | Wei (2023b) | rs7726159 | A | 5:1282319 | 1 | 1 | 1 | rs7726159 | rs7726159 |
| PGP000518b_Wei_2023 | Wei (2023b) | rs201857317 | T | 5:1307632 | 1 | 1 | 1 | rs201857317 | 5:1307632_G_T |
| PGP000518b_Wei_2023 | Wei (2023b) | rs1498606 | C | 5:58337549 | 1 | 1 | 1 | rs1498606 | 5:58337549_C_T |
| PGP000518b_Wei_2023 | Wei (2023b) | rs78098131 | T | 6:32492238 | 1 | 1 | 1 | rs78098131 | 6:32492238_C_T |
| PGP000518b_Wei_2023 | Wei (2023b) | rs184888497 | G | 6:32599757 | 1 | 1 | 1 | rs184888497 | 6:32599757_G_A |
| PGP000518b_Wei_2023 | Wei (2023b) | rs3207985 | C | 6:32609227 | 1 | 1 | 1 | rs3207985 | 6:32609227_A_C |
| PGP000518b_Wei_2023 | Wei (2023b) | rs9275164 | C | 6:32652929 | 1 | 1 | 1 | rs9275164 | 6:32652929_T_C |
| PGP000518b_Wei_2023 | Wei (2023b) | rs2496646 | T | 6:41483367 | 1 | 1 | 1 | rs2496646 | 6:41483367_T_C |
| PGP000518b_Wei_2023 | Wei (2023b) | rs9374663 | A | 6:117782634 | 1 | 1 | 1 | rs9374663 | 6:117782634_A_G |
| PGP000518b_Wei_2023 | Wei (2023b) | rs4236709 | G | 8:32410110 | 1 | 1 | 1 | rs4236709 | 8:32410110_G_A |
| PGP000518b_Wei_2023 | Wei (2023b) | rs10429489 | A | 9:21787521 | 1 | 1 | 1 | rs10429489 | 9:21787521_G_A |
| PGP000518b_Wei_2023 | Wei (2023b) | rs11196063 | C | 10:114460013 | 1 | 1 | 1 | rs11196063 | 10:114460013_A_C |
| PGP000518b_Wei_2023 | Wei (2023b) | rs12275876 | T | 11:118575254 | 1 | 1 | 1 | rs12275876 | 11:118575254_A_T |
| PGP000518b_Wei_2023 | Wei (2023b) | rs55768116 | C | 11:118108331 | 1 | 1 | 1 | rs55768116 | 11:118108331_C_A |
| PGP000518b_Wei_2023 | Wei (2023b) | rs7962469 | G | 12:52348259 | 1 | 1 | 1 | rs7962469 | 12:52348259_A_G |
| PGP000518b_Wei_2023 | Wei (2023b) | rs1200399 | C | 14:35293185 | 1 | 1 | 1 | rs1200399 | rs1200399 |
| PGP000518b_Wei_2023 | Wei (2023b) | rs77468143 | T | 15:49376624 | 1 | 1 | 1 | rs77468143 | 15:49376624_T_G |
| PGP000518b_Wei_2023 | Wei (2023b) | rs72845278 | T | 17:65877076 | 1 | 1 | 1 | rs72845278 | 17:65877076_C_T |
| PGP000542_Kim_2023 | Kim (2023) | rs71658797 | A | 1:77967507 | 1 | 1 | 1 | rs71658797 | 1:77967507_T_A |
| PGP000542_Kim_2023 | Kim (2023) | rs112401627 | A | 5:1300269 | 1 | 1 | 1 | rs112401627 | 5:1300269_G_A |
| PGP000542_Kim_2023 | Kim (2023) | rs2735947 | A | 5:1299392 | 1 | 1 | 1 | rs2735947 | 5:1299392_G_A |
| PGP000542_Kim_2023 | Kim (2023) | rs2853668 | T | 5:1300025 | 1 | 1 | 1 | rs2853668 | 5:1300025_G_T |
| PGP000542_Kim_2023 | Kim (2023) | rs380286 | A | 5:1320247 | 1 | 1 | 1 | rs380286 | 5:1320247_G_A |
| PGP000542_Kim_2023 | Kim (2023) | rs40183 | A | 5:1348352 | 1 | 1 | 1 | rs40183 | 5:1348352_G_A |
| PGP000542_Kim_2023 | Kim (2023) | rs56345976 | G | 5:1276873 | 1 | 1 | 1 | rs56345976 | 5:1276873_G_A |
| PGP000542_Kim_2023 | Kim (2023) | rs7705526 | A | 5:1285974 | 1 | 1 | 1 | rs7705526 | 5:1285974_C_A |
| PGP000542_Kim_2023 | Kim (2023) | rs116629156 | C | 6:30864829 | 0 | 1 | 1 | rs1049623 | rs1049623 |
| PGP000542_Kim_2023 | Kim (2023) | rs116080888 | A | 6:32586236 | 0 | 1 | 1 | rs11751024 | 6:32586236_C_A |
| PGP000542_Kim_2023 | Kim (2023) | rs115915654 | T | 6:30769726 | 0 | 1 | 1 | rs1264372 | rs1264372 |
| PGP000542_Kim_2023 | Kim (2023) | rs13191445 | A | 6:26015489 | 1 | 1 | 1 | rs13191445 | 6:26015489_G_A |
| PGP000542_Kim_2023 | Kim (2023) | rs149442300 | T | 6:29791256 | 0 | 1 | 1 | rs1610679 | 6:29791256_C_T |
| PGP000542_Kim_2023 | Kim (2023) | rs74942078 | T | 6:32605884 | 0 | 1 | 1 | rs2187668 | rs2187668 |
| PGP000542_Kim_2023 | Kim (2023) | rs239935 | G | 6:167411788 | 1 | 1 | 1 | rs239935 | 6:167411788_G_A |
| PGP000542_Kim_2023 | Kim (2023) | rs116822326 | G | 6:31434111 | 0 | 1 | 1 | rs3094604 | 6:31434111_A_G |
| PGP000542_Kim_2023 | Kim (2023) | rs3130975 | T | 6:31081838 | 1 | 1 | 1 | rs3130975 | rs3130975 |
| PGP000542_Kim_2023 | Kim (2023) | rs114279165 | G | 6:31425499 | 0 | 1 | 1 | rs3131621 | rs3131621 |
| PGP000542_Kim_2023 | Kim (2023) | rs115870917 | C | 6:29607101 | 0 | 1 | 1 | rs3131856 | 6:29607101_T_C |
| PGP000542_Kim_2023 | Kim (2023) | rs34661125 | A | 6:28281894 | 1 | 1 | 1 | rs34661125 | 6:28281894_G_A |
| PGP000542_Kim_2023 | Kim (2023) | rs115444303 | G | 6:29942983 | 0 | 1 | 1 | rs378971 | 6:29942983_G_A |
| PGP000542_Kim_2023 | Kim (2023) | rs114103504 | G | 6:31002452 | 0 | 1 | 1 | rs3869098 | 6:31002452_A_G |

## S9 List of Included SNPs

| PGS_name | Label | rsID | Effect allele | Position | rsID in UKB extract | position in UKB extract | found in UKB extract | rsID in UKB | Position in UKB |
|---|---|---|---|---|---|---|---|---|---|
| PGP000542_Kim_2023 | Kim (2023) | rs3999544 | C | 6:26285867 | 1 | 1 | 1 | rs3999544 | 6:26285867_C_T |
| PGP000542_Kim_2023 | Kim (2023) | rs56114371 | T | 6:27274834 | 1 | 1 | 1 | rs56114371 | 6:27274834_C_T |
| PGP000542_Kim_2023 | Kim (2023) | rs6912292 | C | 6:26511805 | 1 | 1 | 1 | rs6912292 | 6:26511805_C_A |
| PGP000542_Kim_2023 | Kim (2023) | rs116383790 | T | 6:31628733 | 0 | 1 | 1 | rs805262 | rs805262 |
| PGP000542_Kim_2023 | Kim (2023) | rs114229481 | A | 6:32743640 | 0 | 1 | 1 | rs9276623 | 6:32743640_G_A |
| PGP000542_Kim_2023 | Kim (2023) | rs11780471 | A | 8:27344719 | 1 | 1 | 1 | rs11780471 | 8:27344719_G_A |
| PGP000542_Kim_2023 | Kim (2023) | rs1629083 | C | 11:118126576 | 1 | 1 | 1 | rs1629083 | 11:118126576_C_T |
| PGP000542_Kim_2023 | Kim (2023) | rs7953330 | C | 12:998819 | 1 | 1 | 1 | rs7953330 | 12:998819_G_C |
| PGP000542_Kim_2023 | Kim (2023) | rs11571833 | T | 13:32972626 | 1 | 1 | 1 | rs11571833 | rs11571833 |
| PGP000542_Kim_2023 | Kim (2023) | rs117189732 | T | 15:79083280 | 1 | 1 | 1 | rs117189732 | rs117189732 |
| PGP000542_Kim_2023 | Kim (2023) | rs12443170 | A | 15:78907736 | 1 | 1 | 1 | rs12443170 | 15:78907736_G_A |
| PGP000542_Kim_2023 | Kim (2023) | rs151118057 | A | 15:78988620 | 1 | 1 | 1 | rs151118057 | 15:78988620_G_A |
| PGP000542_Kim_2023 | Kim (2023) | rs2229961 | A | 15:78880752 | 1 | 1 | 1 | rs2229961 | rs2229961 |
| PGP000542_Kim_2023 | Kim (2023) | rs28580532 | T | 15:79134724 | 1 | 1 | 1 | rs28580532 | 15:79134724_C_T |
| PGP000542_Kim_2023 | Kim (2023) | rs55781567 | G | 15:78857986 | 1 | 1 | 1 | rs55781567 | rs55781567 |
| PGP000542_Kim_2023 | Kim (2023) | rs66759488 | A | 15:47577451 | 1 | 1 | 1 | rs66759488 | 15:47577451_G_A |
| PGP000542_Kim_2023 | Kim (2023) | rs7177201 | T | 15:79065380 | 1 | 1 | 1 | rs7177201 | 15:79065380_T_C |
| PGP000542_Kim_2023 | Kim (2023) | rs76474922 | C | 15:78884553 | 1 | 1 | 1 | rs76474922 | 15:78884553_A_C |
| PGP000542_Kim_2023 | Kim (2023) | rs77468143 | G | 15:49376624 | 1 | 1 | 1 | rs77468143 | 15:49376624_T_G |
| PGP000542_Kim_2023 | Kim (2023) | rs8192479 | T | 15:78909398 | 1 | 1 | 1 | rs8192479 | rs8192479 |
| PGP000542_Kim_2023 | Kim (2023) | rs56113850 | T | 19:41353107 | 1 | 1 | 1 | rs56113850 | 19:41353107_T_C |
| PGP000559_Wang_2023 | Wang (2023) | rs71658797 | A | 1:77967507 | 1 | 1 | 1 | rs71658797 | 1:77967507_T_A |
| PGP000559_Wang_2023 | Wang (2023) | rs13080835 | G | 3:189357199 | 1 | 1 | 1 | rs13080835 | 3:189357199_G_T |
| PGP000559_Wang_2023 | Wang (2023) | rs7705526 | A | 5:1285974 | 1 | 1 | 1 | rs7705526 | 5:1285974_C_A |
| PGP000559_Wang_2023 | Wang (2023) | rs2853677 | G | 5:1287194 | 1 | 1 | 1 | rs2853677 | rs2853677 |
| PGP000559_Wang_2023 | Wang (2023) | rs465498 | A | 5:1325803 | 1 | 1 | 1 | rs465498 | 5:1325803_A_G |
| PGP000559_Wang_2023 | Wang (2023) | rs3115672 | T | 6:31727897 | 1 | 1 | 1 | rs3115672 | 6:31727897_C_T |
| PGP000559_Wang_2023 | Wang (2023) | rs6920364 | C | 6:167376466 | 1 | 1 | 1 | rs6920364 | 6:167376466_G_C |
| PGP000559_Wang_2023 | Wang (2023) | rs4236709 | G | 8:32410110 | 1 | 1 | 1 | rs4236709 | 8:32410110_G_A |
| PGP000559_Wang_2023 | Wang (2023) | rs11780471 | G | 8:27344719 | 1 | 1 | 1 | rs11780471 | 8:27344719_G_A |
| PGP000559_Wang_2023 | Wang (2023) | rs885518 | G | 9:21830157 | 1 | 1 | 1 | rs885518 | 9:21830157_A_G |
| PGP000559_Wang_2023 | Wang (2023) | rs62560775 | G | 9:22052068 | 1 | 1 | 1 | rs62560775 | 9:22052068_A_G |
| PGP000559_Wang_2023 | Wang (2023) | rs1056562 | T | 11:118125625 | 1 | 1 | 1 | rs1056562 | 11:118125625_T_C |
| PGP000559_Wang_2023 | Wang (2023) | rs7953330 | G | 12:998819 | 1 | 1 | 1 | rs7953330 | 12:998819_G_C |
| PGP000559_Wang_2023 | Wang (2023) | rs11571833 | T | 13:32972626 | 1 | 1 | 1 | rs11571833 | rs11571833 |
| PGP000559_Wang_2023 | Wang (2023) | rs66759488 | A | 15:47577451 | 1 | 1 | 1 | rs66759488 | 15:47577451_G_A |
| PGP000559_Wang_2023 | Wang (2023) | rs77468143 | T | 15:49376624 | 1 | 1 | 1 | rs77468143 | 15:49376624_T_G |
| PGP000559_Wang_2023 | Wang (2023) | rs55781567 | G | 15:78857986 | 1 | 1 | 1 | rs55781567 | rs55781567 |
| PGP000559_Wang_2023 | Wang (2023) | rs8042374 | A | 15:78908032 | 1 | 1 | 1 | rs8042374 | 15:78908032_A_G |
| PGP000559_Wang_2023 | Wang (2023) | rs56113850 | C | 19:41353107 | 1 | 1 | 1 | rs56113850 | 19:41353107_T_C |
| barnett_2022_study_12 | Barnett (2022) | rs8042374 | G | 15:78615690 | 1 | 0 | 1 | NA | NA |
| barnett_2022_study_12 | Barnett (2022) | rs3117582 | C | 6:31652743 | 0 | 0 | 0 | NA | NA |
| barnett_2022_study_12 | Barnett (2022) | rs31489 | A | 5:1395714 | 0 | 0 | 0 | NA | NA |
| barnett_2022_study_12 | Barnett (2022) | rs4635969 | T | 5:1361552 | 1 | 0 | 1 | NA | NA |
| barnett_2022_study_12 | Barnett (2022) | rs1996371 | C | 15:76743861 | 0 | 0 | 0 | NA | NA |
| barnett_2022_study_12 | Barnett (2022) | rs1056562 | T | 11:118125625 | 1 | 1 | 1 | rs1056562 | 11:118125625_T_C |
| barnett_2022_study_12 | Barnett (2022) | rs11571833 | T | 13:32972626 | 1 | 1 | 1 | rs11571833 | rs11571833 |
| barnett_2022_study_12 | Barnett (2022) | rs11591710 | C | 10:105687632 | 1 | 1 | 1 | rs11591710 | 10:105687632_A_C |
| barnett_2022_study_12 | Barnett (2022) | rs116822326 | G | 6:31434111 | 0 | 1 | 1 | rs3094604 | 6:31434111_A_G |
| barnett_2022_study_12 | Barnett (2022) | rs11780471 | A | 8:27344719 | 1 | 1 | 1 | rs11780471 | 8:27344719_G_A |
| barnett_2022_study_12 | Barnett (2022) | rs13080835 | T | 3:189357199 | 1 | 1 | 1 | rs13080835 | 3:189357199_G_T |
| barnett_2022_study_12 | Barnett (2022) | rs17879961 | G | 22:29121087 | 1 | 1 | 1 | rs17879961 | rs17879961 |
| barnett_2022_study_12 | Barnett (2022) | rs41309931 | T | 20:62326579 | 1 | 1 | 1 | rs41309931 | 20:62326579_G_T |
| barnett_2022_study_12 | Barnett (2022) | rs4236709 | G | 8:32410110 | 1 | 1 | 1 | rs4236709 | 8:32410110_G_A |
| barnett_2022_study_12 | Barnett (2022) | rs55781567 | G | 15:78857986 | 1 | 1 | 1 | rs55781567 | rs55781567 |
| barnett_2022_study_12 | Barnett (2022) | rs56113850 | T | 19:41353107 | 1 | 1 | 1 | rs56113850 | 19:41353107_T_C |
| barnett_2022_study_12 | Barnett (2022) | rs66759488 | A | 15:47577451 | 1 | 1 | 1 | rs66759488 | 15:47577451_G_A |
| barnett_2022_study_12 | Barnett (2022) | rs6920364 | C | 6:167376466 | 1 | 1 | 1 | rs6920364 | 6:167376466_G_C |
| barnett_2022_study_12 | Barnett (2022) | rs71658797 | A | 1:77967507 | 1 | 1 | 1 | rs71658797 | 1:77967507_T_A |
| barnett_2022_study_12 | Barnett (2022) | rs7705526 | A | 5:1285974 | 1 | 1 | 1 | rs7705526 | 5:1285974_C_A |
| barnett_2022_study_12 | Barnett (2022) | rs77468143 | G | 15:49376624 | 1 | 1 | 1 | rs77468143 | 15:49376624_T_G |
| barnett_2022_study_12 | Barnett (2022) | rs7953330 | C | 12:998819 | 1 | 1 | 1 | rs7953330 | 12:998819_G_C |
| barnett_2022_study_12 | Barnett (2022) | rs885518 | G | 9:21830157 | 1 | 1 | 1 | rs885518 | 9:21830157_A_G |
| barnett_2022_study_12 | Barnett (2022) | rs4324798 | A | 6:28808340 | 0 | 1 | 1 | rs371006603 | 6:28808340_A_T |

## S9 List of Included SNPs

| PGS_name | Label | rsID | Effect allele | Position | rsID in UKB extract | position in UKB extract | found in UKB extract | rsID in UKB | Position in UKB |
|---|---|---|---|---|---|---|---|---|---|
| barnett_2022_study_12 | Barnett (2022) | rs1333040 | C | 9:22083405 | 1 | 0 | 1 | NA | NA |
| blechter_2023_study_41 | Blechter (2023) | rs10901793 | A | 10:126324209 | 1 | 1 | 1 | rs10901793 | 10:126324209_G_A |
| blechter_2023_study_41 | Blechter (2023) | rs11196089 | C | 10:114509290 | 1 | 1 | 1 | rs11196089 | rs11196089 |
| blechter_2023_study_41 | Blechter (2023) | rs116863980 | A | 19:725066 | 1 | 1 | 1 | rs116863980 | 19:725066_G_A |
| blechter_2023_study_41 | Blechter (2023) | rs117715768 | T | 4:44174404 | 1 | 1 | 1 | rs117715768 | 4:44174404_C_T |
| blechter_2023_study_41 | Blechter (2023) | rs1200399 | T | 14:35293185 | 1 | 1 | 1 | rs1200399 | rs1200399 |
| blechter_2023_study_41 | Blechter (2023) | rs1373058 | A | 4:157894892 | 1 | 1 | 1 | rs1373058 | 4:157894892_T_A |
| blechter_2023_study_41 | Blechter (2023) | rs137884934 | T | 3:138570011 | 1 | 1 | 1 | rs137884934 | 3:138570011_C_T |
| blechter_2023_study_41 | Blechter (2023) | rs17038564 | G | 2:65496058 | 1 | 1 | 1 | rs17038564 | 2:65496058_A_G |
| blechter_2023_study_41 | Blechter (2023) | rs174559 | G | 11:61581656 | 1 | 1 | 1 | rs174559 | 11:61581656_G_A |
| blechter_2023_study_41 | Blechter (2023) | rs2293607 | T | 3:169482335 | 1 | 1 | 1 | rs2293607 | 3:169482335_T_C |
| blechter_2023_study_41 | Blechter (2023) | rs2736100 | C | 5:1286516 | 1 | 1 | 1 | rs2736100 | rs2736100 |
| blechter_2023_study_41 | Blechter (2023) | rs2760995 | A | 6:32574358 | 1 | 1 | 1 | rs2760995 | 6:32574358_G_A |
| blechter_2023_study_41 | Blechter (2023) | rs4268071 | T | 7:124373384 | 1 | 1 | 1 | rs4268071 | 7:124373384_T_G |
| blechter_2023_study_41 | Blechter (2023) | rs531557 | A | 6:53389995 | 1 | 1 | 1 | rs531557 | 6:53389995_A_T |
| blechter_2023_study_41 | Blechter (2023) | rs55768116 | A | 11:118108331 | 1 | 1 | 1 | rs55768116 | 11:118108331_C_A |
| blechter_2023_study_41 | Blechter (2023) | rs55779747 | C | 3:189354127 | 1 | 1 | 1 | rs55779747 | 3:189354127_A_C |
| blechter_2023_study_41 | Blechter (2023) | rs59956089 | C | 17:65960854 | 1 | 1 | 1 | rs59956089 | 17:65960854_T_C |
| blechter_2023_study_41 | Blechter (2023) | rs682888 | T | 2:25757709 | 1 | 1 | 1 | rs682888 | 2:25757709_T_C |
| blechter_2023_study_41 | Blechter (2023) | rs6937083 | T | 6:117785308 | 1 | 1 | 1 | rs6937083 | 6:117785308_A_T |
| blechter_2023_study_41 | Blechter (2023) | rs71467682 | G | 15:49757466 | 1 | 1 | 1 | rs71467682 | 15:49757466_A_G |
| blechter_2023_study_41 | Blechter (2023) | rs72658409 | T | 9:22160087 | 1 | 1 | 1 | rs72658409 | rs72658409 |
| blechter_2023_study_41 | Blechter (2023) | rs764014 | G | 15:56454223 | 1 | 1 | 1 | rs764014 | 15:56454223_A_G |
| blechter_2023_study_41 | Blechter (2023) | rs7962469 | G | 12:52348259 | 1 | 1 | 1 | rs7962469 | 12:52348259_A_G |
| blechter_2023_study_41 | Blechter (2023) | rs9367106 | C | 6:41483390 | 1 | 1 | 1 | rs9367106 | 6:41483390_G_C |
| blechter_2023_study_41 | Blechter (2023) | rs9380190 | C | 6:30769565 | 1 | 1 | 1 | rs9380190 | 6:30769565_T_C |
| choi_2022_study_32 | J. Choi (2022c) | rs71658797 | A | 1:NA | 1 | 0 | 1 | NA | NA |
| choi_2022_study_32 | J. Choi (2022c) | rs13080835 | G | 3:NA | 1 | 0 | 1 | NA | NA |
| choi_2022_study_32 | J. Choi (2022c) | rs7705526 | A | 5:NA | 1 | 0 | 1 | NA | NA |
| choi_2022_study_32 | J. Choi (2022c) | rs2853677 | G | 5:NA | 1 | 0 | 1 | NA | NA |
| choi_2022_study_32 | J. Choi (2022c) | rs465498 | A | 5:NA | 1 | 0 | 1 | NA | NA |
| choi_2022_study_32 | J. Choi (2022c) | rs3115672 | T | 6:NA | 1 | 0 | 1 | NA | NA |
| choi_2022_study_32 | J. Choi (2022c) | rs6920364 | C | 6:NA | 1 | 0 | 1 | NA | NA |
| choi_2022_study_32 | J. Choi (2022c) | rs4236709 | G | 8:NA | 1 | 0 | 1 | NA | NA |
| choi_2022_study_32 | J. Choi (2022c) | rs11780471 | G | 8:NA | 1 | 0 | 1 | NA | NA |
| choi_2022_study_32 | J. Choi (2022c) | rs885518 | G | 9:NA | 1 | 0 | 1 | NA | NA |
| choi_2022_study_32 | J. Choi (2022c) | rs62560775 | G | 9:NA | 1 | 0 | 1 | NA | NA |
| choi_2022_study_32 | J. Choi (2022c) | rs1056562 | T | 11:NA | 1 | 0 | 1 | NA | NA |
| choi_2022_study_32 | J. Choi (2022c) | rs7953330 | G | 12:NA | 1 | 0 | 1 | NA | NA |
| choi_2022_study_32 | J. Choi (2022c) | rs11571833 | T | 13:NA | 1 | 0 | 1 | NA | NA |
| choi_2022_study_32 | J. Choi (2022c) | rs66759488 | A | 15:NA | 1 | 0 | 1 | NA | NA |
| choi_2022_study_32 | J. Choi (2022c) | rs77468143 | T | 15:NA | 1 | 0 | 1 | NA | NA |
| choi_2022_study_32 | J. Choi (2022c) | rs55781567 | G | 15:NA | 1 | 0 | 1 | NA | NA |
| choi_2022_study_32 | J. Choi (2022c) | rs8042374 | A | 15:NA | 1 | 0 | 1 | NA | NA |
| choi_2022_study_32 | J. Choi (2022c) | rs56113850 | C | 19:NA | 1 | 0 | 1 | NA | NA |
| choi_2022_study_43_A | J. Choi (2022a) | rs71658797 | A | 1:77967507 | 1 | 1 | 1 | rs71658797 | 1:77967507_T_A |
| choi_2022_study_43_A | J. Choi (2022a) | rs13080835 | G | 3:189357199 | 1 | 1 | 1 | rs13080835 | 3:189357199_G_T |
| choi_2022_study_43_A | J. Choi (2022a) | rs7705526 | A | 5:1285974 | 1 | 1 | 1 | rs7705526 | 5:1285974_C_A |
| choi_2022_study_43_A | J. Choi (2022a) | rs2853677 | G | 5:1287194 | 1 | 1 | 1 | rs2853677 | rs2853677 |
| choi_2022_study_43_A | J. Choi (2022a) | rs465498 | A | 5:1325803 | 1 | 1 | 1 | rs465498 | 5:1325803_A_G |
| choi_2022_study_43_A | J. Choi (2022a) | rs3115672 | T | 6:31727897 | 1 | 1 | 1 | rs3115672 | 6:31727897_C_T |
| choi_2022_study_43_A | J. Choi (2022a) | rs6920364 | C | 6:167376466 | 1 | 1 | 1 | rs6920364 | 6:167376466_G_C |
| choi_2022_study_43_A | J. Choi (2022a) | rs4236709 | G | 8:32410110 | 1 | 1 | 1 | rs4236709 | 8:32410110_G_A |
| choi_2022_study_43_A | J. Choi (2022a) | rs11780471 | G | 8:27344719 | 1 | 1 | 1 | rs11780471 | 8:27344719_G_A |
| choi_2022_study_43_A | J. Choi (2022a) | rs885518 | G | 9:21830157 | 1 | 1 | 1 | rs885518 | 9:21830157_A_G |
| choi_2022_study_43_A | J. Choi (2022a) | rs62560775 | G | 9:22052068 | 1 | 1 | 1 | rs62560775 | 9:22052068_A_G |
| choi_2022_study_43_A | J. Choi (2022a) | rs1056562 | T | 11:118125625 | 1 | 1 | 1 | rs1056562 | 11:118125625_T_C |
| choi_2022_study_43_A | J. Choi (2022a) | rs7953330 | G | 12:998819 | 1 | 1 | 1 | rs7953330 | 12:998819_G_C |
| choi_2022_study_43_A | J. Choi (2022a) | rs11571833 | T | 13:32972626 | 1 | 1 | 1 | rs11571833 | rs11571833 |
| choi_2022_study_43_A | J. Choi (2022a) | rs66759488 | A | 15:47577451 | 1 | 1 | 1 | rs66759488 | 15:47577451_G_A |
| choi_2022_study_43_A | J. Choi (2022a) | rs77468143 | T | 15:49376624 | 1 | 1 | 1 | rs77468143 | 15:49376624_T_G |
| choi_2022_study_43_A | J. Choi (2022a) | rs55781567 | G | 15:78857986 | 1 | 1 | 1 | rs55781567 | rs55781567 |
| choi_2022_study_43_A | J. Choi (2022a) | rs8042374 | A | 15:78908032 | 1 | 1 | 1 | rs8042374 | 15:78908032_A_G |
| choi_2022_study_43_A | J. Choi (2022a) | rs56113850 | C | 19:41353107 | 1 | 1 | 1 | rs56113850 | 19:41353107_T_C |
| choi_2022_study_43_B | J. Choi (2022b) | rs71658797 | A | 1:77967507 | 1 | 1 | 1 | rs71658797 | 1:77967507_T_A |

## S9 List of Included SNPs

| PGS_name | Label | rsID | Effect allele | Position | rsID in UKB extract | position in UKB extract | found in UKB extract | rsID in UKB | Position in UKB |
|---|---|---|---|---|---|---|---|---|---|
| choi_2022_study_43_B | J. Choi (2022b) | rs13080835 | G | 3:189357199 | 1 | 1 | 1 | rs13080835 | 3:189357199_G_T |
| choi_2022_study_43_B | J. Choi (2022b) | rs7705526 | A | 5:1285974 | 1 | 1 | 1 | rs7705526 | 5:1285974_C_A |
| choi_2022_study_43_B | J. Choi (2022b) | rs2853677 | G | 5:1287194 | 1 | 1 | 1 | rs2853677 | rs2853677 |
| choi_2022_study_43_B | J. Choi (2022b) | rs465498 | A | 5:1325803 | 1 | 1 | 1 | rs465498 | 5:1325803_A_G |
| choi_2022_study_43_B | J. Choi (2022b) | rs4236709 | G | 8:32410110 | 1 | 1 | 1 | rs4236709 | 8:32410110_G_A |
| choi_2022_study_43_B | J. Choi (2022b) | rs11780471 | G | 8:27344719 | 1 | 1 | 1 | rs11780471 | 8:27344719_G_A |
| choi_2022_study_43_B | J. Choi (2022b) | rs885518 | G | 9:21830157 | 1 | 1 | 1 | rs885518 | 9:21830157_A_G |
| choi_2022_study_43_B | J. Choi (2022b) | rs62560775 | G | 9:22052068 | 1 | 1 | 1 | rs62560775 | 9:22052068_A_G |
| choi_2022_study_43_B | J. Choi (2022b) | rs1056562 | T | 11:118125625 | 1 | 1 | 1 | rs1056562 | 11:118125625_T_C |
| choi_2022_study_43_B | J. Choi (2022b) | rs7953330 | G | 12:998819 | 1 | 1 | 1 | rs7953330 | 12:998819_G_C |
| choi_2022_study_43_B | J. Choi (2022b) | rs11571833 | T | 13:32972626 | 1 | 1 | 1 | rs11571833 | rs11571833 |
| choi_2022_study_43_B | J. Choi (2022b) | rs66759488 | A | 15:47577451 | 1 | 1 | 1 | rs66759488 | 15:47577451_G_A |
| choi_2022_study_43_B | J. Choi (2022b) | rs77468143 | T | 15:49376624 | 1 | 1 | 1 | rs77468143 | 15:49376624_T_G |
| choi_2022_study_43_B | J. Choi (2022b) | rs55781567 | G | 15:78857986 | 1 | 1 | 1 | rs55781567 | rs55781567 |
| choi_2022_study_43_B | J. Choi (2022b) | rs8042374 | A | 15:78908032 | 1 | 1 | 1 | rs8042374 | 15:78908032_A_G |
| choi_2022_study_43_B | J. Choi (2022b) | rs56113850 | C | 19:41353107 | 1 | 1 | 1 | rs56113850 | 19:41353107_T_C |
| huang_2021_study_17 | Huang (2021) | rs71658797 | T | 1:77967507 | 1 | 1 | 1 | rs71658797 | 1:77967507_T_A |
| huang_2021_study_17 | Huang (2021) | rs13080835 | T | 3:189357199 | 1 | 1 | 1 | rs13080835 | 3:189357199_G_T |
| huang_2021_study_17 | Huang (2021) | rs7705526 | C | 5:1285974 | 1 | 1 | 1 | rs7705526 | 5:1285974_C_A |
| huang_2021_study_17 | Huang (2021) | rs116822326 | A | 6:31434111 | 0 | 1 | 1 | rs3094604 | 6:31434111_A_G |
| huang_2021_study_17 | Huang (2021) | rs6920364 | G | 6:167376466 | 1 | 1 | 1 | rs6920364 | 6:167376466_G_C |
| huang_2021_study_17 | Huang (2021) | rs11780471 | A | 8:27344719 | 1 | 1 | 1 | rs11780471 | 8:27344719_G_A |
| huang_2021_study_17 | Huang (2021) | rs4236709 | A | 8:32410110 | 1 | 1 | 1 | rs4236709 | 8:32410110_G_A |
| huang_2021_study_17 | Huang (2021) | rs885518 | A | 9:21830157 | 1 | 1 | 1 | rs885518 | 9:21830157_A_G |
| huang_2021_study_17 | Huang (2021) | rs11591710 | A | 10:105687632 | 1 | 1 | 1 | rs11591710 | 10:105687632_A_C |
| huang_2021_study_17 | Huang (2021) | rs1056562 | C | 11:118125625 | 1 | 1 | 1 | rs1056562 | 11:118125625_T_C |
| huang_2021_study_17 | Huang (2021) | rs7953330 | C | 12:998819 | 1 | 1 | 1 | rs7953330 | 12:998819_G_C |
| huang_2021_study_17 | Huang (2021) | rs11571833 | A | 13:32972626 | 1 | 1 | 1 | rs11571833 | rs11571833 |
| huang_2021_study_17 | Huang (2021) | rs55781567 | C | 15:78857986 | 1 | 1 | 1 | rs55781567 | rs55781567 |
| huang_2021_study_17 | Huang (2021) | rs77468143 | G | 15:49376624 | 1 | 1 | 1 | rs77468143 | 15:49376624_T_G |
| huang_2021_study_17 | Huang (2021) | rs66759488 | G | 15:47577451 | 1 | 1 | 1 | rs66759488 | 15:47577451_G_A |
| huang_2021_study_17 | Huang (2021) | rs56113850 | T | 19:41353107 | 1 | 1 | 1 | rs56113850 | 19:41353107_T_C |
| huang_2021_study_17 | Huang (2021) | rs41309931 | G | 20:62326579 | 1 | 1 | 1 | rs41309931 | 20:62326579_G_T |
| huang_2021_study_17 | Huang (2021) | rs17879961 | G | 22:29121087 | 1 | 1 | 1 | rs17879961 | rs17879961 |
| jia_2021_study_18 | Jia (2021) | rs71658797 | A | 1:77967507 | 1 | 1 | 1 | rs71658797 | 1:77967507_T_A |
| jia_2021_study_18 | Jia (2021) | rs13080835 | G | 3:189357199 | 1 | 1 | 1 | rs13080835 | 3:189357199_G_T |
| jia_2021_study_18 | Jia (2021) | rs7705526 | A | 5:1285974 | 1 | 1 | 1 | rs7705526 | 5:1285974_C_A |
| jia_2021_study_18 | Jia (2021) | rs2853677 | G | 5:1287194 | 1 | 1 | 1 | rs2853677 | rs2853677 |
| jia_2021_study_18 | Jia (2021) | rs465498 | A | 5:1325803 | 1 | 1 | 1 | rs465498 | 5:1325803_A_G |
| jia_2021_study_18 | Jia (2021) | rs3115672 | T | 6:31727897 | 1 | 1 | 1 | rs3115672 | 6:31727897_C_T |
| jia_2021_study_18 | Jia (2021) | rs6920364 | C | 6:167376466 | 1 | 1 | 1 | rs6920364 | 6:167376466_G_C |
| jia_2021_study_18 | Jia (2021) | rs4236709 | G | 8:32410110 | 1 | 1 | 1 | rs4236709 | 8:32410110_G_A |
| jia_2021_study_18 | Jia (2021) | rs11780471 | G | 8:27344719 | 1 | 1 | 1 | rs11780471 | 8:27344719_G_A |
| jia_2021_study_18 | Jia (2021) | rs885518 | G | 9:21830157 | 1 | 1 | 1 | rs885518 | 9:21830157_A_G |
| jia_2021_study_18 | Jia (2021) | rs62560775 | G | 9:22052068 | 1 | 1 | 1 | rs62560775 | 9:22052068_A_G |
| jia_2021_study_18 | Jia (2021) | rs1056562 | T | 11:118125625 | 1 | 1 | 1 | rs1056562 | 11:118125625_T_C |
| jia_2021_study_18 | Jia (2021) | rs7953330 | G | 12:998819 | 1 | 1 | 1 | rs7953330 | 12:998819_G_C |
| jia_2021_study_18 | Jia (2021) | rs11571833 | T | 13:32972626 | 1 | 1 | 1 | rs11571833 | rs11571833 |
| jia_2021_study_18 | Jia (2021) | rs66759488 | A | 15:47577451 | 1 | 1 | 1 | rs66759488 | 15:47577451_G_A |
| jia_2021_study_18 | Jia (2021) | rs77468143 | T | 15:49376624 | 1 | 1 | 1 | rs77468143 | 15:49376624_T_G |
| jia_2021_study_18 | Jia (2021) | rs55781567 | G | 15:78857986 | 1 | 1 | 1 | rs55781567 | rs55781567 |
| jia_2021_study_18 | Jia (2021) | rs8042374 | A | 15:78908032 | 1 | 1 | 1 | rs8042374 | 15:78908032_A_G |
| jia_2021_study_18 | Jia (2021) | rs56113850 | C | 19:41353107 | 1 | 1 | 1 | rs56113850 | 19:41353107_T_C |
| liang_2023_study_35 | Liang (2023) | rs71658797 | T | 1:NA | 1 | 0 | 1 | NA | NA |
| liang_2023_study_35 | Liang (2023) | rs13080835 | G | 3:NA | 1 | 0 | 1 | NA | NA |
| liang_2023_study_35 | Liang (2023) | rs7705526 | C | 5:NA | 1 | 0 | 1 | NA | NA |
| liang_2023_study_35 | Liang (2023) | rs6920364 | G | 6:NA | 1 | 0 | 1 | NA | NA |
| liang_2023_study_35 | Liang (2023) | rs116822326 | A | 6:NA | 0 | 0 | 0 | NA | NA |
| liang_2023_study_35 | Liang (2023) | rs11780471 | G | 8:NA | 1 | 0 | 1 | NA | NA |
| liang_2023_study_35 | Liang (2023) | rs4236709 | A | 8:NA | 1 | 0 | 1 | NA | NA |
| liang_2023_study_35 | Liang (2023) | rs885518 | A | 9:NA | 1 | 0 | 1 | NA | NA |
| liang_2023_study_35 | Liang (2023) | rs11591710 | A | 10:NA | 1 | 0 | 1 | NA | NA |
| liang_2023_study_35 | Liang (2023) | rs1056562 | C | 11:NA | 1 | 0 | 1 | NA | NA |
| liang_2023_study_35 | Liang (2023) | rs7953330 | G | 12:NA | 1 | 0 | 1 | NA | NA |
| liang_2023_study_35 | Liang (2023) | rs11571833 | A | 13:NA | 1 | 0 | 1 | NA | NA |

## S9 List of Included SNPs

| PGS_name | Label | rsID | Effect allele | Position | rsID in UKB extract | position in UKB extract | found in UKB extract | rsID in UKB | Position in UKB |
|---|---|---|---|---|---|---|---|---|---|
| liang_2023_study_35 | Liang (2023) | rs66759488 | G | 15:NA | 1 | 0 | 1 | NA | NA |
| liang_2023_study_35 | Liang (2023) | rs55781567 | C | 15:NA | 1 | 0 | 1 | NA | NA |
| liang_2023_study_35 | Liang (2023) | rs77468143 | T | 15:NA | 1 | 0 | 1 | NA | NA |
| liang_2023_study_35 | Liang (2023) | rs56113850 | C | 19:NA | 1 | 0 | 1 | NA | NA |
| liang_2023_study_35 | Liang (2023) | rs41309931 | G | 20:NA | 1 | 0 | 1 | NA | NA |
| liang_2023_study_35 | Liang (2023) | rs17879961 | A | 22:NA | 1 | 0 | 1 | NA | NA |
| liu_2022_study_4_A | Liu (2022a) | rs71658797 | A | 1:77967507 | 1 | 1 | 1 | rs71658797 | 1:77967507_T_A |
| liu_2022_study_4_A | Liu (2022a) | rs13080835 | G | 3:189357199 | 1 | 1 | 1 | rs13080835 | 3:189357199_G_T |
| liu_2022_study_4_A | Liu (2022a) | rs7705526 | A | 5:1285974 | 1 | 1 | 1 | rs7705526 | 5:1285974_C_A |
| liu_2022_study_4_A | Liu (2022a) | rs31489 | C | 5:1342714 | 0 | 0 | 0 | NA | NA |
| liu_2022_study_4_A | Liu (2022a) | rs1611182 | T | 6:29781049 | 1 | 1 | 1 | rs1611182 | 6:29781049_T_G |
| liu_2022_study_4_A | Liu (2022a) | rs3117582 | C | 6:31620520 | 0 | 0 | 0 | NA | NA |
| liu_2022_study_4_A | Liu (2022a) | rs4236709 | G | 8:32410110 | 1 | 1 | 1 | rs4236709 | 8:32410110_G_A |
| liu_2022_study_4_A | Liu (2022a) | rs885518 | G | 9:21830157 | 1 | 1 | 1 | rs885518 | 9:21830157_A_G |
| liu_2022_study_4_A | Liu (2022a) | rs62560775 | G | 9:22052068 | 1 | 1 | 1 | rs62560775 | 9:22052068_A_G |
| liu_2022_study_4_A | Liu (2022a) | rs11591710 | C | 10:105687632 | 1 | 1 | 1 | rs11591710 | 10:105687632_A_C |
| liu_2022_study_4_A | Liu (2022a) | rs1056562 | T | 11:118125625 | 1 | 1 | 1 | rs1056562 | 11:118125625_T_C |
| liu_2022_study_4_A | Liu (2022a) | rs77468143 | T | 15:49376624 | 1 | 1 | 1 | rs77468143 | 15:49376624_T_G |
| liu_2022_study_4_A | Liu (2022a) | rs1051730 | T | 15:78894339 | 1 | 1 | 1 | rs1051730 | rs1051730 |
| liu_2022_study_4_A | Liu (2022a) | rs28408315 | G | 15:79185180 | 1 | 1 | 1 | rs28408315 | 15:79185180_G_A |
| liu_2022_study_4_A | Liu (2022a) | rs56113850 | C | 19:41353107 | 1 | 1 | 1 | rs56113850 | 19:41353107_T_C |
| liu_2022_study_4_B | Liu (2022b) | rs467095 | T | 5:1336221 | 1 | 1 | 1 | rs467095 | 5:1336221_T_C |
| liu_2022_study_4_B | Liu (2022b) | rs3094604 | G | 6:31434111 | 1 | 1 | 1 | rs3094604 | 6:31434111_A_G |
| liu_2022_study_4_B | Liu (2022b) | rs7953330 | G | 12:998819 | 1 | 1 | 1 | rs7953330 | 12:998819_G_C |
| liu_2022_study_4_B | Liu (2022b) | rs11571818 | C | 13:32968810 | 1 | 1 | 1 | rs11571818 | 13:32968810_T_C |
| liu_2022_study_4_B | Liu (2022b) | rs8040868 | C | 15:78911181 | 1 | 1 | 1 | rs8040868 | rs8040868 |
| liu_2022_study_4_B | Liu (2022b) | rs56113850 | C | 19:41353107 | 1 | 1 | 1 | rs56113850 | 19:41353107_T_C |
| qian_2016_study22 | Qian (2016) | rs8040868 | C | 15:78911181 | 1 | 1 | 1 | rs8040868 | rs8040868 |
| qian_2016_study22 | Qian (2016) | rs147144681 | T | 15:78900908 | 1 | 1 | 1 | rs147144681 | 15:78900908_C_T |
| qian_2016_study22 | Qian (2016) | rs7169584 | T | 15:78822660 | 1 | 1 | 1 | rs7169584 | 15:78822660_A_T |
| qian_2016_study22 | Qian (2016) | rs421629 | A | 5:1320136 | 1 | 1 | 1 | rs421629 | 5:1320136_G_A |
| qian_2016_study22 | Qian (2016) | rs1150753 | G | 6:32059867 | 1 | 1 | 1 | rs1150753 | rs1150753 |
| qian_2016_study22 | Qian (2016) | rs58049067 | C | 17:7621561 | 1 | 1 | 1 | rs58049067 | 17:7621561_T_C |
| qian_2016_study22 | Qian (2016) | rs9271626 | A | 6:32591944 | 1 | 1 | 1 | rs9271626 | 6:32591944_C_A |
| qian_2016_study22 | Qian (2016) | rs3821170 | T | 2:207453310 | 1 | 1 | 1 | rs3821170 | 2:207453310_C_T |
| qian_2016_study22 | Qian (2016) | rs9522182 | G | 13:111973816 | 1 | 1 | 1 | rs9522182 | rs9522182 |
| qian_2016_study22 | Qian (2016) | rs61782077 | T | 1:51897058 | 1 | 1 | 1 | rs61782077 | 1:51897058_C_T |
| qian_2016_study22 | Qian (2016) | rs141182035 | T | 10:18651810 | 1 | 1 | 1 | rs141182035 | 10:18651810_C_T |
| qian_2016_study22 | Qian (2016) | rs148731271 | C | 7:157302689 | 1 | 1 | 1 | rs148731271 | 7:157302689_A_C |
| qian_2016_study22 | Qian (2016) | rs79428462 | T | 3:119882568 | 1 | 1 | 1 | rs79428462 | 3:119882568_C_T |
| qian_2016_study22 | Qian (2016) | rs8136157 | T | 22:26430757 | 1 | 1 | 1 | rs8136157 | 22:26430757_C_T |
| qian_2016_study22 | Qian (2016) | rs76933969 | G | 3:63099587 | 1 | 1 | 1 | rs76933969 | 3:63099587_T_G |
| qian_2016_study22 | Qian (2016) | rs74799686 | T | 18:76400817 | 1 | 1 | 1 | rs74799686 | 18:76400817_G_T |
| qian_2016_study22 | Qian (2016) | rs4412193 | G | 6:26338056 | 1 | 1 | 1 | rs4412193 | rs4412193 |
| qian_2016_study22 | Qian (2016) | rs9534269 | G | 13:32939286 | 1 | 1 | 1 | rs9534269 | 13:32939286_T_G |
| qian_2016_study22 | Qian (2016) | rs2392656 | A | 2:221179018 | 1 | 1 | 1 | rs2392656 | 2:221179018_A_G |
| qian_2016_study22 | Qian (2016) | rs11853760 | A | 15:47518372 | 1 | 1 | 1 | rs11853760 | 15:47518372_T_A |
| qian_2016_study22 | Qian (2016) | rs11190431 | A | 10:101997490 | 1 | 1 | 1 | rs11190431 | 10:101997490_G_A |
| qian_2016_study22 | Qian (2016) | rs2647964 | G | 12:115985822 | 1 | 1 | 1 | rs2647964 | 12:115985822_A_G |
| qian_2016_study22 | Qian (2016) | rs26534 | A | 5:115175701 | 1 | 1 | 1 | rs26534 | 5:115175701_G_A |
| qian_2016_study22 | Qian (2016) | rs12459781 | T | 19:3762119 | 1 | 1 | 1 | rs12459781 | 19:3762119_T_C |
| qian_2016_study22 | Qian (2016) | rs7659396 | A | 4:180930095 | 1 | 1 | 1 | rs7659396 | 4:180930095_A_G |
| qian_2016_study22 | Qian (2016) | rs10761413 | G | 9:136461381 | 1 | 1 | 1 | rs10761413 | 9:136461381_G_A |
| qian_2016_study22 | Qian (2016) | rs2974937 | C | 1:155168849 | 1 | 1 | 1 | rs2974937 | 1:155168849_C_T |
| qian_2016_study22 | Qian (2016) | rs4619206 | T | 12:1002857 | 1 | 1 | 1 | rs4619206 | 12:1002857_T_C |
| qian_2016_study22 | Qian (2016) | rs10757221 | A | 9:2150467 | 1 | 1 | 1 | rs10757221 | 9:2150467_A_G |
| qian_2016_study22 | Qian (2016) | rs329118 | T | 5:133861663 | 1 | 1 | 1 | rs329118 | 5:133861663_C_T |
| qian_2016_study22 | Qian (2016) | rs58151545 | G | 5:167624533 | 1 | 1 | 1 | rs58151545 | 5:167624533_A_G |
| qian_2016_study22 | Qian (2016) | rs12440453 | T | 15:40691797 | 1 | 1 | 1 | rs12440453 | 15:40691797_C_T |
| qian_2016_study22 | Qian (2016) | rs34718042 | T | 9:5643595 | 1 | 1 | 1 | rs34718042 | 9:5643595_G_T |
| qian_2016_study22 | Qian (2016) | rs11205949 | G | 1:52744754 | 1 | 1 | 1 | rs11205949 | 1:52744754_T_G |
| qian_2016_study22 | Qian (2016) | rs62288380 | A | 3:175277628 | 1 | 1 | 1 | rs62288380 | 3:175277628_G_A |
| qian_2016_study22 | Qian (2016) | rs72969776 | T | 6:126191203 | 1 | 1 | 1 | rs72969776 | 6:126191203_C_T |
| qian_2016_study22 | Qian (2016) | rs7045009 | T | 9:7070962 | 1 | 1 | 1 | rs7045009 | 9:7070962_T_C |
| qian_2016_study22 | Qian (2016) | rs1521654 | T | 2:213027954 | 1 | 1 | 1 | rs1521654 | 2:213027954_T_A |

## S9 List of Included SNPs

| PGS_name | Label | rsID | Effect allele | Position | rsID in UKB extract | position in UKB extract | found in UKB extract | rsID in UKB | Position in UKB |
|---|---|---|---|---|---|---|---|---|---|
| qian_2016_study22 | Qian (2016) | rs6414428 | A | 3:46518003 | 1 | 1 | 1 | rs6414428 | 3:46518003_A_G |
| qian_2016_study22 | Qian (2016) | rs6879664 | G | 5:178974576 | 1 | 1 | 1 | rs6879664 | 5:178974576_T_G |
| qian_2016_study22 | Qian (2016) | rs17806239 | G | 18:55936568 | 1 | 1 | 1 | rs17806239 | rs17806239 |
| qian_2016_study22 | Qian (2016) | rs2779413 | C | 1:237737703 | 1 | 1 | 1 | rs2779413 | 1:237737703_C_G |
| qian_2016_study22 | Qian (2016) | rs143966858 | G | 12:21471110 | 1 | 1 | 1 | rs143966858 | 12:21471110_A_G |
| qian_2016_study22 | Qian (2016) | rs16852464 | A | 3:142344984 | 1 | 1 | 1 | rs16852464 | 3:142344984_G_A |
| qian_2016_study22 | Qian (2016) | rs55920079 | C | 11:57410184 | 1 | 1 | 1 | rs55920079 | 11:57410184_T_C |
| qian_2016_study22 | Qian (2016) | rs73371615 | A | 21:43178590 | 1 | 1 | 1 | rs73371615 | 21:43178590_G_A |
| qian_2016_study22 | Qian (2016) | rs7078193 | T | 10:13714646 | 1 | 1 | 1 | rs7078193 | 10:13714646_C_T |
| qian_2016_study22 | Qian (2016) | rs192623780 | G | 14:50066542 | 1 | 1 | 1 | rs192623780 | 14:50066542_G_C |
| qian_2016_study22 | Qian (2016) | rs9609937 | T | 22:34460669 | 1 | 1 | 1 | rs9609937 | 22:34460669_C_T |
| qian_2016_study22 | Qian (2016) | rs2427022 | A | 20:59852354 | 1 | 1 | 1 | rs2427022 | 20:59852354_A_C |
| qian_2016_study22 | Qian (2016) | rs73014280 | C | 6:154390894 | 1 | 1 | 1 | rs73014280 | 6:154390894_T_C |
| qian_2016_study22 | Qian (2016) | rs74065139 | G | 12:13744036 | 1 | 1 | 1 | rs74065139 | 12:13744036_T_G |
| qian_2016_study22 | Qian (2016) | rs62014008 | C | 16:7347957 | 1 | 1 | 1 | rs62014008 | 16:7347957_C_T |
| qian_2016_study22 | Qian (2016) | rs118039559 | T | 16:75262626 | 1 | 1 | 1 | rs118039559 | 16:75262626_C_T |
| qian_2016_study22 | Qian (2016) | rs28412344 | G | 8:1460750 | 1 | 1 | 1 | rs28412344 | 8:1460750_G_A |
| qian_2016_study22 | Qian (2016) | rs4646515 | C | 19:15769379 | 1 | 1 | 1 | rs4646515 | 19:15769379_G_C |
| qian_2016_study22 | Qian (2016) | rs825733 | C | 15:43581506 | 1 | 1 | 1 | rs825733 | 15:43581506_T_C |
| qian_2016_study22 | Qian (2016) | rs239935 | G | 6:167411788 | 1 | 1 | 1 | rs239935 | 6:167411788_G_A |
| qian_2016_study22 | Qian (2016) | rs235038 | A | 20:48238437 | 1 | 1 | 1 | rs235038 | 20:48238437_A_G |
| qian_2016_study22 | Qian (2016) | rs1810807 | C | 9:32784838 | 1 | 1 | 1 | rs1810807 | rs1810807 |
| qian_2016_study22 | Qian (2016) | rs75240553 | C | 2:51146900 | 1 | 1 | 1 | rs75240553 | 2:51146900_T_C |
| qian_2016_study22 | Qian (2016) | rs149341491 | C | 16:84047110 | 1 | 1 | 1 | rs149341491 | 16:84047110_A_C |
| qian_2016_study22 | Qian (2016) | rs139801073 | A | 2:32709224 | 1 | 1 | 1 | rs139801073 | 2:32709224_G_A |
| qian_2016_study22 | Qian (2016) | rs4019548 | T | 12:94979560 | 1 | 1 | 1 | rs4019548 | 12:94979560_T_C |
| qian_2016_study22 | Qian (2016) | rs659051 | C | 6:4119029 | 1 | 1 | 1 | rs659051 | 6:4119029_T_C |
| qian_2016_study22 | Qian (2016) | rs55647829 | T | 16:5980131 | 1 | 1 | 1 | rs55647829 | 16:5980131_C_T |
| qian_2016_study22 | Qian (2016) | rs10833116 | A | 11:19478945 | 1 | 1 | 1 | rs10833116 | 11:19478945_G_A |
| qian_2016_study22 | Qian (2016) | rs10115585 | A | 9:117370196 | 1 | 1 | 1 | rs10115585 | 9:117370196_A_C |
| qian_2016_study22 | Qian (2016) | rs11015249 | C | 10:27011863 | 1 | 1 | 1 | rs11015249 | 10:27011863_T_C |
| qian_2016_study22 | Qian (2016) | rs72844782 | A | 2:133503708 | 1 | 1 | 1 | rs72844782 | 2:133503708_G_A |
| qian_2016_study22 | Qian (2016) | rs2250241 | T | 11:4413775 | 1 | 1 | 1 | rs2250241 | 11:4413775_T_C |
| qian_2016_study22 | Qian (2016) | rs10095018 | C | 8:134339266 | 1 | 1 | 1 | rs10095018 | rs10095018 |
| qian_2016_study22 | Qian (2016) | rs859052 | C | 1:95371727 | 1 | 1 | 1 | rs859052 | 1:95371727_C_T |
| qian_2016_study22 | Qian (2016) | rs117312475 | T | 8:3540889 | 1 | 1 | 1 | rs117312475 | rs117312475 |
| qian_2016_study22 | Qian (2016) | rs189225157 | T | 5:160300960 | 0 | 1 | 1 | rs62391161 | 5:160300960_G_T |
| qian_2016_study22 | Qian (2016) | rs12474031 | A | 2:34379323 | 1 | 1 | 1 | rs12474031 | 2:34379323_G_A |
| qian_2016_study22 | Qian (2016) | rs1026926 | A | 4:96356534 | 1 | 1 | 1 | rs1026926 | rs1026926 |
| qian_2016_study22 | Qian (2016) | rs146483838 | C | 3:29401390 | 0 | 1 | 1 | rs9755128 | 3:29401390_C_T |
| qian_2016_study22 | Qian (2016) | rs17527232 | G | 12:28699824 | 1 | 1 | 1 | rs17527232 | 12:28699824_A_G |
| qian_2016_study22 | Qian (2016) | rs1461314 | C | 10:14120659 | 1 | 1 | 1 | rs1461314 | 10:14120659_G_C |
| qian_2016_study22 | Qian (2016) | rs10430983 | C | 11:40891793 | 1 | 1 | 1 | rs10430983 | 11:40891793_C_T |
| qian_2016_study22 | Qian (2016) | rs9862711 | T | 3:47086518 | 1 | 1 | 1 | rs9862711 | 3:47086518_C_T |
| qian_2016_study22 | Qian (2016) | rs117656063 | A | 9:124441641 | 1 | 1 | 1 | rs117656063 | 9:124441641_C_A |
| qian_2016_study22 | Qian (2016) | rs4490841 | T | 8:25727467 | 1 | 1 | 1 | rs4490841 | 8:25727467_C_T |
| qian_2016_study22 | Qian (2016) | rs2851682 | G | 11:61616012 | 1 | 1 | 1 | rs2851682 | 11:61616012_A_G |
| qian_2016_study22 | Qian (2016) | rs75137824 | T | 11:35233778 | 1 | 1 | 1 | rs75137824 | 11:35233778_A_T |
| qian_2016_study22 | Qian (2016) | rs6748389 | G | 2:240685573 | 1 | 1 | 1 | rs6748389 | 2:240685573_T_G |
| qian_2016_study22 | Qian (2016) | rs4075554 | G | 16:83549627 | 1 | 1 | 1 | rs4075554 | 16:83549627_A_G |
| qian_2016_study22 | Qian (2016) | rs2177988 | G | 7:76872508 | 1 | 1 | 1 | rs2177988 | 7:76872508_G_A |
| qian_2016_study22 | Qian (2016) | rs11711351 | A | 3:134821129 | 1 | 1 | 1 | rs11711351 | 3:134821129_G_A |
| qian_2016_study22 | Qian (2016) | rs10825124 | T | 10:55612062 | 1 | 1 | 1 | rs10825124 | 10:55612062_C_T |
| qian_2016_study22 | Qian (2016) | rs35215597 | G | 2:175543782 | 1 | 1 | 1 | rs35215597 | 2:175543782_A_G |
| qian_2016_study22 | Qian (2016) | rs58073039 | G | 4:88287363 | 1 | 1 | 1 | rs58073039 | 4:88287363_A_G |
| qian_2016_study22 | Qian (2016) | rs61817959 | A | 1:204492522 | 1 | 1 | 1 | rs61817959 | 1:204492522_G_A |
| qian_2016_study22 | Qian (2016) | rs4522161 | T | 11:40835397 | 1 | 1 | 1 | rs4522161 | 11:40835397_T_C |
| qian_2016_study22 | Qian (2016) | rs3852860 | T | 19:45382966 | 1 | 1 | 1 | rs3852860 | rs3852860 |
| qian_2016_study22 | Qian (2016) | rs71636088 | G | 5:73122067 | 1 | 1 | 1 | rs71636088 | 5:73122067_A_G |
| qian_2016_study22 | Qian (2016) | rs3172494 | T | 3:48731487 | 1 | 1 | 1 | rs3172494 | 3:48731487_G_T |
| qian_2016_study22 | Qian (2016) | rs75227923 | A | 1:199217117 | 1 | 1 | 1 | rs75227923 | 1:199217117_G_A |
| qian_2016_study22 | Qian (2016) | rs11079222 | G | 17:54415434 | 1 | 1 | 1 | rs11079222 | 17:54415434_A_G |
| qian_2016_study22 | Qian (2016) | rs1784684 | G | 11:113939539 | 1 | 1 | 1 | rs1784684 | 11:113939539_A_G |
| qian_2016_study22 | Qian (2016) | rs6546524 | A | 2:69719177 | 1 | 1 | 1 | rs6546524 | 2:69719177_A_C |
| qian_2016_study22 | Qian (2016) | rs28455901 | C | 12:3284029 | 1 | 1 | 1 | rs28455901 | 12:3284029_T_C |

## S9 List of Included SNPs

| PGS_name | Label | rsID | Effect allele | Position | rsID in UKB extract | position in UKB extract | found in UKB extract | rsID in UKB | Position in UKB |
|---|---|---|---|---|---|---|---|---|---|
| qian_2016_study22 | Qian (2016) | rs6776006 | A | 3:58643577 | 1 | 1 | 1 | rs6776006 | 3:58643577_A_C |
| qian_2016_study22 | Qian (2016) | rs8034099 | A | 15:80725930 | 1 | 1 | 1 | rs8034099 | 15:80725930_A_G |
| qian_2016_study22 | Qian (2016) | rs7476396 | C | 10:79006107 | 1 | 1 | 1 | rs7476396 | 10:79006107_A_C |
| qian_2016_study22 | Qian (2016) | rs9973793 | T | 2:54998516 | 1 | 1 | 1 | rs9973793 | 2:54998516_C_T |
| qian_2016_study22 | Qian (2016) | rs11077010 | C | 16:6251339 | 1 | 1 | 1 | rs11077010 | 16:6251339_T_C |
| qian_2016_study22 | Qian (2016) | rs66500423 | C | 19:41195170 | 1 | 1 | 1 | rs66500423 | 19:41195170_T_C |
| qian_2016_study22 | Qian (2016) | rs5025580 | A | 10:134798532 | 1 | 1 | 1 | rs5025580 | 10:134798532_A_G |
| qian_2016_study22 | Qian (2016) | rs9399783 | T | 6:103455376 | 1 | 1 | 1 | rs9399783 | 6:103455376_G_T |
| qian_2016_study22 | Qian (2016) | rs35121269 | T | 14:31677894 | 1 | 1 | 1 | rs35121269 | 14:31677894_T_C |
| qian_2016_study22 | Qian (2016) | rs7781386 | T | 7:16142180 | 1 | 1 | 1 | rs7781386 | 7:16142180_T_C |
| qian_2016_study22 | Qian (2016) | rs4820695 | A | 22:27075975 | 1 | 1 | 1 | rs4820695 | 22:27075975_G_A |
| qian_2016_study22 | Qian (2016) | rs6455355 | G | 6:70829516 | 1 | 1 | 1 | rs6455355 | 6:70829516_A_G |
| qian_2016_study22 | Qian (2016) | rs79793393 | G | 9:113551727 | 1 | 1 | 1 | rs79793393 | rs79793393 |
| qian_2016_study22 | Qian (2016) | rs8136042 | C | 22:33014552 | 1 | 1 | 1 | rs8136042 | 22:33014552_T_C |
| qian_2016_study22 | Qian (2016) | rs140000715 | A | 22:29137095 | 1 | 1 | 1 | rs140000715 | 22:29137095_G_A |
| qian_2016_study22 | Qian (2016) | rs11257111 | C | 10:11513365 | 1 | 1 | 1 | rs11257111 | 10:11513365_A_C |
| qian_2016_study22 | Qian (2016) | rs2239064 | C | 12:2529528 | 1 | 1 | 1 | rs2239064 | 12:2529528_T_C |
| qian_2016_study22 | Qian (2016) | rs6702611 | G | 1:212961710 | 1 | 1 | 1 | rs6702611 | 1:212961710_G_A |
| qian_2016_study22 | Qian (2016) | rs2668215 | A | 3:112291036 | 1 | 1 | 1 | rs2668215 | 3:112291036_G_A |
| qian_2016_study22 | Qian (2016) | rs157577 | G | 5:131563571 | 1 | 1 | 1 | rs157577 | 5:131563571_G_C |
| qian_2016_study22 | Qian (2016) | rs12671174 | C | 7:10061458 | 1 | 1 | 1 | rs12671174 | 7:10061458_T_C |
| qian_2016_study22 | Qian (2016) | rs4236676 | A | 8:27826339 | 1 | 1 | 1 | rs4236676 | 8:27826339_G_A |
| qian_2016_study22 | Qian (2016) | rs214278 | A | 14:73605495 | 1 | 1 | 1 | rs214278 | 14:73605495_A_G |
| qian_2016_study22 | Qian (2016) | rs35561731 | A | 10:107258826 | 1 | 1 | 1 | rs35561731 | 10:107258826_G_A |
| qian_2016_study22 | Qian (2016) | rs2278529 | G | 2:219502132 | 1 | 1 | 1 | rs2278529 | 2:219502132_G_A |
| qian_2016_study22 | Qian (2016) | rs35668984 | C | 3:7495494 | 1 | 1 | 1 | rs35668984 | 3:7495494_A_C |
| qian_2016_study22 | Qian (2016) | rs7581528 | A | 2:47240149 | 1 | 1 | 1 | rs7581528 | 2:47240149_G_A |
| qian_2016_study22 | Qian (2016) | rs72862345 | G | 17:80527430 | 1 | 1 | 1 | rs72862345 | 17:80527430_T_G |
| qian_2016_study22 | Qian (2016) | rs10744725 | A | 12:840736 | 1 | 1 | 1 | rs10744725 | 12:840736_A_G |
| qian_2016_study22 | Qian (2016) | rs72808464 | C | 2:58172111 | 1 | 1 | 1 | rs72808464 | 2:58172111_G_C |
| qian_2016_study22 | Qian (2016) | rs2916512 | C | 2:80126150 | 1 | 1 | 1 | rs2916512 | 2:80126150_A_C |
| qian_2016_study22 | Qian (2016) | rs56897871 | C | 2:59094867 | 1 | 1 | 1 | rs56897871 | 2:59094867_A_C |
| qian_2016_study22 | Qian (2016) | rs13098369 | A | 3:32732253 | 1 | 1 | 1 | rs13098369 | 3:32732253_C_A |
| qian_2016_study22 | Qian (2016) | rs512309 | G | 1:66665720 | 1 | 1 | 1 | rs512309 | 1:66665720_A_G |
| qian_2016_study22 | Qian (2016) | rs13127163 | A | 4:16528453 | 1 | 1 | 1 | rs13127163 | 4:16528453_C_A |
| qian_2016_study22 | Qian (2016) | rs10281764 | G | 7:101132669 | 1 | 1 | 1 | rs10281764 | 7:101132669_A_G |
| qian_2016_study22 | Qian (2016) | rs1537011 | C | 9:117820549 | 1 | 1 | 1 | rs1537011 | 9:117820549_G_C |
| qian_2016_study22 | Qian (2016) | rs3111791 | A | 4:55565408 | 1 | 1 | 1 | rs3111791 | 4:55565408_A_G |
| qian_2016_study22 | Qian (2016) | rs60102864 | A | 16:88669858 | 1 | 1 | 1 | rs60102864 | 16:88669858_A_G |
| qian_2016_study22 | Qian (2016) | rs11665454 | C | 18:68432346 | 1 | 1 | 1 | rs11665454 | 18:68432346_T_C |
| qian_2016_study22 | Qian (2016) | rs12366677 | T | 12:41790695 | 1 | 1 | 1 | rs12366677 | 12:41790695_A_T |
| qian_2016_study22 | Qian (2016) | rs10818289 | C | 9:121993508 | 1 | 1 | 1 | rs10818289 | 9:121993508_T_C |
| qian_2016_study22 | Qian (2016) | rs12227199 | G | 12:100017821 | 1 | 1 | 1 | rs12227199 | 12:100017821_C_G |
| qian_2016_study22 | Qian (2016) | rs6931657 | C | 6:9829023 | 1 | 1 | 1 | rs6931657 | 6:9829023_T_C |
| qian_2016_study22 | Qian (2016) | rs117477664 | A | 19:9050842 | 1 | 1 | 1 | rs117477664 | 19:9050842_T_A |
| qian_2016_study22 | Qian (2016) | rs1127065 | T | 7:44259871 | 1 | 1 | 1 | rs1127065 | 7:44259871_C_T |
| qian_2016_study22 | Qian (2016) | rs56854738 | A | 1:232199482 | 1 | 1 | 1 | rs56854738 | 1:232199482_G_A |
| qian_2016_study22 | Qian (2016) | rs7908298 | T | 10:327780 | 1 | 1 | 1 | rs7908298 | 10:327780_C_T |
| qian_2016_study22 | Qian (2016) | rs7155809 | A | 14:34125314 | 1 | 1 | 1 | rs7155809 | rs7155809 |
| qian_2016_study22 | Qian (2016) | rs9644443 | T | 8:140894951 | 1 | 1 | 1 | rs9644443 | 8:140894951_C_T |
| qian_2016_study22 | Qian (2016) | rs37606 | G | 16:75508173 | 1 | 1 | 1 | rs37606 | 16:75508173_A_G |
| qian_2016_study22 | Qian (2016) | rs2107141 | G | 7:11522119 | 1 | 1 | 1 | rs2107141 | 7:11522119_G_A |
| qian_2016_study22 | Qian (2016) | rs11758872 | T | 6:40404151 | 1 | 1 | 1 | rs11758872 | 6:40404151_C_T |
| qian_2016_study22 | Qian (2016) | rs6703620 | A | 1:151013500 | 1 | 1 | 1 | rs6703620 | 1:151013500_A_T |
| qian_2016_study22 | Qian (2016) | rs12563994 | T | 1:155244092 | 1 | 1 | 1 | rs12563994 | 1:155244092_C_T |
| qian_2016_study22 | Qian (2016) | rs17121879 | G | 11:118080133 | 1 | 1 | 1 | rs17121879 | 11:118080133_G_T |
| qian_2016_study22 | Qian (2016) | rs11627837 | C | 14:34121869 | 1 | 1 | 1 | rs11627837 | rs11627837 |
| qian_2016_study22 | Qian (2016) | rs79644670 | A | 11:36083000 | 1 | 1 | 1 | rs79644670 | 11:36083000_G_A |
| qian_2016_study22 | Qian (2016) | rs12511912 | C | 4:37642867 | 1 | 1 | 1 | rs12511912 | 4:37642867_A_C |
| qian_2016_study22 | Qian (2016) | rs28796910 | G | 18:41910532 | 1 | 1 | 1 | rs28796910 | 18:41910532_A_G |
| qian_2016_study22 | Qian (2016) | rs183459006 | A | 13:64260736 | 1 | 1 | 1 | rs183459006 | 13:64260736_G_A |
| qian_2016_study22 | Qian (2016) | rs74890148 | A | 1:7284420 | 1 | 1 | 1 | rs74890148 | 1:7284420_C_A |
| qian_2016_study22 | Qian (2016) | rs1878221 | T | 4:183302059 | 1 | 1 | 1 | rs1878221 | 4:183302059_T_C |
| qian_2016_study22 | Qian (2016) | rs7265275 | T | 20:61713394 | 1 | 1 | 1 | rs7265275 | 20:61713394_C_T |
| qian_2016_study22 | Qian (2016) | rs144349639 | C | 1:6658874 | 1 | 1 | 1 | rs144349639 | 1:6658874_T_C |

## S9 List of Included SNPs

| PGS_name | Label | rsID | Effect allele | Position | rsID in UKB extract | position in UKB extract | found in UKB extract | rsID in UKB | Position in UKB |
|---|---|---|---|---|---|---|---|---|---|
| qian_2016_study22 | Qian (2016) | rs10761003 | G | 9:106722483 | 1 | 1 | 1 | rs10761003 | 9:106722483_G_C |
| qian_2016_study22 | Qian (2016) | rs706157 | T | 7:134103235 | 1 | 1 | 1 | rs706157 | 7:134103235_C_T |
| qian_2016_study22 | Qian (2016) | rs7604288 | G | 2:173998431 | 1 | 1 | 1 | rs7604288 | 2:173998431_G_T |
| qian_2016_study22 | Qian (2016) | rs544633 | C | 11:100110095 | 1 | 1 | 1 | rs544633 | 11:100110095_T_C |
| qian_2016_study22 | Qian (2016) | rs34937552 | A | 11:45123798 | 1 | 1 | 1 | rs34937552 | 11:45123798_G_A |
| qian_2016_study22 | Qian (2016) | rs10072369 | T | 5:96520112 | 1 | 1 | 1 | rs10072369 | 5:96520112_C_T |
| qian_2016_study22 | Qian (2016) | rs7424771 | A | 2:161276378 | 1 | 1 | 1 | rs7424771 | 2:161276378_G_A |
| qian_2016_study22 | Qian (2016) | rs212040 | T | 3:60038372 | 1 | 1 | 1 | rs212040 | 3:60038372_C_T |
| qian_2016_study22 | Qian (2016) | rs143165581 | C | 13:111640656 | 1 | 1 | 1 | rs143165581 | 13:111640656_T_C |
| qian_2016_study22 | Qian (2016) | rs6692032 | C | 1:228853984 | 1 | 1 | 1 | rs6692032 | 1:228853984_G_C |
| qian_2016_study22 | Qian (2016) | rs2051923 | A | 7:18976694 | 1 | 1 | 1 | rs2051923 | 7:18976694_G_A |
| qian_2016_study22 | Qian (2016) | rs1018577 | C | 20:1298227 | 1 | 1 | 1 | rs1018577 | rs1018577 |
| qian_2016_study22 | Qian (2016) | rs4748573 | T | 10:7015404 | 1 | 1 | 1 | rs4748573 | 10:7015404_G_T |
| qian_2016_study22 | Qian (2016) | rs13199422 | G | 6:21857511 | 1 | 1 | 1 | rs13199422 | rs13199422 |
| qian_2016_study22 | Qian (2016) | rs851055 | G | 17:41836860 | 1 | 1 | 1 | rs851055 | 17:41836860_G_A |
| qian_2016_study22 | Qian (2016) | rs818022 | C | 2:206871458 | 1 | 1 | 1 | rs818022 | 2:206871458_C_T |
| qian_2016_study22 | Qian (2016) | rs1195591 | C | 12:131194222 | 1 | 1 | 1 | rs1195591 | 12:131194222_C_G |
| qian_2016_study22 | Qian (2016) | rs264082 | A | 3:65791375 | 1 | 1 | 1 | rs264082 | 3:65791375_G_A |
| qian_2016_study22 | Qian (2016) | rs72954418 | C | 18:64202599 | 1 | 1 | 1 | rs72954418 | 18:64202599_T_C |
| qian_2016_study22 | Qian (2016) | rs3181030 | C | 1:158220291 | 1 | 1 | 1 | rs3181030 | 1:158220291_T_C |
| qian_2016_study22 | Qian (2016) | rs10762744 | C | 10:78734988 | 1 | 1 | 1 | rs10762744 | 10:78734988_T_C |
| qian_2016_study22 | Qian (2016) | rs62405489 | C | 5:179904861 | 1 | 1 | 1 | rs62405489 | 5:179904861_T_C |
| qian_2016_study22 | Qian (2016) | rs56832849 | T | 7:21609625 | 1 | 1 | 1 | rs56832849 | 7:21609625_C_T |
| qian_2016_study22 | Qian (2016) | rs117666100 | A | 7:110192014 | 1 | 1 | 1 | rs117666100 | 7:110192014_G_A |
| qian_2016_study22 | Qian (2016) | rs142031269 | C | 11:42240713 | 1 | 1 | 1 | rs142031269 | 11:42240713_G_C |
| qian_2016_study22 | Qian (2016) | rs73736221 | A | 6:26380473 | 1 | 1 | 1 | rs73736221 | 6:26380473_C_A |
| qian_2016_study22 | Qian (2016) | rs6049387 | G | 20:24075174 | 1 | 1 | 1 | rs6049387 | 20:24075174_A_G |
| qian_2016_study22 | Qian (2016) | rs783541 | C | 15:83255061 | 1 | 1 | 1 | rs783541 | 15:83255061_T_C |
| qian_2016_study22 | Qian (2016) | rs62448750 | C | 7:14864330 | 1 | 1 | 1 | rs62448750 | 7:14864330_T_C |
| qian_2016_study22 | Qian (2016) | rs9514878 | G | 13:109341054 | 1 | 1 | 1 | rs9514878 | 13:109341054_A_G |
| qian_2016_study22 | Qian (2016) | rs3027095 | A | 13:78471025 | 1 | 1 | 1 | rs3027095 | 13:78471025_G_A |
| qian_2016_study22 | Qian (2016) | rs11026783 | G | 11:22792812 | 1 | 1 | 1 | rs11026783 | 11:22792812_G_A |
| qian_2016_study22 | Qian (2016) | rs4857900 | A | 3:128227662 | 1 | 1 | 1 | rs4857900 | 3:128227662_G_A |
| qian_2016_study22 | Qian (2016) | rs9533840 | A | 13:44969719 | 1 | 1 | 1 | rs9533840 | 13:44969719_G_A |
| qian_2016_study22 | Qian (2016) | rs7309654 | T | 12:2965808 | 1 | 1 | 1 | rs7309654 | 12:2965808_C_T |
| qian_2016_study22 | Qian (2016) | rs10780526 | T | 9:84262437 | 1 | 1 | 1 | rs10780526 | 9:84262437_A_T |
| qian_2016_study22 | Qian (2016) | rs62432748 | G | 6:154839209 | 1 | 1 | 1 | rs62432748 | 6:154839209_A_G |
| qian_2016_study22 | Qian (2016) | rs10092830 | G | 8:72969723 | 1 | 1 | 1 | rs10092830 | 8:72969723_C_G |
| qian_2016_study22 | Qian (2016) | rs11233812 | A | 11:83635347 | 1 | 1 | 1 | rs11233812 | 11:83635347_C_A |
| qian_2016_study22 | Qian (2016) | rs10746190 | C | 12:81901318 | 1 | 1 | 1 | rs10746190 | 12:81901318_C_A |
| qian_2016_study22 | Qian (2016) | rs78744080 | T | 4:168251992 | 1 | 1 | 1 | rs78744080 | rs78744080 |
| qian_2016_study22 | Qian (2016) | rs4945453 | T | 11:80619752 | 1 | 1 | 1 | rs4945453 | 11:80619752_G_T |
| qian_2016_study22 | Qian (2016) | rs56109029 | A | 9:87242762 | 1 | 1 | 1 | rs56109029 | 9:87242762_G_A |
| qian_2016_study22 | Qian (2016) | rs17709789 | T | 15:53246617 | 1 | 1 | 1 | rs17709789 | rs17709789 |
| qian_2016_study22 | Qian (2016) | rs1992578 | A | 4:107631471 | 1 | 1 | 1 | rs1992578 | 4:107631471_A_C |
| qian_2016_study22 | Qian (2016) | rs9652677 | A | 16:77773410 | 1 | 1 | 1 | rs9652677 | 16:77773410_A_C |
| qian_2016_study22 | Qian (2016) | rs73617443 | T | 6:151622137 | 1 | 1 | 1 | rs73617443 | rs73617443 |
| qian_2016_study22 | Qian (2016) | rs7104262 | A | 11:130951599 | 1 | 1 | 1 | rs7104262 | 11:130951599_T_A |
| qian_2016_study22 | Qian (2016) | rs3773494 | G | 3:71012580 | 1 | 1 | 1 | rs3773494 | rs3773494 |
| qian_2016_study22 | Qian (2016) | rs2249218 | A | 1:15712885 | 1 | 1 | 1 | rs2249218 | 1:15712885_C_A |
| qian_2016_study22 | Qian (2016) | rs10267303 | T | 7:70082913 | 1 | 1 | 1 | rs10267303 | 7:70082913_C_T |
| qian_2016_study22 | Qian (2016) | rs845559 | T | 7:55247960 | 1 | 1 | 1 | rs845559 | 7:55247960_C_T |
| qian_2016_study22 | Qian (2016) | rs476478 | G | 11:90217059 | 1 | 1 | 1 | rs476478 | 11:90217059_T_G |
| qian_2016_study22 | Qian (2016) | rs6490024 | G | 12:115980907 | 1 | 1 | 1 | rs6490024 | 12:115980907_G_A |
| qian_2016_study22 | Qian (2016) | rs2206889 | A | 20:43886487 | 1 | 1 | 1 | rs2206889 | 20:43886487_C_A |
| qian_2016_study22 | Qian (2016) | rs12320018 | C | 12:93512993 | 1 | 1 | 1 | rs12320018 | 12:93512993_T_C |
| qian_2016_study22 | Qian (2016) | rs62449807 | G | 7:24630401 | 1 | 1 | 1 | rs62449807 | rs62449807 |
| qian_2016_study22 | Qian (2016) | rs638141 | G | 6:161361888 | 1 | 1 | 1 | rs638141 | 6:161361888_A_G |
| qian_2016_study22 | Qian (2016) | rs17229339 | G | 21:38802669 | 1 | 1 | 1 | rs17229339 | 21:38802669_C_G |
| qian_2016_study22 | Qian (2016) | rs174532 | A | 11:61548874 | 1 | 1 | 1 | rs174532 | rs174532 |
| qian_2016_study22 | Qian (2016) | rs7330155 | T | 13:102533398 | 1 | 1 | 1 | rs7330155 | 13:102533398_C_T |
| qian_2016_study22 | Qian (2016) | rs4970481 | C | 1:27068202 | 1 | 1 | 1 | rs4970481 | 1:27068202_C_T |
| qian_2016_study22 | Qian (2016) | rs79590234 | A | 3:35152960 | 1 | 1 | 1 | rs79590234 | 3:35152960_C_A |
| qian_2016_study22 | Qian (2016) | rs2560826 | T | 5:16578135 | 1 | 1 | 1 | rs2560826 | 5:16578135_T_C |
| qian_2016_study22 | Qian (2016) | rs113239581 | C | 6:1999494 | 1 | 1 | 1 | rs113239581 | 6:1999494_T_C |

## S9 List of Included SNPs

| PGS_name | Label | rsID | Effect allele | Position | rsID in UKB extract | position in UKB extract | found in UKB extract | rsID in UKB | Position in UKB |
|---|---|---|---|---|---|---|---|---|---|
| qian_2016_study22 | Qian (2016) | rs17128766 | T | 1:86506271 | 1 | 1 | 1 | rs17128766 | 1:86506271_C_T |
| qian_2016_study22 | Qian (2016) | rs113707946 | C | 15:42763370 | 1 | 1 | 1 | rs113707946 | 15:42763370_A_C |
| qian_2016_study22 | Qian (2016) | rs75381291 | T | 16:80161774 | 1 | 1 | 1 | rs75381291 | 16:80161774_G_T |
| qian_2016_study22 | Qian (2016) | rs661407 | A | 1:112376191 | 1 | 1 | 1 | rs661407 | 1:112376191_A_G |
| qian_2016_study22 | Qian (2016) | rs9295663 | C | 6:25486800 | 1 | 1 | 1 | rs9295663 | 6:25486800_T_C |
| qian_2016_study22 | Qian (2016) | rs78649693 | G | 4:12898434 | 1 | 1 | 1 | rs78649693 | 4:12898434_T_G |
| qian_2016_study22 | Qian (2016) | rs59492146 | A | 18:49891603 | 1 | 1 | 1 | rs59492146 | 18:49891603_T_A |
| qian_2016_study22 | Qian (2016) | rs73196614 | G | 12:89929892 | 1 | 1 | 1 | rs73196614 | 12:89929892_T_G |
| qian_2016_study22 | Qian (2016) | rs150034466 | G | 9:19332507 | 1 | 1 | 1 | rs150034466 | 9:19332507_C_G |
| qian_2016_study22 | Qian (2016) | rs4703950 | A | 5:82626018 | 1 | 1 | 1 | rs4703950 | rs4703950 |
| qian_2016_study22 | Qian (2016) | rs77795102 | C | 4:185363639 | 1 | 1 | 1 | rs77795102 | 4:185363639_A_C |
| qian_2016_study22 | Qian (2016) | rs72736486 | C | 5:17190003 | 1 | 1 | 1 | rs72736486 | 5:17190003_T_C |
| qian_2016_study22 | Qian (2016) | rs116250377 | T | 5:8372303 | 1 | 1 | 1 | rs116250377 | 5:8372303_C_T |
| qian_2016_study22 | Qian (2016) | rs2238067 | A | 12:2453337 | 1 | 1 | 1 | rs2238067 | 12:2453337_C_A |
| qian_2016_study22 | Qian (2016) | rs2369008 | T | 16:23560204 | 1 | 1 | 1 | rs2369008 | 16:23560204_T_C |
| qian_2016_study22 | Qian (2016) | rs7574750 | A | 2:101755235 | 1 | 1 | 1 | rs7574750 | 2:101755235_C_A |
| qian_2016_study22 | Qian (2016) | rs7037276 | C | 9:6247430 | 1 | 1 | 1 | rs7037276 | 9:6247430_C_T |
| qian_2016_study22 | Qian (2016) | rs2404149 | C | 5:78697464 | 1 | 1 | 1 | rs2404149 | 5:78697464_G_C |
| qian_2016_study22 | Qian (2016) | rs112740214 | C | 13:105609643 | 1 | 1 | 1 | rs112740214 | 13:105609643_T_C |
| qian_2016_study22 | Qian (2016) | rs6517614 | T | 21:42150494 | 1 | 1 | 1 | rs6517614 | 21:42150494_A_T |
| qian_2016_study22 | Qian (2016) | rs12470543 | A | 2:88437314 | 1 | 1 | 1 | rs12470543 | 2:88437314_C_A |
| qian_2016_study22 | Qian (2016) | rs148036156 | A | 17:77314894 | 1 | 1 | 1 | rs148036156 | 17:77314894_G_A |
| qian_2016_study22 | Qian (2016) | rs34020075 | A | 13:67900106 | 1 | 1 | 1 | rs34020075 | 13:67900106_G_A |
| qian_2016_study22 | Qian (2016) | rs4696958 | T | 4:20515850 | 1 | 1 | 1 | rs4696958 | rs4696958 |
| qian_2016_study22 | Qian (2016) | rs2786494 | G | 1:98064334 | 1 | 1 | 1 | rs2786494 | 1:98064334_G_A |
| qian_2016_study22 | Qian (2016) | rs685725 | T | 11:105754802 | 1 | 1 | 1 | rs685725 | 11:105754802_C_T |
| qian_2016_study22 | Qian (2016) | rs117839973 | A | 9:3913844 | 1 | 1 | 1 | rs117839973 | 9:3913844_G_A |
| qian_2016_study22 | Qian (2016) | rs2821254 | A | 1:72557858 | 1 | 1 | 1 | rs2821254 | 1:72557858_G_A |
| qian_2016_study22 | Qian (2016) | rs6881653 | T | 5:65961733 | 1 | 1 | 1 | rs6881653 | 5:65961733_T_C |
| qian_2016_study22 | Qian (2016) | rs9365544 | C | 6:163607456 | 1 | 1 | 1 | rs9365544 | 6:163607456_C_T |
| qian_2016_study22 | Qian (2016) | rs75218693 | T | 1:16593404 | 1 | 1 | 1 | rs75218693 | 1:16593404_C_T |
| qian_2016_study22 | Qian (2016) | rs4975572 | T | 5:1054197 | 1 | 1 | 1 | rs4975572 | rs4975572 |
| qian_2016_study22 | Qian (2016) | rs2962038 | T | 5:1512868 | 1 | 1 | 1 | rs2962038 | 5:1512868_T_C |
| qian_2016_study22 | Qian (2016) | rs77046884 | T | 8:97997158 | 1 | 1 | 1 | rs77046884 | 8:97997158_C_T |
| qian_2016_study22 | Qian (2016) | rs7254296 | G | 19:17600547 | 1 | 1 | 1 | rs7254296 | 19:17600547_G_A |
| qian_2016_study22 | Qian (2016) | rs11771703 | C | 7:132095581 | 1 | 1 | 1 | rs11771703 | 7:132095581_C_A |
| qian_2016_study22 | Qian (2016) | rs66666940 | C | 3:98624077 | 1 | 1 | 1 | rs66666940 | 3:98624077_T_C |
| qian_2016_study22 | Qian (2016) | rs10512913 | C | 3:133539574 | 1 | 1 | 1 | rs10512913 | 3:133539574_A_C |
| qian_2016_study22 | Qian (2016) | rs77572938 | T | 13:33640673 | 1 | 1 | 1 | rs77572938 | 13:33640673_G_T |
| qian_2016_study22 | Qian (2016) | rs56309599 | T | 10:115352263 | 1 | 1 | 1 | rs56309599 | 10:115352263_C_T |
| qian_2016_study22 | Qian (2016) | rs74886612 | A | 9:135732186 | 1 | 1 | 1 | rs74886612 | 9:135732186_G_A |
| qian_2016_study22 | Qian (2016) | rs138281596 | C | 14:86544961 | 0 | 1 | 1 | rs28851821 | 14:86544961_T_C |
| qian_2016_study22 | Qian (2016) | rs74006937 | G | 15:27672263 | 1 | 1 | 1 | rs74006937 | 15:27672263_A_G |
| qian_2016_study22 | Qian (2016) | rs55697235 | A | 16:71493214 | 1 | 1 | 1 | rs55697235 | rs55697235 |
| qian_2016_study22 | Qian (2016) | rs10492332 | A | 12:114574726 | 1 | 1 | 1 | rs10492332 | 12:114574726_G_A |
| qian_2016_study22 | Qian (2016) | rs6062621 | C | 20:62683265 | 1 | 1 | 1 | rs6062621 | 20:62683265_G_C |
| qian_2016_study22 | Qian (2016) | rs73448548 | A | 11:21166598 | 1 | 1 | 1 | rs73448548 | 11:21166598_G_A |
| qian_2016_study22 | Qian (2016) | rs72827468 | T | 6:23347236 | 1 | 1 | 1 | rs72827468 | 6:23347236_C_T |
| qian_2016_study22 | Qian (2016) | rs9896511 | T | 17:4336762 | 1 | 1 | 1 | rs9896511 | 17:4336762_T_C |
| qian_2016_study22 | Qian (2016) | rs6837483 | T | 4:90863959 | 1 | 1 | 1 | rs6837483 | 4:90863959_G_T |
| qian_2016_study22 | Qian (2016) | rs6744770 | T | 2:175457926 | 1 | 1 | 1 | rs6744770 | 2:175457926_C_T |
| qian_2016_study22 | Qian (2016) | rs255832 | C | 5:168947145 | 1 | 1 | 1 | rs255832 | 5:168947145_C_A |
| qian_2016_study22 | Qian (2016) | rs11899850 | C | 2:212421401 | 1 | 1 | 1 | rs11899850 | 2:212421401_A_C |
| qian_2016_study22 | Qian (2016) | rs62056316 | A | 16:30458692 | 1 | 1 | 1 | rs62056316 | 16:30458692_G_A |
| qian_2016_study22 | Qian (2016) | rs6433497 | G | 2:175478692 | 1 | 1 | 1 | rs6433497 | 2:175478692_G_A |
| qian_2016_study22 | Qian (2016) | rs7571614 | C | 2:69246868 | 1 | 1 | 1 | rs7571614 | 2:69246868_T_C |
| qian_2016_study22 | Qian (2016) | rs4890303 | C | 18:43421439 | 1 | 1 | 1 | rs4890303 | 18:43421439_C_T |
| qian_2016_study22 | Qian (2016) | rs12765929 | T | 10:88682555 | 1 | 1 | 1 | rs12765929 | rs12765929 |
| qian_2016_study22 | Qian (2016) | rs34225521 | G | 3:179488706 | 1 | 1 | 1 | rs34225521 | 3:179488706_T_G |
| qian_2016_study22 | Qian (2016) | rs11136797 | A | 8:4786522 | 1 | 1 | 1 | rs11136797 | 8:4786522_G_A |
| qian_2016_study22 | Qian (2016) | rs7650518 | G | 3:107994456 | 1 | 1 | 1 | rs7650518 | 3:107994456_G_A |
| qian_2016_study22 | Qian (2016) | rs4944065 | G | 11:74763261 | 1 | 1 | 1 | rs4944065 | 11:74763261_A_G |
| qian_2016_study22 | Qian (2016) | rs2549146 | A | 16:82858144 | 1 | 1 | 1 | rs2549146 | 16:82858144_G_A |
| qian_2016_study22 | Qian (2016) | rs6422514 | T | 1:93032520 | 1 | 1 | 1 | rs6422514 | 1:93032520_T_C |
| qian_2016_study22 | Qian (2016) | rs11241667 | C | 5:122295037 | 1 | 1 | 1 | rs11241667 | 5:122295037_T_C |

## S9 List of Included SNPs

| PGS_name | Label | rsID | Effect allele | Position | rsID in UKB extract | position in UKB extract | found in UKB extract | rsID in UKB | Position in UKB |
|---|---|---|---|---|---|---|---|---|---|
| qian_2016_study22 | Qian (2016) | rs6975275 | G | 7:94681177 | 1 | 1 | 1 | rs6975275 | 7:94681177_A_G |
| qian_2016_study22 | Qian (2016) | rs1888471 | T | 21:39903923 | 1 | 1 | 1 | rs1888471 | 21:39903923_T_C |
| qian_2016_study22 | Qian (2016) | rs182738418 | G | 5:87608960 | 1 | 1 | 1 | rs182738418 | 5:87608960_C_G |
| shi_2023_study_27_A | Shi (2023a) | rs2736100 | A | 5:1286516 | 1 | 1 | 1 | rs2736100 | rs2736100 |
| shi_2023_study_27_A | Shi (2023a) | rs11196089 | C | 10:114509290 | 1 | 1 | 1 | rs11196089 | rs11196089 |
| shi_2023_study_27_A | Shi (2023a) | rs55779747 | C | 3:189354127 | 1 | 1 | 1 | rs55779747 | 3:189354127_A_C |
| shi_2023_study_27_A | Shi (2023a) | rs6937083 | T | 6:117785308 | 1 | 1 | 1 | rs6937083 | 6:117785308_A_T |
| shi_2023_study_27_A | Shi (2023a) | rs2293607 | C | 3:169482335 | 1 | 1 | 1 | rs2293607 | 3:169482335_T_C |
| shi_2023_study_27_A | Shi (2023a) | rs9367106 | C | 6:41483390 | 1 | 1 | 1 | rs9367106 | 6:41483390_G_C |
| shi_2023_study_27_A | Shi (2023a) | rs13167280 | A | 5:1280477 | 1 | 1 | 1 | rs13167280 | 5:1280477_G_A |
| shi_2023_study_27_A | Shi (2023a) | rs2760995 | A | 6:32574358 | 1 | 1 | 1 | rs2760995 | 6:32574358_G_A |
| shi_2023_study_27_A | Shi (2023a) | rs59956089 | C | 17:65960854 | 1 | 1 | 1 | rs59956089 | 17:65960854_T_C |
| shi_2023_study_27_A | Shi (2023a) | rs7962469 | G | 12:52348259 | 1 | 1 | 1 | rs7962469 | 12:52348259_A_G |
| shi_2023_study_27_A | Shi (2023a) | rs72658409 | T | 9:22160087 | 1 | 1 | 1 | rs72658409 | rs72658409 |
| shi_2023_study_27_A | Shi (2023a) | rs62332591 | G | 5:1290319 | 1 | 1 | 1 | rs62332591 | 5:1290319_T_G |
| shi_2023_study_27_A | Shi (2023a) | rs9380190 | C | 6:30769565 | 1 | 1 | 1 | rs9380190 | 6:30769565_T_C |
| shi_2023_study_27_A | Shi (2023a) | rs55768116 | A | 11:118108331 | 1 | 1 | 1 | rs55768116 | 11:118108331_C_A |
| shi_2023_study_27_A | Shi (2023a) | rs17038564 | G | 2:65496058 | 1 | 1 | 1 | rs17038564 | 2:65496058_A_G |
| shi_2023_study_27_A | Shi (2023a) | rs1200399 | T | 14:35293185 | 1 | 1 | 1 | rs1200399 | rs1200399 |
| shi_2023_study_27_A | Shi (2023a) | rs71467682 | G | 15:49757466 | 1 | 1 | 1 | rs71467682 | 15:49757466_A_G |
| shi_2023_study_27_A | Shi (2023a) | rs137884934 | T | 3:138570011 | 1 | 1 | 1 | rs137884934 | 3:138570011_C_T |
| shi_2023_study_27_A | Shi (2023a) | rs682888 | C | 2:25757709 | 1 | 1 | 1 | rs682888 | 2:25757709_T_C |
| shi_2023_study_27_A | Shi (2023a) | rs174559 | A | 11:61581656 | 1 | 1 | 1 | rs174559 | 11:61581656_G_A |
| shi_2023_study_27_A | Shi (2023a) | rs10901793 | A | 10:126324209 | 1 | 1 | 1 | rs10901793 | 10:126324209_G_A |
| shi_2023_study_27_A | Shi (2023a) | rs12664490 | T | 6:41483960 | 1 | 1 | 1 | rs12664490 | 6:41483960_C_T |
| shi_2023_study_27_A | Shi (2023a) | rs4268071 | T | 7:124373384 | 1 | 1 | 1 | rs4268071 | 7:124373384_T_G |
| shi_2023_study_27_A | Shi (2023a) | rs531557 | T | 6:53389995 | 1 | 1 | 1 | rs531557 | 6:53389995_A_T |
| shi_2023_study_27_A | Shi (2023a) | rs116863980 | A | 19:725066 | 1 | 1 | 1 | rs116863980 | 19:725066_G_A |
| shi_2023_study_27_A | Shi (2023a) | rs764014 | G | 15:56454223 | 1 | 1 | 1 | rs764014 | 15:56454223_A_G |
| shi_2023_study_27_A | Shi (2023a) | rs117715768 | T | 4:44174404 | 1 | 1 | 1 | rs117715768 | 4:44174404_C_T |
| shi_2023_study_27_A | Shi (2023a) | rs1373058 | A | 4:157894892 | 1 | 1 | 1 | rs1373058 | 4:157894892_T_A |
| shi_2023_study_27_B | Shi (2023b) | rs2736100 | A | 5:1286516 | 1 | 1 | 1 | rs2736100 | rs2736100 |
| shi_2023_study_27_B | Shi (2023b) | rs11196089 | C | 10:114509290 | 1 | 1 | 1 | rs11196089 | rs11196089 |
| shi_2023_study_27_B | Shi (2023b) | rs55779747 | C | 3:189354127 | 1 | 1 | 1 | rs55779747 | 3:189354127_A_C |
| shi_2023_study_27_B | Shi (2023b) | rs6937083 | T | 6:117785308 | 1 | 1 | 1 | rs6937083 | 6:117785308_A_T |
| shi_2023_study_27_B | Shi (2023b) | rs9367106 | C | 6:41483390 | 1 | 1 | 1 | rs9367106 | 6:41483390_G_C |
| shi_2023_study_27_B | Shi (2023b) | rs2760995 | A | 6:32574358 | 1 | 1 | 1 | rs2760995 | 6:32574358_G_A |
| shi_2023_study_27_B | Shi (2023b) | rs59956089 | C | 17:65960854 | 1 | 1 | 1 | rs59956089 | 17:65960854_T_C |
| shi_2023_study_27_B | Shi (2023b) | rs7962469 | G | 12:52348259 | 1 | 1 | 1 | rs7962469 | 12:52348259_A_G |
| shi_2023_study_27_B | Shi (2023b) | rs72658409 | T | 9:22160087 | 1 | 1 | 1 | rs72658409 | rs72658409 |
| shi_2023_study_27_B | Shi (2023b) | rs9380190 | C | 6:30769565 | 1 | 1 | 1 | rs9380190 | 6:30769565_T_C |
| shi_2023_study_27_B | Shi (2023b) | rs55768116 | A | 11:118108331 | 1 | 1 | 1 | rs55768116 | 11:118108331_C_A |
| shi_2023_study_27_B | Shi (2023b) | rs71467682 | G | 15:49757466 | 1 | 1 | 1 | rs71467682 | 15:49757466_A_G |
| shi_2023_study_27_B | Shi (2023b) | rs137884934 | T | 3:138570011 | 1 | 1 | 1 | rs137884934 | 3:138570011_C_T |
| shi_2023_study_27_B | Shi (2023b) | rs682888 | C | 2:25757709 | 1 | 1 | 1 | rs682888 | 2:25757709_T_C |
| shi_2023_study_27_B | Shi (2023b) | rs174559 | A | 11:61581656 | 1 | 1 | 1 | rs174559 | 11:61581656_G_A |
| shi_2023_study_27_B | Shi (2023b) | rs10901793 | A | 10:126324209 | 1 | 1 | 1 | rs10901793 | 10:126324209_G_A |
| shi_2023_study_27_B | Shi (2023b) | rs4268071 | T | 7:124373384 | 1 | 1 | 1 | rs4268071 | 7:124373384_T_G |
| shi_2023_study_27_B | Shi (2023b) | rs531557 | T | 6:53389995 | 1 | 1 | 1 | rs531557 | 6:53389995_A_T |
| shi_2023_study_27_B | Shi (2023b) | rs116863980 | A | 19:725066 | 1 | 1 | 1 | rs116863980 | 19:725066_G_A |
| shi_2023_study_27_B | Shi (2023b) | rs764014 | G | 15:56454223 | 1 | 1 | 1 | rs764014 | 15:56454223_A_G |
| shi_2023_study_27_B | Shi (2023b) | rs117715768 | T | 4:44174404 | 1 | 1 | 1 | rs117715768 | 4:44174404_C_T |
| shi_2023_study_27_B | Shi (2023b) | rs1373058 | A | 4:157894892 | 1 | 1 | 1 | rs1373058 | 4:157894892_T_A |
| shi_2023_study_27_C | Shi (2023c) | rs2736100 | A | 5:1286516 | 1 | 1 | 1 | rs2736100 | rs2736100 |
| shi_2023_study_27_C | Shi (2023c) | rs11196089 | C | 10:114509290 | 1 | 1 | 1 | rs11196089 | rs11196089 |
| shi_2023_study_27_C | Shi (2023c) | rs55779747 | C | 3:189354127 | 1 | 1 | 1 | rs55779747 | 3:189354127_A_C |
| shi_2023_study_27_C | Shi (2023c) | rs6937083 | T | 6:117785308 | 1 | 1 | 1 | rs6937083 | 6:117785308_A_T |
| shi_2023_study_27_C | Shi (2023c) | rs9367106 | C | 6:41483390 | 1 | 1 | 1 | rs9367106 | 6:41483390_G_C |
| shi_2023_study_27_C | Shi (2023c) | rs2760995 | A | 6:32574358 | 1 | 1 | 1 | rs2760995 | 6:32574358_G_A |
| shi_2023_study_27_C | Shi (2023c) | rs59956089 | C | 17:65960854 | 1 | 1 | 1 | rs59956089 | 17:65960854_T_C |
| shi_2023_study_27_C | Shi (2023c) | rs7962469 | G | 12:52348259 | 1 | 1 | 1 | rs7962469 | 12:52348259_A_G |
| shi_2023_study_27_C | Shi (2023c) | rs72658409 | T | 9:22160087 | 1 | 1 | 1 | rs72658409 | rs72658409 |
| shi_2023_study_27_C | Shi (2023c) | rs9380190 | C | 6:30769565 | 1 | 1 | 1 | rs9380190 | 6:30769565_T_C |
| shi_2023_study_27_C | Shi (2023c) | rs55768116 | A | 11:118108331 | 1 | 1 | 1 | rs55768116 | 11:118108331_C_A |
| shi_2023_study_27_C | Shi (2023c) | rs71467682 | G | 15:49757466 | 1 | 1 | 1 | rs71467682 | 15:49757466_A_G |

## S9 List of Included SNPs

| PGS_name | Label | rsID | Effect allele | Position | rsID in UKB extract | position in UKB extract | found in UKB extract | rsID in UKB | Position in UKB |
|---|---|---|---|---|---|---|---|---|---|
| shi_2023_study_27_C | Shi (2023c) | rs137884934 | T | 3:138570011 | 1 | 1 | 1 | rs137884934 | 3:138570011_C_T |
| shi_2023_study_27_C | Shi (2023c) | rs682888 | C | 2:25757709 | 1 | 1 | 1 | rs682888 | 2:25757709_T_C |
| shi_2023_study_27_C | Shi (2023c) | rs174559 | A | 11:61581656 | 1 | 1 | 1 | rs174559 | 11:61581656_G_A |
| shi_2023_study_27_C | Shi (2023c) | rs10901793 | A | 10:126324209 | 1 | 1 | 1 | rs10901793 | 10:126324209_G_A |
| shi_2023_study_27_C | Shi (2023c) | rs4268071 | T | 7:124373384 | 1 | 1 | 1 | rs4268071 | 7:124373384_T_G |
| shi_2023_study_27_C | Shi (2023c) | rs531557 | T | 6:53389995 | 1 | 1 | 1 | rs531557 | 6:53389995_A_T |
| shi_2023_study_27_C | Shi (2023c) | rs116863980 | A | 19:725066 | 1 | 1 | 1 | rs116863980 | 19:725066_G_A |
| shi_2023_study_27_C | Shi (2023c) | rs764014 | G | 15:56454223 | 1 | 1 | 1 | rs764014 | 15:56454223_A_G |
| shi_2023_study_27_C | Shi (2023c) | rs117715768 | T | 4:44174404 | 1 | 1 | 1 | rs117715768 | 4:44174404_C_T |
| shi_2023_study_27_C | Shi (2023c) | rs1373058 | A | 4:157894892 | 1 | 1 | 1 | rs1373058 | 4:157894892_T_A |
| wang_2021_study_38 | Wang (2021) | rs71658797 | T | 1:NA | 1 | 0 | 1 | NA | NA |
| wang_2021_study_38 | Wang (2021) | rs13080835 | G | 3:NA | 1 | 0 | 1 | NA | NA |
| wang_2021_study_38 | Wang (2021) | rs7705526 | C | 5:NA | 1 | 0 | 1 | NA | NA |
| wang_2021_study_38 | Wang (2021) | rs3094604 | A | 6:NA | 1 | 0 | 1 | NA | NA |
| wang_2021_study_38 | Wang (2021) | rs6920364 | G | 6:NA | 1 | 0 | 1 | NA | NA |
| wang_2021_study_38 | Wang (2021) | rs11780471 | G | 8:NA | 1 | 0 | 1 | NA | NA |
| wang_2021_study_38 | Wang (2021) | rs4236709 | A | 8:NA | 1 | 0 | 1 | NA | NA |
| wang_2021_study_38 | Wang (2021) | rs885518 | A | 9:NA | 1 | 0 | 1 | NA | NA |
| wang_2021_study_38 | Wang (2021) | rs11591710 | A | 10:NA | 1 | 0 | 1 | NA | NA |
| wang_2021_study_38 | Wang (2021) | rs1056562 | C | 11:NA | 1 | 0 | 1 | NA | NA |
| wang_2021_study_38 | Wang (2021) | rs7953330 | G | 12:NA | 1 | 0 | 1 | NA | NA |
| wang_2021_study_38 | Wang (2021) | rs11571833 | A | 13:NA | 1 | 0 | 1 | NA | NA |
| wang_2021_study_38 | Wang (2021) | rs55781567 | C | 15:NA | 1 | 0 | 1 | NA | NA |
| wang_2021_study_38 | Wang (2021) | rs77468143 | T | 15:NA | 1 | 0 | 1 | NA | NA |
| wang_2021_study_38 | Wang (2021) | rs66759488 | G | 15:NA | 1 | 0 | 1 | NA | NA |
| wang_2021_study_38 | Wang (2021) | rs56113850 | C | 19:NA | 1 | 0 | 1 | NA | NA |
| wang_2021_study_38 | Wang (2021) | rs41309931 | G | 20:NA | 1 | 0 | 1 | NA | NA |
| wang_2021_study_38 | Wang (2021) | rs17879961 | A | 22:NA | 1 | 0 | 1 | NA | NA |
| wang_2022_study_13 | L. Wang (2022) | rs10118776 | G | 9:6227418 | 1 | 1 | 1 | rs10118776 | 9:6227418_G_A |
| wang_2022_study_13 | L. Wang (2022) | rs10146472 | A | 14:33545815 | 0 | 0 | 0 | NA | NA |
| wang_2022_study_13 | L. Wang (2022) | rs10758203 | G | 9:33421422 | 0 | 0 | 0 | NA | NA |
| wang_2022_study_13 | L. Wang (2022) | rs1108581 | A | 9:133640119 | 0 | 0 | 0 | NA | NA |
| wang_2022_study_13 | L. Wang (2022) | rs111960002 | C | 8:143640250 | 1 | 0 | 1 | NA | NA |
| wang_2022_study_13 | L. Wang (2022) | rs114928225 | A | 2:118692164 | 1 | 0 | 1 | NA | NA |
| wang_2022_study_13 | L. Wang (2022) | rs114951367 | T | 6:32936274 | 0 | 0 | 0 | NA | NA |
| wang_2022_study_13 | L. Wang (2022) | rs11514963 | C | 7:131247904 | 0 | 0 | 0 | NA | NA |
| wang_2022_study_13 | L. Wang (2022) | rs115707823 | A | 6:30374976 | 0 | 0 | 0 | NA | NA |
| wang_2022_study_13 | L. Wang (2022) | rs115729734 | G | 6:29931238 | 0 | 0 | 0 | NA | NA |
| wang_2022_study_13 | L. Wang (2022) | rs11591710 | C | 10:103927874 | 1 | 0 | 1 | NA | NA |
| wang_2022_study_13 | L. Wang (2022) | rs116080888 | C | 6:32618459 | 0 | 0 | 0 | NA | NA |
| wang_2022_study_13 | L. Wang (2022) | rs116427960 | T | 6:31351449 | 0 | 0 | 0 | NA | NA |
| wang_2022_study_13 | L. Wang (2022) | rs116506680 | T | 6:29813272 | 0 | 0 | 0 | NA | NA |
| wang_2022_study_13 | L. Wang (2022) | rs116822326 | G | 6:31466334 | 0 | 0 | 0 | NA | NA |
| wang_2022_study_13 | L. Wang (2022) | rs117729306 | C | 11:8465623 | 0 | 0 | 0 | NA | NA |
| wang_2022_study_13 | L. Wang (2022) | rs11855650 | T | 15:70139434 | 1 | 0 | 1 | NA | NA |
| wang_2022_study_13 | L. Wang (2022) | rs12439944 | T | 15:74783309 | 0 | 0 | 0 | NA | NA |
| wang_2022_study_13 | L. Wang (2022) | rs12981718 | A | 19:54064604 | 1 | 0 | 1 | NA | NA |
| wang_2022_study_13 | L. Wang (2022) | rs13080835 | G | 3:189639410 | 1 | 0 | 1 | NA | NA |
| wang_2022_study_13 | L. Wang (2022) | rs13212562 | G | 6:27332531 | 0 | 0 | 0 | NA | NA |
| wang_2022_study_13 | L. Wang (2022) | rs1333040 | C | 9:22083405 | 1 | 0 | 1 | NA | NA |
| wang_2022_study_13 | L. Wang (2022) | rs144503462 | T | 1:243029970 | 0 | 0 | 0 | NA | NA |
| wang_2022_study_13 | L. Wang (2022) | rs1629083 | C | 11:118255861 | 1 | 0 | 1 | NA | NA |
| wang_2022_study_13 | L. Wang (2022) | rs17181550 | T | 17:72303817 | 1 | 0 | 1 | NA | NA |
| wang_2022_study_13 | L. Wang (2022) | rs17185553 | C | 9:17934122 | 1 | 0 | 1 | NA | NA |
| wang_2022_study_13 | L. Wang (2022) | rs17391694 | T | 1:78157942 | 0 | 0 | 0 | NA | NA |
| wang_2022_study_13 | L. Wang (2022) | rs190996050 | C | 5:1048492 | 0 | 0 | 0 | NA | NA |
| wang_2022_study_13 | L. Wang (2022) | rs191205566 | T | 9:99824951 | 1 | 0 | 1 | NA | NA |
| wang_2022_study_13 | L. Wang (2022) | rs2006209 | T | 19:41589873 | 0 | 0 | 0 | NA | NA |
| wang_2022_study_13 | L. Wang (2022) | rs2316515 | G | 6:410848 | 1 | 1 | 1 | rs2316515 | 6:410848_A_G |
| wang_2022_study_13 | L. Wang (2022) | rs2320614 | C | 4:163148970 | 0 | 0 | 0 | NA | NA |
| wang_2022_study_13 | L. Wang (2022) | rs2741348 | G | 8:27563265 | 0 | 0 | 0 | NA | NA |
| wang_2022_study_13 | L. Wang (2022) | rs28408315 | G | 15:78892838 | 1 | 0 | 1 | NA | NA |
| wang_2022_study_13 | L. Wang (2022) | rs28412066 | T | 6:25775342 | 0 | 0 | 0 | NA | NA |
| wang_2022_study_13 | L. Wang (2022) | rs35440884 | G | 9:105051392 | 0 | 0 | 0 | NA | NA |
| wang_2022_study_13 | L. Wang (2022) | rs35772018 | C | 5:150730677 | 0 | 0 | 0 | NA | NA |

## S9 List of Included SNPs

| PGS_name | Label | rsID | Effect allele | Position | rsID in UKB extract | position in UKB extract | found in UKB extract | rsID in UKB | Position in UKB |
|---|---|---|---|---|---|---|---|---|---|
| wang_2022_study_13 | L. Wang (2022) | rs3847711 | G | 12:58440252 | 0 | 0 | 0 | NA | NA |
| wang_2022_study_13 | L. Wang (2022) | rs3944100 | C | 7:17014722 | 0 | 0 | 0 | NA | NA |
| wang_2022_study_13 | L. Wang (2022) | rs3999544 | C | 6:26285639 | 1 | 0 | 1 | NA | NA |
| wang_2022_study_13 | L. Wang (2022) | rs4236709 | G | 8:32552592 | 1 | 0 | 1 | NA | NA |
| wang_2022_study_13 | L. Wang (2022) | rs467095 | T | 5:1336106 | 1 | 0 | 1 | NA | NA |
| wang_2022_study_13 | L. Wang (2022) | rs4886591 | A | 15:78782176 | 0 | 0 | 0 | NA | NA |
| wang_2022_study_13 | L. Wang (2022) | rs56113850 | C | 19:40847202 | 1 | 0 | 1 | NA | NA |
| wang_2022_study_13 | L. Wang (2022) | rs56404467 | A | 13:32265853 | 1 | 0 | 1 | NA | NA |
| wang_2022_study_13 | L. Wang (2022) | rs59396197 | G | 2:139675756 | 0 | 0 | 0 | NA | NA |
| wang_2022_study_13 | L. Wang (2022) | rs60280813 | C | 10:97913426 | 0 | 0 | 0 | NA | NA |
| wang_2022_study_13 | L. Wang (2022) | rs62560775 | G | 9:22052069 | 1 | 0 | 1 | NA | NA |
| wang_2022_study_13 | L. Wang (2022) | rs62621207 | T | 10:100912491 | 1 | 0 | 1 | NA | NA |
| wang_2022_study_13 | L. Wang (2022) | rs66500423 | C | 19:40689265 | 1 | 0 | 1 | NA | NA |
| wang_2022_study_13 | L. Wang (2022) | rs66759488 | A | 15:47285254 | 1 | 0 | 1 | NA | NA |
| wang_2022_study_13 | L. Wang (2022) | rs67824503 | T | 8:128523018 | 0 | 0 | 0 | NA | NA |
| wang_2022_study_13 | L. Wang (2022) | rs7182948 | G | 15:49549483 | 0 | 0 | 0 | NA | NA |
| wang_2022_study_13 | L. Wang (2022) | rs72981884 | G | 19:4840787 | 0 | 0 | 0 | NA | NA |
| wang_2022_study_13 | L. Wang (2022) | rs7299138 | T | 12:128586392 | 0 | 0 | 0 | NA | NA |
| wang_2022_study_13 | L. Wang (2022) | rs7383287 | G | 6:32815309 | 1 | 0 | 1 | NA | NA |
| wang_2022_study_13 | L. Wang (2022) | rs74501188 | G | 5:90931162 | 0 | 0 | 0 | NA | NA |
| wang_2022_study_13 | L. Wang (2022) | rs75685923 | T | 9:133410109 | 1 | 0 | 1 | NA | NA |
| wang_2022_study_13 | L. Wang (2022) | rs76980472 | C | 2:67567320 | 0 | 0 | 0 | NA | NA |
| wang_2022_study_13 | L. Wang (2022) | rs7705526 | A | 5:1285859 | 1 | 0 | 1 | NA | NA |
| wang_2022_study_13 | L. Wang (2022) | rs78062588 | T | 1:154593749 | 1 | 0 | 1 | NA | NA |
| wang_2022_study_13 | L. Wang (2022) | rs78154696 | A | 5:1000041 | 1 | 0 | 1 | NA | NA |
| wang_2022_study_13 | L. Wang (2022) | rs78334599 | G | 11:116128039 | 1 | 0 | 1 | NA | NA |
| wang_2022_study_13 | L. Wang (2022) | rs78442819 | G | 16:10647125 | 1 | 0 | 1 | NA | NA |
| wang_2022_study_13 | L. Wang (2022) | rs78853063 | C | 11:57482553 | 1 | 0 | 1 | NA | NA |
| wang_2022_study_13 | L. Wang (2022) | rs79368540 | T | 2:44962598 | 1 | 0 | 1 | NA | NA |
| wang_2022_study_13 | L. Wang (2022) | rs79421398 | C | 18:23161171 | 1 | 0 | 1 | NA | NA |
| wang_2022_study_13 | L. Wang (2022) | rs7953330 | G | 12:889653 | 1 | 0 | 1 | NA | NA |
| wang_2022_study_13 | L. Wang (2022) | rs8040868 | C | 15:78618839 | 1 | 0 | 1 | NA | NA |
| wang_2022_study_13 | L. Wang (2022) | rs9308062 | T | 4:163517048 | 0 | 0 | 0 | NA | NA |
| wang_2022_study_13 | L. Wang (2022) | rs9387479 | T | 6:117471449 | 0 | 0 | 0 | NA | NA |
| wang_2022_study_13 | L. Wang (2022) | rs9602270 | T | 13:83706928 | 1 | 0 | 1 | NA | NA |
| wang_2022_study_13 | L. Wang (2022) | rs9668978 | T | 12:64519457 | 1 | 0 | 1 | NA | NA |
| wang_2022_study_37 | X. Wang (2022) | rs71658797 | T | 1:65496058 | 1 | 0 | 1 | NA | NA |
| wang_2022_study_37 | X. Wang (2022) | rs13080835 | T | 3:169482335 | 1 | 1 | 1 | rs2293607 | 3:169482335_T_C |
| wang_2022_study_37 | X. Wang (2022) | rs7705526 | C | 5:1280477 | 1 | 1 | 1 | rs13167280 | 5:1280477_G_A |
| wang_2022_study_37 | X. Wang (2022) | rs116822326 | A | 6:1285974 | 0 | 0 | 0 | NA | NA |
| wang_2022_study_37 | X. Wang (2022) | rs6920364 | G | 6:1286516 | 1 | 0 | 1 | NA | NA |
| wang_2022_study_37 | X. Wang (2022) | rs11780471 | A | 8:1287194 | 1 | 0 | 1 | NA | NA |
| wang_2022_study_37 | X. Wang (2022) | rs4236709 | A | 8:32368087 | 1 | 0 | 1 | NA | NA |
| wang_2022_study_37 | X. Wang (2022) | rs885518 | A | 9:32433167 | 1 | 0 | 1 | NA | NA |
| wang_2022_study_37 | X. Wang (2022) | rs11591710 | A | 10:32410110 | 1 | 0 | 1 | NA | NA |
| wang_2022_study_37 | X. Wang (2022) | rs1056562 | C | 11:21787521 | 1 | 0 | 1 | NA | NA |
| wang_2022_study_37 | X. Wang (2022) | rs7953330 | C | 12:21830157 | 1 | 0 | 1 | NA | NA |
| wang_2022_study_37 | X. Wang (2022) | rs11571833 | A | 13:33422488 | 1 | 0 | 1 | NA | NA |
| wang_2022_study_37 | X. Wang (2022) | rs55781567 | C | 15:105687632 | 1 | 0 | 1 | NA | NA |
| wang_2022_study_37 | X. Wang (2022) | rs77468143 | G | 15:118108331 | 1 | 0 | 1 | NA | NA |
| wang_2022_study_37 | X. Wang (2022) | rs66759488 | G | 15:118125625 | 1 | 0 | 1 | NA | NA |
| wang_2022_study_37 | X. Wang (2022) | rs56113850 | T | 19:35293185 | 1 | 0 | 1 | NA | NA |
| wang_2022_study_37 | X. Wang (2022) | rs41309931 | G | 20:49376624 | 1 | 0 | 1 | NA | NA |
| wang_2022_study_37 | X. Wang (2022) | rs17879961 | G | 22:62326579 | 1 | 0 | 1 | NA | NA |
| xiao_2023_study_15 | Xiao (2023) | rs11780471 | A | 8:27344719 | 1 | 1 | 1 | rs11780471 | 8:27344719_G_A |
| xiao_2023_study_15 | Xiao (2023) | rs148696809 | C | 6:28934352 | 1 | 1 | 1 | rs148696809 | 6:28934352_T_C |
| xiao_2023_study_15 | Xiao (2023) | rs149543464 | A | 6:30400763 | 1 | 1 | 1 | rs149543464 | 6:30400763_G_A |
| xiao_2023_study_15 | Xiao (2023) | rs149866169 | A | 6:27441723 | 1 | 1 | 1 | rs149866169 | 6:27441723_T_A |
| xiao_2023_study_15 | Xiao (2023) | rs1629083 | C | 11:118126576 | 1 | 1 | 1 | rs1629083 | 11:118126576_C_T |
| xiao_2023_study_15 | Xiao (2023) | rs1632908 | G | 6:29893403 | 1 | 1 | 1 | rs1632908 | 6:29893403_C_G |
| xiao_2023_study_15 | Xiao (2023) | rs239935 | G | 6:167411788 | 1 | 1 | 1 | rs239935 | 6:167411788_G_A |
| xiao_2023_study_15 | Xiao (2023) | rs2523573 | G | 6:31328988 | 1 | 1 | 1 | rs2523573 | 6:31328988_C_G |
| xiao_2023_study_15 | Xiao (2023) | rs28412066 | T | 6:25809751 | 0 | 0 | 0 | NA | NA |
| xiao_2023_study_15 | Xiao (2023) | rs34661125 | A | 6:28281894 | 1 | 1 | 1 | rs34661125 | 6:28281894_G_A |
| xiao_2023_study_15 | Xiao (2023) | rs380286 | A | 5:1320247 | 1 | 1 | 1 | rs380286 | 5:1320247_G_A |

## S9 List of Included SNPs

| PGS_name | Label | rsID | Effect allele | Position | rsID in UKB extract | position in UKB extract | found in UKB extract | rsID in UKB | Position in UKB |
|---|---|---|---|---|---|---|---|---|---|
| xiao_2023_study_15 | Xiao (2023) | rs3861180 | A | 15:79043393 | 1 | 1 | 1 | rs3861180 | 15:79043393_A_G |
| xiao_2023_study_15 | Xiao (2023) | rs501942 | T | 6:31840477 | 1 | 1 | 1 | rs501942 | 6:31840477_C_T |
| xiao_2023_study_15 | Xiao (2023) | rs66759488 | A | 15:47584440 | 1 | 0 | 1 | NA | NA |
| xiao_2023_study_15 | Xiao (2023) | rs7705526 | A | 5:1285974 | 1 | 1 | 1 | rs7705526 | 5:1285974_C_A |
| xiao_2023_study_15 | Xiao (2023) | rs77468143 | G | 15:49376624 | 1 | 1 | 1 | rs77468143 | 15:49376624_T_G |
| xiao_2023_study_15 | Xiao (2023) | rs7953330 | C | 12:998819 | 1 | 1 | 1 | rs7953330 | 12:998819_G_C |
| xiao_2023_study_15 | Xiao (2023) | rs80264589 | A | 6:26927602 | 1 | 1 | 1 | rs80264589 | 6:26927602_G_A |
| xiao_2023_study_15 | Xiao (2023) | rs9271633 | G | 6:32592072 | 1 | 1 | 1 | rs9271633 | 6:32592072_T_G |
| xin_2023_study34 | Xin (2023) | rs71658797 | A | 1:NA | 1 | 0 | 1 | NA | NA |
| xin_2023_study34 | Xin (2023) | rs13080835 | G | 3:NA | 1 | 0 | 1 | NA | NA |
| xin_2023_study34 | Xin (2023) | rs7705526 | A | 5:NA | 1 | 0 | 1 | NA | NA |
| xin_2023_study34 | Xin (2023) | rs3094604 | G | 6:NA | 1 | 0 | 1 | NA | NA |
| xin_2023_study34 | Xin (2023) | rs6920364 | C | 6:NA | 1 | 0 | 1 | NA | NA |
| xin_2023_study34 | Xin (2023) | rs11780471 | G | 8:NA | 1 | 0 | 1 | NA | NA |
| xin_2023_study34 | Xin (2023) | rs4236709 | G | 8:NA | 1 | 0 | 1 | NA | NA |
| xin_2023_study34 | Xin (2023) | rs885518 | G | 9:NA | 1 | 0 | 1 | NA | NA |
| xin_2023_study34 | Xin (2023) | rs11591710 | C | 10:NA | 1 | 0 | 1 | NA | NA |
| xin_2023_study34 | Xin (2023) | rs1056562 | T | 11:NA | 1 | 0 | 1 | NA | NA |
| xin_2023_study34 | Xin (2023) | rs7953330 | G | 12:NA | 1 | 0 | 1 | NA | NA |
| xin_2023_study34 | Xin (2023) | rs66759488 | A | 15:NA | 1 | 0 | 1 | NA | NA |
| xin_2023_study34 | Xin (2023) | rs77468143 | T | 15:NA | 1 | 0 | 1 | NA | NA |
| xin_2023_study34 | Xin (2023) | rs55781567 | G | 15:NA | 1 | 0 | 1 | NA | NA |
| xin_2023_study34 | Xin (2023) | rs56113850 | C | 19:NA | 1 | 0 | 1 | NA | NA |
| xin_2023_study34 | Xin (2023) | rs41309931 | T | 20:NA | 1 | 0 | 1 | NA | NA |
| yang_2016_study_31 | Cheng (2016) | rs17728461 | C | 22:30598552 | 1 | 1 | 1 | rs17728461 | rs17728461 |
| yang_2016_study_31 | Cheng (2016) | rs465498 | A | 5:1325803 | 1 | 1 | 1 | rs465498 | 5:1325803_A_G |
| yang_2016_study_31 | Cheng (2016) | rs753955 | A | 13:24293859 | 1 | 1 | 1 | rs753955 | rs753955 |
| yang_2016_study_31 | Cheng (2016) | rs2895680 | T | 5:146644115 | 1 | 1 | 1 | rs2895680 | 5:146644115_C_T |
| yang_2016_study_31 | Cheng (2016) | rs12296850 | A | 12:100820085 | 1 | 1 | 1 | rs12296850 | 12:100820085_A_G |
| yang_2016_study_31 | Cheng (2016) | rs4488809 | C | 3:189356261 | 1 | 1 | 1 | rs4488809 | rs4488809 |
| yang_2016_study_31 | Cheng (2016) | rs2736100 | A | 5:1286516 | 1 | 1 | 1 | rs2736100 | rs2736100 |
| yang_2016_study_31 | Cheng (2016) | rs9439519 | T | 1:5364634 | 1 | 1 | 1 | rs9439519 | 1:5364634_C_T |
| yang_2016_study_31 | Cheng (2016) | rs383362 | G | 16:79245820 | 1 | 1 | 1 | rs383362 | rs383362 |
| yang_2016_study_31 | Cheng (2016) | rs6573 | C | 1:112255389 | 1 | 1 | 1 | rs6573 | 1:112255389_C_A |
| yang_2016_study_31 | Cheng (2016) | rs247008 | G | 5:131447104 | 1 | 1 | 1 | rs247008 | 5:131447104_A_G |
| yang_2016_study_31 | Cheng (2016) | rs4809957 | G | 20:52771171 | 1 | 1 | 1 | rs4809957 | 20:52771171_A_G |
| yang_2016_study_31 | Cheng (2016) | rs4246215 | G | 11:61564299 | 1 | 1 | 1 | rs4246215 | rs4246215 |
| yang_2016_study_31 | Cheng (2016) | rs1663689 | T | 10:9025195 | 1 | 1 | 1 | rs1663689 | 10:9025195_T_C |
| yang_2016_study_31 | Cheng (2016) | rs7086803 | G | 10:114498476 | 1 | 1 | 1 | rs7086803 | rs7086803 |
| yang_2016_study_31 | Cheng (2016) | rs4083914 | G | 6:153427706 | 1 | 1 | 1 | rs4083914 | 6:153427706_C_G |
| yang_2016_study_31 | Cheng (2016) | rs2286455 | C | 4:16020162 | 1 | 1 | 1 | rs2286455 | rs2286455 |
| yang_2016_study_31 | Cheng (2016) | rs3764340 | C | 16:78466437 | 1 | 1 | 1 | rs3764340 | rs3764340 |
| yang_2016_study_31 | Cheng (2016) | rs36600 | C | 22:30337586 | 1 | 1 | 1 | rs36600 | rs36600 |
| yang_2016_study_31 | Cheng (2016) | rs842461 | T | 3:195535614 | 1 | 1 | 1 | rs842461 | 3:195535614_G_T |
| yang_2016_study_31 | Cheng (2016) | rs2285053 | C | 16:55512377 | 1 | 1 | 1 | rs2285053 | 16:55512377_C_T |
| yang_2016_study_31 | Cheng (2016) | rs2131877 | A | 3:194858374 | 1 | 1 | 1 | rs2131877 | rs2131877 |
| yang_2016_study_31 | Cheng (2016) | rs1801133 | G | 1:11856378 | 1 | 1 | 1 | rs1801133 | rs1801133 |
| yang_2016_study_31 | Cheng (2016) | rs3866958 | C | 17:19281006 | 1 | 1 | 1 | rs3866958 | 17:19281006_A_C |
| yang_2016_study_31 | Cheng (2016) | rs1800625 | A | 6:32152442 | 1 | 1 | 1 | rs1800625 | 6:32152442_A_G |
| yang_2016_study_31 | Cheng (2016) | rs9387478 | C | 6:117786180 | 1 | 1 | 1 | rs9387478 | rs9387478 |
| yang_2016_study_31 | Cheng (2016) | rs743572 | G | 10:104597152 | 1 | 1 | 1 | rs743572 | rs743572 |
| yang_2016_study_31 | Cheng (2016) | rs4291 | A | 17:61554194 | 1 | 1 | 1 | rs4291 | 17:61554194_T_A |
| yang_2016_study_31 | Cheng (2016) | rs10845498 | A | 12:12394574 | 1 | 1 | 1 | rs10845498 | 12:12394574_G_A |
| yang_2016_study_31 | Cheng (2016) | rs7326277 | T | 13:28876214 | 1 | 1 | 1 | rs7326277 | 13:28876214_T_C |
| yang_2016_study_31 | Cheng (2016) | rs931127 | G | 11:65405300 | 1 | 1 | 1 | rs931127 | 11:65405300_G_A |
| yang_2016_study_31 | Cheng (2016) | rs2016520 | T | 6:35378778 | 1 | 1 | 1 | rs2016520 | rs2016520 |
| yang_2016_study_31 | Cheng (2016) | rs25406 | G | 20:5099636 | 1 | 1 | 1 | rs25406 | rs25406 |
| yang_2016_study_31 | Cheng (2016) | rs2240688 | T | 4:15970349 | 1 | 1 | 1 | rs2240688 | rs2240688 |
| yang_2016_study_31 | Cheng (2016) | rs34843907 | G | 6:32610059 | 1 | 1 | 1 | rs34843907 | 6:32610059_T_G |
| yang_2016_study_31 | Cheng (2016) | rs2070600 | C | 6:32151443 | 1 | 1 | 1 | rs2070600 | rs2070600 |
| yang_2016_study_31 | Cheng (2016) | rs189037 | G | 11:108093833 | 1 | 1 | 1 | rs189037 | 11:108093833_G_A |
| yang_2016_study_31 | Cheng (2016) | rs3817963 | T | 6:32368087 | 1 | 1 | 1 | rs3817963 | rs3817963 |
| yu_2020_study_10 | Yu (2020) | rs71658797 | A | 1:77967507 | 1 | 1 | 1 | rs71658797 | 1:77967507_T_A |
| yu_2020_study_10 | Yu (2020) | rs11683501 | G | 2:184159036 | 1 | 1 | 1 | rs11683501 | 2:184159036_A_G |
| yu_2020_study_10 | Yu (2020) | rs13080835 | G | 3:189357199 | 1 | 1 | 1 | rs13080835 | 3:189357199_G_T |

## S9 List of Included SNPs

| PGS_name | Label | rsID | Effect allele | Position | rsID in UKB extract | position in UKB extract | found in UKB extract | rsID in UKB | Position in UKB |
|---|---|---|---|---|---|---|---|---|---|
| yu_2020_study_10 | Yu (2020) | rs2736100 | C | 5:1286516 | 1 | 1 | 1 | rs2736100 | rs2736100 |
| yu_2020_study_10 | Yu (2020) | rs2853677 | G | 5:1287194 | 1 | 1 | 1 | rs2853677 | rs2853677 |
| yu_2020_study_10 | Yu (2020) | rs31489 | C | 5:1342714 | 0 | 0 | 0 | NA | NA |
| yu_2020_study_10 | Yu (2020) | rs4635969 | G | 5:1308552 | 1 | 1 | 1 | rs4635969 | rs4635969 |
| yu_2020_study_10 | Yu (2020) | rs4975616 | A | 5:1315660 | 1 | 1 | 1 | rs4975616 | rs4975616 |
| yu_2020_study_10 | Yu (2020) | rs1150752 | C | 6:32064726 | 1 | 1 | 1 | rs1150752 | rs1150752 |
| yu_2020_study_10 | Yu (2020) | rs2523571 | A | 6:31329691 | 1 | 1 | 1 | rs2523571 | 6:31329691_T_A |
| yu_2020_study_10 | Yu (2020) | rs6920364 | C | 6:167376466 | 1 | 1 | 1 | rs6920364 | 6:167376466_G_C |
| yu_2020_study_10 | Yu (2020) | rs11780471 | G | 8:27344719 | 1 | 1 | 1 | rs11780471 | 8:27344719_G_A |
| yu_2020_study_10 | Yu (2020) | rs4236709 | G | 8:32410110 | 1 | 1 | 1 | rs4236709 | 8:32410110_G_A |
| yu_2020_study_10 | Yu (2020) | rs1333040 | C | 9:22083404 | 1 | 1 | 1 | rs1333040 | rs1333040 |
| yu_2020_study_10 | Yu (2020) | rs1537372 | G | 9:22103183 | 1 | 1 | 1 | rs1537372 | 9:22103183_G_T |
| yu_2020_study_10 | Yu (2020) | rs885518 | G | 9:21830157 | 1 | 1 | 1 | rs885518 | 9:21830157_A_G |
| yu_2020_study_10 | Yu (2020) | rs11591710 | C | 10:105687632 | 1 | 1 | 1 | rs11591710 | 10:105687632_A_C |
| yu_2020_study_10 | Yu (2020) | rs1056562 | T | 11:118125625 | 1 | 1 | 1 | rs1056562 | 11:118125625_T_C |
| yu_2020_study_10 | Yu (2020) | rs10849605 | C | 12:1064438 | 1 | 1 | 1 | rs10849605 | rs10849605 |
| yu_2020_study_10 | Yu (2020) | rs7953330 | G | 12:998819 | 1 | 1 | 1 | rs7953330 | 12:998819_G_C |
| yu_2020_study_10 | Yu (2020) | rs11571833 | T | 13:32972626 | 1 | 1 | 1 | rs11571833 | rs11571833 |
| yu_2020_study_10 | Yu (2020) | rs56084662 | A | 13:32869864 | 1 | 1 | 1 | rs56084662 | 13:32869864_G_A |
| yu_2020_study_10 | Yu (2020) | rs12914385 | T | 15:78898723 | 1 | 1 | 1 | rs12914385 | rs12914385 |
| yu_2020_study_10 | Yu (2020) | rs8042374 | A | 15:78908032 | 1 | 1 | 1 | rs8042374 | 15:78908032_A_G |
| yu_2020_study_10 | Yu (2020) | rs55781567 | G | 15:78857986 | 1 | 1 | 1 | rs55781567 | rs55781567 |
| yu_2020_study_10 | Yu (2020) | rs66759488 | A | 15:47577451 | 1 | 1 | 1 | rs66759488 | 15:47577451_G_A |
| yu_2020_study_10 | Yu (2020) | rs680244 | C | 15:78871288 | 1 | 1 | 1 | rs680244 | rs680244 |
| yu_2020_study_10 | Yu (2020) | rs77468143 | T | 15:49376624 | 1 | 1 | 1 | rs77468143 | 15:49376624_T_G |
| yu_2020_study_10 | Yu (2020) | rs56113850 | C | 19:41353107 | 1 | 1 | 1 | rs56113850 | 19:41353107_T_C |
| yu_2020_study_10 | Yu (2020) | rs41309931 | T | 20:62326579 | 1 | 1 | 1 | rs41309931 | 20:62326579_G_T |
| yu_2020_study_10 | Yu (2020) | rs17879961 | A | 22:29121087 | 1 | 1 | 1 | rs17879961 | rs17879961 |
| zhang_2023_study_40 | Zhang (2023) | rs71658797 | A | 1:77967507 | 1 | 1 | 1 | rs71658797 | 1:77967507_T_A |
| zhang_2023_study_40 | Zhang (2023) | rs13080835 | T | 3:189357199 | 1 | 1 | 1 | rs13080835 | 3:189357199_G_T |
| zhang_2023_study_40 | Zhang (2023) | rs7705526 | A | 5:1285974 | 1 | 1 | 1 | rs7705526 | 5:1285974_C_A |
| zhang_2023_study_40 | Zhang (2023) | rs6920364 | C | 6:167377165 | 1 | 0 | 1 | NA | NA |
| zhang_2023_study_40 | Zhang (2023) | rs116822326 | G | 6:31434111 | 0 | 1 | 1 | rs3094604 | 6:31434111_A_G |
| zhang_2023_study_40 | Zhang (2023) | rs11780471 | A | 8:27344719 | 1 | 1 | 1 | rs11780471 | 8:27344719_G_A |
| zhang_2023_study_40 | Zhang (2023) | rs4236709 | G | 8:32410110 | 1 | 1 | 1 | rs4236709 | 8:32410110_G_A |
| zhang_2023_study_40 | Zhang (2023) | rs885518 | G | 9:21830157 | 1 | 1 | 1 | rs885518 | 9:21830157_A_G |
| zhang_2023_study_40 | Zhang (2023) | rs11591710 | C | 10:105687632 | 1 | 1 | 1 | rs11591710 | 10:105687632_A_C |
| zhang_2023_study_40 | Zhang (2023) | rs1056562 | T | 11:118125625 | 1 | 1 | 1 | rs1056562 | 11:118125625_T_C |
| zhang_2023_study_40 | Zhang (2023) | rs7953330 | C | 12:998819 | 1 | 1 | 1 | rs7953330 | 12:998819_G_C |
| zhang_2023_study_40 | Zhang (2023) | rs11571833 | T | 13:32972626 | 1 | 1 | 1 | rs11571833 | rs11571833 |
| zhang_2023_study_40 | Zhang (2023) | rs55781567 | G | 15:78857986 | 1 | 1 | 1 | rs55781567 | rs55781567 |
| zhang_2023_study_40 | Zhang (2023) | rs66759488 | A | 15:47577451 | 1 | 1 | 1 | rs66759488 | 15:47577451_G_A |
| zhang_2023_study_40 | Zhang (2023) | rs77468143 | G | 15:49376624 | 1 | 1 | 1 | rs77468143 | 15:49376624_T_G |
| zhang_2023_study_40 | Zhang (2023) | rs56113850 | T | 19:41353107 | 1 | 1 | 1 | rs56113850 | 19:41353107_T_C |
| zhang_2023_study_40 | Zhang (2023) | rs41309931 | T | 20:62326579 | 1 | 1 | 1 | rs41309931 | 20:62326579_G_T |
| zhang_2023_study_40 | Zhang (2023) | rs17879961 | G | 22:29121087 | 1 | 1 | 1 | rs17879961 | rs17879961 |
| Zhang_R_2022 | R. Zhang (2022) | rs71658797 | A | 1:77967507 | 1 | 1 | 1 | rs71658797 | 1:77967507_T_A |
| Zhang_R_2022 | R. Zhang (2022) | rs13080835 | T | 3:189357199 | 1 | 1 | 1 | rs13080835 | 3:189357199_G_T |
| Zhang_R_2022 | R. Zhang (2022) | rs7705526 | A | 5:1285974 | 1 | 1 | 1 | rs7705526 | 5:1285974_C_A |
| Zhang_R_2022 | R. Zhang (2022) | rs112290073 | A | 5:1286032 | 1 | 1 | 1 | rs112290073 | 5:1286032_G_A |
| Zhang_R_2022 | R. Zhang (2022) | rs2736098 | T | 5:1294086 | 1 | 1 | 1 | rs2736098 | 5:1294086_C_T |
| Zhang_R_2022 | R. Zhang (2022) | rs2853668 | T | 5:1300025 | 1 | 1 | 1 | rs2853668 | 5:1300025_G_T |
| Zhang_R_2022 | R. Zhang (2022) | rs401681 | T | 5:1322087 | 1 | 1 | 1 | rs401681 | rs401681 |
| Zhang_R_2022 | R. Zhang (2022) | rs466502 | G | 5:1325767 | 1 | 1 | 1 | rs466502 | 5:1325767_A_G |
| Zhang_R_2022 | R. Zhang (2022) | rs6903823 | G | 6:28354519 | 0 | 0 | 0 | NA | NA |
| Zhang_R_2022 | R. Zhang (2022) | rs116822326 | G | 6:31434111 | 0 | 1 | 1 | rs3094604 | 6:31434111_A_G |
| Zhang_R_2022 | R. Zhang (2022) | rs2855812 | T | 6:31504943 | 0 | 0 | 0 | NA | NA |
| Zhang_R_2022 | R. Zhang (2022) | rs805262 | T | 6:31628733 | 1 | 1 | 1 | rs805262 | rs805262 |
| Zhang_R_2022 | R. Zhang (2022) | rs6916278 | A | 6:31678774 | 1 | 1 | 1 | rs6916278 | rs6916278 |
| Zhang_R_2022 | R. Zhang (2022) | rs3129763 | A | 6:32590925 | 1 | 1 | 1 | rs3129763 | 6:32590925_G_A |
| Zhang_R_2022 | R. Zhang (2022) | rs114544105 | A | 6:32667852 | 0 | 0 | 0 | NA | NA |
| Zhang_R_2022 | R. Zhang (2022) | rs6920364 | C | 6:167376466 | 1 | 1 | 1 | rs6920364 | 6:167376466_G_C |
| Zhang_R_2022 | R. Zhang (2022) | rs11780471 | A | 8:27344719 | 1 | 1 | 1 | rs11780471 | 8:27344719_G_A |
| Zhang_R_2022 | R. Zhang (2022) | rs4236709 | G | 8:32410110 | 1 | 1 | 1 | rs4236709 | 8:32410110_G_A |
| Zhang_R_2022 | R. Zhang (2022) | rs885518 | G | 9:21830157 | 1 | 1 | 1 | rs885518 | 9:21830157_A_G |

## S9 List of Included SNPs

| PGS_name | Label | rsID | Effect allele | Position | rsID in UKB extract | position in UKB extract | found in UKB extract | rsID in UKB | Position in UKB |
|---|---|---|---|---|---|---|---|---|---|
| Zhang_R_2022 | R. Zhang (2022) | rs2007153 | T | 9:136503819 | 1 | 1 | 1 | rs2007153 | rs2007153 |
| Zhang_R_2022 | R. Zhang (2022) | rs11591710 | C | 10:105687632 | 1 | 1 | 1 | rs11591710 | 10:105687632_A_C |
| Zhang_R_2022 | R. Zhang (2022) | rs1056562 | T | 11:118125625 | 1 | 1 | 1 | rs1056562 | 11:118125625_T_C |
| Zhang_R_2022 | R. Zhang (2022) | rs7953330 | C | 12:998819 | 1 | 1 | 1 | rs7953330 | 12:998819_G_C |
| Zhang_R_2022 | R. Zhang (2022) | rs11571833 | T | 13:32972626 | 1 | 1 | 1 | rs11571833 | rs11571833 |
| Zhang_R_2022 | R. Zhang (2022) | rs689647 | T | 15:43762196 | 1 | 1 | 1 | rs689647 | rs689647 |
| Zhang_R_2022 | R. Zhang (2022) | rs66759488 | A | 15:47577451 | 1 | 1 | 1 | rs66759488 | 15:47577451_G_A |
| Zhang_R_2022 | R. Zhang (2022) | rs77468143 | G | 15:49376624 | 1 | 1 | 1 | rs77468143 | 15:49376624_T_G |
| Zhang_R_2022 | R. Zhang (2022) | rs3885951 | G | 15:78825917 | 1 | 1 | 1 | rs3885951 | rs3885951 |
| Zhang_R_2022 | R. Zhang (2022) | rs55781567 | G | 15:78857986 | 1 | 1 | 1 | rs55781567 | rs55781567 |
| Zhang_R_2022 | R. Zhang (2022) | rs7177699 | C | 15:79089734 | 1 | 1 | 1 | rs7177699 | 15:79089734_T_C |
| Zhang_R_2022 | R. Zhang (2022) | rs62070270 | G | 17:29936962 | 0 | 0 | 0 | NA | NA |
| Zhang_R_2022 | R. Zhang (2022) | rs1542752 | T | 17:72938100 | 1 | 1 | 1 | rs1542752 | rs1542752 |
| Zhang_R_2022 | R. Zhang (2022) | rs56113850 | T | 19:41353107 | 1 | 1 | 1 | rs56113850 | 19:41353107_T_C |
| Zhang_R_2022 | R. Zhang (2022) | rs41309931 | T | 20:62326579 | 1 | 1 | 1 | rs41309931 | 20:62326579_G_T |
| Zhang_R_2022 | R. Zhang (2022) | rs17879961 | G | 22:29121087 | 1 | 1 | 1 | rs17879961 | rs17879961 |
| Zhang_R_2022 | R. Zhang (2022) | rs71641333 | A | 1:78743005 | 1 | 1 | 1 | rs71641333 | 1:78743005_T_A |
| Zhang_R_2022 | R. Zhang (2022) | rs78062588 | C | 1:154566225 | 1 | 1 | 1 | rs78062588 | 1:154566225_T_C |
| Zhang_R_2022 | R. Zhang (2022) | rs114737056 | A | 1:168511081 | 1 | 1 | 1 | rs114737056 | 1:168511081_G_A |
| Zhang_R_2022 | R. Zhang (2022) | rs145733018 | C | 2:38567201 | 1 | 1 | 1 | rs145733018 | 2:38567201_T_C |
| Zhang_R_2022 | R. Zhang (2022) | rs79368540 | T | 2:45189737 | 1 | 1 | 1 | rs79368540 | 2:45189737_C_T |
| Zhang_R_2022 | R. Zhang (2022) | rs11692700 | C | 2:67510377 | 1 | 1 | 1 | rs11692700 | 2:67510377_T_C |
| Zhang_R_2022 | R. Zhang (2022) | rs114928225 | A | 2:119449740 | 1 | 1 | 1 | rs114928225 | 2:119449740_T_A |
| Zhang_R_2022 | R. Zhang (2022) | rs7592999 | C | 2:140398327 | 1 | 1 | 1 | rs7592999 | 2:140398327_T_C |
| Zhang_R_2022 | R. Zhang (2022) | rs722864 | A | 2:173983204 | 1 | 1 | 1 | rs722864 | 2:173983204_A_G |
| Zhang_R_2022 | R. Zhang (2022) | rs1866631 | G | 2:174075761 | 1 | 1 | 1 | rs1866631 | 2:174075761_G_A |
| Zhang_R_2022 | R. Zhang (2022) | rs185666783 | G | 4:67833774 | 1 | 1 | 1 | rs185666783 | 4:67833774_C_G |
| Zhang_R_2022 | R. Zhang (2022) | rs7676823 | G | 4:164007992 | 1 | 1 | 1 | rs7676823 | 4:164007992_G_A |
| Zhang_R_2022 | R. Zhang (2022) | rs78154696 | A | 5:1000156 | 1 | 1 | 1 | rs78154696 | 5:1000156_G_A |
| Zhang_R_2022 | R. Zhang (2022) | rs112333466 | T | 5:1249816 | 1 | 1 | 1 | rs112333466 | 5:1249816_C_T |
| Zhang_R_2022 | R. Zhang (2022) | rs56345976 | G | 5:1276873 | 1 | 1 | 1 | rs56345976 | 5:1276873_G_A |
| Zhang_R_2022 | R. Zhang (2022) | rs2853677 | G | 5:1287194 | 1 | 1 | 1 | rs2853677 | rs2853677 |
| Zhang_R_2022 | R. Zhang (2022) | rs112401627 | A | 5:1300269 | 1 | 1 | 1 | rs112401627 | 5:1300269_G_A |
| Zhang_R_2022 | R. Zhang (2022) | rs6875416 | T | 5:90250631 | 1 | 1 | 1 | rs6875416 | 5:90250631_A_T |
| Zhang_R_2022 | R. Zhang (2022) | rs114136906 | C | 5:150121458 | 1 | 1 | 1 | rs114136906 | 5:150121458_G_C |
| Zhang_R_2022 | R. Zhang (2022) | rs2316515 | A | 6:410848 | 1 | 1 | 1 | rs2316515 | 6:410848_A_G |
| Zhang_R_2022 | R. Zhang (2022) | rs629444 | T | 6:25885814 | 1 | 1 | 1 | rs629444 | rs629444 |
| Zhang_R_2022 | R. Zhang (2022) | rs2179517 | C | 6:25885814 | 1 | 1 | 1 | rs629444 | rs629444 |
| Zhang_R_2022 | R. Zhang (2022) | rs68141011 | T | 6:28217797 | 1 | 1 | 1 | rs68141011 | 6:28217797_G_A |
| Zhang_R_2022 | R. Zhang (2022) | rs114722608 | C | 6:29223493 | 0 | 1 | 1 | rs3130825 | 6:29223493_G_C |
| Zhang_R_2022 | R. Zhang (2022) | rs115123779 | T | 6:29477821 | 0 | 1 | 1 | rs1233478 | rs1233478 |
| Zhang_R_2022 | R. Zhang (2022) | rs138488080 | A | 6:29606761 | 0 | 1 | 1 | rs3095268 | 6:29606761_G_A |
| Zhang_R_2022 | R. Zhang (2022) | rs114192654 | A | 6:29759750 | 0 | 1 | 1 | rs1611203 | 6:29759750_G_A |
| Zhang_R_2022 | R. Zhang (2022) | rs116675020 | G | 6:29922740 | 0 | 1 | 1 | rs16896742 | rs16896742 |
| Zhang_R_2022 | R. Zhang (2022) | rs115993819 | A | 6:30074163 | 0 | 1 | 1 | rs2517600 | rs2517600 |
| Zhang_R_2022 | R. Zhang (2022) | rs116534499 | G | 6:30138162 | 0 | 1 | 1 | rs1029239 | rs1029239 |
| Zhang_R_2022 | R. Zhang (2022) | rs116629156 | C | 6:30864829 | 0 | 1 | 1 | rs1049623 | rs1049623 |
| Zhang_R_2022 | R. Zhang (2022) | rs114103504 | G | 6:31002452 | 0 | 1 | 1 | rs3869098 | 6:31002452_A_G |
| Zhang_R_2022 | R. Zhang (2022) | rs114052224 | G | 6:31067852 | 0 | 1 | 1 | rs3130548 | 6:31067852_A_G |
| Zhang_R_2022 | R. Zhang (2022) | rs2233959 | C | 6:31081065 | 1 | 1 | 1 | rs2233959 | 6:31081065_T_C |
| Zhang_R_2022 | R. Zhang (2022) | rs114689412 | G | 6:31117577 | 0 | 1 | 1 | rs1265113 | 6:31117577_C_G |
| Zhang_R_2022 | R. Zhang (2022) | rs2596499 | A | 6:31321429 | 1 | 1 | 1 | rs2596499 | 6:31321429_T_A |
| Zhang_R_2022 | R. Zhang (2022) | rs2596496 | C | 6:31322782 | 1 | 1 | 1 | rs2596496 | 6:31322782_C_G |
| Zhang_R_2022 | R. Zhang (2022) | rs2596490 | G | 6:31324996 | 1 | 1 | 1 | rs2596490 | 6:31324996_G_A |
| Zhang_R_2022 | R. Zhang (2022) | rs115176861 | C | 6:31412961 | 0 | 1 | 1 | rs2596464 | Affx-28456540 |
| Zhang_R_2022 | R. Zhang (2022) | rs553108 | A | 6:31840455 | 1 | 1 | 1 | rs553108 | 6:31840455_A_G |
| Zhang_R_2022 | R. Zhang (2022) | rs115200960 | A | 6:32335204 | 0 | 1 | 1 | rs2143462 | rs2143462 |
| Zhang_R_2022 | R. Zhang (2022) | rs12722051 | T | 6:32609147 | 1 | 1 | 1 | rs12722051 | 6:32609147_A_T |
| Zhang_R_2022 | R. Zhang (2022) | rs116767258 | G | 6:32757737 | 0 | 1 | 1 | rs2857165 | rs2857165 |
| Zhang_R_2022 | R. Zhang (2022) | rs7383287 | G | 6:32783086 | 1 | 1 | 1 | rs7383287 | rs7383287 |
| Zhang_R_2022 | R. Zhang (2022) | rs117534741 | A | 6:72384541 | 1 | 1 | 1 | rs117534741 | rs117534741 |
| Zhang_R_2022 | R. Zhang (2022) | rs1321817 | G | 6:117734267 | 1 | 1 | 1 | rs1321817 | 6:117734267_A_G |
| Zhang_R_2022 | R. Zhang (2022) | rs6957511 | C | 7:130668618 | 1 | 1 | 1 | rs6957511 | 7:130668618_C_T |
| Zhang_R_2022 | R. Zhang (2022) | rs2565064 | C | 8:27327841 | 1 | 1 | 1 | rs2565064 | 8:27327841_C_G |
| Zhang_R_2022 | R. Zhang (2022) | rs67749759 | T | 8:27397087 | 0 | 1 | 1 | rs4149254 | 8:27397087_C_T |

## S9 List of Included SNPs

| PGS_name | Label | rsID | Effect allele | Position | rsID in UKB extract | position in UKB extract | found in UKB extract | rsID in UKB | Position in UKB |
|---|---|---|---|---|---|---|---|---|---|
| Zhang_R_2022 | R. Zhang (2022) | rs111960002 | C | 8:144722420 | 1 | 1 | 1 | rs111960002 | 8:144722420_T_C |
| Zhang_R_2022 | R. Zhang (2022) | rs10118776 | G | 9:6227418 | 1 | 1 | 1 | rs10118776 | 9:6227418_G_A |
| Zhang_R_2022 | R. Zhang (2022) | rs17185553 | C | 9:17934120 | 1 | 1 | 1 | rs17185553 | 9:17934120_G_C |
| Zhang_R_2022 | R. Zhang (2022) | rs2518717 | C | 9:21959751 | 1 | 1 | 1 | rs2518717 | 9:21959751_T_C |
| Zhang_R_2022 | R. Zhang (2022) | rs28557075 | A | 9:22066572 | 1 | 1 | 1 | rs28557075 | 9:22066572_G_A |
| Zhang_R_2022 | R. Zhang (2022) | rs1333040 | C | 9:22083404 | 1 | 1 | 1 | rs1333040 | rs1333040 |
| Zhang_R_2022 | R. Zhang (2022) | rs4879704 | C | 9:33427322 | 1 | 1 | 1 | rs4879704 | 9:33427322_A_C |
| Zhang_R_2022 | R. Zhang (2022) | rs191205566 | T | 9:102587233 | 1 | 1 | 1 | rs191205566 | 9:102587233_C_T |
| Zhang_R_2022 | R. Zhang (2022) | rs75685923 | T | 9:136275229 | 1 | 1 | 1 | rs75685923 | rs75685923 |
| Zhang_R_2022 | R. Zhang (2022) | rs7897454 | A | 10:102011702 | 1 | 1 | 1 | rs7897454 | 10:102011702_G_A |
| Zhang_R_2022 | R. Zhang (2022) | rs62621207 | T | 10:102672248 | 1 | 1 | 1 | rs62621207 | 10:102672248_A_T |
| Zhang_R_2022 | R. Zhang (2022) | rs78853063 | T | 11:57250026 | 1 | 1 | 1 | rs78853063 | 11:57250026_C_T |
| Zhang_R_2022 | R. Zhang (2022) | rs78334599 | A | 11:115998756 | 1 | 1 | 1 | rs78334599 | 11:115998756_G_A |
| Zhang_R_2022 | R. Zhang (2022) | rs7487683 | T | 12:1036042 | 1 | 1 | 1 | rs7487683 | rs7487683 |
| Zhang_R_2022 | R. Zhang (2022) | rs73351723 | A | 12:58831070 | 1 | 1 | 1 | rs73351723 | 12:58831070_G_A |
| Zhang_R_2022 | R. Zhang (2022) | rs9668978 | T | 12:64913237 | 1 | 1 | 1 | rs9668978 | rs9668978 |
| Zhang_R_2022 | R. Zhang (2022) | rs9602270 | T | 13:84281063 | 1 | 1 | 1 | rs9602270 | 13:84281063_A_T |
| Zhang_R_2022 | R. Zhang (2022) | rs8003466 | A | 14:34013721 | 1 | 1 | 1 | rs8003466 | 14:34013721_G_A |
| Zhang_R_2022 | R. Zhang (2022) | rs8031813 | C | 15:49253961 | 1 | 1 | 1 | rs8031813 | 15:49253961_C_A |
| Zhang_R_2022 | R. Zhang (2022) | rs6493361 | G | 15:49615952 | 1 | 1 | 1 | rs6493361 | 15:49615952_G_C |
| Zhang_R_2022 | R. Zhang (2022) | rs11855650 | T | 15:70431773 | 1 | 1 | 1 | rs11855650 | rs11855650 |
| Zhang_R_2022 | R. Zhang (2022) | rs79149102 | T | 15:75055819 | 1 | 1 | 1 | rs79149102 | 15:75055819_C_T |
| Zhang_R_2022 | R. Zhang (2022) | rs2229961 | A | 15:78880752 | 1 | 1 | 1 | rs2229961 | rs2229961 |
| Zhang_R_2022 | R. Zhang (2022) | rs8192479 | T | 15:78909398 | 1 | 1 | 1 | rs8192479 | rs8192479 |
| Zhang_R_2022 | R. Zhang (2022) | rs2869551 | G | 15:78981423 | 1 | 1 | 1 | rs2869551 | 15:78981423_A_G |
| Zhang_R_2022 | R. Zhang (2022) | rs12593207 | A | 15:78987225 | 1 | 1 | 1 | rs12593207 | rs12593207 |
| Zhang_R_2022 | R. Zhang (2022) | rs189146505 | G | 15:79058730 | 1 | 1 | 1 | rs189146505 | 15:79058730_A_G |
| Zhang_R_2022 | R. Zhang (2022) | rs28450923 | G | 15:79065557 | 1 | 1 | 1 | rs28450923 | 15:79065557_A_G |
| Zhang_R_2022 | R. Zhang (2022) | rs28624856 | C | 15:79075233 | 1 | 1 | 1 | rs28624856 | 15:79075233_C_T |
| Zhang_R_2022 | R. Zhang (2022) | rs77719127 | T | 15:79110783 | 1 | 1 | 1 | rs77719127 | 15:79110783_C_T |
| Zhang_R_2022 | R. Zhang (2022) | rs76164573 | G | 15:79198760 | 1 | 1 | 1 | rs76164573 | rs76164573 |
| Zhang_R_2022 | R. Zhang (2022) | rs78442819 | C | 16:10740982 | 1 | 1 | 1 | rs78442819 | 16:10740982_G_C |
| Zhang_R_2022 | R. Zhang (2022) | rs9926896 | C | 16:26980646 | 1 | 1 | 1 | rs9926896 | 16:26980646_T_C |
| Zhang_R_2022 | R. Zhang (2022) | rs17181550 | G | 17:70299958 | 1 | 1 | 1 | rs17181550 | rs17181550 |
| Zhang_R_2022 | R. Zhang (2022) | rs79421398 | C | 18:20741135 | 1 | 1 | 1 | rs79421398 | 18:20741135_T_C |
| Zhang_R_2022 | R. Zhang (2022) | rs66500423 | C | 19:41195170 | 1 | 1 | 1 | rs66500423 | 19:41195170_T_C |
| Zhang_R_2022 | R. Zhang (2022) | rs4803356 | G | 19:41207206 | 1 | 1 | 1 | rs4803356 | 19:41207206_C_G |
| Zhang_R_2022 | R. Zhang (2022) | rs11881918 | A | 19:41334199 | 1 | 1 | 1 | rs11881918 | 19:41334199_G_A |
| Zhang_R_2022 | R. Zhang (2022) | rs2258380 | G | 19:41338988 | 1 | 1 | 1 | rs2258380 | Affx-36248730 |
| Zhang_R_2022 | R. Zhang (2022) | rs67210567 | T | 19:41357457 | 1 | 1 | 1 | rs67210567 | 19:41357457_G_T |
| Zhang_R_2022 | R. Zhang (2022) | rs184589612 | C | 19:41412192 | 1 | 1 | 1 | rs184589612 | 19:41412192_T_C |
| Zhang_R_2022 | R. Zhang (2022) | rs12981718 | A | 19:54567858 | 1 | 1 | 1 | rs12981718 | 19:54567858_G_A |
| Zhang_R_2022 | R. Zhang (2022) | rs13036436 | G | 20:61988382 | 1 | 1 | 1 | rs13036436 | 20:61988382_G_A |
| Zhang_R_2022 | R. Zhang (2022) | rs61541144 | A | 20:62527305 | 1 | 1 | 1 | rs61541144 | 20:62527305_G_A |
| Zhu_2023_Study_2_A | Zhu (2023a) | rs17038564 | G | 2:NA | 1 | 0 | 1 | rs17038564 | NA |
| Zhu_2023_Study_2_A | Zhu (2023a) | rs2293607 | T | 3:NA | 1 | 0 | 1 | rs2293607 | NA |
| Zhu_2023_Study_2_A | Zhu (2023a) | rs11375254 | T | 3:NA | 1 | 0 | 1 | rs11375254 | NA |
| Zhu_2023_Study_2_A | Zhu (2023a) | rs13167280 | A | 5:NA | 1 | 0 | 1 | rs13167280 | NA |
| Zhu_2023_Study_2_A | Zhu (2023a) | rs401681 | C | 5:NA | 1 | 0 | 1 | rs401681 | NA |
| Zhu_2023_Study_2_A | Zhu (2023a) | rs115790694 | A | 6:NA | 0 | 0 | 0 | NA | NA |
| Zhu_2023_Study_2_A | Zhu (2023a) | rs3817963 | C | 6:NA | 1 | 0 | 1 | rs3817963 | NA |
| Zhu_2023_Study_2_A | Zhu (2023a) | rs1853837 | A | 6:NA | 1 | 0 | 1 | rs1853837 | NA |
| Zhu_2023_Study_2_A | Zhu (2023a) | rs5879422 | T | 6:NA | 1 | 0 | 1 | rs5879422 | NA |
| Zhu_2023_Study_2_A | Zhu (2023a) | rs4236709 | G | 8:NA | 1 | 0 | 1 | rs4236709 | NA |
| Zhu_2023_Study_2_A | Zhu (2023a) | rs10429489 | A | 9:NA | 1 | 0 | 1 | rs10429489 | NA |
| Zhu_2023_Study_2_A | Zhu (2023a) | rs35201538 | C | 9:NA | 1 | 0 | 1 | rs35201538 | NA |
| Zhu_2023_Study_2_A | Zhu (2023a) | rs4573350 | T | 9:NA | 1 | 0 | 1 | rs4573350 | NA |
| Zhu_2023_Study_2_A | Zhu (2023a) | rs12265047 | G | 10:NA | 1 | 0 | 1 | rs12265047 | NA |
| Zhu_2023_Study_2_A | Zhu (2023a) | rs55768116 | C | 11:NA | 1 | 0 | 1 | rs55768116 | NA |
| Zhu_2023_Study_2_A | Zhu (2023a) | rs11610143 | C | 12:NA | 1 | 0 | 1 | rs11610143 | NA |
| Zhu_2023_Study_2_A | Zhu (2023a) | rs1200399 | C | 14:NA | 1 | 0 | 1 | rs1200399 | NA |
| Zhu_2023_Study_2_A | Zhu (2023a) | rs77468143 | T | 15:NA | 1 | 0 | 1 | rs77468143 | NA |
| Zhu_2023_Study_2_A | Zhu (2023a) | rs200595745 | A | 17:NA | 0 | 0 | 0 | NA | NA |
| Zhu_2023_Study_2_B | Zhu (2023b) | rs71658797 | A | 1:NA | 1 | 0 | 1 | rs71658797 | NA |
| Zhu_2023_Study_2_B | Zhu (2023b) | rs3769821 | C | 2:NA | 1 | 0 | 1 | rs3769821 | NA |

## S9 List of Included SNPs

| PGS_name | Label | rsID | Effect allele | Position | rsID in UKB extract | position in UKB extract | found in UKB extract | rsID in UKB | Position in UKB |
|---|---|---|---|---|---|---|---|---|---|
| Zhu_2023_Study_2_B | Zhu (2023b) | rs11375254 | T | 3:NA | 1 | 0 | 1 | rs11375254 | NA |
| Zhu_2023_Study_2_B | Zhu (2023b) | rs13167280 | A | 5:NA | 1 | 0 | 1 | rs13167280 | NA |
| Zhu_2023_Study_2_B | Zhu (2023b) | rs2853677 | G | 5:NA | 1 | 0 | 1 | rs2853677 | NA |
| Zhu_2023_Study_2_B | Zhu (2023b) | rs401681 | C | 5:NA | 1 | 0 | 1 | rs401681 | NA |
| Zhu_2023_Study_2_B | Zhu (2023b) | rs115790694 | A | 6:NA | 0 | 0 | 0 | NA | NA |
| Zhu_2023_Study_2_B | Zhu (2023b) | rs116822326 | G | 6:NA | 0 | 0 | 0 | NA | NA |
| Zhu_2023_Study_2_B | Zhu (2023b) | rs6920364 | C | 6:NA | 1 | 0 | 1 | rs6920364 | NA |
| Zhu_2023_Study_2_B | Zhu (2023b) | rs11780471 | G | 8:NA | 1 | 0 | 1 | rs11780471 | NA |
| Zhu_2023_Study_2_B | Zhu (2023b) | rs4236709 | G | 8:NA | 1 | 0 | 1 | rs4236709 | NA |
| Zhu_2023_Study_2_B | Zhu (2023b) | rs10429489 | A | 9:NA | 1 | 0 | 1 | rs10429489 | NA |
| Zhu_2023_Study_2_B | Zhu (2023b) | rs1333040 | C | 9:NA | 1 | 0 | 1 | rs1333040 | NA |
| Zhu_2023_Study_2_B | Zhu (2023b) | rs11591710 | C | 10:NA | 1 | 0 | 1 | rs11591710 | NA |
| Zhu_2023_Study_2_B | Zhu (2023b) | rs55768116 | C | 11:NA | 1 | 0 | 1 | rs55768116 | NA |
| Zhu_2023_Study_2_B | Zhu (2023b) | rs6489769 | T | 12:NA | 1 | 0 | 1 | rs6489769 | NA |
| Zhu_2023_Study_2_B | Zhu (2023b) | rs11571833 | T | 13:NA | 1 | 0 | 1 | rs11571833 | NA |
| Zhu_2023_Study_2_B | Zhu (2023b) | rs66759488 | A | 15:NA | 1 | 0 | 1 | rs66759488 | NA |
| Zhu_2023_Study_2_B | Zhu (2023b) | rs77468143 | T | 15:NA | 1 | 0 | 1 | rs77468143 | NA |
| Zhu_2023_Study_2_B | Zhu (2023b) | rs55781567 | G | 15:NA | 1 | 0 | 1 | rs55781567 | NA |
| Zhu_2023_Study_2_B | Zhu (2023b) | rs56113850 | C | 19:NA | 1 | 0 | 1 | rs56113850 | NA |
| Zhu_2023_Study_2_B | Zhu (2023b) | rs41309931 | T | 20:NA | 1 | 0 | 1 | rs41309931 | NA |
| Zhu_2023_Study_2_B | Zhu (2023b) | rs17879961 | A | 22:NA | 1 | 0 | 1 | rs17879961 | NA |
| Zhu_2023_Study_2_C | Zhu (2023c) | rs17038564 | G | 2:NA | 1 | 0 | 1 | rs17038564 | NA |
| Zhu_2023_Study_2_C | Zhu (2023c) | rs3769821 | C | 2:NA | 1 | 0 | 1 | rs3769821 | NA |
| Zhu_2023_Study_2_C | Zhu (2023c) | rs2293607 | T | 3:NA | 1 | 0 | 1 | rs2293607 | NA |
| Zhu_2023_Study_2_C | Zhu (2023c) | rs11375254 | T | 3:NA | 1 | 0 | 1 | rs11375254 | NA |
| Zhu_2023_Study_2_C | Zhu (2023c) | rs13167280 | A | 5:NA | 1 | 0 | 1 | rs13167280 | NA |
| Zhu_2023_Study_2_C | Zhu (2023c) | rs465498 | A | 5:NA | 1 | 0 | 1 | rs465498 | NA |
| Zhu_2023_Study_2_C | Zhu (2023c) | rs115790694 | A | 6:NA | 0 | 0 | 0 | NA | NA |
| Zhu_2023_Study_2_C | Zhu (2023c) | rs2395185 | T | 6:NA | 1 | 0 | 1 | rs2395185 | NA |
| Zhu_2023_Study_2_C | Zhu (2023c) | rs1853837 | A | 6:NA | 1 | 0 | 1 | rs1853837 | NA |
| Zhu_2023_Study_2_C | Zhu (2023c) | rs5879422 | T | 6:NA | 1 | 0 | 1 | rs5879422 | NA |
| Zhu_2023_Study_2_C | Zhu (2023c) | rs6920364 | C | 6:NA | 1 | 0 | 1 | rs6920364 | NA |
| Zhu_2023_Study_2_C | Zhu (2023c) | rs4236709 | G | 8:NA | 1 | 0 | 1 | rs4236709 | NA |
| Zhu_2023_Study_2_C | Zhu (2023c) | rs10429489 | A | 9:NA | 1 | 0 | 1 | rs10429489 | NA |
| Zhu_2023_Study_2_C | Zhu (2023c) | rs35201538 | C | 9:NA | 1 | 0 | 1 | rs35201538 | NA |
| Zhu_2023_Study_2_C | Zhu (2023c) | rs4573350 | T | 9:NA | 1 | 0 | 1 | rs4573350 | NA |
| Zhu_2023_Study_2_C | Zhu (2023c) | rs11591710 | C | 10:NA | 1 | 0 | 1 | rs11591710 | NA |
| Zhu_2023_Study_2_C | Zhu (2023c) | rs12265047 | G | 10:NA | 1 | 0 | 1 | rs12265047 | NA |
| Zhu_2023_Study_2_C | Zhu (2023c) | rs55768116 | C | 11:NA | 1 | 0 | 1 | rs55768116 | NA |
| Zhu_2023_Study_2_C | Zhu (2023c) | rs3748522 | C | 12:NA | 1 | 0 | 1 | rs3748522 | NA |
| Zhu_2023_Study_2_C | Zhu (2023c) | rs1200399 | C | 14:NA | 1 | 0 | 1 | rs1200399 | NA |
| Zhu_2023_Study_2_C | Zhu (2023c) | rs77468143 | T | 15:NA | 1 | 0 | 1 | rs77468143 | NA |
| Zhu_2023_Study_2_C | Zhu (2023c) | rs55781567 | G | 15:NA | 1 | 0 | 1 | rs55781567 | NA |
| Zhu_2023_Study_2_C | Zhu (2023c) | rs6495306 | A | 15:NA | 1 | 0 | 1 | rs6495306 | NA |
| Zhu_2023_Study_2_C | Zhu (2023c) | rs200595745 | A | 17:NA | 0 | 0 | 0 | NA | NA |
| Zhu_2023_Study_2_C | Zhu (2023c) | rs56113850 | C | 19:NA | 1 | 0 | 1 | rs56113850 | NA |

## S10 Missing SNPs

| PGS_name | Number of SNPs in PGS | Number avalible in UKB data | Percentage avalible in UKB (%) | Number of SNPs missing in UKB validation | Notes |
|---|---|---|---|---|---|
| *PGP000049_Dai_2019* | 19 | 19 | 100.0 | 0 | |
| *PGP000050_Graff_2021* | 109 | 109 | 100.0 | 0 | |
| *PGP000075_Shi_2019* | 6 | 6 | 100.0 | 0 | |
| *PGP000135_Jia_2020* | 19 | 19 | 100.0 | 0 | |
| *PGP000148_Hung_2021* | 128 | 124 | 96.9 | 4 | |
| *PGP000186_Kachuri_2020* | 109 | 109 | 100.0 | 0 | |
| *PGP000226_Xie_2021* | 18 | 18 | 100.0 | 0 | |
| *PGP000300_Zhang_2022* | 33 | 33 | 100.0 | 0 | |
| *PGP000387_Qin_2022* | 20 | 20 | 100.0 | 0 | |
| *PGP000518a_Wei_2023* | 16 | 16 | 100.0 | 0 | |
| *PGP000518b_Wei_2023* | 21 | 21 | 100.0 | 0 | |
| *PGP000542_Kim_2023* | 43 | 43 | 100.0 | 0 | |
| *PGP000559_Wang_2023* | 19 | 19 | 100.0 | 0 | |
| *Zhang_R_2022* | 128 | 124 | 96.9 | 4 | |
| *Zhu_2023_Study_2_A* | 19 | 17 | 89.5 | 2 | |
| *Zhu_2023_Study_2_B* | 23 | 21 | 91.3 | 2 | |
| *Zhu_2023_Study_2_C* | 25 | 23 | 92.0 | 2 | |
| *barnett_2022_study_12* | 25 | 22 | 88.0 | 3 | |
| *blechter_2023_study_41* | 25 | 25 | 100.0 | 0 | |
| *choi_2022_study_32* | 19 | 19 | 100.0 | 0 | |
| *choi_2022_study_43_A* | 19 | 19 | 100.0 | 0 | |
| *choi_2022_study_43_B* | 17 | 17 | 100.0 | 0 | |
| *huang_2021_study_17* | 18 | 18 | 100.0 | 0 | |
| *jia_2021_study_18* | 19 | 19 | 100.0 | 0 | |
| *liang_2023_study_35* | 18 | 17 | 94.4 | 1 | |
| *liu_2022_study_4_A* | 15 | 13 | 86.7 | 2 | |
| *liu_2022_study_4_B* | 6 | 6 | 100.0 | 0 | |
| *qian_2016_study22* | 301 | 301 | 100.0 | 0 | |
| *shi_2023_study_27_A* | 28 | 28 | 100.0 | 0 | |
| *shi_2023_study_27_B* | 22 | 22 | 100.0 | 0 | |
| *shi_2023_study_27_C* | 22 | 22 | 100.0 | 0 | |
| *wang_2021_study_38* | 18 | 18 | 100.0 | 0 | |
| *wang_2022_study_13* | 73 | 37 | 50.7 | 36 | *excluded from validation due to high proportion (>50%) of the SNPs not being avaliable in UKB* |
| *wang_2022_study_37* | 18 | 17 | 94.4 | 1 | |
| *xiao_2023_study_15* | 19 | 18 | 94.7 | 1 | |
| *xin_2023_study34* | 16 | 16 | 100.0 | 0 | |
| *yang_2016_study_31* | 38 | 38 | 100.0 | 0 | |
| *yu_2020_study_10* | 31 | 30 | 96.8 | 1 | |
| *zhang_2023_study_40* | 18 | 18 | 100.0 | 0 | |

**S11 PGS with duplicate SNPs**

| | |
|---|---|
| Same 19 SNPs | Jia2020, Jia2021, J. Choi2022a, J. Choi2022c, Wang2023 |
| Same 109 SNPs | Graff 2021, Kachuri2020 |
| Same 128 SNPs | Hung2021, R. Zhang 2022 |
| Same 18 SNPs | Xie 2021, Huang2021, Liang2023, Wang 2022b, Zhang2023 |
| Same 22 SNPs | Shi2023b, Shi2023c |

*Note Qian 2016 (301 SNPs) not seen in any other PGS. Yang 2016 shares max 8% of its 38 SNPs (with P. Zhang 2022)*
*Several use all the SNPs in other PGS, with others added – e.g. the 128 SNPs used in Hung2021 (and R. Zhang2022) contain all of the 18 SNPs used in Xie2021 (Huang2021, Liang2023, Wang 2022b, Zhang2023).*

**S12 C-indices**

| Polygenic Score | C-indices in UK Biobank | | | | | | | |
|---|---|---|---|---|---|---|---|---|
| | Whole Cohort | Men Only | Women Only | Non-white individuals | White Individuals | Current Smokers | Former Smokers | Never Smokers |
| | *n=429,665* | *n=202,714* | *n=226,951* | *n=24558* | *n=402,318* | *n=45,391* | *n=146,777* | *n=235,297* |
| *Cheng (2016)* | 0.521 (0.531-0.512) | 0.514 (0.527-0.501) | 0.529 (0.542-0.515) | 0.516 (0.460-0.573) | 0.518 (0.528-0.508) | 0.518 (0.533-0.503) | 0.525 (0.539-0.511) | 0.522 (0.547-0.497) |
| *Qian (2016)* | 0.532 (0.523-0.541) | 0.539 (0.526-0.552) | 0.525 (0.511-0.539) | 0.511 (0.457-0.565) | 0.536 (0.527-0.546) | 0.536 (0.522-0.551) | 0.534 (0.520-0.549) | 0.506 (0.532-0.480) |
| *Dai (2019)* | 0.539 (0.529-0.548) | 0.536 (0.523-0.550) | 0.542 (0.528-0.556) | 0.576 (0.527-0.626) | 0.543 (0.534-0.553) | 0.527 (0.512-0.542) | 0.549 (0.535-0.563) | 0.566 (0.540-0.592) |
| *Shi (2019)* | 0.534 (0.525-0.544) | 0.533 (0.520-0.547) | 0.534 (0.521-0.548) | 0.505 (0.449-0.561) | 0.533 (0.523-0.542) | 0.543 (0.528-0.557) | 0.538 (0.524-0.553) | 0.506 (0.479-0.532) |
| *Jia (2020)* | 0.569 (0.559-0.578) | 0.565 (0.553-0.578) | 0.573 (0.559-0.586) | 0.524 (0.468-0.581) | 0.567 (0.558-0.577) | 0.571 (0.556-0.585) | 0.575 (0.561-0.589) | 0.536 (0.509-0.562) |
| *Kachuri (2020)* | 0.555 (0.545-0.564) | 0.558 (0.545-0.571) | 0.551 (0.538-0.565) | 0.525 (0.469-0.582) | 0.552 (0.542-0.561) | 0.569 (0.555-0.583) | 0.550 (0.536-0.564) | 0.528 (0.502-0.553) |
| *Yu (2020)* | 0.565 (0.555-0.574) | 0.563 (0.550-0.576) | 0.567 (0.553-0.581) | 0.523 (0.466-0.580) | 0.562 (0.553-0.572) | 0.569 (0.554-0.583) | 0.567 (0.553-0.581) | 0.542 (0.516-0.568) |
| *Graff (2021)* | 0.555 (0.545-0.564) | 0.558 (0.545-0.571) | 0.551 (0.538-0.565) | 0.525 (0.469-0.582) | 0.552 (0.542-0.561) | 0.569 (0.555-0.583) | 0.550 (0.536-0.564) | 0.528 (0.502-0.553) |
| *Huang (2021)* | 0.559 (0.568-0.549) | 0.557 (0.570-0.544) | 0.560 (0.574-0.547) | 0.542 (0.483-0.600) | 0.558 (0.567-0.548) | 0.565 (0.580-0.551) | 0.566 (0.580-0.551) | 0.516 (0.542-0.489) |
| *Hung (2021)* | 0.566 (0.557-0.576) | 0.563 (0.550-0.575) | 0.571 (0.557-0.584) | 0.503 (0.445-0.560) | 0.568 (0.558-0.577) | 0.563 (0.548-0.577) | 0.576 (0.562-0.590) | 0.548 (0.523-0.573) |
| *Jia (2021)* | 0.569 (0.559-0.578) | 0.565 (0.553-0.578) | 0.573 (0.559-0.586) | 0.524 (0.468-0.581) | 0.567 (0.558-0.577) | 0.571 (0.556-0.585) | 0.575 (0.561-0.589) | 0.536 (0.509-0.562) |
| *Wang (2021)* | 0.556 (0.566-0.547) | 0.555 (0.568-0.542) | 0.558 (0.571-0.544) | 0.552 (0.495-0.609) | 0.556 (0.566-0.546) | 0.557 (0.571-0.542) | 0.566 (0.580-0.552) | 0.525 (0.552-0.498) |
| *Xie (2021)* | 0.559 (0.549-0.568) | 0.557 (0.544-0.570) | 0.561 (0.547-0.574) | 0.542 (0.484-0.600) | 0.558 (0.548-0.567) | 0.565 (0.551-0.580) | 0.566 (0.551-0.580) | 0.516 (0.489-0.542) |
| *Barnett (2022)* | 0.560 (0.551-0.569) | 0.560 (0.547-0.573) | 0.560 (0.546-0.574) | 0.547 (0.491-0.603) | 0.559 (0.549-0.568) | 0.561 (0.546-0.575) | 0.567 (0.553-0.581) | 0.532 (0.506-0.559) |
| *J. Choi (2022a)* | 0.569 (0.559-0.578) | 0.565 (0.552-0.578) | 0.572 (0.559-0.586) | 0.524 (0.468-0.581) | 0.567 (0.558-0.577) | 0.570 (0.556-0.585) | 0.575 (0.561-0.590) | 0.536 (0.509-0.562) |
| *J. Choi (2022b)* | 0.567 (0.558-0.576) | 0.563 (0.550-0.576) | 0.571 (0.558-0.585) | 0.519 (0.463-0.575) | 0.566 (0.556-0.575) | 0.569 (0.554-0.583) | 0.573 (0.559-0.587) | 0.538 (0.512-0.565) |
| *J. Choi (2022c)* | 0.569 (0.559-0.578) | 0.565 (0.552-0.578) | 0.572 (0.559-0.586) | 0.524 (0.468-0.581) | 0.567 (0.558-0.577) | 0.570 (0.556-0.585) | 0.575 (0.561-0.590) | 0.536 (0.509-0.562) |
| *Liu (2022a)* | 0.558 (0.549-0.568) | 0.553 (0.540-0.566) | 0.564 (0.550-0.577) | 0.531 (0.474-0.588) | 0.557 (0.548-0.567) | 0.554 (0.539-0.569) | 0.567 (0.553-0.581) | 0.547 (0.521-0.573) |
| *Liu (2022b)* | 0.555 (0.545-0.564) | 0.555 (0.542-0.568) | 0.554 (0.540-0.567) | 0.565 (0.508-0.622) | 0.551 (0.542-0.561) | 0.565 (0.551-0.580) | 0.556 (0.541-0.570) | 0.525 (0.499-0.551) |
| *Qin (2022)* | 0.548 (0.539-0.558) | 0.546 (0.533-0.559) | 0.551 (0.538-0.565) | 0.550 (0.494-0.606) | 0.550 (0.540-0.559) | 0.536 (0.521-0.551) | 0.557 (0.543-0.571) | 0.562 (0.536-0.588) |
| *X. Wang (2022)* | 0.556 (0.566-0.547) | 0.553 (0.566-0.540) | 0.560 (0.574-0.547) | 0.530 (0.473-0.587) | 0.556 (0.566-0.547) | 0.562 (0.576-0.547) | 0.564 (0.578-0.550) | 0.517 (0.543-0.491) |
| *P. Zhang (2022)* | 0.548 (0.538-0.557) | 0.546 (0.533-0.559) | 0.550 (0.536-0.563) | 0.565 (0.511-0.619) | 0.548 (0.539-0.558) | 0.549 (0.534-0.563) | 0.554 (0.540-0.568) | 0.538 (0.513-0.563) |
| *R. Zhang (2022)* | 0.561 (0.552-0.571) | 0.560 (0.547-0.573) | 0.563 (0.549-0.577) | 0.520 (0.463-0.578) | 0.560 (0.551-0.570) | 0.568 (0.554-0.583) | 0.560 (0.546-0.574) | 0.543 (0.518-0.569) |
| *Blechter (2023)* | 0.501 (0.492-0.511) | 0.503 (0.490-0.516) | 0.500 (0.514-0.487) | 0.528 (0.474-0.581) | 0.501 (0.511-0.491) | 0.501 (0.516-0.486) | 0.505 (0.491-0.519) | 0.501 (0.526-0.475) |
| *Kim (2023)* | 0.560 (0.550-0.569) | 0.564 (0.551-0.577) | 0.555 (0.542-0.569) | 0.538 (0.483-0.593) | 0.555 (0.546-0.565) | 0.572 (0.558-0.587) | 0.556 (0.542-0.570) | 0.531 (0.506-0.556) |
| *Liang (2023)* | 0.552 (0.561-0.542) | 0.547 (0.560-0.534) | 0.556 (0.570-0.542) | 0.533 (0.477-0.589) | 0.553 (0.562-0.543) | 0.547 (0.561-0.533) | 0.564 (0.578-0.550) | 0.529 (0.555-0.503) |
| *Shi (2023a)* | 0.526 (0.516-0.535) | 0.521 (0.508-0.535) | 0.531 (0.517-0.544) | 0.511 (0.454-0.568) | 0.526 (0.516-0.535) | 0.523 (0.509-0.538) | 0.526 (0.512-0.541) | 0.544 (0.518-0.571) |
| *Shi (2023b)* | 0.522 (0.512-0.531) | 0.516 (0.503-0.530) | 0.528 (0.514-0.541) | 0.507 (0.449-0.564) | 0.520 (0.510-0.529) | 0.517 (0.502-0.531) | 0.524 (0.509-0.538) | 0.538 (0.512-0.565) |
| *Shi (2023c)* | 0.519 (0.510-0.528) | 0.513 (0.500-0.526) | 0.526 (0.512-0.539) | 0.518 (0.459-0.576) | 0.517 (0.508-0.527) | 0.515 (0.500-0.529) | 0.520 (0.506-0.534) | 0.537 (0.511-0.564) |
| *Wang (2023)* | 0.569 (0.559-0.578) | 0.565 (0.553-0.578) | 0.573 (0.559-0.586) | 0.524 (0.468-0.581) | 0.567 (0.558-0.577) | 0.571 (0.556-0.585) | 0.575 (0.561-0.589) | 0.536 (0.509-0.562) |
| *Wei (2023a)* | 0.523 (0.514-0.533) | 0.521 (0.509-0.534) | 0.525 (0.511-0.539) | 0.539 (0.484-0.593) | 0.522 (0.512-0.532) | 0.516 (0.501-0.531) | 0.528 (0.513-0.542) | 0.539 (0.514-0.565) |
| *Wei (2023b)* | 0.524 (0.515-0.534) | 0.521 (0.508-0.534) | 0.527 (0.514-0.541) | 0.550 (0.492-0.607) | 0.525 (0.515-0.534) | 0.516 (0.501-0.530) | 0.529 (0.515-0.544) | 0.548 (0.523-0.574) |
| *Xiao (2023)* | 0.534 (0.525-0.544) | 0.537 (0.524-0.550) | 0.531 (0.517-0.544) | 0.505 (0.447-0.562) | 0.533 (0.524-0.543) | 0.538 (0.523-0.553) | 0.534 (0.520-0.548) | 0.527 (0.501-0.553) |
| *Xin (2023)* | 0.556 (0.547-0.566) | 0.556 (0.543-0.569) | 0.556 (0.542-0.570) | 0.546 (0.490-0.601) | 0.556 (0.546-0.566) | 0.555 (0.541-0.569) | 0.567 (0.553-0.582) | 0.525 (0.498-0.551) |
| *Zhang (2023)* | 0.556 (0.547-0.566) | 0.555 (0.542-0.568) | 0.558 (0.544-0.571) | 0.552 (0.495-0.610) | 0.556 (0.547-0.566) | 0.556 (0.542-0.571) | 0.566 (0.552-0.581) | 0.525 (0.499-0.552) |
| *Zhu (2023a)* | 0.535 (0.525-0.544) | 0.529 (0.516-0.543) | 0.541 (0.527-0.555) | 0.554 (0.502-0.606) | 0.541 (0.531-0.550) | 0.521 (0.506-0.535) | 0.548 (0.534-0.562) | 0.561 (0.535-0.587) |
| *Zhu (2023b)* | 0.562 (0.552-0.571) | 0.558 (0.545-0.570) | 0.566 (0.553-0.580) | 0.516 (0.459-0.574) | 0.564 (0.555-0.574) | 0.559 (0.544-0.574) | 0.572 (0.558-0.586) | 0.542 (0.517-0.568) |
| *Zhu (2023c)* | 0.552 (0.543-0.562) | 0.549 (0.536-0.562) | 0.557 (0.543-0.570) | 0.539 (0.484-0.593) | 0.559 (0.549-0.568) | 0.546 (0.531-0.561) | 0.566 (0.552-0.580) | 0.550 (0.525-0.574) |

**S13 HRs per sd**

| Polygenic Score | HRs per SD in UK Biobank | | | | | | | |
|---|---|---|---|---|---|---|---|---|
| | Whole Cohort | Men Only | Women Only | Non-white individuals | White Individuals | Current Smokers | Former Smokers | Never Smokers |
| | *n=429,665* | *n=202,714* | *n=226,951* | *n=24558* | *n=402,318* | *n=45,391* | *n=146,777* | *n=235,297* |
| Cheng (2016) | 0.925 (0.895-0.956) | 0.945 (0.903-0.989) | 0.905 (0.863-0.949) | 1.036 (0.851-1.262) | 0.938 (0.907-0.970) | 0.935 (0.888-0.984) | 0.916 (0.871-0.962) | 0.927 (0.847-1.014) |
| Qian (2016) | 1.104 (1.069-1.141) | 1.127 (1.078-1.179) | 1.081 (1.031-1.133) | 0.966 (0.792-1.178) | 1.132 (1.094-1.170) | 1.113 (1.058-1.170) | 1.122 (1.069-1.179) | 0.982 (0.897-1.074) |
| Dai (2019) | 1.146 (1.110-1.184) | 1.137 (1.087-1.189) | 1.157 (1.104-1.213) | 1.268 (1.042-1.544) | 1.167 (1.129-1.207) | 1.092 (1.038-1.149) | 1.195 (1.138-1.255) | 1.255 (1.148-1.371) |
| Shi (2019) | 1.123 (1.087-1.160) | 1.123 (1.074-1.175) | 1.122 (1.071-1.177) | 1.039 (0.853-1.265) | 1.116 (1.080-1.154) | 1.157 (1.100-1.217) | 1.142 (1.087-1.199) | 1.007 (0.920-1.102) |
| Jia (2020) | 1.276 (1.234-1.318) | 1.260 (1.204-1.319) | 1.292 (1.232-1.355) | 1.099 (0.904-1.336) | 1.269 (1.227-1.312) | 1.291 (1.227-1.359) | 1.299 (1.236-1.364) | 1.142 (1.044-1.250) |
| Kachuri (2020) | 1.199 (1.162-1.237) | 1.213 (1.162-1.266) | 1.183 (1.131-1.238) | 1.166 (0.966-1.407) | 1.187 (1.149-1.225) | 1.258 (1.199-1.319) | 1.180 (1.126-1.237) | 1.094 (1.002-1.195) |
| Yu (2020) | 1.260 (1.220-1.301) | 1.249 (1.195-1.306) | 1.271 (1.213-1.332) | 1.126 (0.928-1.365) | 1.250 (1.209-1.291) | 1.282 (1.219-1.348) | 1.271 (1.211-1.334) | 1.157 (1.058-1.264) |
| Graff (2021) | 1.199 (1.162-1.237) | 1.213 (1.162-1.266) | 1.183 (1.131-1.238) | 1.166 (0.966-1.407) | 1.187 (1.149-1.225) | 1.258 (1.199-1.319) | 1.180 (1.126-1.237) | 1.094 (1.002-1.195) |
| Huang (2021) | 0.816 (0.791-0.843) | 0.819 (0.784-0.857) | 0.813 (0.776-0.852) | 0.859 (0.709-1.042) | 0.819 (0.793-0.847) | 0.797 (0.758-0.838) | 0.798 (0.760-0.838) | 0.946 (0.865-1.035) |
| Hung (2021) | 1.264 (1.224-1.306) | 1.250 (1.195-1.307) | 1.279 (1.221-1.341) | 1.024 (0.841-1.247) | 1.270 (1.229-1.313) | 1.257 (1.196-1.322) | 1.299 (1.237-1.364) | 1.183 (1.082-1.293) |
| Jia (2021) | 1.276 (1.234-1.318) | 1.260 (1.204-1.319) | 1.292 (1.232-1.355) | 1.099 (0.904-1.336) | 1.269 (1.227-1.312) | 1.291 (1.227-1.359) | 1.299 (1.236-1.364) | 1.142 (1.044-1.250) |
| Wang (2021) | 0.821 (0.795-0.848) | 0.822 (0.786-0.859) | 0.820 (0.782-0.860) | 0.841 (0.693-1.021) | 0.822 (0.795-0.850) | 0.817 (0.777-0.859) | 0.794 (0.756-0.833) | 0.922 (0.843-1.008) |
| Xie (2021) | 1.225 (1.186-1.265) | 1.220 (1.167-1.276) | 1.231 (1.174-1.289) | 1.164 (0.961-1.411) | 1.221 (1.181-1.261) | 1.255 (1.194-1.319) | 1.253 (1.194-1.315) | 1.057 (0.966-1.156) |
| Barnett (2022) | 1.240 (1.200-1.281) | 1.238 (1.184-1.295) | 1.241 (1.184-1.301) | 1.190 (0.980-1.445) | 1.234 (1.194-1.276) | 1.248 (1.186-1.312) | 1.269 (1.208-1.333) | 1.119 (1.023-1.224) |
| J. Choi (2022a) | 1.275 (1.234-1.318) | 1.261 (1.205-1.319) | 1.292 (1.232-1.355) | 1.099 (0.904-1.337) | 1.269 (1.227-1.312) | 1.290 (1.226-1.358) | 1.299 (1.236-1.365) | 1.142 (1.044-1.249) |
| J. Choi (2022b) | 1.266 (1.225-1.308) | 1.249 (1.194-1.307) | 1.284 (1.225-1.347) | 1.086 (0.893-1.320) | 1.260 (1.218-1.303) | 1.277 (1.213-1.344) | 1.286 (1.224-1.351) | 1.156 (1.057-1.265) |
| J. Choi (2022c) | 1.275 (1.234-1.318) | 1.261 (1.205-1.319) | 1.292 (1.232-1.355) | 1.099 (0.904-1.337) | 1.269 (1.227-1.312) | 1.290 (1.226-1.358) | 1.299 (1.236-1.365) | 1.142 (1.044-1.249) |
| Liu (2022a) | 1.219 (1.180-1.259) | 1.201 (1.149-1.257) | 1.238 (1.181-1.298) | 1.105 (0.909-1.342) | 1.215 (1.175-1.256) | 1.204 (1.145-1.266) | 1.254 (1.194-1.316) | 1.177 (1.077-1.287) |
| Liu (2022b) | 1.202 (1.164-1.241) | 1.198 (1.147-1.252) | 1.205 (1.150-1.261) | 1.259 (1.043-1.519) | 1.190 (1.152-1.229) | 1.255 (1.195-1.318) | 1.198 (1.142-1.257) | 1.092 (0.999-1.193) |
| Qin (2022) | 1.182 (1.144-1.220) | 1.163 (1.112-1.215) | 1.202 (1.148-1.260) | 1.190 (0.981-1.443) | 1.186 (1.147-1.225) | 1.140 (1.085-1.199) | 1.213 (1.156-1.273) | 1.231 (1.128-1.343) |
| X. Wang (2022) | 0.823 (0.797-0.850) | 0.832 (0.795-0.870) | 0.814 (0.776-0.853) | 0.883 (0.728-1.071) | 0.824 (0.798-0.852) | 0.810 (0.770-0.851) | 0.802 (0.764-0.842) | 0.936 (0.855-1.023) |
| P. Zhang (2022) | 1.179 (1.141-1.218) | 1.171 (1.120-1.225) | 1.188 (1.133-1.245) | 1.207 (0.991-1.468) | 1.183 (1.144-1.223) | 1.190 (1.131-1.251) | 1.196 (1.139-1.257) | 1.141 (1.044-1.248) |
| R. Zhang (2022) | 1.234 (1.195-1.273) | 1.225 (1.173-1.280) | 1.242 (1.187-1.300) | 1.123 (0.926-1.361) | 1.227 (1.188-1.267) | 1.273 (1.212-1.336) | 1.226 (1.169-1.285) | 1.150 (1.054-1.255) |
| Blechter (2023) | 1.007 (0.975-1.041) | 1.012 (0.967-1.060) | 1.003 (0.956-1.052) | 1.115 (0.916-1.357) | 0.998 (0.965-1.032) | 0.997 (0.948-1.050) | 1.022 (0.973-1.074) | 1.002 (0.915-1.096) |
| Kim (2023) | 1.213 (1.176-1.251) | 1.227 (1.176-1.280) | 1.197 (1.144-1.252) | 1.199 (0.998-1.442) | 1.196 (1.159-1.235) | 1.269 (1.210-1.331) | 1.193 (1.139-1.251) | 1.109 (1.017-1.210) |
| Liang (2023) | 0.835 (0.808-0.863) | 0.847 (0.810-0.886) | 0.822 (0.785-0.862) | 0.886 (0.729-1.076) | 0.832 (0.805-0.860) | 0.847 (0.805-0.891) | 0.803 (0.765-0.843) | 0.897 (0.821-0.981) |
| Shi (2023a) | 1.091 (1.056-1.128) | 1.077 (1.029-1.127) | 1.107 (1.056-1.161) | 1.040 (0.854-1.266) | 1.091 (1.055-1.128) | 1.075 (1.022-1.132) | 1.095 (1.042-1.151) | 1.182 (1.081-1.293) |
| Shi (2023b) | 1.080 (1.045-1.116) | 1.058 (1.011-1.107) | 1.104 (1.052-1.157) | 1.033 (0.849-1.258) | 1.071 (1.035-1.107) | 1.056 (1.004-1.112) | 1.085 (1.033-1.140) | 1.163 (1.063-1.273) |
| Shi (2023c) | 1.069 (1.034-1.104) | 1.047 (1.001-1.096) | 1.092 (1.042-1.146) | 1.056 (0.867-1.285) | 1.062 (1.027-1.099) | 1.048 (0.995-1.103) | 1.072 (1.020-1.126) | 1.153 (1.054-1.261) |
| Wang (2023) | 1.276 (1.234-1.318) | 1.260 (1.204-1.319) | 1.292 (1.232-1.355) | 1.099 (0.904-1.336) | 1.269 (1.227-1.312) | 1.291 (1.227-1.359) | 1.299 (1.236-1.364) | 1.142 (1.044-1.250) |
| Wei (2023a) | 1.092 (1.056-1.128) | 1.079 (1.031-1.129) | 1.105 (1.054-1.159) | 1.114 (0.917-1.352) | 1.087 (1.052-1.124) | 1.063 (1.010-1.118) | 1.110 (1.057-1.166) | 1.165 (1.066-1.274) |
| Wei (2023b) | 1.094 (1.058-1.130) | 1.079 (1.031-1.129) | 1.110 (1.059-1.164) | 1.157 (0.953-1.404) | 1.097 (1.061-1.134) | 1.059 (1.006-1.114) | 1.115 (1.061-1.171) | 1.198 (1.096-1.309) |
| Xiao (2023) | 1.116 (1.082-1.150) | 1.129 (1.082-1.178) | 1.100 (1.052-1.151) | 1.119 (0.936-1.339) | 1.104 (1.070-1.139) | 1.150 (1.097-1.205) | 1.101 (1.051-1.154) | 1.075 (0.986-1.171) |
| Xin (2023) | 1.219 (1.180-1.259) | 1.226 (1.172-1.283) | 1.212 (1.156-1.270) | 1.158 (0.953-1.406) | 1.218 (1.179-1.259) | 1.216 (1.156-1.279) | 1.267 (1.207-1.331) | 1.089 (0.996-1.192) |
| Zhang (2023) | 1.217 (1.178-1.257) | 1.216 (1.163-1.272) | 1.219 (1.163-1.278) | 1.189 (0.979-1.444) | 1.216 (1.177-1.257) | 1.222 (1.162-1.285) | 1.260 (1.200-1.323) | 1.085 (0.992-1.187) |
| Zhu (2023a) | 1.124 (1.088-1.161) | 1.105 (1.057-1.156) | 1.146 (1.093-1.201) | 1.191 (0.978-1.450) | 1.152 (1.114-1.191) | 1.063 (1.011-1.119) | 1.184 (1.128-1.244) | 1.228 (1.124-1.342) |
| Zhu (2023b) | 1.239 (1.200-1.280) | 1.217 (1.164-1.272) | 1.265 (1.207-1.325) | 1.069 (0.879-1.300) | 1.251 (1.210-1.293) | 1.228 (1.168-1.291) | 1.284 (1.223-1.347) | 1.166 (1.066-1.274) |
| Zhu (2023c) | 1.194 (1.155-1.233) | 1.179 (1.127-1.233) | 1.211 (1.156-1.270) | 1.163 (0.956-1.414) | 1.223 (1.183-1.264) | 1.161 (1.104-1.222) | 1.261 (1.201-1.324) | 1.181 (1.081-1.292) |

**S14 Percentiles - whole cohort**

| PGS n=429,665 | Percentage of cases found in top % of score | | | | | | | | |
|---|---|---|---|---|---|---|---|---|---|
| | 1% | 5% | 10% | 20% | 50% | 80% | 90% | 95% | 99% |
| *Cheng (2016)* | 1.2 | 6.2 | 11 | 20.9 | 53.1 | 82.6 | 91.6 | 96.3 | 99.5 |
| *Qian (2016)* | 0.8 | 5.6 | 11.4 | 22.6 | 54.2 | 83.5 | 91.7 | 95.8 | 99.2 |
| *Dai (2019)* | 1.6 | 6.6 | 12.4 | 23.6 | 55.9 | 83.2 | 92.5 | 96.3 | 99.5 |
| *Shi (2019)* | 1.1 | 5.9 | 12.3 | 23.5 | 54.8 | 83.2 | 91.2 | 95.1 | 99.2 |
| *Jia (2020)* | 1.4 | 7.6 | 14.4 | 27.2 | 60.2 | 86.5 | 94.5 | 97.5 | 99.5 |
| *Kachuri (2020)* | 1.6 | 6.7 | 13 | 25.1 | 58.1 | 85.3 | 93.4 | 97.3 | 99.4 |
| *Yu (2020)* | 1.7 | 7.5 | 15.1 | 27.4 | 58.8 | 86.7 | 94.3 | 97.2 | 99.7 |
| *Graff (2021)* | 1.6 | 6.7 | 13 | 25.1 | 58.1 | 85.3 | 93.4 | 97.3 | 99.4 |
| *Huang (2021)* | 1.5 | 7.3 | 13.5 | 26.1 | 57.6 | 86 | 93.4 | 96.9 | 99.5 |
| *Hung (2021)* | 1.6 | 7.5 | 15 | 27.1 | 59 | 86.7 | 93.7 | 97.2 | 99.5 |
| *Jia (2021)* | 1.4 | 7.6 | 14.4 | 27.2 | 60.2 | 86.5 | 94.5 | 97.5 | 99.5 |
| *Wang (2021)* | 1.7 | 7.1 | 13.6 | 25.8 | 57.6 | 84.9 | 92.9 | 97 | 99.5 |
| *Xie (2021)* | 1.5 | 7.3 | 13.4 | 26 | 57.6 | 86 | 93.4 | 96.8 | 99.5 |
| *Barnett (2022)* | 1.7 | 7.5 | 14 | 26.4 | 57.9 | 85.9 | 93.4 | 97.3 | 99.5 |
| *J. Choi (2022a)* | 1.4 | 7.6 | 14.5 | 27.1 | 59.6 | 86.6 | 94.3 | 97.5 | 99.6 |
| *J. Choi (2022b)* | 1.4 | 7.1 | 14.1 | 26.7 | 58.7 | 86.7 | 94 | 97.1 | 99.5 |
| *J. Choi (2022c)* | 1.4 | 7.6 | 14.5 | 27.1 | 59.6 | 86.6 | 94.3 | 97.5 | 99.6 |
| *Liu (2022a)* | 1.4 | 6.9 | 13.2 | 26.6 | 58.1 | 85.1 | 93 | 96.8 | 99.5 |
| *Liu (2022b)* | 1.5 | 6.7 | 12.4 | 25.4 | 56.5 | 85 | 92.8 | 96.5 | 99.4 |
| *Qin (2022)* | 1.5 | 7.1 | 13.3 | 25.3 | 57 | 84.3 | 92.6 | 96 | 99.2 |
| *X. Wang (2022)* | 1.6 | 7.2 | 14.2 | 25.6 | 57.6 | 85.5 | 93.5 | 97 | 99.4 |
| *P. Zhang (2022)* | 1.8 | 6.8 | 13.1 | 24.3 | 56.4 | 85.1 | 93 | 96.2 | 99.2 |
| *R. Zhang (2022)* | 1.7 | 7.8 | 14.3 | 25.8 | 58.4 | 86.1 | 93.7 | 97 | 99.6 |
| *Blechter (2023)* | 0.9 | 5.2 | 9.7 | 19 | 50.4 | 80.8 | 90.9 | 95.6 | 99.4 |
| *Kim (2023)* | 1.5 | 7 | 13.3 | 26.1 | 58.5 | 85.7 | 93.2 | 96.8 | 99.4 |
| *Liang (2023)* | 1.6 | 7 | 13.3 | 25.5 | 57 | 85 | 93.1 | 96.6 | 99.3 |
| *Shi (2023a)* | 1.4 | 5.8 | 11.6 | 22.2 | 54.2 | 82.8 | 91 | 95.5 | 99.1 |
| *Shi (2023b)* | 1 | 6 | 11.1 | 21.8 | 53.5 | 83.1 | 92 | 96.2 | 99.2 |
| *Shi (2023c)* | 1 | 5.8 | 11.2 | 21.4 | 52.3 | 82.7 | 91.8 | 95.9 | 99 |
| *Wang (2023)* | 1.4 | 7.6 | 14.4 | 27.2 | 60.2 | 86.5 | 94.5 | 97.5 | 99.5 |
| *Wei (2023a)* | 1.3 | 6.1 | 12 | 22.3 | 53.1 | 82.5 | 91.6 | 95.9 | 99.4 |
| *Wei (2023b)* | 1.3 | 6.4 | 11.6 | 23.1 | 53.2 | 82 | 91.5 | 96.4 | 99.4 |
| *Xiao (2023)* | 1.4 | 6.6 | 12.5 | 23 | 55.1 | 82.9 | 92.3 | 96.2 | 99.4 |
| *Xin (2023)* | 1.8 | 7.1 | 13.3 | 25.6 | 57.4 | 84.9 | 92.9 | 97.1 | 99.5 |
| *Zhang (2023)* | 1.7 | 7.1 | 13.6 | 25.8 | 57.6 | 84.9 | 92.9 | 97 | 99.5 |
| *Zhu (2023a)* | 1.3 | 6 | 11.6 | 23 | 55.4 | 83.3 | 91.8 | 96.1 | 99.3 |
| *Zhu (2023b)* | 1.5 | 7.3 | 14.2 | 26.4 | 58.5 | 85.9 | 93.8 | 97.1 | 99.6 |
| *Zhu (2023c)* | 1 | 6.8 | 13.2 | 25 | 57.2 | 85.7 | 93.6 | 96.5 | 99.1 |

## S15 Percentiles - Women Only

| PGS | Percentage of cases found in top % of score | | | | | | | | |
|---|---|---|---|---|---|---|---|---|---|
| n=226,951 | 1% | 5% | 10% | 20% | 50% | 80% | 90% | 95% | 99% |
| *Cheng (2016)* | 1.3 | 6.6 | 11.6 | 22.6 | 53.9 | 83.5 | 91.6 | 96 | 99.3 |
| *Qian (2016)* | 0.7 | 5.2 | 11.8 | 23.1 | 52.7 | 83 | 92.1 | 95.7 | 99.1 |
| *Dai (2019)* | 1.8 | 6.5 | 12.2 | 23.4 | 56.9 | 83.5 | 92.7 | 96.3 | 99.5 |
| *Shi (2019)* | 1.1 | 6.2 | 11.8 | 22.9 | 54.9 | 83.2 | 91.9 | 95.5 | 99.2 |
| *Jia (2020)* | 1.6 | 8.1 | 15 | 28.4 | 61.3 | 86.1 | 94.4 | 97.4 | 99.5 |
| *Kachuri (2020)* | 1.2 | 6.5 | 13.3 | 24.9 | 58.2 | 85 | 93 | 97.1 | 99.5 |
| *Yu (2020)* | 1.9 | 8.1 | 15.7 | 28.2 | 59 | 86.3 | 93.9 | 97.2 | 99.6 |
| *Graff (2021)* | 1.2 | 6.5 | 13.3 | 24.9 | 58.2 | 85 | 93 | 97.1 | 99.5 |
| *Huang (2021)* | 1.7 | 7.9 | 14 | 26.8 | 57.5 | 85.3 | 93.4 | 96.3 | 99.4 |
| *Hung (2021)* | 1.7 | 7.5 | 15.3 | 27.4 | 59.9 | 86.6 | 93.7 | 97.1 | 99.6 |
| *Jia (2021)* | 1.6 | 8.1 | 15 | 28.4 | 61.3 | 86.1 | 94.4 | 97.4 | 99.5 |
| *Wang (2021)* | 2 | 7.2 | 14.1 | 26.2 | 57.8 | 84.1 | 92.7 | 96.8 | 99.3 |
| *Xie (2021)* | 1.7 | 7.8 | 14 | 26.7 | 57.6 | 85.2 | 93.4 | 96.3 | 99.4 |
| *Barnett (2022)* | 2 | 7.6 | 15.1 | 26.5 | 58.2 | 85.3 | 93.1 | 97 | 99.3 |
| *J. Choi (2022a)* | 1.6 | 7.9 | 14.9 | 28.4 | 60.7 | 86.2 | 94.3 | 97.5 | 99.5 |
| *J. Choi (2022b)* | 1.5 | 7.7 | 14.8 | 27.7 | 59.1 | 86.4 | 94 | 97.3 | 99.4 |
| *J. Choi (2022c)* | 1.6 | 7.9 | 14.9 | 28.4 | 60.7 | 86.2 | 94.3 | 97.5 | 99.5 |
| *Liu (2022a)* | 1.7 | 7.2 | 13.9 | 26.7 | 58.9 | 85.1 | 92.9 | 96.6 | 99.6 |
| *Liu (2022b)* | 1.4 | 7.1 | 12.7 | 25.7 | 56.2 | 84.7 | 93 | 96.7 | 99.2 |
| *Qin (2022)* | 1.6 | 7.5 | 14.1 | 25.5 | 57.3 | 83.8 | 93.5 | 96.6 | 99.2 |
| *X. Wang (2022)* | 1.5 | 7.3 | 14.8 | 27 | 58.5 | 84.7 | 93.7 | 96.7 | 99.2 |
| *P. Zhang (2022)* | 2.3 | 7.3 | 13.9 | 24.3 | 57.1 | 85.2 | 92.8 | 95.4 | 98.9 |
| *R. Zhang (2022)* | 1.8 | 8.2 | 14.9 | 25.9 | 59.2 | 86.2 | 94.1 | 97.2 | 99.6 |
| *Blechter (2023)* | 0.8 | 4.5 | 9.2 | 18.7 | 49.5 | 81.2 | 91.1 | 96.1 | 99.6 |
| *Kim (2023)* | 1.4 | 6.8 | 13.2 | 26.2 | 57.8 | 84.8 | 92.8 | 96.4 | 99.5 |
| *Liang (2023)* | 1.7 | 7.5 | 13.9 | 26.5 | 57.6 | 84.6 | 93.1 | 96.4 | 99.2 |
| *Shi (2023a)* | 1.2 | 5.4 | 11.7 | 22.3 | 54.5 | 83.4 | 91.4 | 95.7 | 99.2 |
| *Shi (2023b)* | 1.1 | 5.7 | 11.2 | 22.5 | 53 | 84.2 | 92.7 | 96.6 | 99.5 |
| *Shi (2023c)* | 1.1 | 5.9 | 11 | 22.3 | 53 | 83.7 | 92.2 | 96.3 | 99.2 |
| *Wang (2023)* | 1.6 | 8.1 | 15 | 28.4 | 61.3 | 86.1 | 94.4 | 97.4 | 99.5 |
| *Wei (2023a)* | 1.6 | 6.6 | 13.3 | 23.2 | 52.8 | 81.9 | 91.8 | 95.9 | 99.5 |
| *Wei (2023b)* | 1.6 | 7.2 | 12.4 | 23.9 | 52.5 | 82 | 91.1 | 96.1 | 99.5 |
| *Xiao (2023)* | 1.2 | 6.2 | 12.1 | 22.7 | 55.2 | 82.2 | 91.7 | 96.1 | 99.5 |
| *Xin (2023)* | 1.7 | 7.2 | 13.6 | 25.5 | 57.4 | 84.1 | 92.8 | 96.9 | 99.3 |
| *Zhang (2023)* | 1.9 | 7.2 | 14.1 | 26.2 | 57.6 | 84 | 92.7 | 96.7 | 99.2 |
| *Zhu (2023a)* | 1.4 | 5.3 | 11.7 | 23.6 | 56.1 | 84.1 | 92.1 | 96.2 | 99.4 |
| *Zhu (2023b)* | 1.7 | 7.9 | 15.4 | 27.9 | 58.9 | 85.7 | 93.8 | 96.6 | 99.7 |
| *Zhu (2023c)* | 1.1 | 6.8 | 14.5 | 25.8 | 57.9 | 86.1 | 93.4 | 96.6 | 99.1 |

**S16 Percentiles - Men Only**

| PGS | % of cases found in percentiles of PGS | | | | | | | | |
|---|---|---|---|---|---|---|---|---|---|
| n=202,714 | *top 1%* | *top 5%* | *top 10%* | *top 20%* | *top 50%* | *top 80%* | *top 90%* | *top 95%* | *top 99%* |
| *Cheng (2016)* | 1.2 | 5.9 | 10.5 | 19.5 | 52.2 | 81.9 | 91.5 | 96.3 | 99.6 |
| *Qian (2016)* | 1 | 5.8 | 11.2 | 22.3 | 55.7 | 83.9 | 91.4 | 96 | 99.4 |
| *Dai (2019)* | 1.3 | 6.7 | 12.6 | 23.8 | 54.9 | 82.9 | 92.2 | 96.2 | 99.5 |
| *Shi (2019)* | 1.2 | 5.7 | 12.9 | 24 | 54.7 | 83.2 | 90.4 | 94.8 | 99.1 |
| *Jia (2020)* | 1.3 | 7.2 | 13.8 | 26.2 | 59 | 86.8 | 94.5 | 97.5 | 99.7 |
| *Kachuri (2020)* | 1.8 | 6.8 | 12.7 | 25.4 | 57.6 | 85.8 | 93.9 | 97.5 | 99.2 |
| *Yu (2020)* | 1.5 | 6.9 | 14.5 | 26.6 | 58.5 | 87 | 94.6 | 97.3 | 99.8 |
| *Graff (2021)* | 1.8 | 6.8 | 12.7 | 25.4 | 57.6 | 85.8 | 93.9 | 97.5 | 99.2 |
| *Huang (2021)* | 1.3 | 6.9 | 13 | 25.4 | 57.6 | 86.6 | 93.4 | 97.4 | 99.6 |
| *Hung (2021)* | 1.7 | 7.5 | 14.5 | 26.9 | 58.1 | 86.8 | 93.7 | 97.2 | 99.5 |
| *Jia (2021)* | 1.3 | 7.2 | 13.8 | 26.2 | 59 | 86.8 | 94.5 | 97.5 | 99.7 |
| *Wang (2021)* | 1.6 | 7 | 12.9 | 25.3 | 57.4 | 85.6 | 93 | 97.1 | 99.7 |
| *Xie (2021)* | 1.3 | 6.9 | 12.7 | 25.5 | 57.6 | 86.6 | 93.5 | 97.4 | 99.6 |
| *Barnett (2022)* | 1.5 | 7.3 | 13.1 | 26.2 | 57.6 | 86.3 | 93.7 | 97.5 | 99.7 |
| *J. Choi (2022a)* | 1.3 | 7.3 | 14.1 | 25.9 | 58.7 | 86.9 | 94.5 | 97.5 | 99.7 |
| *J. Choi (2022b)* | 1.3 | 6.6 | 13.6 | 25.8 | 58.4 | 86.9 | 94 | 96.9 | 99.7 |
| *J. Choi (2022c)* | 1.3 | 7.3 | 14.1 | 25.9 | 58.7 | 86.9 | 94.5 | 97.5 | 99.7 |
| *Liu (2022a)* | 1.2 | 6.7 | 12.7 | 26.4 | 57.3 | 85.1 | 93.1 | 96.8 | 99.5 |
| *Liu (2022b)* | 0.9 | 6.3 | 12.1 | 25.3 | 56.8 | 85.3 | 92.7 | 96.3 | 99.6 |
| *Qin (2022)* | 1.4 | 6.8 | 12.4 | 25.1 | 56.7 | 84.8 | 91.7 | 95.5 | 99.3 |
| *X. Wang (2022)* | 1.7 | 7 | 13.6 | 24.3 | 56.7 | 86.2 | 93.3 | 97.5 | 99.7 |
| *P. Zhang (2022)* | 1.4 | 6.1 | 12.4 | 24.1 | 56.1 | 84.9 | 93.2 | 96.9 | 99.5 |
| *R. Zhang (2022)* | 1.6 | 7.4 | 13.7 | 25.6 | 57.4 | 86.1 | 93.4 | 96.7 | 99.5 |
| *Blechter (2023)* | 1 | 5.9 | 10.2 | 19.2 | 51.3 | 80.5 | 90.7 | 94.9 | 99.4 |
| *Kim (2023)* | 1.6 | 7.1 | 13.4 | 26 | 59.2 | 86.5 | 93.5 | 97.2 | 99.4 |
| *Liang (2023)* | 1.6 | 6.5 | 12.7 | 24.6 | 56.6 | 85.5 | 93.2 | 97 | 99.4 |
| *Shi (2023a)* | 1.5 | 6.2 | 11.4 | 22.1 | 54 | 82.2 | 90.7 | 95.3 | 99 |
| *Shi (2023b)* | 1 | 6.1 | 11 | 21.1 | 54 | 82.1 | 91.4 | 95.8 | 99 |
| *Shi (2023c)* | 0.9 | 5.7 | 11.4 | 20.6 | 51.7 | 81.8 | 91.4 | 95.6 | 98.8 |
| *Wang (2023)* | 1.3 | 7.2 | 13.8 | 26.2 | 59 | 86.8 | 94.5 | 97.5 | 99.7 |
| *Wei (2023a)* | 1.1 | 5.6 | 10.9 | 21.4 | 53.3 | 83.1 | 91.4 | 96 | 99.2 |
| *Wei (2023b)* | 1 | 5.7 | 10.9 | 22.3 | 53.8 | 81.9 | 92 | 96.7 | 99.4 |
| *Xiao (2023)* | 1.5 | 7 | 12.8 | 23.4 | 55.1 | 83.7 | 92.9 | 96.2 | 99.4 |
| *Xin (2023)* | 1.8 | 7 | 13.2 | 25.7 | 57.4 | 85.5 | 93 | 97.2 | 99.8 |
| *Zhang (2023)* | 1.5 | 7 | 13 | 25.3 | 57.5 | 85.7 | 93 | 97.1 | 99.7 |
| *Zhu (2023a)* | 1.1 | 6.5 | 11.5 | 22.4 | 54.9 | 82.7 | 91.6 | 96 | 99.2 |
| *Zhu (2023b)* | 1.3 | 6.6 | 13.1 | 25.1 | 58.2 | 86.1 | 93.8 | 97.6 | 99.5 |
| *Zhu (2023c)* | 1 | 6.7 | 12.4 | 24.3 | 56.5 | 85.3 | 93.8 | 96.6 | 99.1 |

## S17 Percentiles - White Only

| PGS n=402,318 | Percentage of cases found in top % of score | | | | | | | | |
|---|---|---|---|---|---|---|---|---|---|
| | 1% | 5% | 10% | 20% | 50% | 80% | 90% | 95% | 99% |
| *Cheng (2016)* | 1.2 | 6.1 | 11 | 20.7 | 52.6 | 82.4 | 91.2 | 95.8 | 99.3 |
| *Qian (2016)* | 1.1 | 6.3 | 12.3 | 23.6 | 55 | 83.5 | 91.7 | 95.8 | 99.2 |
| *Dai (2019)* | 1.8 | 7.1 | 13 | 24.2 | 56 | 83.5 | 92.5 | 96.3 | 99.5 |
| *Shi (2019)* | 1.1 | 5.6 | 12.2 | 23.3 | 54.7 | 81.1 | 91.3 | 95.2 | 99.2 |
| *Jia (2020)* | 1.4 | 7.6 | 14.3 | 27.1 | 59.9 | 86.3 | 94.2 | 97.4 | 99.5 |
| *Kachuri (2020)* | 1.4 | 6.6 | 12.8 | 24.5 | 57.6 | 85.4 | 93.5 | 97.3 | 99.4 |
| *Yu (2020)* | 1.7 | 7.3 | 14.8 | 26.9 | 58.4 | 86.4 | 94.2 | 97.2 | 99.7 |
| *Graff (2021)* | 1.4 | 6.6 | 12.8 | 24.5 | 57.6 | 85.4 | 93.5 | 97.3 | 99.4 |
| *Huang (2021)* | 1.5 | 7.3 | 13.4 | 26 | 57.7 | 86.1 | 93.4 | 96.8 | 99.5 |
| *Hung (2021)* | 1.6 | 7.6 | 15.1 | 27.2 | 59.1 | 86.8 | 93.7 | 97.2 | 99.5 |
| *Jia (2021)* | 1.4 | 7.6 | 14.3 | 27.1 | 59.9 | 86.3 | 94.2 | 97.4 | 99.5 |
| *Wang (2021)* | 1.7 | 7.1 | 13.5 | 25.5 | 57.6 | 84.9 | 92.8 | 96.9 | 99.5 |
| *Xie (2021)* | 1.5 | 7.3 | 13.3 | 25.9 | 57.6 | 86.1 | 93.5 | 96.7 | 99.5 |
| *Barnett (2022)* | 1.8 | 7.4 | 13.9 | 26.3 | 57.9 | 85.6 | 93.3 | 97.2 | 99.5 |
| *J. Choi (2022a)* | 1.5 | 7.5 | 14.4 | 27.2 | 59.8 | 86.2 | 94.2 | 97.4 | 99.5 |
| *J. Choi (2022b)* | 1.4 | 7.1 | 14.3 | 26.5 | 59 | 86.5 | 93.8 | 97 | 99.5 |
| *J. Choi (2022c)* | 1.5 | 7.5 | 14.4 | 27.2 | 59.8 | 86.2 | 94.2 | 97.4 | 99.5 |
| *Liu (2022a)* | 1.4 | 6.9 | 13.3 | 26.6 | 57.9 | 84.9 | 93 | 96.7 | 99.5 |
| *Liu (2022b)* | 1.1 | 6.6 | 12.5 | 24.8 | 56.8 | 82.6 | 92.3 | 96.5 | 99.4 |
| *L. Wang (2022)* | 1.6 | 6.6 | 12.7 | 26 | 57.5 | 84.8 | 93.4 | 97 | 99.5 |
| *X. Wang (2022)* | 1.6 | 6.9 | 14.1 | 25.4 | 57.6 | 85.6 | 93.4 | 97 | 99.4 |
| *P. Zhang (2022)* | 1.8 | 6.9 | 13.1 | 24.3 | 56.5 | 85.2 | 93 | 96.2 | 99.2 |
| *R. Zhang (2022)* | 1.7 | 7.5 | 13.9 | 25.4 | 58.3 | 86.2 | 93.8 | 97 | 99.6 |
| *Blechter (2023)* | 0.9 | 5 | 9.6 | 18.8 | 49.9 | 80.5 | 90.5 | 95.3 | 99.4 |
| *Kim (2023)* | 1.4 | 6.7 | 13 | 25.6 | 58.1 | 85.6 | 93.2 | 96.8 | 99.5 |
| *Liang (2023)* | 1.6 | 7 | 13.3 | 25.5 | 57.3 | 85 | 93.1 | 96.7 | 99.3 |
| *Shi (2023a)* | 1.4 | 5.8 | 11.5 | 22.1 | 54.2 | 82.9 | 91.1 | 95.4 | 99.1 |
| *Shi (2023b)* | 1.1 | 5.8 | 10.8 | 21.4 | 53.1 | 83 | 92 | 96.1 | 99.2 |
| *Shi (2023c)* | 1 | 5.6 | 11.1 | 21.1 | 52.2 | 82.4 | 91.9 | 95.8 | 98.9 |
| *Wang (2023)* | 1.4 | 7.6 | 14.3 | 27.1 | 59.9 | 86.3 | 94.2 | 97.4 | 99.5 |
| *Wei (2023a)* | 1.4 | 6.2 | 12.1 | 22.3 | 52.9 | 82.3 | 91.3 | 95.9 | 99.3 |
| *Wei (2023b)* | 1.3 | 6.5 | 11.7 | 23.1 | 53.4 | 82.1 | 91.7 | 96.4 | 99.4 |
| *Xiao (2023)* | 1.4 | 6.5 | 12.2 | 22.4 | 55.1 | 83.2 | 92.4 | 96.3 | 99.4 |
| *Xin (2023)* | 1.8 | 7.1 | 13.4 | 25.4 | 57.4 | 84.9 | 92.8 | 97 | 99.5 |
| *Zhang (2023)* | 1.7 | 7.1 | 13.4 | 25.5 | 57.6 | 84.9 | 92.8 | 96.9 | 99.5 |
| *Zhu (2023a)* | 1.7 | 6.4 | 12.7 | 24 | 56 | 83.5 | 92.2 | 96.2 | 99.4 |
| *Zhu (2023b)* | 1.6 | 7.4 | 14.3 | 26.7 | 59 | 86.4 | 94 | 97.3 | 99.6 |
| *Zhu (2023c)* | 1.2 | 7.2 | 14 | 25.6 | 57.9 | 86 | 93.6 | 96.6 | 99.1 |

**S18 Percentiles - Individuals who currently smoke**

| PGS | Percentage of cases found in top % of score | | | | | | | | |
|---|---|---|---|---|---|---|---|---|---|
| n=45,391 | 1% | 5% | 10% | 20% | 50% | 80% | 90% | 95% | 99% |
| *Cheng (2016)* | 1.2 | 5.9 | 10.5 | 20.7 | 52.6 | 82 | 91.5 | 96.2 | 99.3 |
| *Qian (2016)* | 0.5 | 5.2 | 11.1 | 23.1 | 54.8 | 83.6 | 91.7 | 96 | 99.5 |
| *Dai (2019)* | 1.1 | 5.9 | 11.3 | 21.8 | 54.8 | 81.9 | 91.7 | 95.8 | 99.5 |
| *Shi (2019)* | 1.3 | 5.7 | 12.6 | 24.6 | 56.1 | 83.9 | 91.5 | 95.3 | 99.1 |
| *Jia (2020)* | 1.6 | 7.8 | 14.8 | 26.7 | 60.4 | 86.6 | 95.4 | 97.7 | 99.7 |
| *Kachuri (2020)* | 1.8 | 7.1 | 13.8 | 26.5 | 60.4 | 86.6 | 93.7 | 97.5 | 99.5 |
| *Yu (2020)* | 1.7 | 7.5 | 15.4 | 27.8 | 58.7 | 86.6 | 94.4 | 97.5 | 99.9 |
| *Graff (2021)* | 1.8 | 7.1 | 13.8 | 26.5 | 60.4 | 86.6 | 93.7 | 97.5 | 99.5 |
| *Huang (2021)* | 1.6 | 7.4 | 13.9 | 26.1 | 58.7 | 87.3 | 94 | 97.2 | 99.7 |
| *Hung (2021)* | 1.8 | 7.8 | 14.7 | 27.4 | 58.2 | 86.4 | 93.9 | 97.4 | 99.6 |
| *Jia (2021)* | 1.6 | 7.8 | 14.8 | 26.7 | 60.4 | 86.6 | 95.4 | 97.7 | 99.7 |
| *Wang (2021)* | 1.8 | 6.9 | 13.4 | 24.7 | 57.5 | 85.9 | 93.4 | 97.3 | 99.7 |
| *Xie (2021)* | 1.7 | 7.2 | 13.7 | 25.9 | 58.9 | 87.2 | 93.9 | 97.3 | 99.7 |
| *Barnett (2022)* | 1.6 | 7 | 13.6 | 26.2 | 57.2 | 86.7 | 93.9 | 98 | 99.5 |
| *J. Choi (2022a)* | 1.6 | 7.8 | 14.9 | 26.5 | 59.8 | 86.7 | 95.1 | 97.8 | 99.7 |
| *J. Choi (2022b)* | 1.2 | 7.2 | 14.5 | 25.8 | 58.2 | 86.9 | 94.5 | 97.5 | 99.7 |
| *J. Choi (2022c)* | 1.6 | 7.8 | 14.9 | 26.5 | 59.8 | 86.7 | 95.1 | 97.8 | 99.7 |
| *Liu (2022a)* | 1.5 | 6.5 | 12.1 | 26.7 | 57.2 | 84.8 | 93 | 97.1 | 99.3 |
| *Liu (2022b)* | 1.9 | 7.2 | 12.8 | 25.6 | 60.4 | 86.2 | 93.7 | 97.5 | 99.7 |
| *Qin (2022)* | 1.4 | 6.9 | 12.7 | 24.8 | 55.2 | 82.5 | 92 | 95.7 | 99 |
| *X. Wang (2022)* | 1.4 | 6.9 | 14.3 | 25.6 | 58.3 | 86.4 | 93.7 | 97.3 | 99.6 |
| *P. Zhang (2022)* | 1.6 | 7.4 | 14.3 | 24.8 | 56 | 84.5 | 93 | 96 | 99.2 |
| *R. Zhang (2022)* | 2.1 | 8 | 15.2 | 26.7 | 59 | 86.6 | 94.3 | 97.6 | 99.7 |
| *Blechter (2023)* | 0.9 | 4.7 | 9.7 | 19.2 | 50.1 | 79.9 | 90 | 95.9 | 99.6 |
| *Kim (2023)* | 1.9 | 7.6 | 13.7 | 27.4 | 60.2 | 86.6 | 93.8 | 97.4 | 99.5 |
| *Liang (2023)* | 1.4 | 6.9 | 12.7 | 23.9 | 56.8 | 84.9 | 93.9 | 96.9 | 99.4 |
| *Shi (2023a)* | 0.9 | 4.8 | 10.3 | 21.6 | 53.7 | 83.8 | 91 | 95.5 | 99 |
| *Shi (2023b)* | 0.8 | 4.9 | 10 | 20.6 | 52.9 | 83.4 | 92 | 96.3 | 99.4 |
| *Shi (2023c)* | 1 | 5 | 10.4 | 20.5 | 51.6 | 82.7 | 91.6 | 96 | 99 |
| *Wang (2023)* | 1.6 | 7.8 | 14.8 | 26.7 | 60.4 | 86.6 | 95.4 | 97.7 | 99.7 |
| *Wei (2023a)* | 1.3 | 5.7 | 11.1 | 21.3 | 51.5 | 81.9 | 91.5 | 95.7 | 99.2 |
| *Wei (2023b)* | 1.1 | 6 | 10.6 | 22 | 52 | 81.1 | 90.5 | 96.2 | 99.5 |
| *Xiao (2023)* | 1.8 | 7.2 | 13.1 | 24.4 | 55.5 | 82.5 | 92.2 | 96.5 | 99.6 |
| *Xin (2023)* | 1.8 | 6.6 | 13 | 24.2 | 57 | 85.7 | 93.3 | 97.3 | 99.6 |
| *Zhang (2023)* | 1.7 | 6.8 | 13.2 | 24.7 | 57.2 | 85.8 | 93.3 | 97.3 | 99.5 |
| *Zhu (2023a)* | 0.8 | 4.8 | 10 | 20.8 | 54.4 | 82.5 | 91.1 | 95.8 | 99.3 |
| *Zhu (2023b)* | 1.2 | 7.5 | 14.5 | 26.6 | 58.5 | 84.6 | 93.7 | 96.9 | 99.6 |
| *Zhu (2023c)* | 0.8 | 6 | 12.6 | 24.8 | 56.5 | 83.8 | 92.4 | 95.8 | 99 |

## S19 Percentiles - Individuals with former tobacco use

| PGS | Percentage of cases found in top % of score | | | | | | | | |
|---|---|---|---|---|---|---|---|---|---|
| n=146,777 | 1% | 5% | 10% | 20% | 50% | 80% | 90% | 95% | 99% |
| *Cheng (2016)* | 1.1 | 6.8 | 12 | 22.2 | 53.5 | 83.2 | 91.2 | 95.8 | 99.3 |
| *Qian (2016)* | 1.3 | 5.6 | 11.2 | 22.7 | 55.1 | 83.7 | 92.3 | 95.9 | 99 |
| *Dai (2019)* | 1.9 | 6.8 | 13.6 | 25.2 | 55.8 | 84.4 | 93.1 | 96.7 | 99.5 |
| *Shi (2019)* | 1.2 | 7 | 12.8 | 23.2 | 54.6 | 83.7 | 91.4 | 95.1 | 99.3 |
| *Jia (2020)* | 1.3 | 7.7 | 14.3 | 27.8 | 61.4 | 86.8 | 94.5 | 97.2 | 99.4 |
| *Kachuri (2020)* | 1.4 | 6.7 | 13 | 24.3 | 56.9 | 84.9 | 93.9 | 97.4 | 99.4 |
| *Yu (2020)* | 1.5 | 7.6 | 15 | 27.4 | 59.5 | 87 | 95.1 | 97.2 | 99.8 |
| *Graff (2021)* | 1.4 | 6.7 | 13 | 24.3 | 56.9 | 84.9 | 93.9 | 97.4 | 99.4 |
| *Huang (2021)* | 1.6 | 7.9 | 13.7 | 26.9 | 58.7 | 85.9 | 92.9 | 96.7 | 99.6 |
| *Hung (2021)* | 1.5 | 7.8 | 15.4 | 27.8 | 60.5 | 87.5 | 93.9 | 97 | 99.4 |
| *Jia (2021)* | 1.3 | 7.7 | 14.3 | 27.8 | 61.4 | 86.8 | 94.5 | 97.2 | 99.4 |
| *Wang (2021)* | 1.9 | 7.5 | 14.1 | 26.6 | 59.3 | 85.2 | 92.6 | 97.1 | 99.7 |
| *Xie (2021)* | 1.5 | 7.9 | 13.6 | 26.9 | 58.4 | 86 | 93 | 96.7 | 99.6 |
| *Barnett (2022)* | 1.8 | 8.1 | 14.7 | 26.7 | 59.5 | 85.8 | 93.3 | 96.9 | 99.7 |
| *J. Choi (2022a)* | 1.2 | 7.7 | 14.2 | 27.9 | 61.3 | 87 | 94.4 | 97.2 | 99.4 |
| *J. Choi (2022b)* | 1.5 | 7.1 | 13.6 | 27.4 | 60.6 | 87.3 | 93.7 | 96.7 | 99.4 |
| *J. Choi (2022c)* | 1.2 | 7.7 | 14.2 | 27.9 | 61.3 | 87 | 94.4 | 97.2 | 99.4 |
| *Liu (2022a)* | 1.4 | 7.4 | 13.8 | 27 | 59.5 | 85.5 | 93.1 | 96.9 | 99.7 |
| *Liu (2022b)* | 0.8 | 6.4 | 12.4 | 26.2 | 57 | 82.7 | 92 | 95.8 | 99.2 |
| *Qin (2022)* | 1.4 | 6.9 | 13.6 | 25.5 | 57.4 | 86 | 93.4 | 96.4 | 99.4 |
| *X. Wang (2022)* | 1.9 | 7.7 | 14.3 | 26.6 | 59 | 85.7 | 93 | 96.9 | 99.6 |
| *P. Zhang (2022)* | 1.8 | 6.3 | 12.7 | 24.6 | 58 | 85.8 | 93 | 96.1 | 99.2 |
| *R. Zhang (2022)* | 1.8 | 7.5 | 14 | 25.2 | 58.5 | 86.3 | 93.6 | 96.7 | 99.5 |
| *Blechter (2023)* | 1.2 | 5.6 | 9.6 | 18.6 | 51.2 | 81.5 | 91.5 | 95.6 | 99.6 |
| *Kim (2023)* | 1.2 | 6.9 | 13.4 | 25.5 | 58.3 | 86 | 92.8 | 96 | 99.3 |
| *Liang (2023)* | 1.8 | 7 | 14.2 | 27.3 | 58.8 | 85.5 | 93.1 | 96.7 | 99.4 |
| *Shi (2023a)* | 1.5 | 6.4 | 11.9 | 22 | 54.4 | 82.1 | 91 | 95.4 | 99.2 |
| *Shi (2023b)* | 1 | 6.4 | 11.7 | 21.9 | 53.3 | 83.4 | 91.8 | 95.8 | 99 |
| *Shi (2023c)* | 0.8 | 5.7 | 11.5 | 21.8 | 52.4 | 83.1 | 91.9 | 95.6 | 98.7 |
| *Wang (2023)* | 1.3 | 7.7 | 14.3 | 27.8 | 61.4 | 86.8 | 94.5 | 97.2 | 99.4 |
| *Wei (2023a)* | 1.4 | 6.5 | 12.7 | 22.9 | 54 | 82.5 | 91 | 95.8 | 99.4 |
| *Wei (2023b)* | 1.2 | 6.9 | 12.6 | 24 | 54.3 | 82.2 | 91.9 | 96.5 | 99.5 |
| *Xiao (2023)* | 1.2 | 6.8 | 12.2 | 22.2 | 55.6 | 83.4 | 92.6 | 96.2 | 99.4 |
| *Xin (2023)* | 1.9 | 7.7 | 14.3 | 27 | 59.5 | 85.1 | 92.8 | 97.2 | 99.8 |
| *Zhang (2023)* | 1.8 | 7.4 | 14.3 | 26.8 | 59.3 | 85.4 | 92.6 | 97.1 | 99.7 |
| *Zhu (2023a)* | 1.9 | 6.7 | 13.1 | 25.1 | 56.2 | 84.2 | 92.8 | 96.6 | 99.5 |
| *Zhu (2023b)* | 1.6 | 7.1 | 14.3 | 27.4 | 59.7 | 87.6 | 94.2 | 97.4 | 99.7 |
| *Zhu (2023c)* | 1.5 | 7.6 | 14.7 | 27.3 | 58.6 | 87.2 | 95.1 | 97.6 | 99.2 |

## S20 Percentiles - Individuals with no history of tobacco use

| PGS n=235,297 | Percentage of cases found in top % of score | | | | | | | | |
|---|---|---|---|---|---|---|---|---|---|
| | 1% | 5% | 10% | 20% | 50% | 80% | 90% | 95% | 99% |
| *Cheng (2016)* | 1.5 | 5.1 | 9.5 | 18.6 | 54.7 | 82.8 | 92.2 | 96.4 | 100 |
| *Qian (2016)* | 0.6 | 5.5 | 11 | 18.9 | 48.5 | 80.3 | 89.6 | 94.3 | 99.4 |
| *Dai (2019)* | 1.9 | 9.3 | 13.3 | 26.9 | 61.2 | 84.5 | 93.2 | 96.8 | 99.6 |
| *Shi (2019)* | 0.4 | 3.6 | 10.6 | 20.8 | 51.3 | 80.7 | 89.6 | 94.9 | 98.7 |
| *Jia (2020)* | 1.9 | 6.8 | 12.1 | 25.6 | 53.8 | 83.1 | 91.5 | 96.8 | 99.6 |
| *Kachuri (2020)* | 1.5 | 5.3 | 10.2 | 22.9 | 52.5 | 83.1 | 91.9 | 96.8 | 99.2 |
| *Yu (2020)* | 1.9 | 6.6 | 13.8 | 24.8 | 55.9 | 84.1 | 91.1 | 96 | 99.2 |
| *Graff (2021)* | 1.5 | 5.3 | 10.2 | 22.9 | 52.5 | 83.1 | 91.9 | 96.8 | 99.2 |
| *Huang (2021)* | 0.8 | 5.7 | 11.9 | 22.9 | 50.2 | 82 | 92.8 | 95.8 | 98.5 |
| *Hung (2021)* | 1.7 | 5.7 | 13.8 | 23.5 | 55.5 | 85.8 | 92.4 | 97.2 | 99.6 |
| *Jia (2021)* | 1.9 | 6.8 | 12.1 | 25.6 | 53.8 | 83.1 | 91.5 | 96.8 | 99.6 |
| *Wang (2021)* | 1.3 | 6.1 | 12.5 | 25.6 | 52.5 | 79.9 | 91.3 | 95.1 | 98.3 |
| *Xie (2021)* | 0.8 | 5.7 | 12.1 | 22.7 | 50.6 | 81.8 | 92.8 | 95.8 | 98.5 |
| *Barnett (2022)* | 1.7 | 7 | 12.7 | 24.8 | 54.2 | 82.8 | 90.7 | 95.8 | 98.9 |
| *J. Choi (2022a)* | 1.9 | 6.6 | 12.5 | 25.4 | 53.4 | 82.8 | 91.5 | 96.8 | 99.6 |
| *J. Choi (2022b)* | 1.7 | 6.6 | 14.4 | 25.8 | 54 | 83.5 | 92.6 | 96.2 | 99.6 |
| *J. Choi (2022c)* | 1.9 | 6.6 | 12.5 | 25.4 | 53.4 | 82.8 | 91.5 | 96.8 | 99.6 |
| *Liu (2022a)* | 1.7 | 6.4 | 14.2 | 25.2 | 56.4 | 84.1 | 92.2 | 95.3 | 99.6 |
| *Liu (2022b)* | 1.9 | 7 | 11.4 | 22.5 | 51.7 | 81.6 | 91.7 | 96 | 98.9 |
| *Qin (2022)* | 1.9 | 8.3 | 14 | 25.6 | 60.8 | 85.4 | 92.6 | 96.2 | 98.9 |
| *X. Wang (2022)* | 1.3 | 6.4 | 13.6 | 21.8 | 50.6 | 81.6 | 93.9 | 96.4 | 98.5 |
| *P. Zhang (2022)* | 2.1 | 6.8 | 11.4 | 22 | 55.5 | 86.2 | 93 | 96.8 | 99.2 |
| *R. Zhang (2022)* | 1.3 | 7 | 11.7 | 24.6 | 55.3 | 85 | 92.4 | 95.6 | 99.4 |
| *Blechter (2023)* | 0.6 | 5.1 | 10.2 | 20.1 | 48.5 | 80.7 | 91.5 | 94.9 | 99.2 |
| *Kim (2023)* | 1.5 | 5.3 | 11.7 | 23.3 | 53 | 82.2 | 93 | 97.5 | 99.6 |
| *Liang (2023)* | 1.7 | 7 | 12.3 | 24.4 | 52.8 | 83.3 | 90.9 | 96.2 | 99.4 |
| *Shi (2023a)* | 2.3 | 7.4 | 14.4 | 25.8 | 57.4 | 83.5 | 92.4 | 96.6 | 99.8 |
| *Shi (2023b)* | 1.5 | 7.8 | 13.3 | 26.1 | 56.6 | 81.8 | 93.2 | 97.2 | 100 |
| *Shi (2023c)* | 1.5 | 8.1 | 13.1 | 24.6 | 54.9 | 82.6 | 92.8 | 97 | 99.8 |
| *Wang (2023)* | 1.9 | 6.8 | 12.1 | 25.6 | 53.8 | 83.1 | 91.5 | 96.8 | 99.6 |
| *Wei (2023a)* | 1.9 | 7 | 13.6 | 24.6 | 55.5 | 84.1 | 93 | 96.8 | 99.2 |
| *Wei (2023b)* | 2.3 | 7.6 | 12.7 | 25.2 | 55.7 | 85.4 | 93.4 | 96.6 | 99.2 |
| *Xiao (2023)* | 0.8 | 4.4 | 12.1 | 22 | 53.6 | 82.4 | 91.3 | 95.3 | 98.7 |
| *Xin (2023)* | 1.3 | 6.4 | 12.5 | 24.8 | 52.3 | 80.7 | 91.5 | 95.3 | 98.5 |
| *Zhang (2023)* | 1.3 | 6.1 | 12.5 | 25.4 | 53 | 80.3 | 91.5 | 95.1 | 98.3 |
| *Zhu (2023a)* | 1.5 | 8.3 | 15 | 26.1 | 58.3 | 84.7 | 92.2 | 96 | 99.4 |
| *Zhu (2023b)* | 2.1 | 6.8 | 12.7 | 23.3 | 55.7 | 85.6 | 93.6 | 96.8 | 99.2 |
| *Zhu (2023c)* | 1.5 | 6.8 | 12.9 | 21.6 | 56.6 | 88.1 | 93.6 | 96.6 | 98.9 |

## S21 PRISMA Checklist

| Section and Topic | Item # | Checklist item | Location where item is reported |
|---|---|---|---|
| **TITLE** | | | |
| Title | 1 | Identify the report as a systematic review. | Line 1 |
| **ABSTRACT** | | | |
| Abstract | 2 | See the PRISMA 2020 for Abstracts checklist. | Page 2 |
| **INTRODUCTION** | | | |
| Rationale | 3 | Describe the rationale for the review in the context of existing knowledge. | Lines 66-82 |
| Objectives | 4 | Provide an explicit statement of the objective(s) or question(s) the review addresses. | Lines 78-82 |
| **METHODS** | | | |
| Eligibility criteria | 5 | Specify the inclusion and exclusion criteria for the review and how studies were grouped for the syntheses. | Lines 101-112 |
| Information sources | 6 | Specify all databases, registers, websites, organisations, reference lists and other sources searched or consulted to identify studies. Specify the date when each source was last searched or consulted. | Lines 89-90 |
| Search strategy | 7 | Present the full search strategies for all databases, registers and websites, including any filters and limits used. | S1 and S2 |
| Selection process | 8 | Specify the methods used to decide whether a study met the inclusion criteria of the review, including how many reviewers screened each record and each report retrieved, whether they worked independently, and if applicable, details of automation tools used in the process. | Lines 114-121 |
| Data collection process | 9 | Specify the methods used to collect data from reports, including how many reviewers collected data from each report, whether they worked independently, any processes for obtaining or confirming data from study investigators, and if applicable, details of automation tools used in the process. | Lines 123-133 |
| Data items | 10a | List and define all outcomes for which data were sought. Specify whether all results that were compatible with each outcome domain in each study were sought (e.g. for all measures, time points, analyses), and if not, the methods used to decide which results to collect. | Lines 128-135 |
| | 10b | List and define all other variables for which data were sought (e.g. participant and intervention characteristics, funding sources). Describe any assumptions made about any missing or unclear information. | Lines 133-135 |
| Study risk of bias assessment | 11 | Specify the methods used to assess risk of bias in the included studies, including details of the tool(s) used, how many reviewers assessed each study and whether they worked independently, and if applicable, details of automation tools used in the process . | N/A |
| Effect measures | 12 | Specify for each outcome the effect measure(s) (e.g. risk ratio, mean difference) used in the synthesis or presentation of results. | Lines 123-137 |
| Synthesis methods | 13a | Describe the processes used to decide which studies were eligible for each synthesis (e.g. tabulating the study intervention characteristics and comparing against the planned groups for each synthesis (item #5)). | Lines 210-216 |
| | 13b | Describe any methods required to prepare the data for presentation or synthesis, such as handling of missing summary statistics, or data conversions. | Lines 230-239 |
| | 13c | Describe any methods used to tabulate or visually display results of individual studies and syntheses. | Lines 123-133 and 154-161 |
| | 13d | Describe any methods used to synthesize results and provide a rationale for the choice(s). If meta-analysis was performed, describe the model(s), method(s) to identify the presence and extent of statistical heterogeneity, and software package(s) used. | Lines 135-137 |

**S21 PRISMA Checklist**

| Section and Topic | Item # | Checklist item | Location where item is reported |
|---|---|---|---|
| | 13e | Describe any methods used to explore possible causes of heterogeneity among study results (e.g. subgroup analysis, meta-regression). | Lines 167-168 |
| | 13f | Describe any sensitivity analyses conducted to assess robustness of the synthesized results. | N/A |
| Reporting bias assessment | 14 | Describe any methods used to assess risk of bias due to missing results in a synthesis (arising from reporting biases). | N/A |
| Certainty assessment | 15 | Describe any methods used to assess certainty (or confidence) in the body of evidence for an outcome. | N/A |
| **RESULTS** | | | |
| Study selection | 16a | Describe the results of the search and selection process, from the number of records identified in the search to the number of studies included in the review, ideally using a flow diagram. | Figure S1 |
| | 16b | Cite studies that might appear to meet the inclusion criteria, but which were excluded, and explain why they were excluded . | Lines 108-112, Lines 210-216 |
| Study characteristics | 17 | Cite each included study and present its characteristics . | Table 1 |
| Risk of bias in studies | 18 | Present assessments of risk of bias for each included study. | N/A |
| Results of individual studies | 19 | For all outcomes, present, for each study: (a) summary statistics for each group (where appropriate) and (b) an effect estimate and its precision (e.g. confidence/credible interval), ideally using structured tables or plots. | N/A |
| Results of syntheses | 20a | For each synthesis, briefly summarise the characteristics and risk of bias among contributing studies . | Lines 174-208 |
| | 20b | Present results of all statistical syntheses conducted. If meta-analysis was done, present for each the summary estimate and its precision (e.g. confidence/credible interval) and measures of statistical heterogeneity. If comparing groups, describe the direction of the effect. | N/A |
| | 20c | Present results of all investigations of possible causes of heterogeneity among study results. | N/A |
| | 20d | Present results of all sensitivity analyses conducted to assess the robustness of the synthesized results. | N/A |
| Reporting biases | 21 | Present assessments of risk of bias due to missing results (arising from reporting biases) for each synthesis assessed. | N/A |
| Certainty of evidence | 22 | Present assessments of certainty (or confidence) in the body of evidence for each outcome assessed. | N/A |
| **DISCUSSION** | | | |
| Discussion | 23a | Provide a general interpretation of the results in the context of other evidence. | Lines 276-284 |
| | 23b | Discuss any limitations of the evidence included in the review. | Lines 301-306 |
| | 23c | Discuss any limitations of the review processes used . | Lines 333-336 |
| | 23d | Discuss implications of the results for practice, policy, and future research. | Lines 340-352 |
| **OTHER INFORMATIO N** | | | |
| Registration and protocol | 24a | Provide registration information for the review, including register name and registration number, or state that the review was not registered. | N/A |
| | 24b | Indicate where the review protocol can be accessed, or state that a protocol was not prepared . | N/A |
| | 24c | Describe and explain any amendments to information provided at registration or in the protocol. | N/A |

## S21 PRISMA Checklist

| Section and Topic | Item # | Checklist item | Location where item is reported |
|---|---|---|---|
| Support | 25 | Describe sources of financial or non-financial support for the review, and the role of the funders or sponsors in the review. | Page 29 |
| Competing interests | 26 | Declare any competing interests of review authors. | Page 29 |
| Availability of data, code and other materials | 27 | Report which of the following are publicly available and where they can be found: template data collection forms; data extracted from included studies; data used for all analyses; analytic code; any other materials used in the review . | Page 28 |
