# Supplementary material for: The current state of polygenic scores for the development of lung cancer: a systematic review and validation in UK Biobank
Source: Br J Cancer. 2026 Jan 8;134(6):939–48. doi: 10.1038/s41416-025-03330-9 (PMC12960659; doi:10.1038/s41416-025-03330-9)

Figure S1: PRISMA Diagram for systematic review


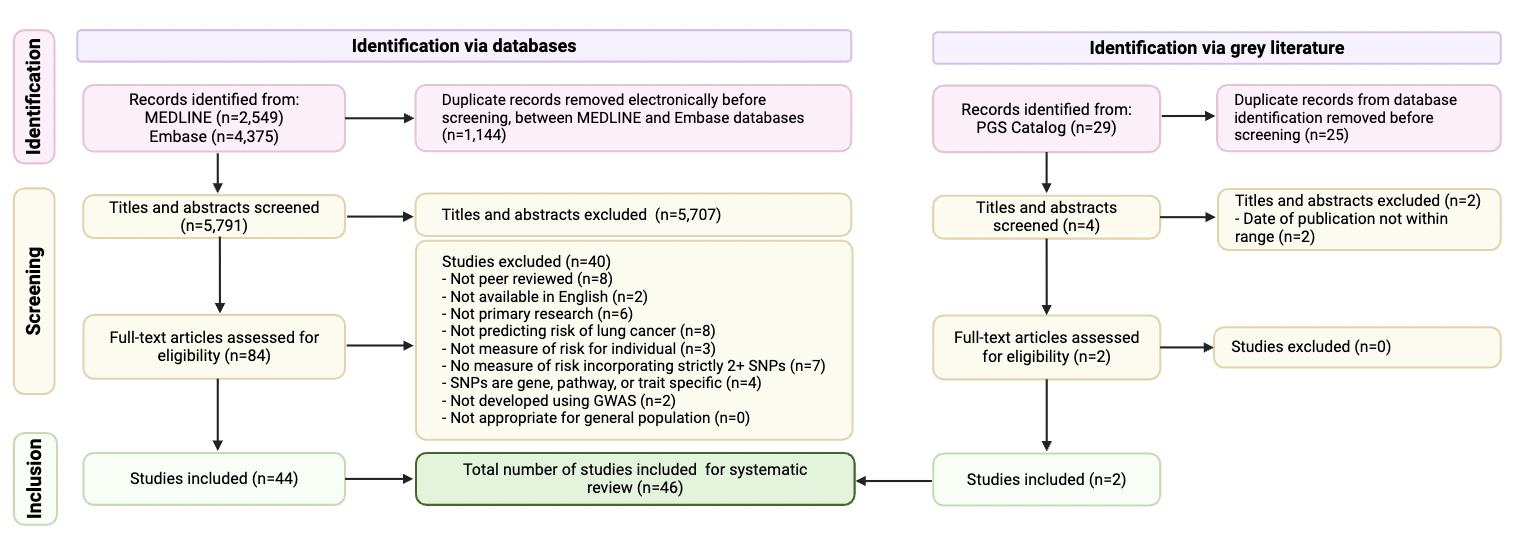


Figure S2: Derivation of validation cohort


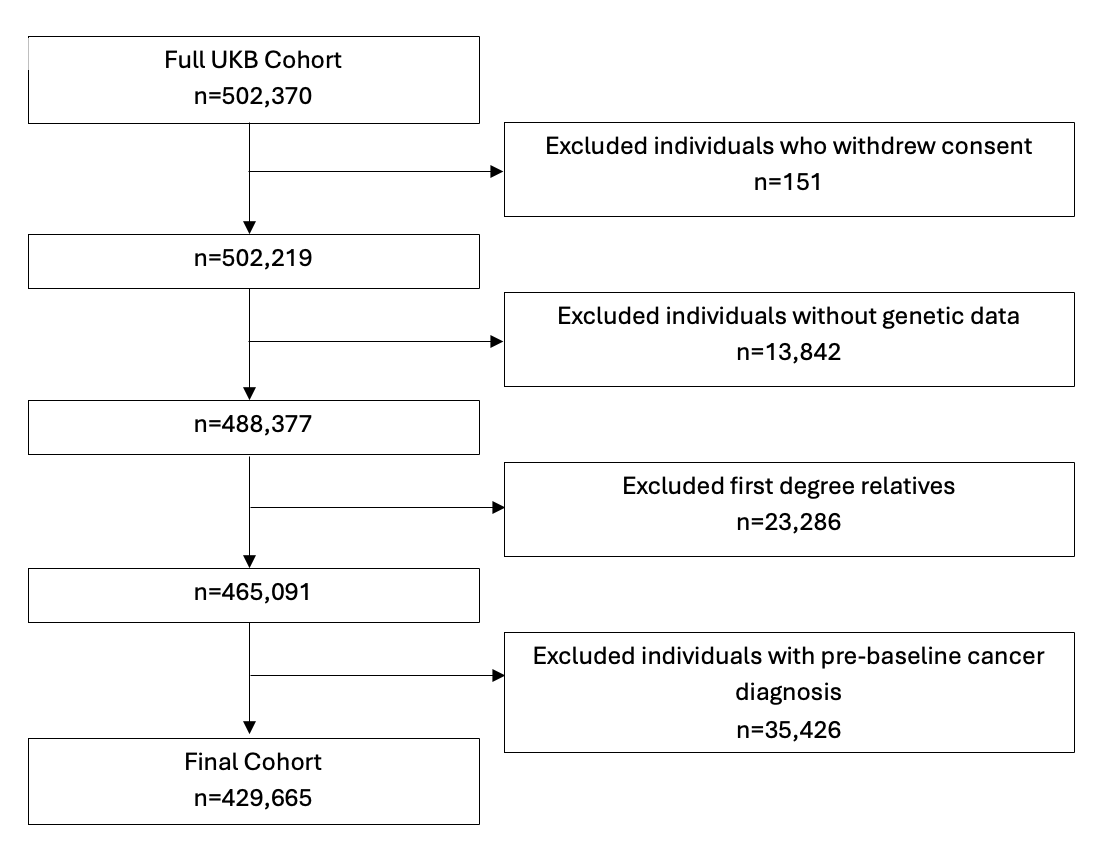


Figure S3: Distributions in the UKB cohort (by case and control) of the 38 PGS included in the validation

| **PGS Ref** | **Distribution of PGS for cases and controls in UKB cohort** |
| --- | --- |
| Barnett (2022) | 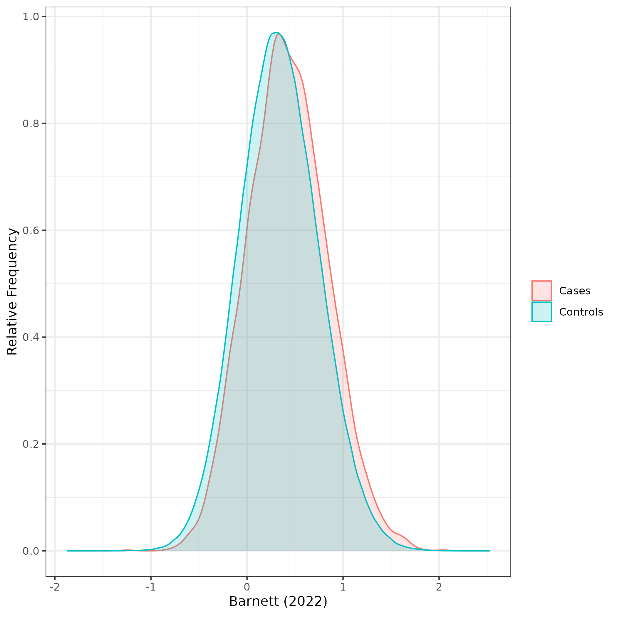 |
| Bletcher (2023) | 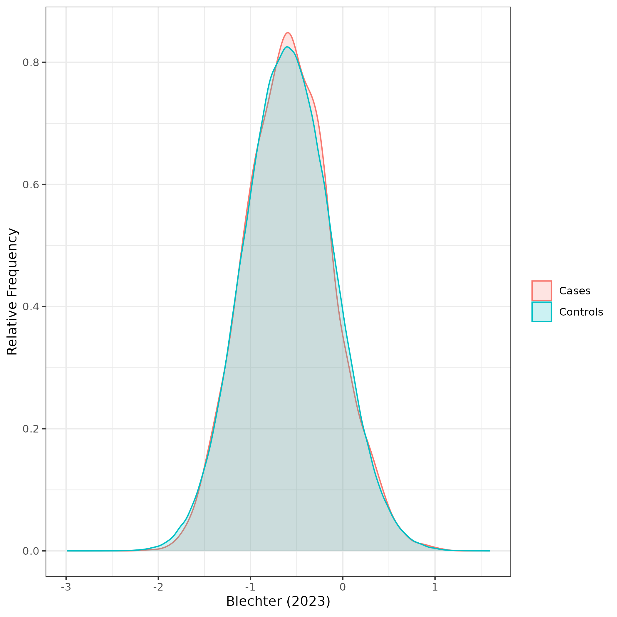 |
| Dai (2019) | 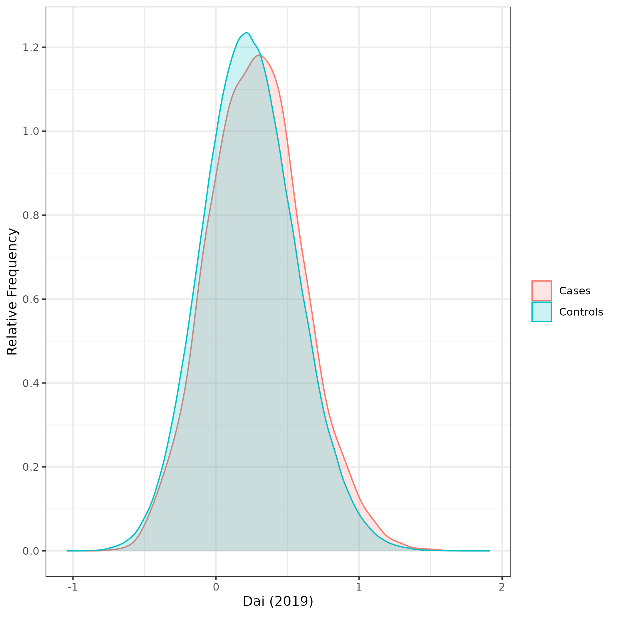 |
| Graff (2021) | 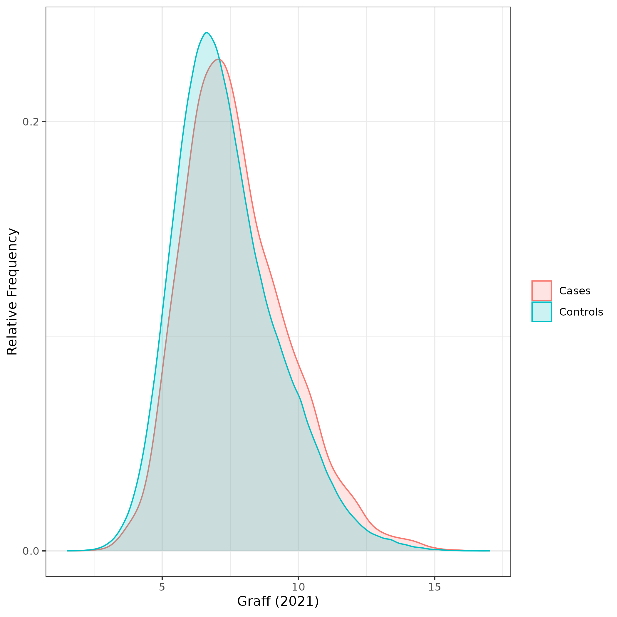 |
| Huang (2021) | 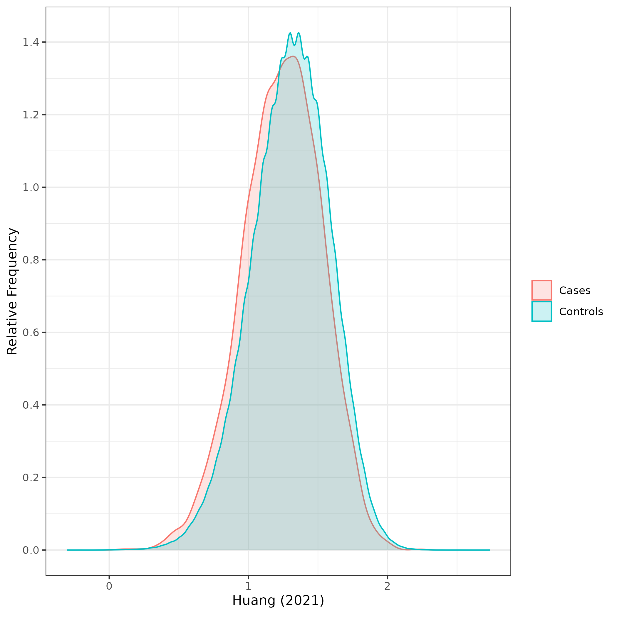 |
| Hung (2021) | 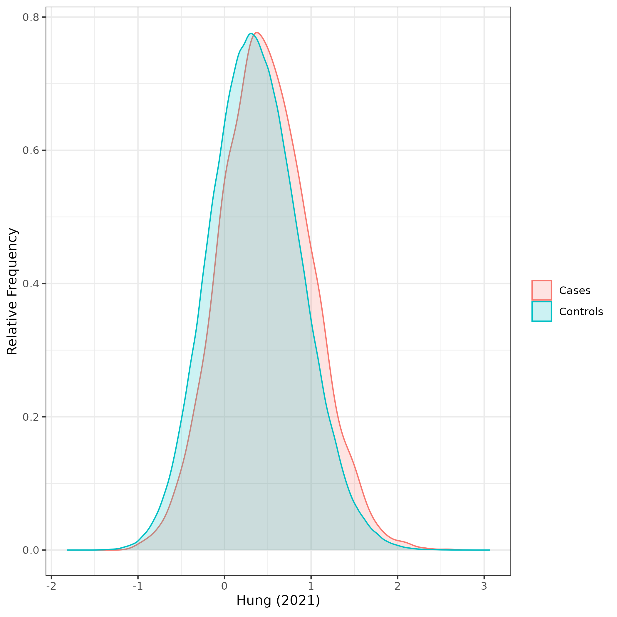 |
| J. Choi (2022c) | 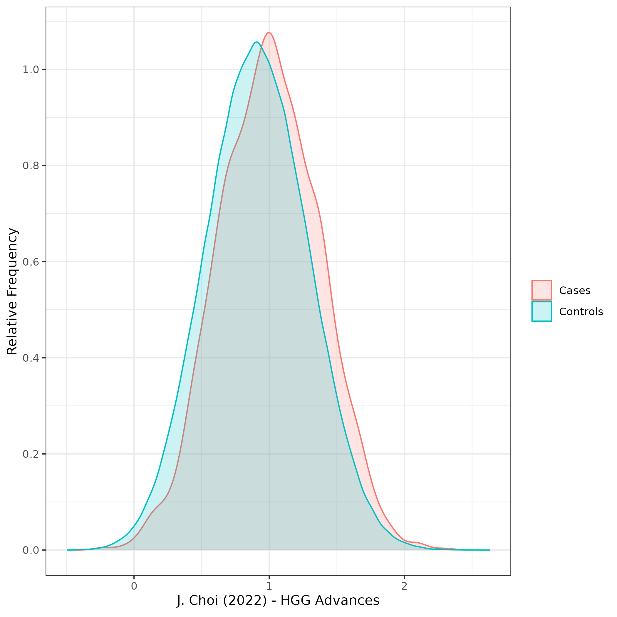 |
| J. Choi (2022a) | 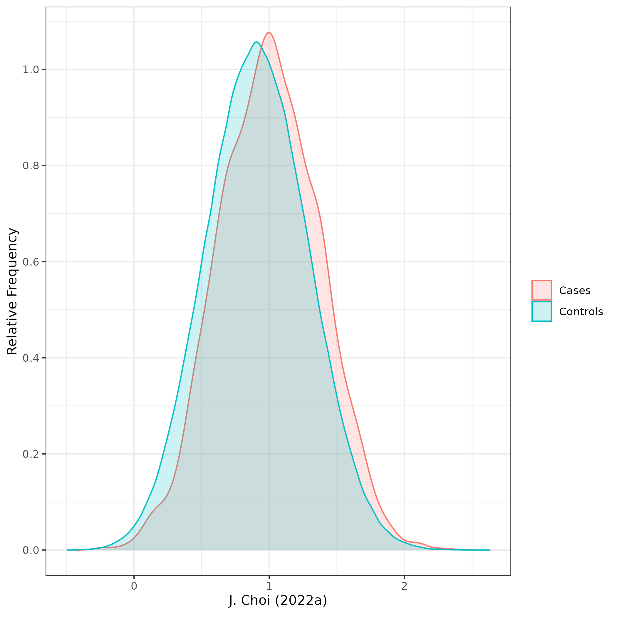 |
| J. Choi (2022b) | 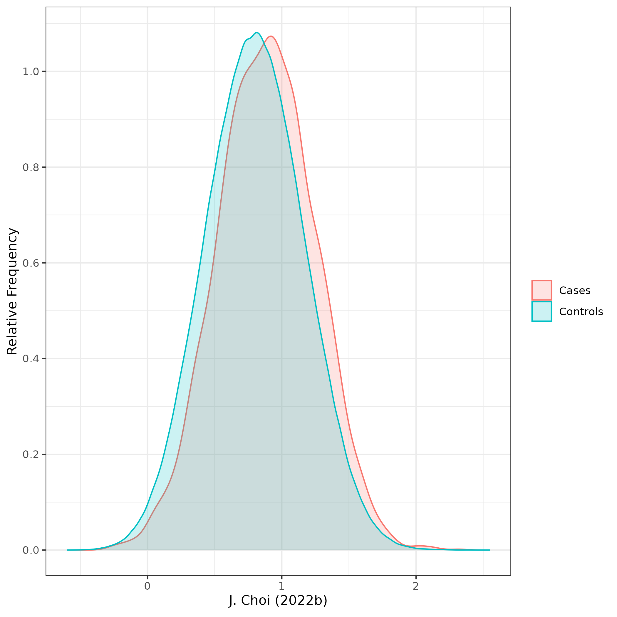 |
| Jia (2020) | 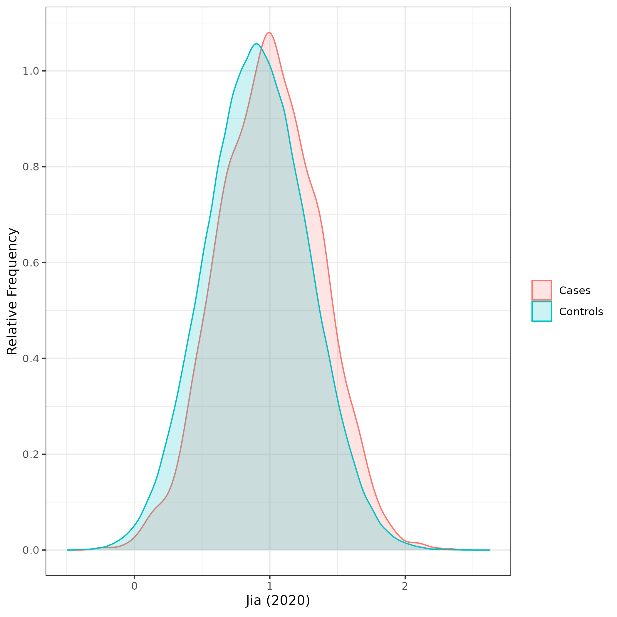 |
| Jia (2021) | 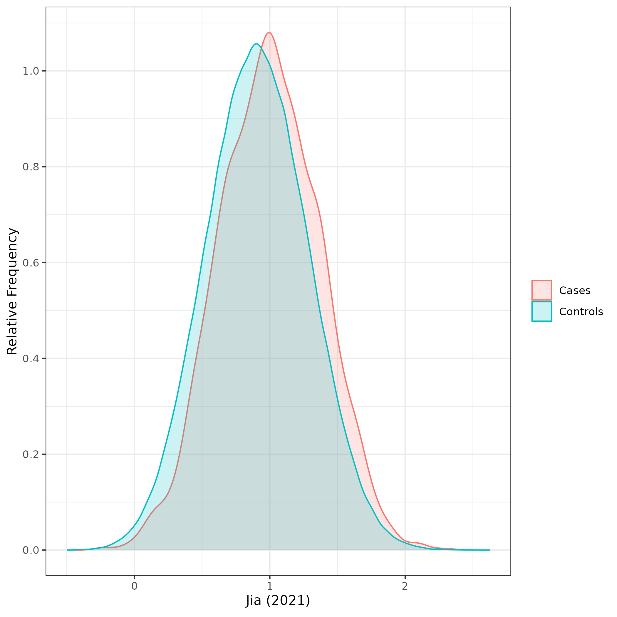 |
| Kachuri (2020) | 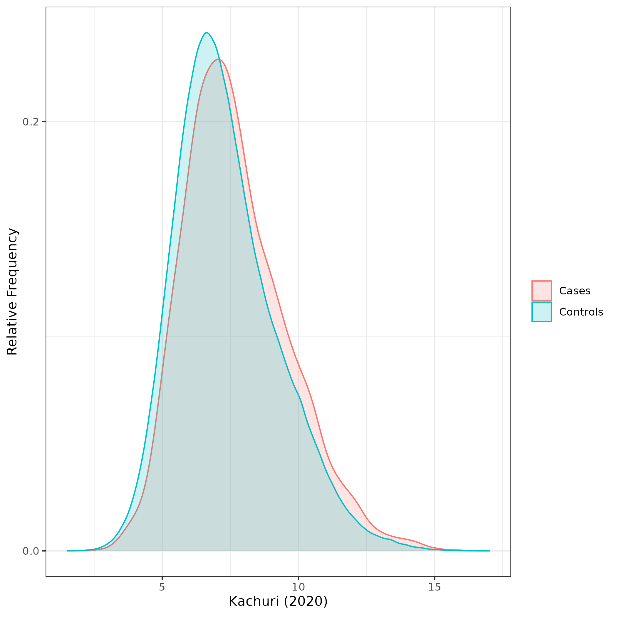 |
| Kim (2023) | 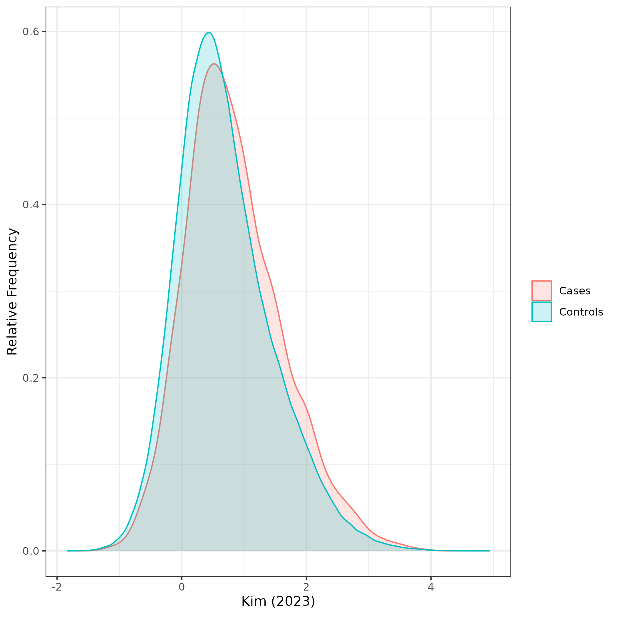 |
| L. Wang (2022) | 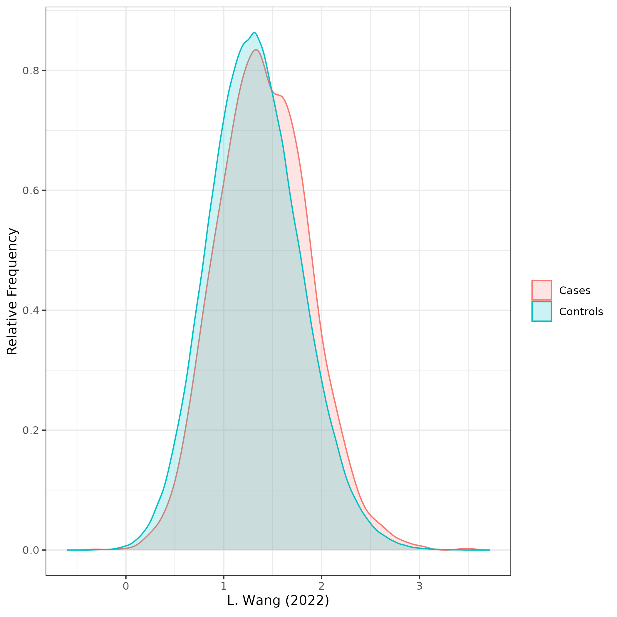 |
| Liang (2023) | 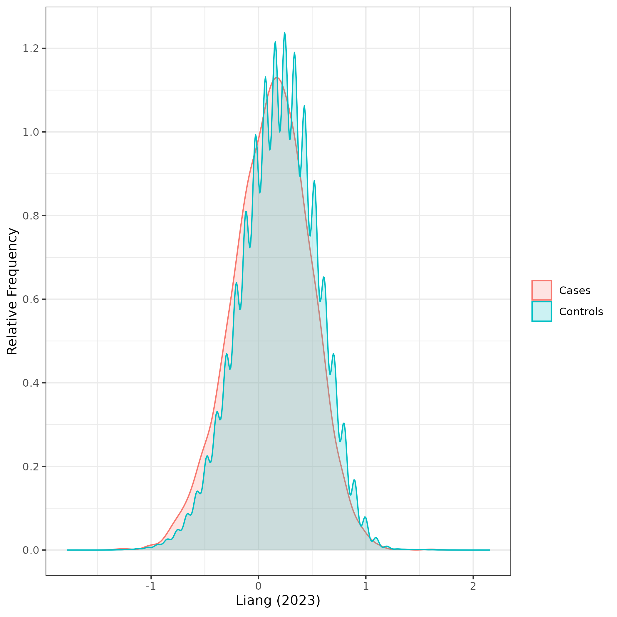 |
| Liu (2022a) | 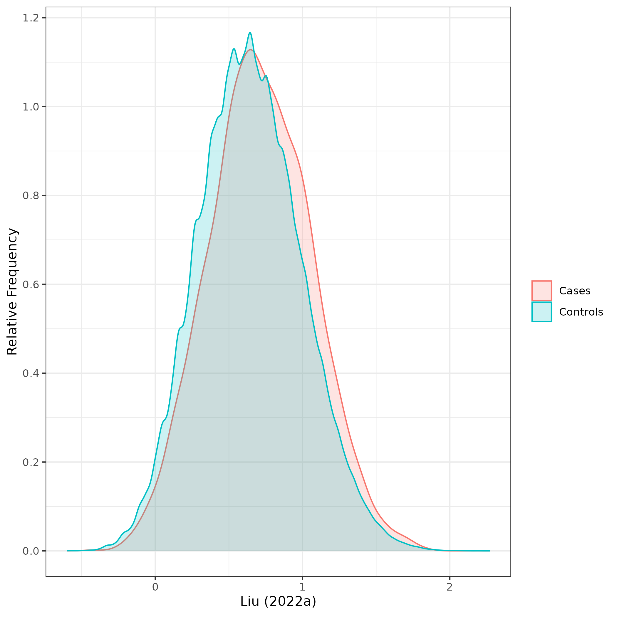 |
| Liu (2022b) | 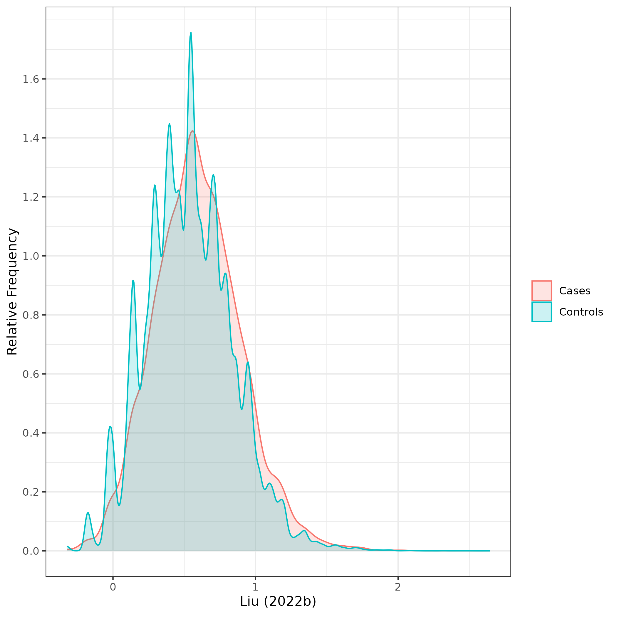 |
| P. Zhang (2022) | 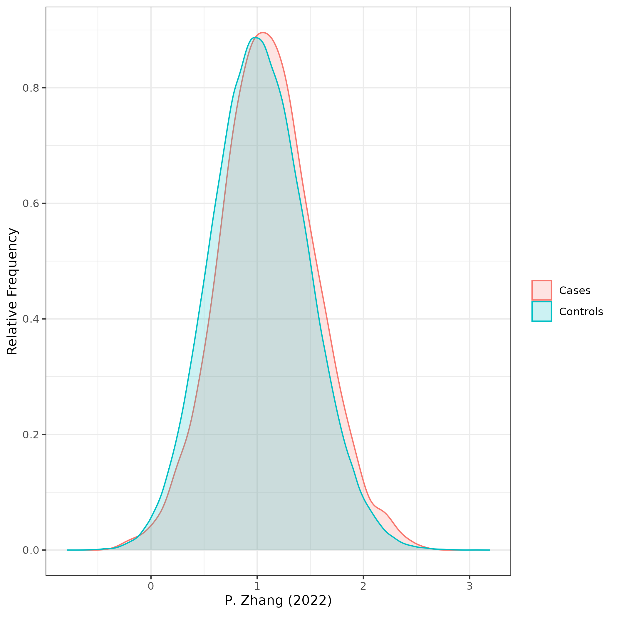 |
| Qian (2016) | 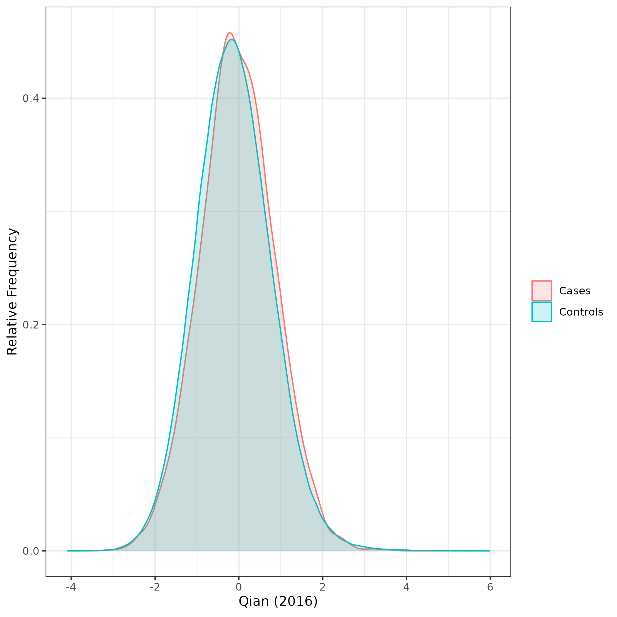 |
| Qin (2022) | 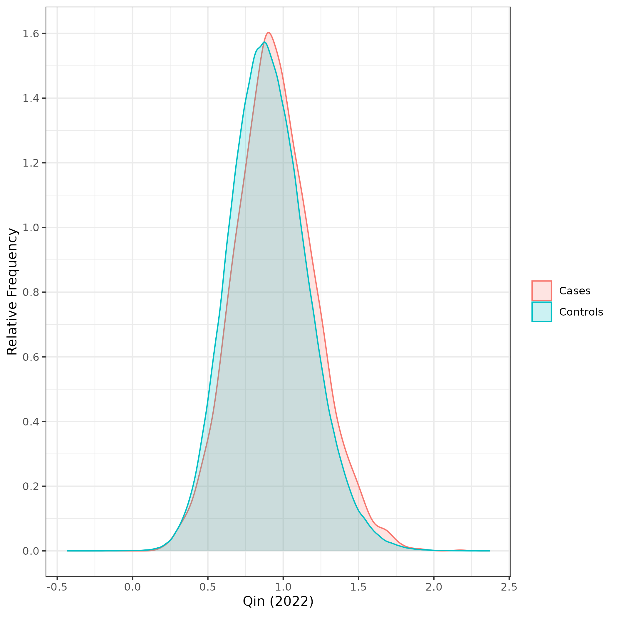 |
| R. Zhang (2022) | 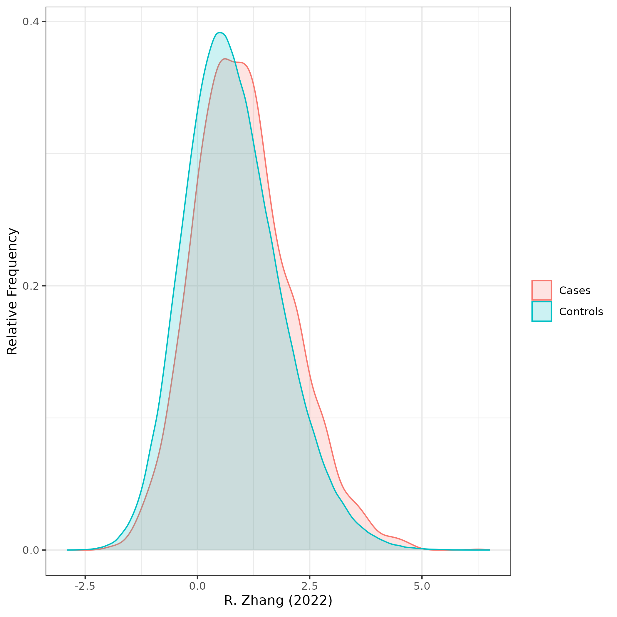 |
| Shi (2019) | 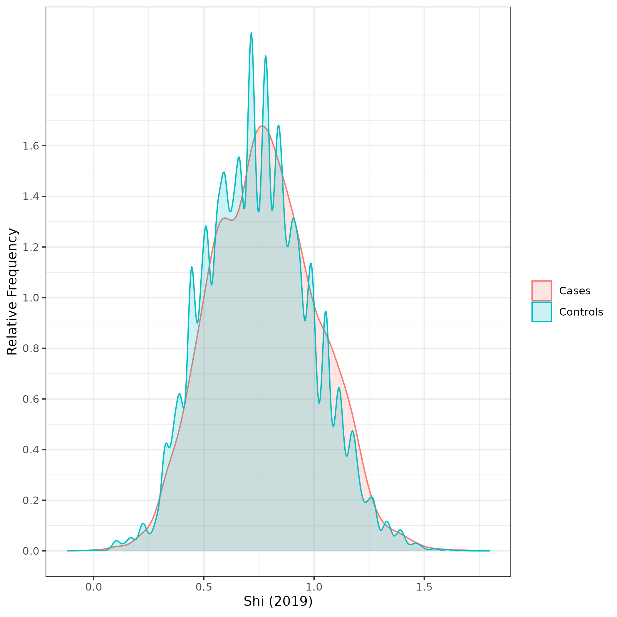 |
| Shi (2023a) | 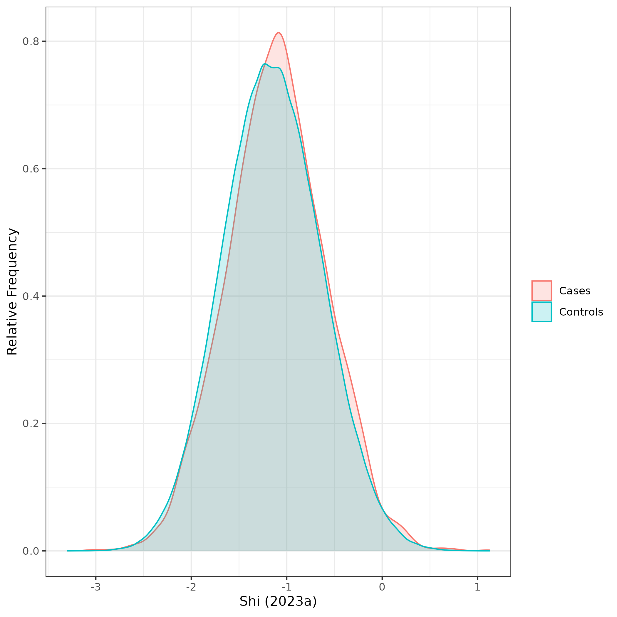 |
| Shi (2023b) | 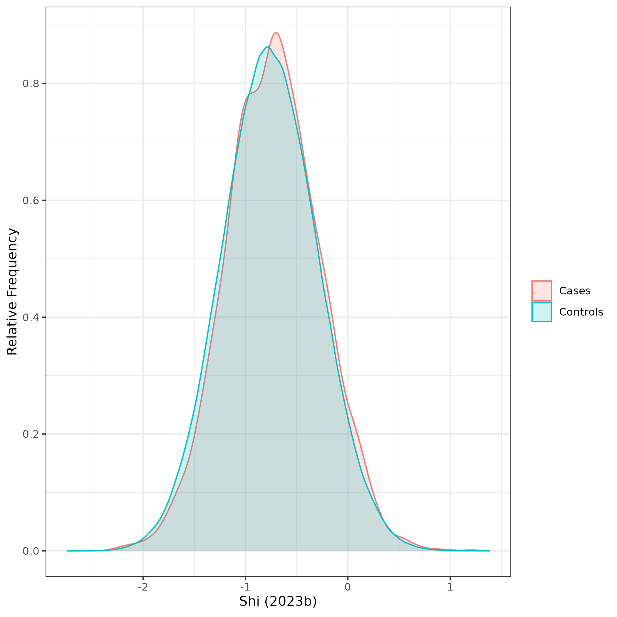 |
| Shi (2023c) | 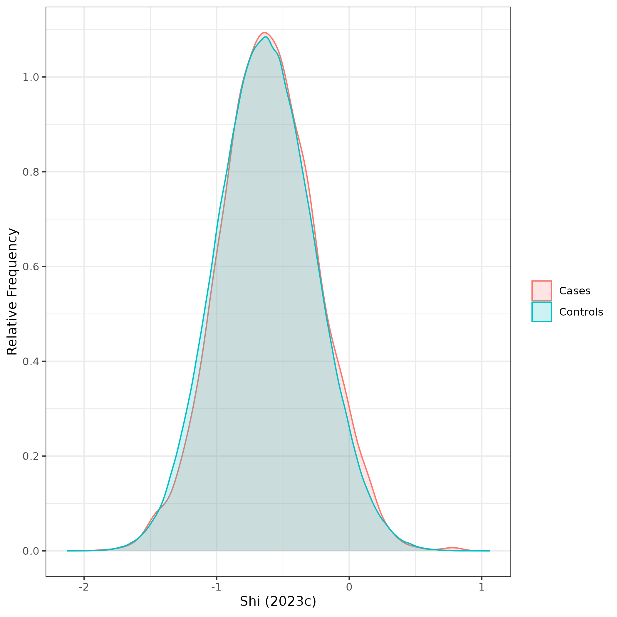 |
| Wang (2021) | 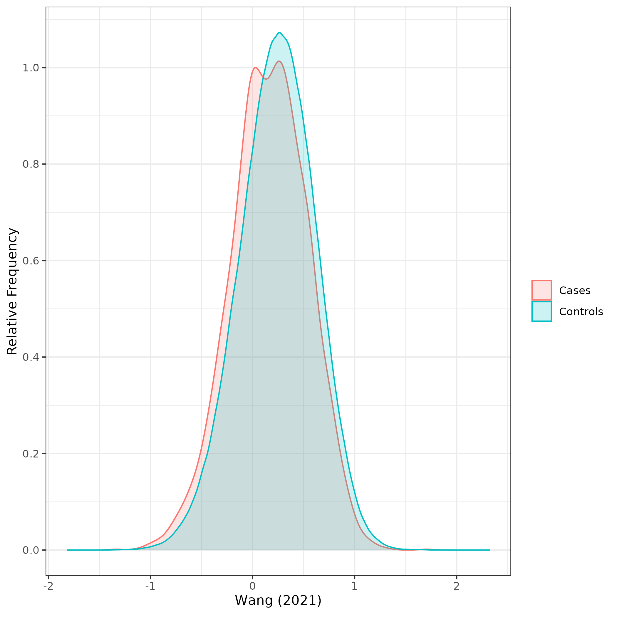 |
| Wang (2023) | 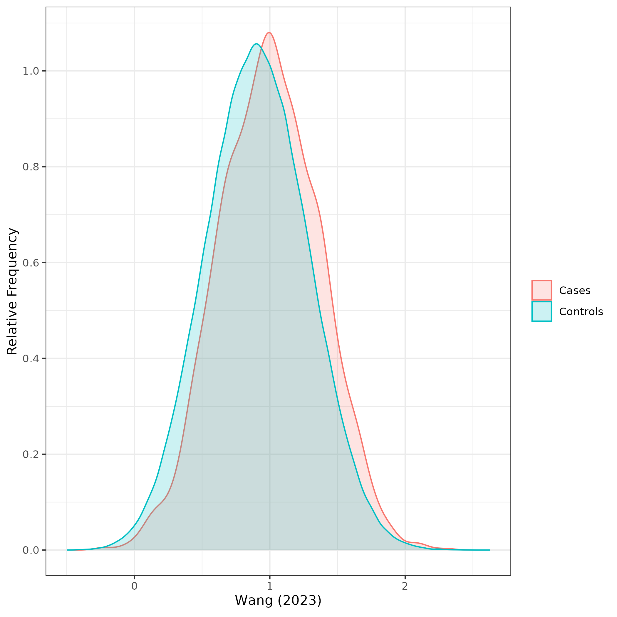 |
| Wei (2023a) | 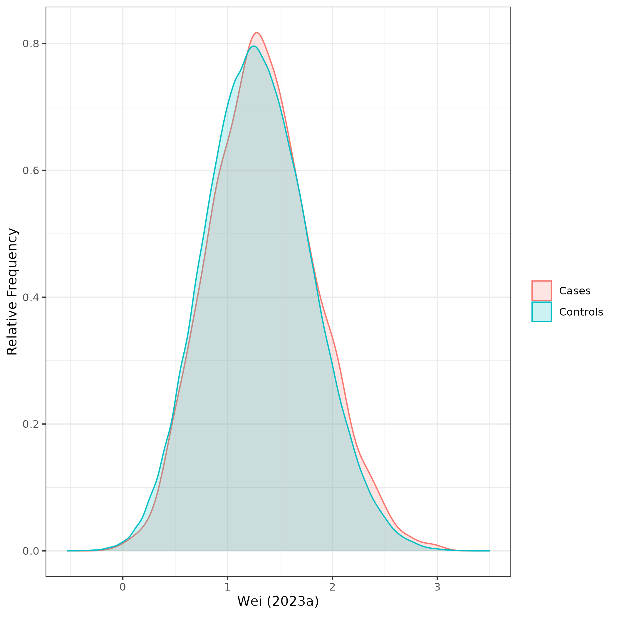 |
| Wei (2023b) | 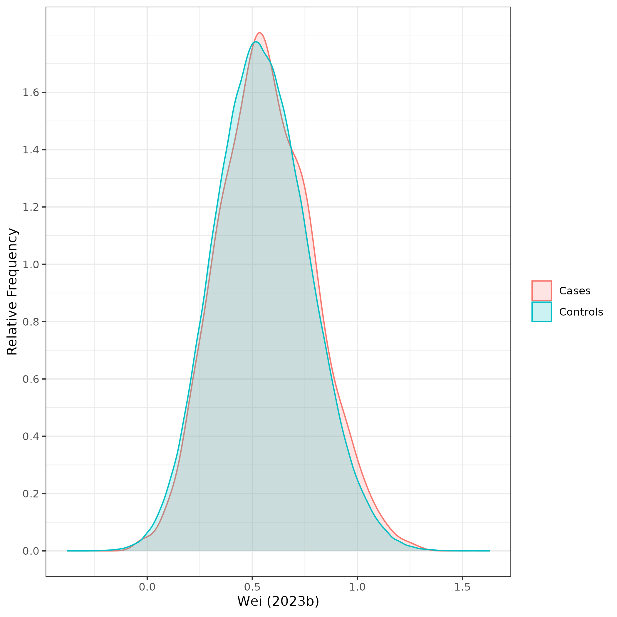 |
| X. Wang (2022) | 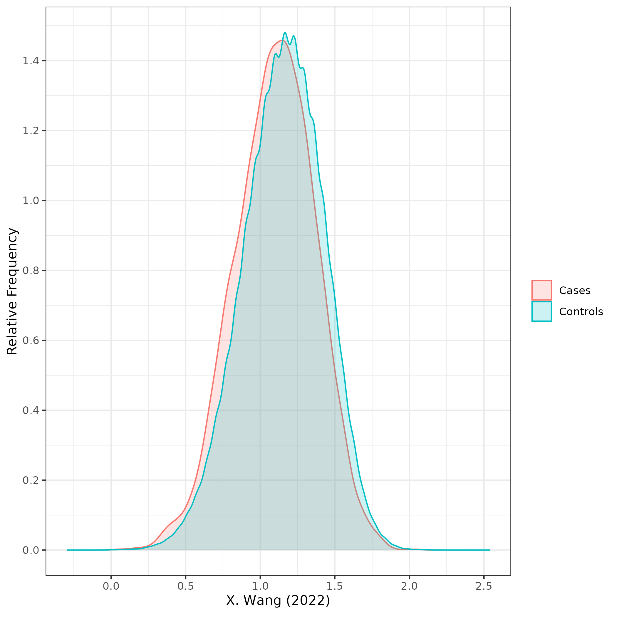 |
| Xiao (2023) | 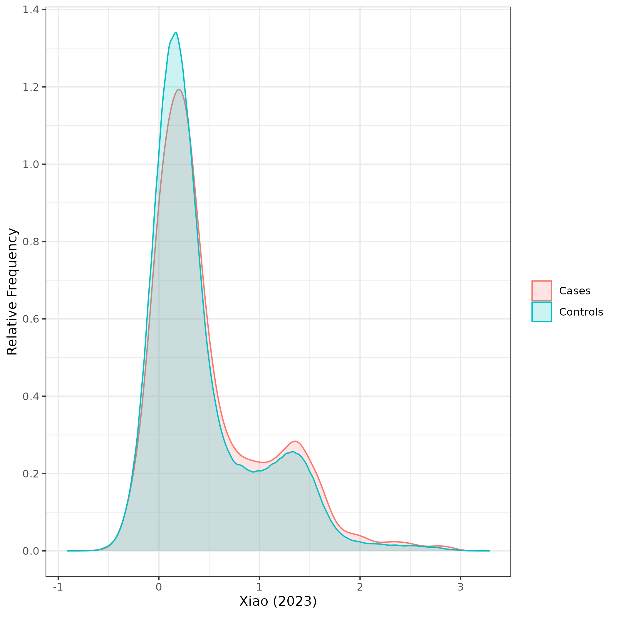 |
| Xie (2021) | 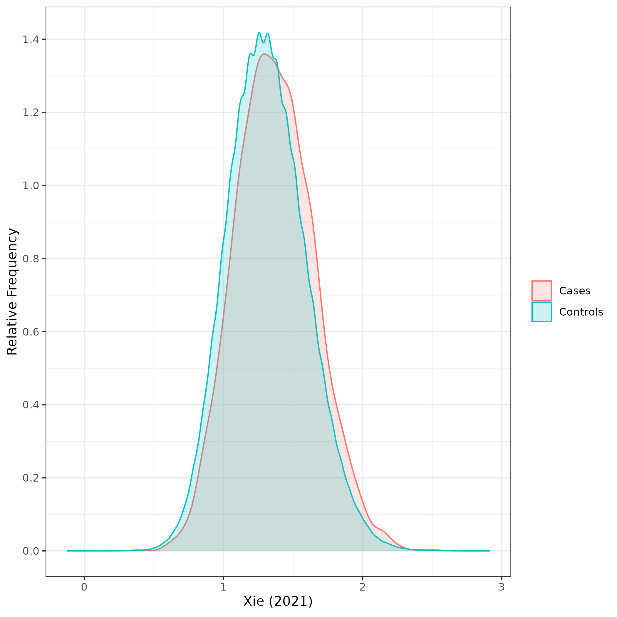 |
| Xin (2023) | 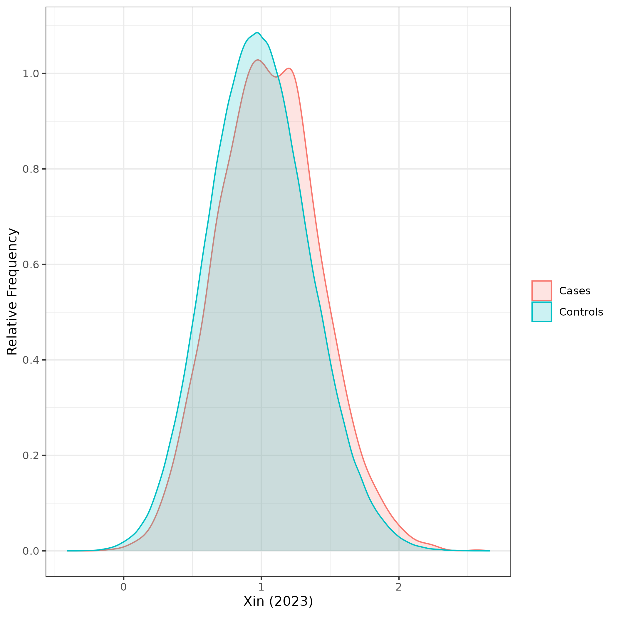 |
| Yang (2016) | 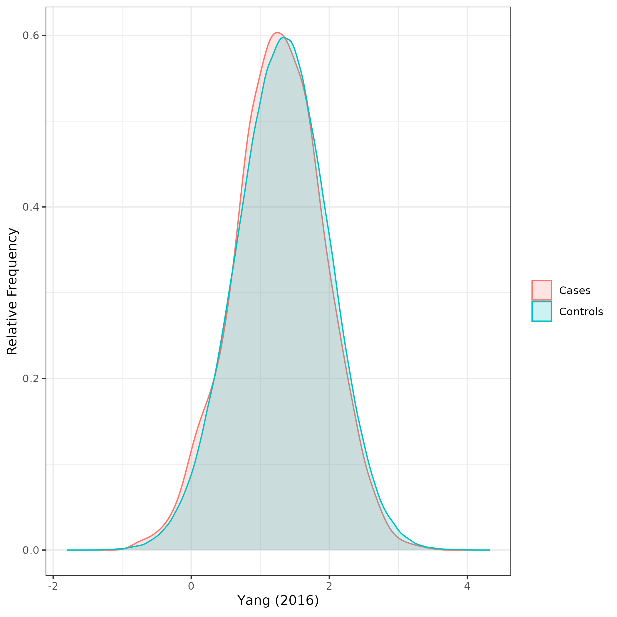 |
| Yu (2020) | 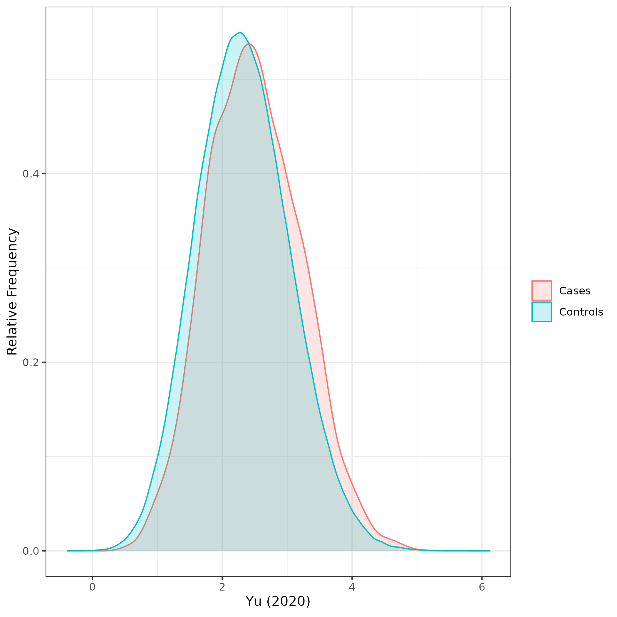 |
| Zhang (2023) | 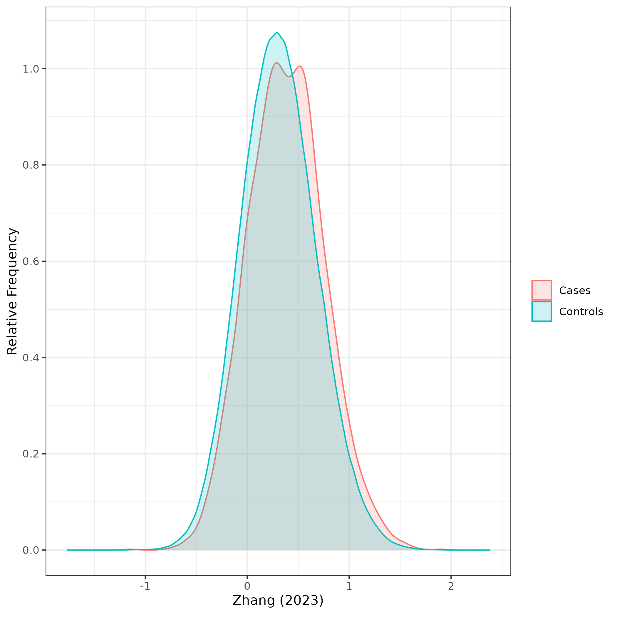 |
| Zhu (2023a) | 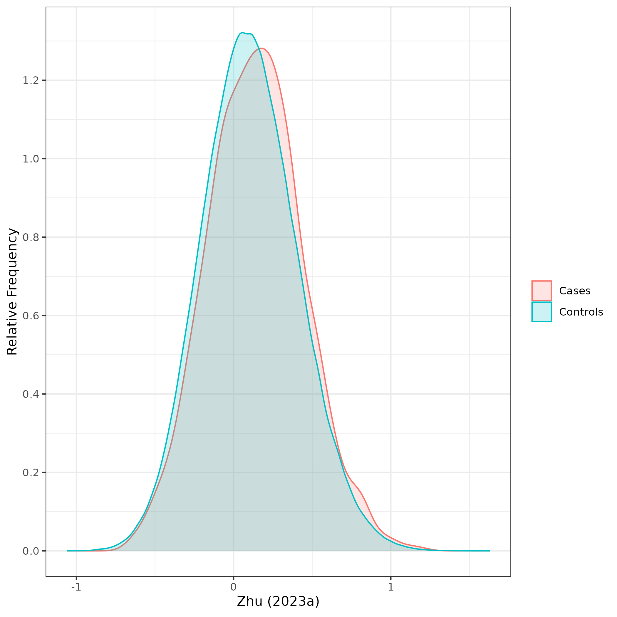 |
| Zhu (2023b) | 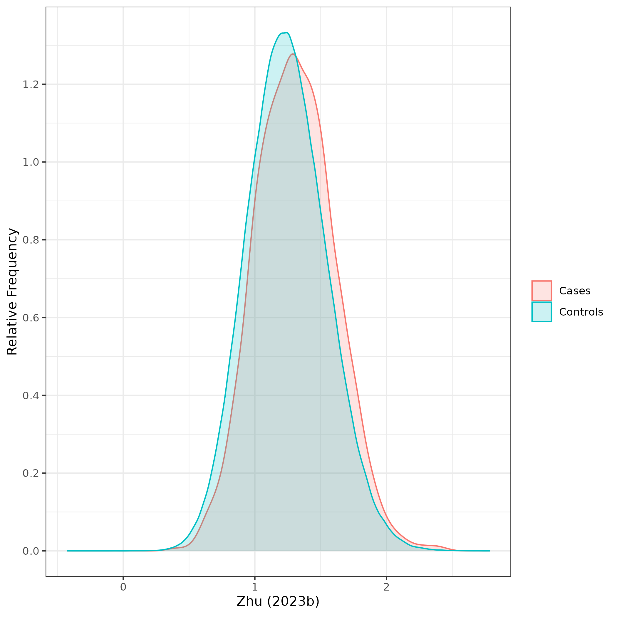 |
| Zhu (2023c) | 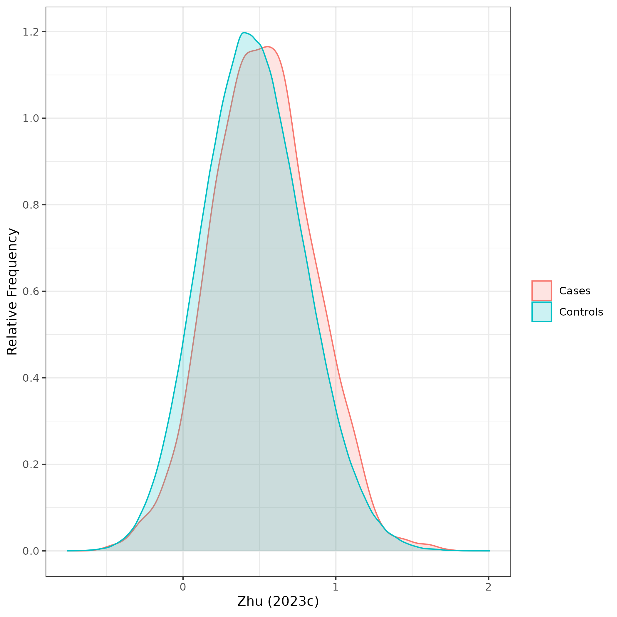 |

Figure S4: C-indices of the PGS in UK Biobank compared by number of SNPs included in the score.
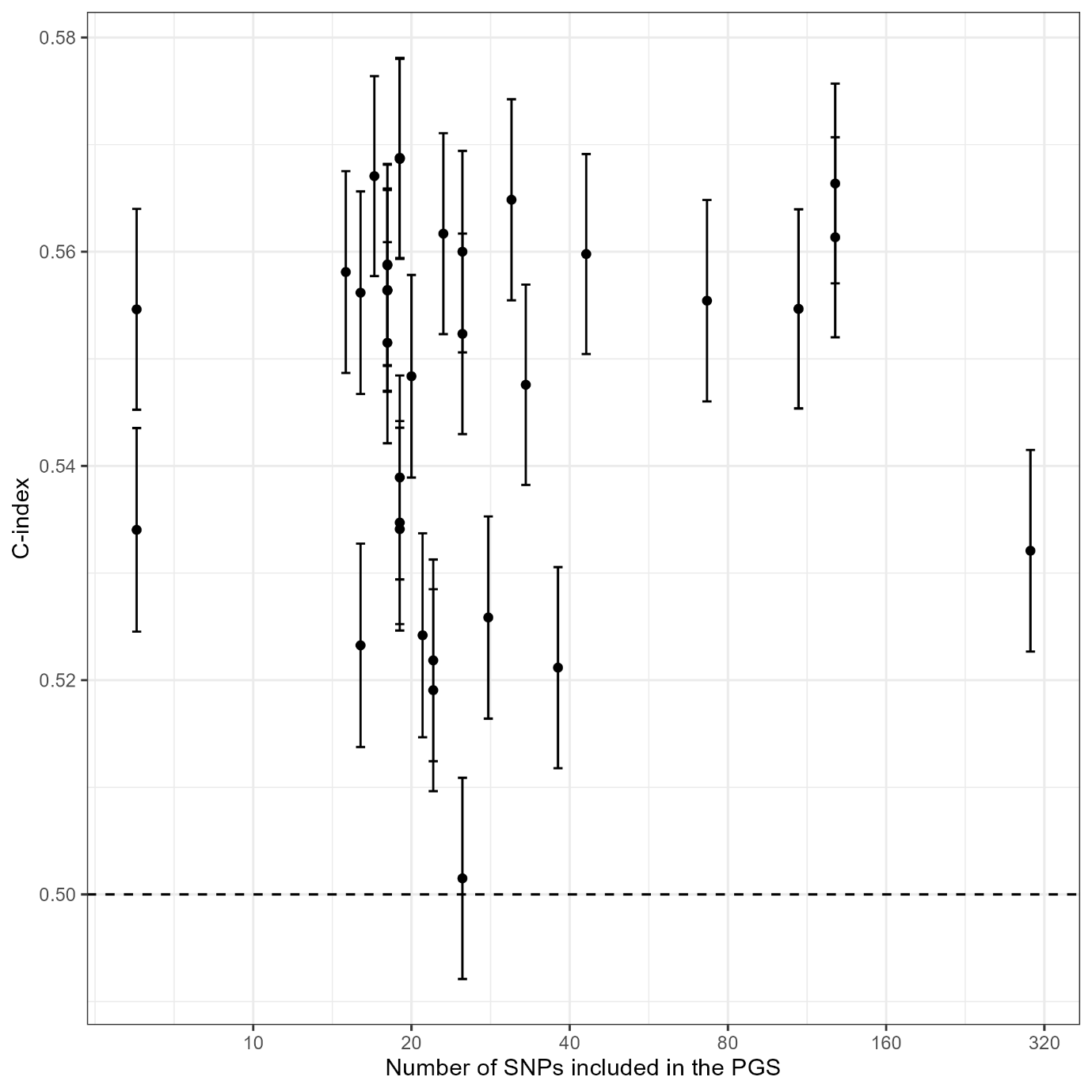


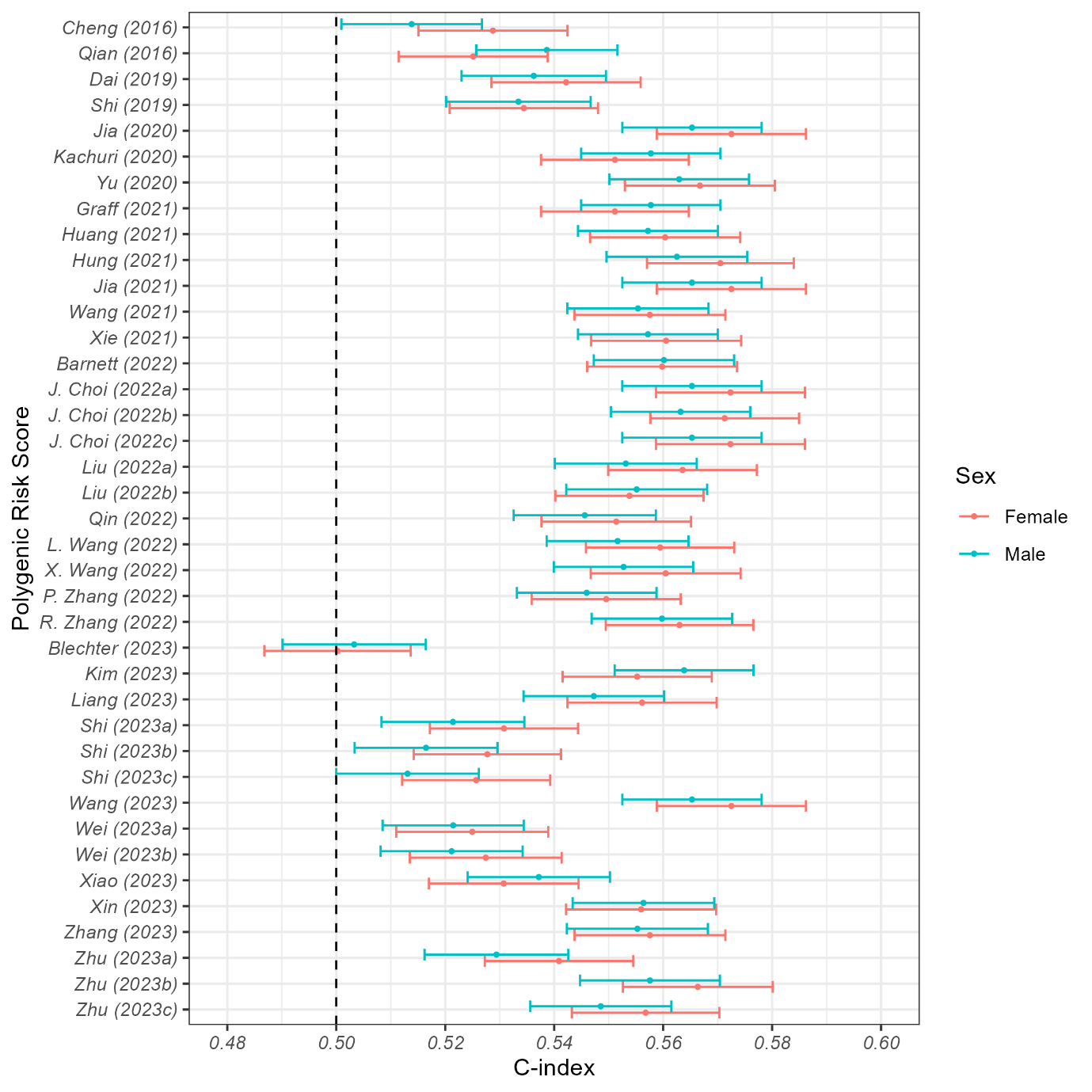
Figure S5: C-indices of the polygenic scores by sex.

Figure S6: C-indices of the polygenic scores by Ethnicity (comparing the whole cohort to a subset of only white individuals).


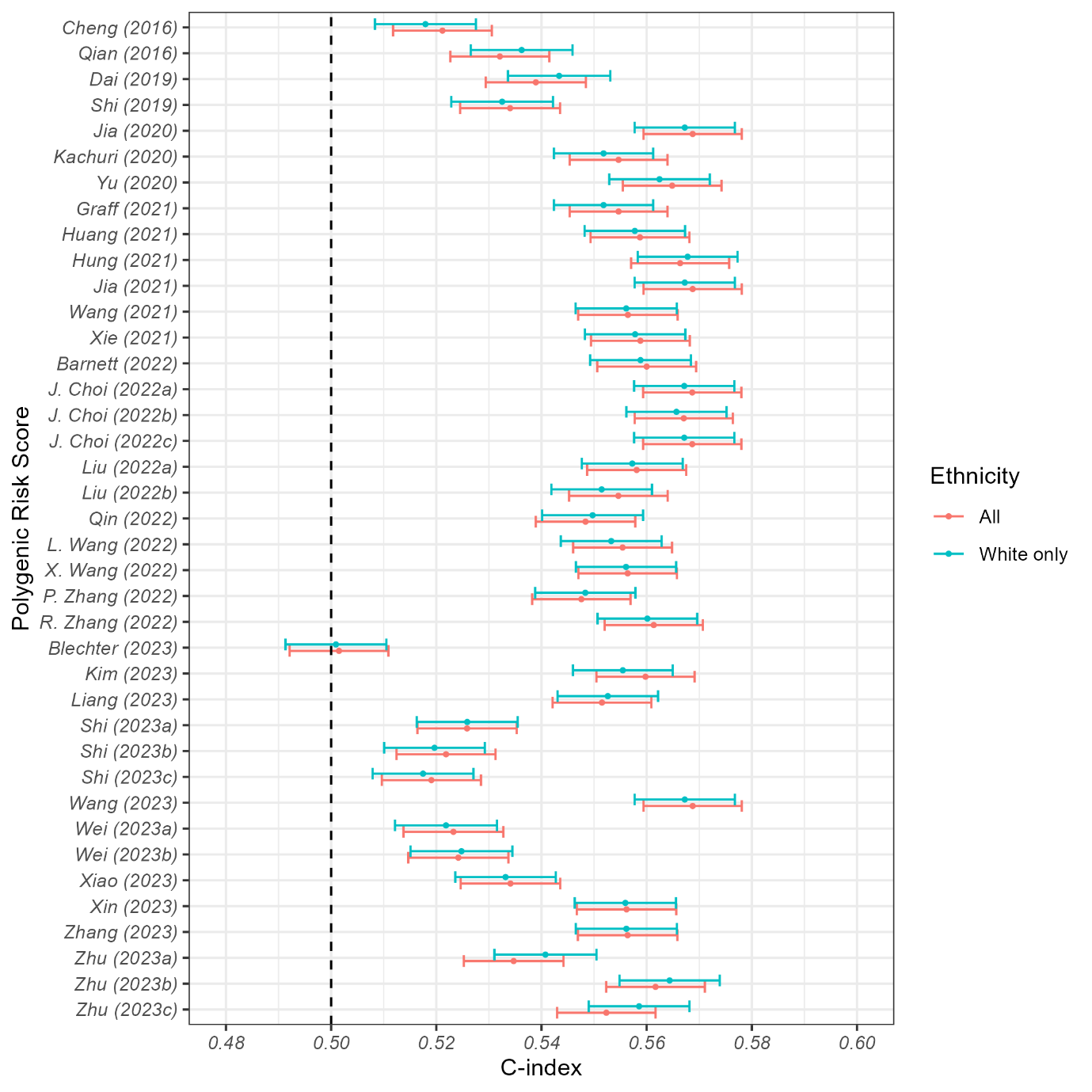

Supplement: Supplementary file 2 — Supplementary Figures [file 41416_2025_3330_MOESM2_ESM.docx]
